# Analysis of carbohydrates and glycoconjugates by matrix‐assisted laser desorption/ionization mass spectrometry: An update for 2021–2022

**DOI:** 10.1002/mas.21873

**Published:** 2024-06-24

**Authors:** David J. Harvey

**Affiliations:** ^1^ Department of Biochemistry Oxford UK

**Keywords:** carbohydrates, glycolipids, glycoproteins, MALDI, synthesis, natural products

## Abstract

The use of matrix‐assisted laser desorption/ionization (MALDI) mass spectrometry for the analysis of carbohydrates and glycoconjugates is a well‐established technique and this review is the 12^th^ update of the original article published in 1999 and brings coverage of the literature to the end of 2022. As with previous review, this review also includes a few papers that describe methods appropriate to analysis by MALDI, such as sample preparation, even though the ionization method is not MALDI. The review follows the same format as previous reviews. It is divided into three sections: (1) general aspects such as theory of the MALDI process, matrices, derivatization, MALDI imaging, fragmentation, quantification and the use of computer software for structural identification. (2) Applications to various structural types such as oligo‐ and polysaccharides, glycoproteins, glycolipids, glycosides and biopharmaceuticals, and (3) other general areas such as medicine, industrial processes, natural products and glycan synthesis where MALDI is extensively used. Much of the material relating to applications is presented in tabular form. MALDI is still an ideal technique for carbohydrate analysis, particularly in its ability to produce single ions from each analyte and advancements in the technique and range of applications show little sign of diminishing.

**Abbreviations**
*p* (as in Gal*p*): pyranose form of sugar
2‐AB: 2‐aminobenzamide
2VP: butyl‐terminated poly(2‐vinylpyridine
A2F: core‐fucosylated biantennary, *N*‐glycan
AA: aminoacridine
AC: aminocinnoline‐3‐carboxamide
ACE2: angiotensin converting enzyme 2
ADCC: antibody‐dependent cellular cytotoxicity
AEAB: 2‐amino(*N*‐aminoethyl)benzamide
AEC: anion‐exchange chromatography
AETMA: (2‐aminoethyl)trimethylammonium chloride hydrochloride
AGE: advanced glycation end products
AGP: alpha‐1‐acid glycoprotein
Ala: alanine
ALG: mannosyltransferase (gene)
AlgL: alginate lyase
AMAC: aminoacridone
AP: aminopyridine, or atmospheric pressure, or 1‐(2‐aminoethyl)piperazine
APBA: 3‐aminophenylboronic acid
APCI: atmospheric pressure chemical ionization
apoC: apolipoprotein C
APP: amyloid‐β precursor protein
APTS: 8‐aminopyrene‐1,3,6‐trisulphonic acid
AQ: aminoquinoline
AQC: 6‐aminoquinolyl‐*N*‐hydroxysuccinimidyl carbamate
AraN: aminoarabinose
Arg: arginine
Asn: asparagine
Asp: aspartic acid
ATCC: American Type Culture Collection (bacteria)
ATD: arrival time distribution
ATP: adenosine triphosphate
ATR: attenuated total reflection
AuNPs: gold nanoparticles
BCG: Bacillus Calmette–Guérin (vaccine)
BDA: bovine serum albumin
BNDM: 1,10‐binaphthyl‐2,20‐diamine
BOA: *O*‐benzylhydroxylamine
BODIPY: boron‐dipyrromethene(4,4‐difluoro‐4‐bora‐3a,4a‐diaza‐*s*‐indacene)
BSH: benzenesulfonyl hydrazine
CA: caffeic acid
CAMLG: calcium modulating ligand (gene)
CBM: carbohydrate binding module
CD: cyclodextrin
CDG: congenital disorders of glycosylation
CE: capillary electrophoresis
Cer: ceramide
CFTR: cystic fibrosis transmembrane conductance regulator
CHCA: α‐cyano‐4‐hydroxycinnamic acid
Chit42: endochitinase 42
CHO: Chinese hamster ovary
CI: chemical ionization
CID: collision‐induced dissociation
Cit: citric acid
ClCCA: 4‐chloro‐α‐cyanocinnamic acid
CMBT: 5‐chloro‐2‐mercaptobenzothiazole
CNF: carbon fiber
CNS: central nervous system
COG6: component of oligomeric Golgi complex 6 (gene)
COPD: chronic obstructive pulmonary disease
CORA: Cellular *O*‐Glycome Reporter/Amplification
CoV: coronavirus
COV: covalent organic framework
CPH: 1‐(4‐cyanophenyl)‐4‐piperidinyl hydrazide
CPMP: carboxy‐1‐phenyl‐3‐methyl‐5‐pyrazolone
CRC: colorectal cancer
CRISPR: clustered regularly interspaced short palindromic repeats
CRM: cross‐reacting material
CSDB: Carbohydrate Structure Database
CSF: cerebrospinal fluid
CTA: 2‑cyano‐3‐(2‐thienyl)acrylic acid
CTD: charge‐transfer dissociation
CuACC: Copper‐catalysed 1,3‐dipolar azide‐alkyne cycloaddition
CV: coefficient of variation
CZE: capillary zone electrophoresis
Da: Dalton
DABP: 3,4‐diaminobenzophenone
DAN: 1,5‐diaminonaphthalene
DBA: 4‐(dimethylamino)phenylboronic acid
DBD: dielectric barrier discharge
DCLK1: doublecortin like kinase 1
DC‐SIGN: dendritic cell‐specific ICAM3‐grabbing nonintegrin
DCTB: 2‐[4‐*tert*‐butylphenyl‐2‐methylprop‐2‐enylidene]‐malonitrile
DESI: desorption electrospray ionization
DHA (or DHAP): 2,5‐dihydroxyacetophenone
DHB: dihydroxybenzoic acid (2,5‐isomer unless otherwise stated)
DIUTHAME: desorption ionization using through‐hole alumina membrane
DMA: dimethylamine
DMABA: 4‐dimethylaminobenzaldehyde
DMAPA: *N,N*‐dimethylamino‐*p*‐phenylenediamine
DMCA: 3,4‐dimethoxycinnamic acid
DMDT: *N*,*N*‐dimethylpropylenetriamine
DMEN: *N*,*N*‐dimethylenediamine
DMHA: *N,O*‐dimethylhydroxylamine
DMPA: 3‐(dimethylamino)‐1‐propylamine
DMSO: dimethylsulfoxide
DMT‐MM: 4‐(4,6‐dimethoxy‐1,2,3‐triazil‐2‐yl)‐4‐methylmorpholinium chloride
DNA: deoxyribonucleic acid
DOSG+: derivatization of sialylated glycopeptides plus
DP: degree of polymerization
DSPE: 1,2‐distearoyl‐*sn*‐glycero‐3‐phosphoethanolamine
DTT: 1,4‐dithiothreitol
EAD: electron‐activated dissociation
ECD: electron‐capture dissociation
EDC: 1‐ethyl‐3‐(3‐dimethylaminopropyl)carbodiimide
EDD: electron detachment dissociation
EDMA: ethylene glycol dimethacrylate
EDTA: ethylenediamine tetra‐acetic acid
EED: electronic excitation dissociation
EGF: epidermal growth factor
EGFR: epidermal growth factor receptor
EI: electron ionization (impact)
EIEIO: electron‐impact excitation of ions from organics
EMBL: European Molecular Biology Laboratory
Endo: endoglycosidase
EPO: erythropoietin
EPS: exopolysaccharide
ER: endoplasmic reticulum
ESI: electrospray ionization
EThcD: electron‐transfer/higher‐energy collision dissociation
EtN: ethanolamine
*f* (as in Gal*f*): furanose form of sugar
FAB: fast atom bombardment
FAIMS: high‐field asymmetric waveform ion mobility spectrometry
Fc: fragment (crystallisable) region of IgG
FFPE: formalin‐fixed and paraffin‐embedded
FLAT: fast lipid analysis technique
FLR: fluorescence
FRET: fluorescence resonance energy transfer
Fru: fructose
FT: Fourier‐transfer
Fuc: fucose
FUT: fucosyltransferase
FWHM: full width at half maximum
GADS: Glycopeptide Abundance Distribution Spectra
GAG: glycosaminoglycan
Gal: galactose
GalA: galacturonic acid
GALAXY: Glycoanalysis by the Three Axes of MS and Chromatography
GalN: galactosamine
GalNAc: *N*‐acetylgalactosamine
GAQ: glucosylated aminoquinoline
GC/MS: combined gas chromatography/mass spectrometry
GDP: guanosine diphosphate
GLC: gas‐liquid chromatography
Glc: glucose
GlcA: glucuronic acid
GlcNAc: *N*‐acetyl glucosamine
GLP: glucagon‐like peptide
Glu: glutamine
GM3: ganglioside (αNeu5Ac‐(2→3)‐β‐d‐Gal*p‐*(1→4)‐β‐d‐Glc*p‐*(1→1)Cer)
GO: graphene oxide
GOS: galactooligosaccharide
GSL: glycosphingolipid
HA: hyaluronic acid
HABA: 2‐(4‐hydroxyphenylazo)benzoic acid
Hb: haemoglobin
HBA: 3‐hydrazinobenzoic acid
HCD: higher‐energy collisional dissociation
HCQ: hydroxychloroquine
HDX: hydrogen/deuterium exchange
HEK: human embryonic kidney
HeLa: Henrietta Lacks cancer cell line
Hex: hexose
HexCer: hexosylceramide
HexNAc: *N*‐acetylhexosamine
HF: high field
HILIC: hydrophilic interaction liquid chromatography
HIV: human immunodeficiency virus
HOBt: 1‐hydroxybenzotriazole
HOIL‐1: heme‐oxidized IRP2 ubiquitin ligase 1
HPA: hydroxypyridine‐2‐carboxylic acid
HPAEC: high performance anion exchange chromatography
HPLC: high‐performance liquid chromatography
HQ: 2‐hydrazinoquinoline
HRP: horseradish peroxidase
HSA: human serum albumin
IC: ion chromatography
ICR: ion cyclotron resonance
IDA: iminodiacetic acid
IFMALDI: intensity‐fading matrix‐assisted laser desorption ionization
IGF: insulin‐like growth factor
IgG(M): immunoglobulin G(M)
IM: ion mobility
IMAC: immobilized metal affinity chromatography
INLIGHT: Individuality Normalization when Labeling with Isotopic Glycan Hydrazide Tags
IR: infrared
IRP2: iron regulatory protein 2
ISD: in‐source decay
IT: ion trap
ITO: indium‐tin oxide
IUPAC: International Union of Pure and Applied Chemistry
KCP: keratinocyte‐associated protein
KDO: 3‐deoxy‐D‐*manno*‐oct‐2‐ulosonic acid
KEGG: Kyoto Encyclopedia of Genes and Genomes
KLH: keyhole limpet antigen
Ko: *glycero*‐d‐*talo*‐oct‐2‐ulosonic acid
*L*
: linear (as in *L*‐TOF)
Lac: lactose
LAESI: laser ablation electrospray ionization
LALDI‐MS: label‐assisted laser desorption/ionization mass spectrometry
LC: liquid chromatography
LDI: laser desorption/ionization
LIF: laser‐induced fluorescence
LINUCS: Linear Notation for Unique Description of Carbohydrate Sequences
LNT: lacto‐*N*‐triaose
LOD: limit of detection
LODES: logically derived sequence
LOQ: limit of quantification
LOS: lipooligosaccharides
LPMO: lytic polysaccharide monooxygenase
LPS: lipopolysaccharide
LTQ: linear trap quadrupole
MALDESI: Combined MALDI and ESI
MALDI: matrix‐assisted laser desorption/ionization
Man: mannose
ManNAc: *N*‐acetylmannosamine
MBA: methylbenzylamine
MBT: 2‐mercaptobenzothiazole
MCR: mobile colistin resistance
MEKC: micellar electrokinetic chromatography
Met: methionine
MFSD1: major facilitator superfamily domain containing 1 (protein‐coding gene)
*MGAT*
: mannosyl‐glycoprotein‐2‐beta‐*N*‐acetylglucosaminyltransferase
MIRAGE: minimum information required for a glycomics experiment
MOF: metal‐organic framework
MOGS: mannosyl‐oligosaccharide glucosidase
MPI: mannose phosphate isomerase
MPyCA: 2‐mercaptopyridine‐3‐carboxylic acid
MRI: magnetic resonance imaging
MS: mass spectrometry
MS^n^
: successive MS fragmentation n times
MSI: mass spectrometry imaging
MUC: mucin
MurNAc: *N*‐acetylmuraminic acid
MW: molecular weight
NAH: 1‐naphthaleneacethydrazide
NAO: neoagarooligosaccharide
NAPA: (silicon) nanopost arrays
NAT: natural
NCBI: National Center for Biotechnology Information
NEDC: *N*‐(1‐naphthyl) ethylenediamine dihydrochloride
NETD: negative electron transfer dissociation
Neu5Ac: *N*‐acetylneuraminic acid
Neu5Gc: *N*‐glycolylneuraminic acid
NIMS: nanostructure‑initiator mass spectrometry
NK: natural killer
NMCR: nonmobile colistin resistance
NMR: nuclear magnetic resonance
NSI: nanoelectrospray
P_4_HZD: (4‐hydrazidebutyl)triphenylphosphonium bromide
P2VP: butyl‐terminated poly(2‐vinylpyridine
PAD: pulsed amperometric detection
PAGE: polyacrylamide gel electrophoresis
PAMAM: poly(amidoamine)
PAN: polyacrylonitrile
PAPAN: 2‐phenyl‐3‐(*p*‐aminophenyl)acrylonitrile
PC: phosphorylcholine
PEG: polyethylene glycol
Pen: pentose
PET: polyethylene terephthalate
PEtN: phosphatidylethanolamine
PGC: porous graphitic carbon
PMM: phosphomannomutase
PMP: 1‐phenyl‐3‐methyl‐5‐pyrazolone
pNA: *para‐*nitroaniline
PNGase: peptide‐*N*‐glycosidase
PSA: prostate‐specific antigen
PSD: postsource decay
PSSE: poly‐synchronous surface extraction
PTM: posttranslational modification
PVDF: polyvinylidene fluoride
PVK: *N*‐vinylcarbazole
PYAB: 2‐amino‐*N*‐(prop‐2‐yn‐1‐yl)benzamide
PyAOP: (7‐azabenzotriazol‐1‐yloxy)tripyrrolidinophosphonium hexafluorophosphate
Q: quadrupole
*R*
: reflectron (as in *R*‐TOF)
RBC: red blood cells
RBD: receptor‐binding domain
REMPI: resonance enhanced two‐photon ionization
RF: radio frequency
Rha: rhamnose
RNase: ribonuclease
RP: reversed phase
RSD: relative standard deviation
SA: sinapinic acid
SALDI: surface‐assisted laser desorption/ionization
SALSA: sialic acid linkage‐specific alkylamidation
SARS: severe acute respiratory syndrome
SDC: sodium deoxycholate
s‐DHB: super DHB (DHB plus 2‐hydroxy‐5‐methoxybenzoic acid)
SDS: sodium dodecyl sulfate
SEC: size‐exclusion chromatography
Ser: serine
SETs: surface energy traps
SICRIT: soft ionization by chemical reaction in transfer
SIL: stable isotope label
SIMS: secondary ion mass spectrometry
SK3: small conductance calcium‐activated potassium channel 3
SLC: solute carrier
SLGO: single‐layer graphene oxide
SLIM: structures for lossless ion manipulation
SNFG: symbolic nomenclature for glycans
SPE: solid‐phase extraction
TAG: Toolbox Accelerating Glycomics
TEA: trimethylamine
TFA: trifluoroacetic acid
THAP: 2,4,6‐trihydroxyacetophenone
Thr: threonine
TIMS: trapped ion mobility spectrometry
TLC: thin‐layer chromatography
TLR: toll‐like receptor
TMS: trimethylsilyl
Tn: Thomsen Friesenreich (antigen)
TOF: time‐of‐flight
TSG: *N*‐(3‐triethoxysilylpropyl)gluconamide
TWIMS: travelling wave ion mobility spectrometry
Tyr: tyrosine
UDP: uridine diphosphate
UltraGIG: Ultrafast Glycoprotein Immobilization for Glycan extraction
UPLC: ultra‐performance liquid chromatography
UV: ultraviolet
VPA: vinylphosphonic acid
VPBA: 4‐vinylbenzeneboronic acid
WAX: weak anion exchange
Xyl (or X): xylose
YAG: yttrium aluminium garnet
YLF: yttrium lithium fluoride
ZIC: zwitterionic

## 1 INTRODUCTION

This review is a continuation of the 11 earlier ones in this series (Harvey, 1999, 2006, 2008, 2009, 2011, 2012, 2015, 2017, 2018, 2021, 2023) on the application of matrix‐assisted laser desorption/ionization (MALDI) mass spectrometry to the analysis of carbohydrates and glycoconjugates. It is intended to bring the coverage of the literature to the end of 2022 and includes papers with cover dates of 2021 and 2022 (as well as a few papers that were missed in earlier reviews). Papers published on preprint servers are not included because these have not been peer reviewed. Also excluded are uncorrected proofs and other versions of papers that are not fully published; these will be included in later reviews when the final versions are available. In addition, the review does not cover papers that simply report the mass of glycoproteins and those concerned with nucleotides and nucleosides. It does, however include papers describing methods for carbohydrate analysis that are relevant to MALDI analysis, even though MALDI has not been used as the analytical technique. Most applications of MALDI analysis are reported in tables with the main text being restricted to reports of analytical methods. Some papers are difficult to classify; for example, a paper on MALDI imaging of cancer biomarkers might be listed under imaging or medical applications. For reviews, the number of cited references is include to give the reader some idea of the extent of coverage.

## 2 GENERAL

Several books and review articles directly concerned with, or including MALDI analysis of carbohydrates and glycoconjugates, have been published during the review period. Those of a general nature are listed in Table 1; those concerned with specific carbohydrate types are listed in the appropriate sections.

**Table 1:** Books and general reviews on the analysis of carbohydrates with specific reference to matrix‐assisted laser desorption/ionization analysis.

Subject	Comments	Citations	References
Mass spectrometry in metabolomics	General review of mass spectrometers and applications to biomarkers, drug development, nutrition, toxicology, and forensic science	53	Amoresano and Pucci (2022)
Carbohydrate analysis by mass spectrometry	General review of different types of mass spectrometry	‐	Chizhov (2022)
Glycosylation: Methods and Protocols (Book)	Several sections: Analytical and Bioinformatics, glycoengineering, glycan networks and biomarkers. Several chapters covered in this review	‐	Davy (2022)
The value of coupling thin‐layer chromatography to mass spectrometry in lipid research (glycolipids also included)	Emphases the importance of separating components of mixtures to prevent phenomena such as ion suppression	73	Engel and Schiller (2021)
The Art of Carbohydrate Analysis (Book)	General coverage with protocols	‐	Gerwig (2021g)
Analytical techniques to study carbohydrates	Short overview of different methods including hydrolysis, separation techniques, (TLC, SEC, HPLC, PGC, anion/cation exchange chromatography, high pH, AEC), glycan labelling (with protocol), permethylation, GLC.	102	Gerwig (2021c)
Analysis of carbohydrates by mass spectrometry	Short general review with emphasis on *N*‐ and *O*‐linked glycans	76	Gerwig (2021b)
Mass spectrometry‐based techniques to elucidate the sugar code	Instrumentation, sugar types (milk sugars, *N*‐ and *O*‐glycans, GAGs, glycopeptides)	655	Grabarics et al. (2022)
Tools for mammalian glycoscience research	Primer, glycan structure and analysis, synthesis, glycan‐protein interactions, mention of MALDI imaging but not much else on MALDI.	172	Griffin and Hsieh‐Wilson (2022)
Recent advances in mass spectrometry‐based structural elucidation techniques	General review with sections on proteins and lipids as well as glycans	173	Ma (2022)
An overview of biological applications and fundamentals of new inlet and vacuum ionization technologies	Covers ESI, laserspray, vacuum laserspray, vacuum MALDI and applications	153	Trimpin et al. (2021)
Essentials of Glycobiology, Fourth edition	Main Glycobiology textbook	‐	Varki et al. (2022)
Mass spectrometry for structural elucidation and sequencing of carbohydrates	Methods for monosaccharide identification, linkage, sequence determination, applications	168	Wang, Zhao, Nie, et al. (2021)
Mass spectrometry as a crucial analytical basis for omics sciences	General review with a small section on glycomics	175	Zaikin and Borisov (2021)

## 3 THEORY

Fewer papers on the theory of the MALDI process have been published than in previous years. However, the ionization mechanism of UV‐MALDI using 2,5‐dihydroxybenzoic acid (DHB, **1**) as the matrix has been studied with two separate temperature‐dependent experiments. First, the angular resolved intensity and velocity distributions of neutrals desorbed from a solid sample of DHB with a UV laser (355 nm) were measured using a rotating quadrupole mass spectrometer. Second, the desorbed neutrals, at an angle normal to the surface, and the desorbed ions were simultaneously detected for each laser shot using a quadrupole mass spectrometer and a time‐of‐flight (TOF) mass spectrometer, respectively. Both experiments were conducted at initial temperatures of 100 and 300^o^K and the measurements were used to calculate the initial temperature dependence of the ion‐to‐neutral ratio. The results closely agreed with the predictions of the temperature‐dependent ion‐to neutral ratio using the thermal model, indicating that thermally induced proton transfer is the dominant reaction that generates initial ions from DHB in UV‐MALDI (Lin, Dyakov, et al., 2021).


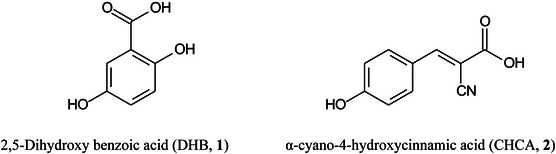


The matrix α‐cyano‐4‐hydroxycinnamic acid (CHCA, **2**) is able to protonate some compounds and form alkali metal adducts from others. Lou, Miley, et al. (2021) have provided evidence that the matrix can exist in two interconverting forms; the alkali metal (e.g., Na) adduct of the acid ([[CHCA]Na]^+^) or a protonated alkali metal salt ([[CHCA‐H+Na]H]^+^) with each version able to produce the appropriate MALDI ion.

The dynamics initiated by both chirped picosecond and femtosecond laser pulses have been investigated and three‐dimensional (3D) momentum images of desorbed ions from DHB have been obtained for the first time. The two different pulses produced a striking difference between the processes initiated by each one. The lack of initial momentum in ions produced by femtosecond pulses suggested a suppression of plume formation, which could be exploited to increase the sensitivity of the ionization process (Stewart et al., 2022).

## 4 INSTRUMENTATION

Murray (2021) has reviewed lasers used for MALDI over the 35 years that the technique has been used. The original lasers were UV fixed‐wavelength nitrogen and Nd:YAG lasers, but over the years, several additional types of laser have been introduced with wavelengths ranging from the IR to the UV and pulse widths ranging from nanoseconds to femtoseconds. Wavelength tuneable lasers have been employed in both the IR and UV ranges, and repetition rates have increased from tens of Hz to tens of kHz as MALDI has been used for mass spectrometry imaging. Dual‐pulse configurations have been implemented with two lasers directed at the target or with a second laser generating ions in the plume of desorbed material. These techniques are described in more detail in the section on MALDI imaging.

## 5 METHODS

A review on “Recent advances in combinations of TLC with MALDI and other desorption/ionization mass‐spectrometry techniques” with 82 references (Borisov et al., 2021) covers recent advances in the combined techniques.

### 5.1 Calibration

Butyl‐terminated poly(2‐vinylpyridine) (P2VP, **3**), C_4_H_9_(C_7_H_7_N)_n_H, has been reported to be an excellent external and internal mass calibrant for positive‐ion MALDI‐MS covering the range *m/z* 450–4500 with ion spacings of 105.0578 mass units ([M + H]^+^ ions). It was found suitable to calibrate a TOF mass spectrometer in linear and reflector mode, an ion mobility‐quadrupole‐time‐of‐flight (IM‐Q‐TOF) mass spectrometer, and an Fourier‐transfer ion cyclotron resonance (FT‐ICR) instrument (Gross, 2021).


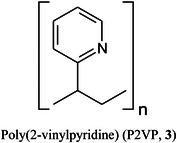


### 5.2 Ion mobility mass spectrometry

A review with 60 references on the application of ion mobility to glycomics covering free and permethylated *N*‐ and *O*‐linked glycans, glycosaminoglycans (GAGs) and glycolipids has been published in the book “New Developments in Mass Spectrometry No. 11” (Struwe, 2021). Ion mobility collision cross sections (singly‐, doubly‐, and triply‐protonated ions) and liquid chromatography retention times from 71 pyridylaminated *N* **‑**linked oligosaccharides have been published (Manabe et al., 2022).

**Figure 1:**
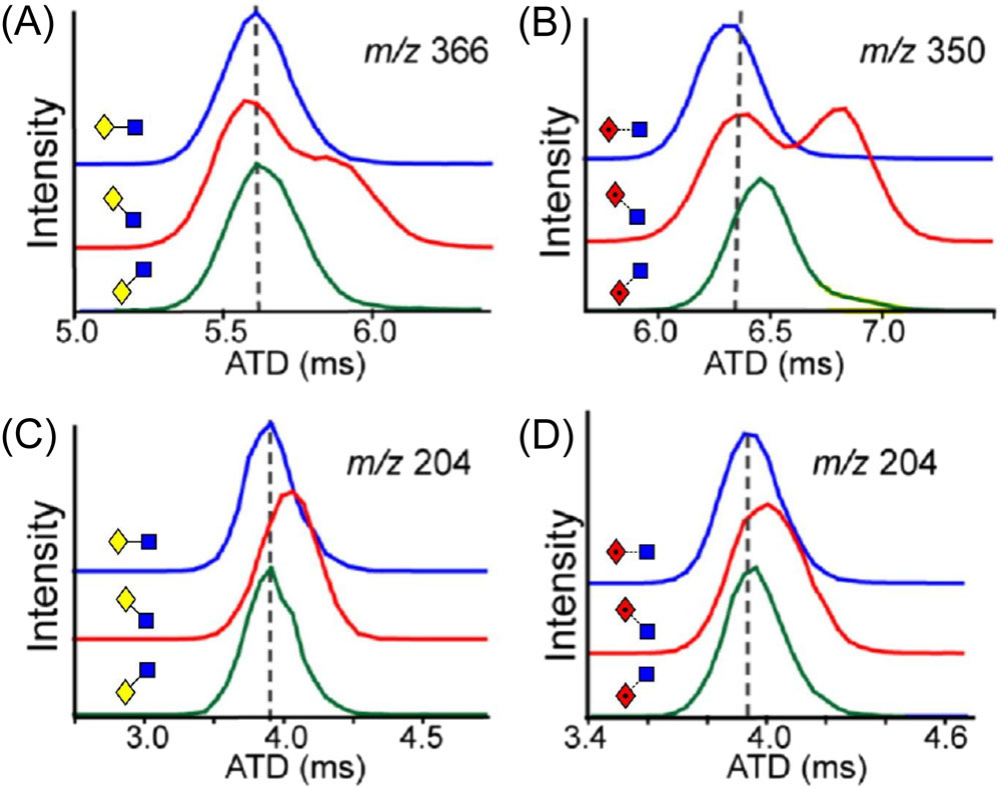
ATDs (N_2_) of (A) *m/z* 366 ([M – H_2_O]^+^) from the three isomers (**4**–**6**) of Gal‐GlcNAc, (B) The corresponding ATDs from the isomers (**7**–**9**) from Fuc‐GlcNAc. (C) *m/z* 204 ([M – H_2_O ‐ Gal]^+^) from the three isomers (**4**–**6**) of Gal‐GlcNAc, (D) The corresponding ATDs from the isomers (**7**–**9**) from Fuc‐GlcNAc. From (Mookherjee et al., 2021), with permission from the American Chemical Society. The glycan symbols have been changed to the “Oxford” system to conform with those used in the rest of the review for consistency.


Mookherjee et al. (2021) have shown that although the MS^2^ and MS^3^ spectra of Gal‐GlcNAc and Fuc‐GlcNAc in different linkages (**4**–**9**) are very similar, some differences can be observed in their ion mobility spectra. In nitrogen, although the arrival‐time distributions for the [M – H_2_O]^+^ ion from the β1→3‐ and β1→4‐ linkage isomers of Gal‐GlcNAc (**4**,**5**) were virtually identical, the β1→6‐isomer (**6**) gave two semi‐resolved peaks, clearly providing differentiation (Figure 1). Separations of the corresponding ion from Fuc‐GlcNAc (**9**) was even more pronounced. The ions formed by further loss of galactose or fucose (*m/z* 204) from the β1→6‐isomers (**6**, **9**) also gave a different ATD from the others showing that the ions formed from the different isomers had different gas‐phase structures that retained some of the original linkage information, a phenomenon termed linkage memory.

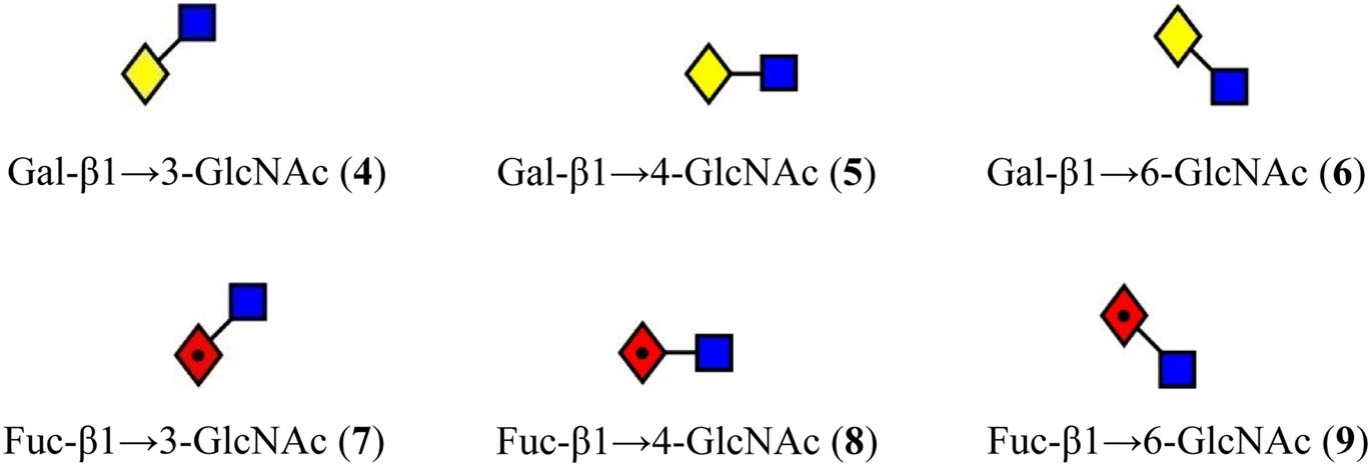


Symbols for the monosaccharides used in this review are shown below. These symbols from the so‐called “Oxford” system (Harvey et al., 2009) are used in preference to those from the more commonly used “Symbol Nomenclature for Glycans” (SNFG) system (Neelamegham et al., 2019; Varki et al., 2015) because they overcome some of the problems and inconsistencies inherent with the SNFG system.


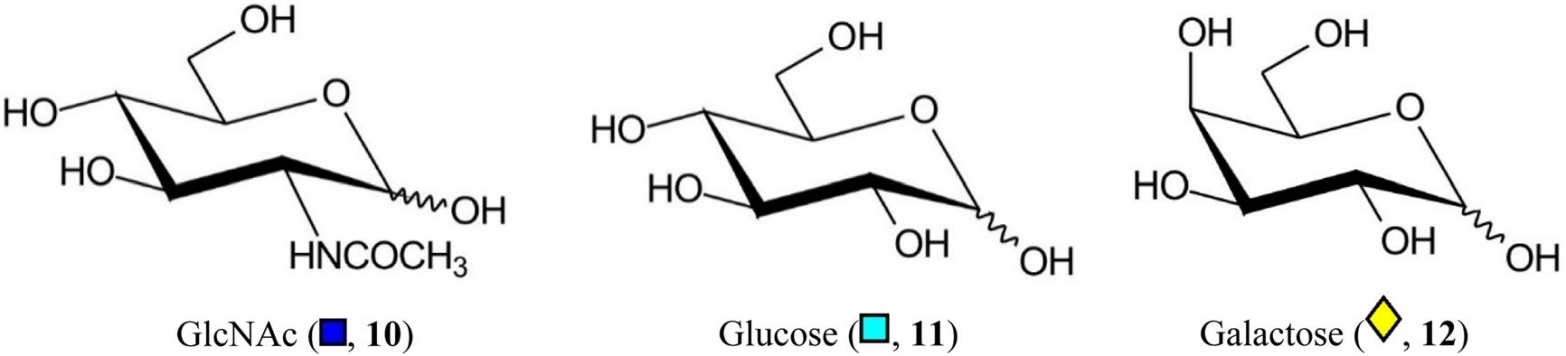



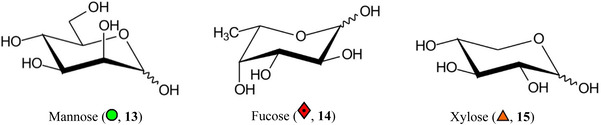



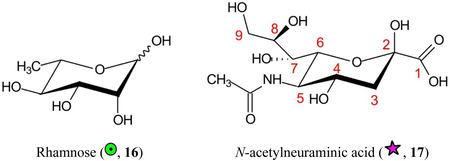


Isomer separation is a major application of ion mobility and is of particular relevance to glycomics. One application where ion mobility has had an impact is in the separation of sialic acid isomers. In one method that used a Waters travelling wave (TWIMS) instrument, it was found that ion mobility could successfully distinguish between α2→3‐ and α2→6‐linked sialic acids in complex *N*‐glycans by separation of the fragment ions Neu5Ac‐Gal‐GlcNAc (**18**, **19**) respectively following collision‐induced dissociation (CID) (Feng et al., 2021). Using the method, the authors demonstrated aberrant sialylation of haptoglobin in hepatocellular carcinoma where the ratios of α2→3‐ to α2→6‐ sialylation of seven *N*‐glycopeptides were found to be significantly altered (*p* < 0.01) in cancer (*n* = 27) compared with healthy controls (*n* = 27). Quantification was also possible with good linearity (*R* ^2^ = 0.99) with a dynamic range of two orders of magnitude and high reproducibility (coefficient of variation [CV] < 10%, *n* = 3).


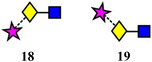


Early instruments with ion mobility cells did not possess sufficient resolution to separate many isomers but two recent instruments, the Waters cyclic‐TWIMS mass spectrometer (Giles et al., 2019) and the instrument based on lossless ion manipulation (SLIM) technology (Deng et al., 2017) produce considerably improved resolutions. Using these instruments, separation of many glycan isomers has been possible. Thus, monogalactosylated biantennary isomers (**20**, **21**) have been separated to base line with a Waters cyclic TWIMS mass spectrometer after two circuits of the cyclic mobility cell (Oganesyan et al., 2022). Further cycles produced partial separation of various conformers. The concomitant separation of conformers or anomers somewhat complicates the picture and sometimes requires separate experiments to distinguish between the two. Confirmation of nonisomeric separations was provided in the work by Oganesyan et al. by the observation of multiple peaks with the aglycosylated biantennary glycan (**22**) after eight cycles and after three cycles for the fully galactosylated glycan (**23**). Neither of these glycans should contain isomers.


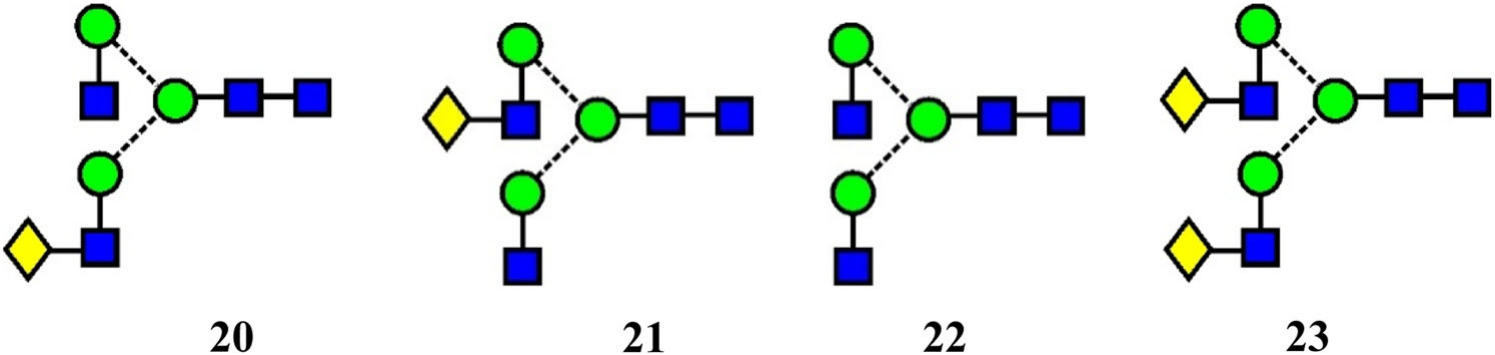


**Figure 2:**
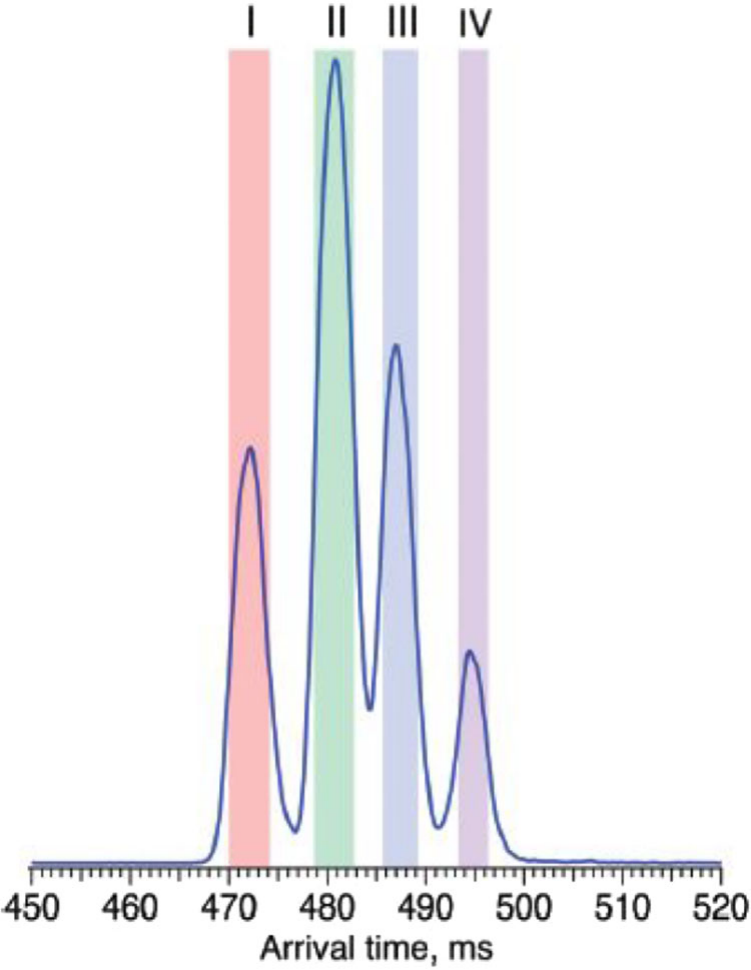
Arrival time distributions for the biantennary glycans **24** and **25** ([M + 2Na]^2+^ ions) recorded with the SLIM device. From Dyukova et al. (2021) with permission from the Royal Society of Chemistry.


Isomers of the core‐fucosylated analogues of glycans **20** and **21** (glycans 25 and 24) have been resolved to baseline with a SLIM device (60 M flight path giving an estimated resolution of about 5000), (Figure 2). Two peaks were resolved for each isomer, probably attributed to anomers (Dyukova et al., 2021).

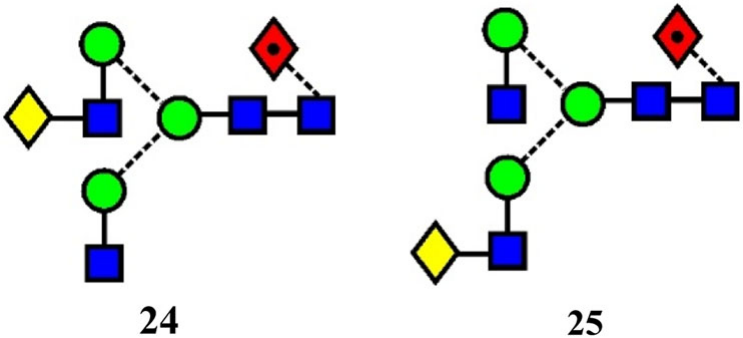


Ion mobility is proving to be a great asset to glycan analysis (Struwe, 2021). In addition to its ability to separate isomers (Gao, Li, et al., 2021; Mastellone et al., 2022), as discussed above, it provides the ability to measure collisional cross sections which are relatively instrument independent and provide an alternative to the glucose units that are familiar to most glycobiologists. In addition, it provides a method for cleaning spectra by removing extraneous ions from noisy backgrounds (Harvey, Crispin, et al., 2015), particularly when these ions are multiply charged. The technique is also invaluable for removing contaminating ions from MS/MS spectra (Harvey et al., 2016). It is expected that ion mobility will be increasingly used for glycan analysis in the coming years.

## 6 MATRICES

Reviews and general articles relating to MALDI matrices are listed in Table 2.

**Table 2:** Reviews and general articles on matrices.

Subject	Comments	Citations	References
Recent progress in the matrix for analysis of low molecular weight compounds using matrix assisted laser desorption ionization time‐of‐flight mass spectrometry	Comprehensive review. Discusses each type of matrix. In Chinese	89	Chen, Gao, et al. (2022)
Inorganic matrices assisted laser desorption/ionization mass spectrometry for metabolic analysis in biofluids	General coverage with several references to glycan analysis	89	Ding et al. (2022)
Recent advancements of carbon dots in analytical techniques	General chapter on carbon dots. Short section on MALDI	82	Gedda et al. (2022)
Diverse applications of ionic liquids: A comprehensive review	General review with short section on use of ionic liquids as MALDI matrices	258	Kaur et al. (2022)
Graphene oxide derivatives and their nanohybrid structures for MALDI analysis of small molecules	Applications mainly to amino acids, peptides, monosaccharides and small oligosaccharides	104	Kim, Kwon et al. (2021)
Nanostructured substrates as matrices for surface assisted laser desorption/ionization mass spectrometry: A progress report from material research to biomedical applications	General review including references to carbohydrates	178	Ma, Li, Li, et al. (2021)
Interfacial assembly of functional mesoporous nanomatrices for laser desorption/ionization mass spectrometry	Summarises recent advances in the fabrication strategies, properties and MALDI‐MS mechanisms of optical heterostructures based on mesoporous nanomaterials	308	Ma, Xie, et al. (2022)
MALDI Matrices for the analysis of low molecular weight compounds: Rational design, challenges and perspectives	Classic matrices, binary, hybrid and nanomaterial‐based matrices, reactive matrices, negative ion matrices	126	Qiao and Lissel (2021)

The development of new matrices continues with much of the emphasis on those for low‐mass compounds that give ions in the same region as many organic matrices. These new matrices enable molecules such as small as monosaccharides to be examined.

### 6.1 Simple organic matrices

2‑Cyano‐3‐(2‐thienyl)acrylic acid (CTA, **26**) has been reported as a new matrix for a wide variety of analytes such as peptides, lipids, polyethylene glycol (PEG), carbohydrates (β‐cyclodextrin [β‐CD, **27**], maltotriose [**28**], sugammadex [**29**], and lactose [**30**]) and glycosides (Yerra et al., 2021). Signal strengths were reported to be higher than those produced by common matrices such as DHB although peptides gave similar signals with this matrix and CHCA. As with DHB, carbohydrates gave [M + Na]^+^ and [M + K]^+^ ions.


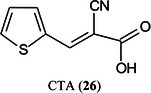



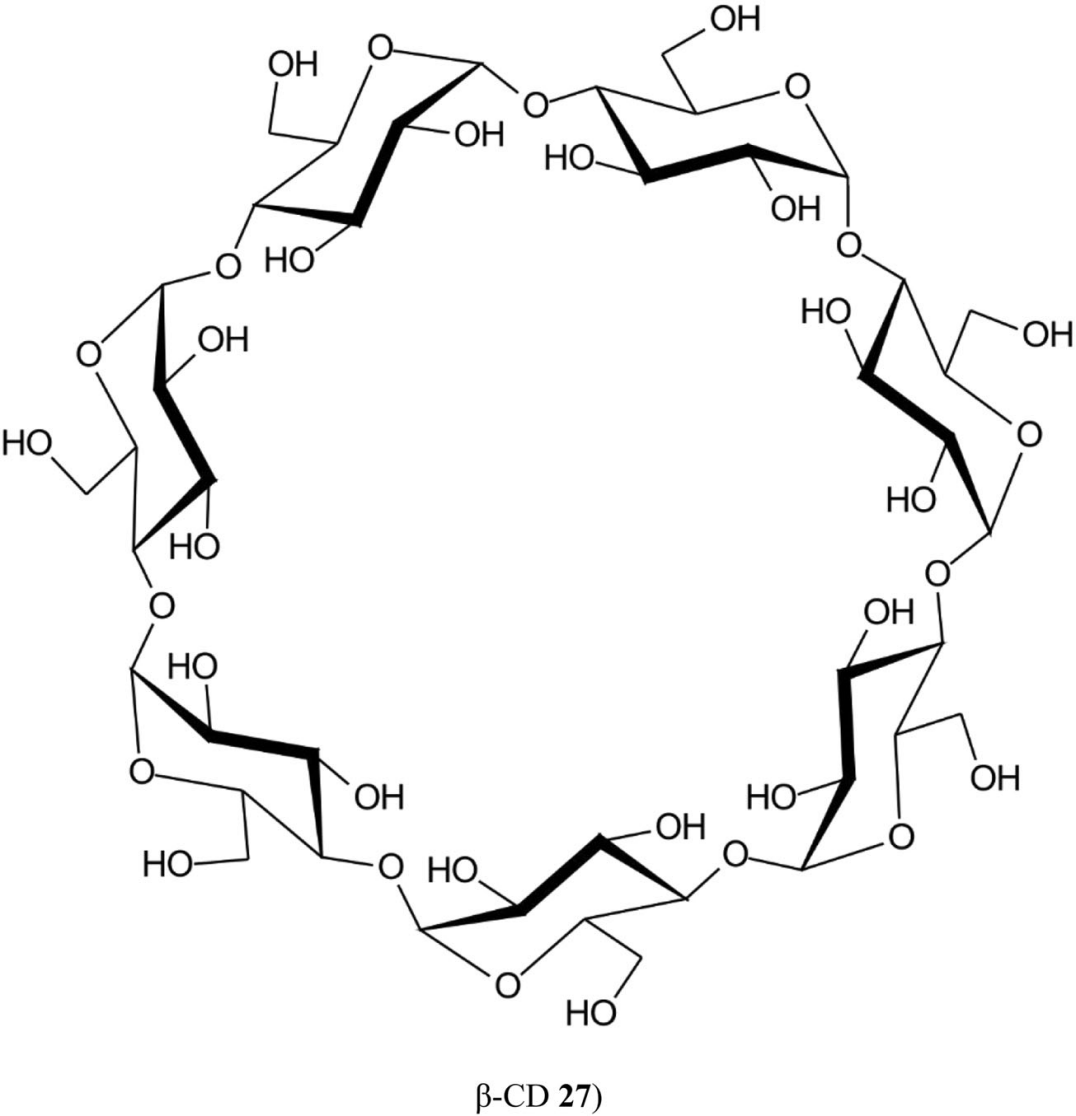



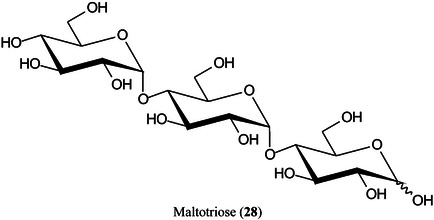



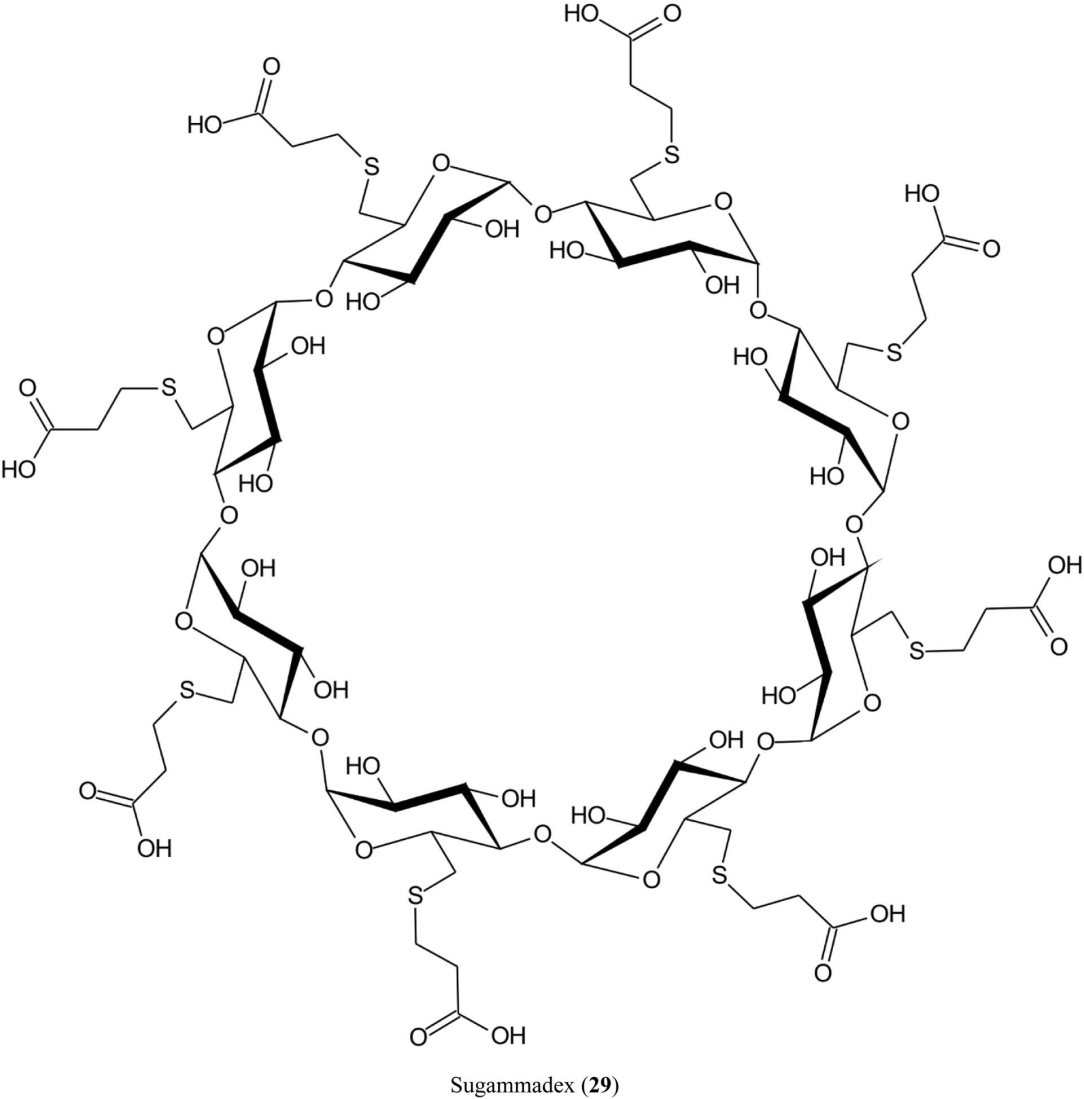



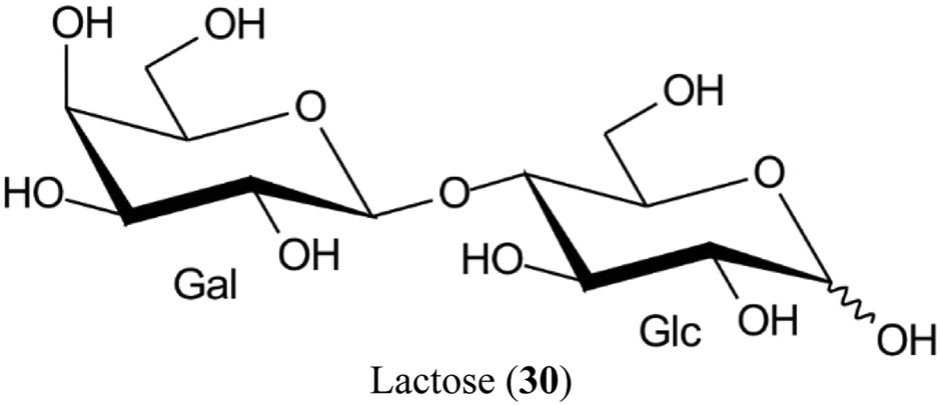


Sinapinic acid (SA), the most widely used matrix for proteins and glycoproteins, exists as two isomers: *E*‐ (**31**) and *Z*‐SA (**32**). It has long been known that *Z*‐cinnamic acid outperforms the *E*‐acids when acting as a MALDI matrix. Using ESI, MS/MS and titration experiments, and a variety of carbohydrates, De León et al. (2022) have shown that the *Z*‐isomer forms stronger gas‐phase complexes with the carbohydrates than the *E*‐isomer, thus explaining the phenomenon. Over time, the *Z*‐form isomerizes to *E*‐SA accounting for the reduction in signal strength of analytes with aged matrix samples.


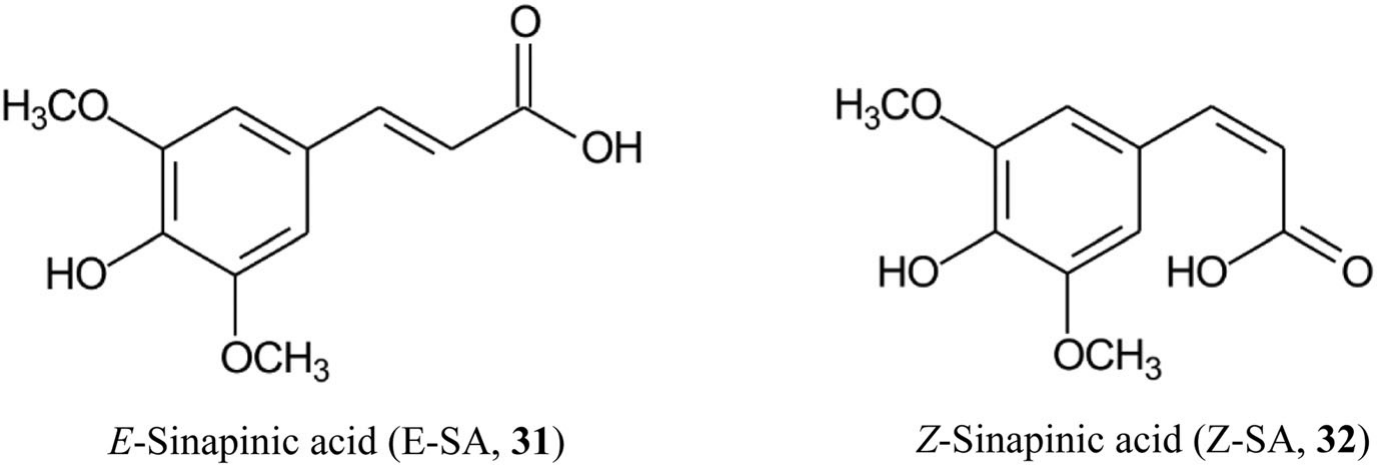


Dealkaline lignin (complex branched polymer formed mainly from *p*‐coumaryl alcohol (**33**), coniferyl alcohol (**34**) and sinapyl alcohol (**35**) has been found to be a good matrix for several types of small molecule including oligosaccharides, glycosides, esters, vitamins, amino acids, hydroxyl‐acids, and fatty acids in both positive and negative ion modes. Linear quantitative results were obtained with excellent correlation with parallel high‐performance liquid chromatographic (HPLC) analyses. The performance of lignin as a matrix was said to be due to its superior optical property and abundant conjugated structure (Zhao, Wang, Liu, et al., 2021).


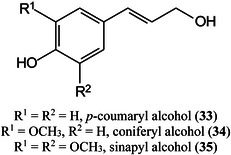


### 6.2 Binary and mixed matrices

**Scheme 1:**
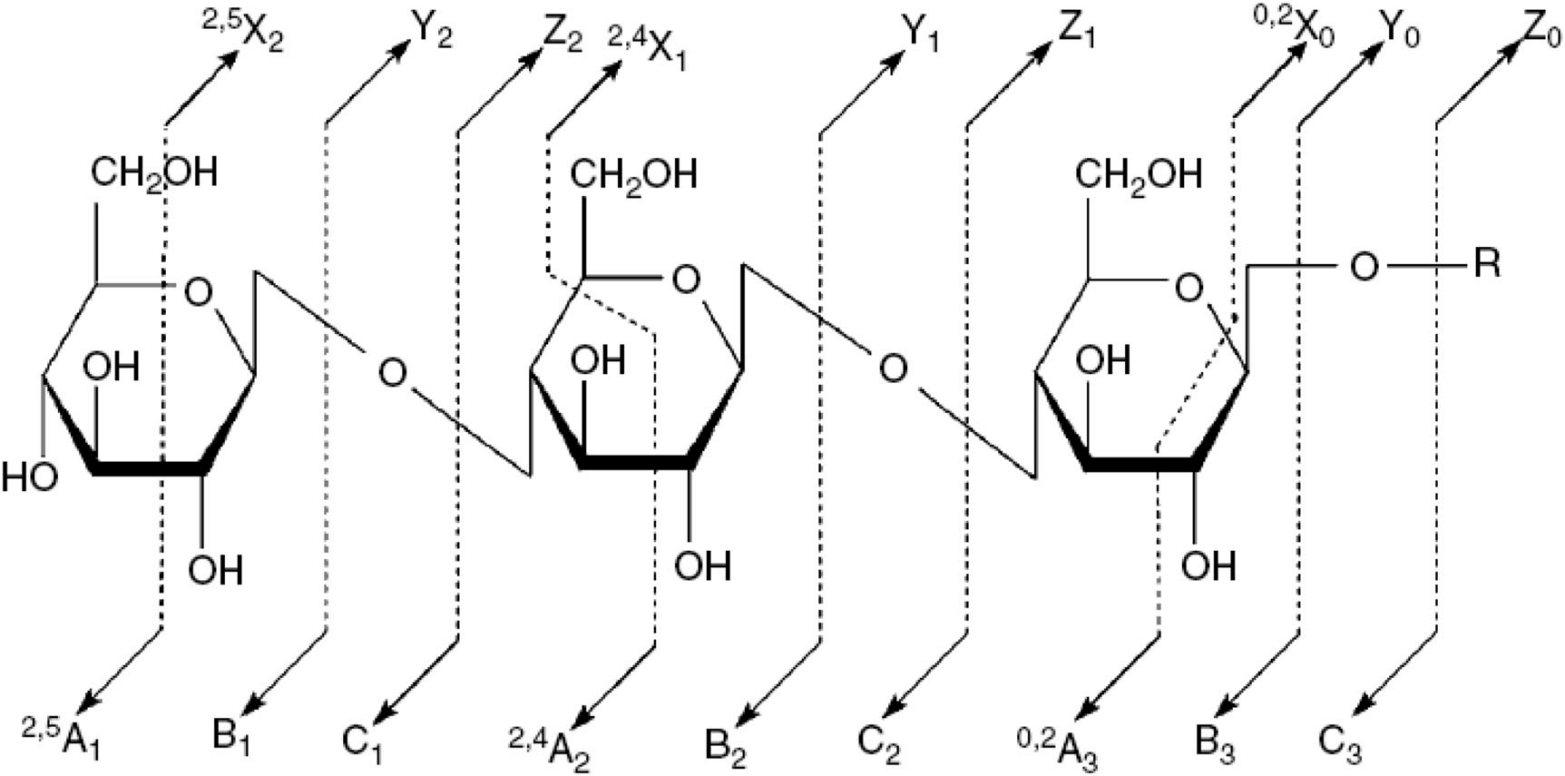
Method for naming fragment ions as devised by Domon and Costello (1988). Fragments with the charge at the nonreducing end of the molecule are designated with the letters A (cross‐ring), B and C (glycosidic) with the following subscript number indicating the position of cleavage. Corresponding ions from the reducing end are designated X, Y, and X. For the cross‐ring ions, the bonds that are cleaved are indicated by superscript numbers preceeding the lertters.


Urakami and Hinou (2022c) have developed a mixed matrix of 1,5‐diaminonaphthalene (DAN, **36**)/DHB/Na (2:10:1) and have use this to examine small glycopeptides directly. The matrix gave a more homogeneous target than DHB and promoted in‐source (ISD) fragmentation such that the glycans were released as ^0,2^A and ^2,4^A fragments from the reducing end (see Scheme 1). Further fragmentation in the TOF/TOF instrument yielded mainly glycosidic cleavage ions. Applications were to ovomucoid and egg white but some of the reported structures, high‐mannose glycans in particular, deviate from those established from known biosynthetic pathways.

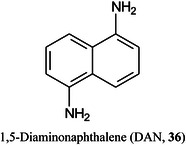


### 6.3 Ionic liquid matrices

Ionic liquid matrices present a homogeneous surface to the laser beam, thus eliminating the concept of “sweet spots” and a review with 61 references on their use for quantification of small molecules, including carbohydrates has been published by Kobylis et al. (2021). However, little is known about their properties. In one of the latest of a series of papers investigating ionicity (Kobylis et al., 2022; MacFarlane et al., 2009) have studied four truly liquid matrices (see Kobylis et al., 2019), namely CHCA/trimethylamine (TEA), ferulic acid (**37**)/TEA, 2‐(4‐hydroxyphenylazo)benzoic acid (HABA, **38)**/(α‐methylbenzylamine (α‐MBA, **39**), and 2,5‐DHB/α‐MBA), The results, particularly as shown by a Walden plot (Molar conductivity against viscosity) showed that HABA/α‐MBA was the best ionic matrix. The ionicity of the other matrices was reduced because of intermolecular interactions. It was concluded that although the tested matrices differed in iconicity, this made no difference to their auto‐ionization properties.


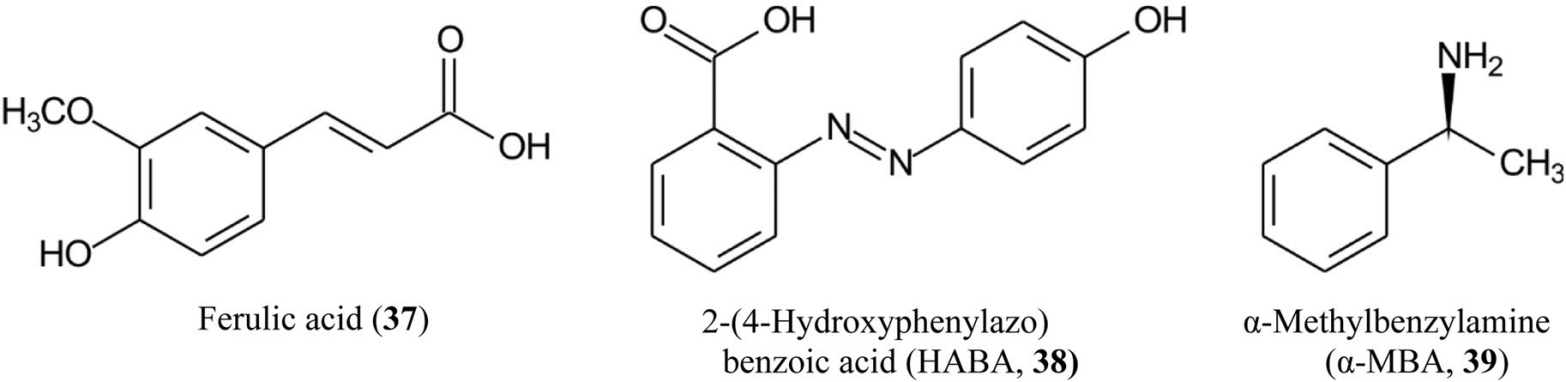


Urakami and Hinou (2022b) have analysed *N*‐glycans from ribonuclease B (RNase B) with the ionic liquid matrix DHB‐aniline‐Na and observed both molecular ([M + Na]^+^) and ^0,2^A and ^2,4^A in‐source cleavage ions from the high‐mannose glycans. Formation of peptide fragment ions were of minor relative abundance. Further LIFT fragmentation was used to characterise the glycans.

### 6.4 Carbon‐based matrices

Carbon fiber (CNF), prepared by carbonization of electrospun polyacrylonitrile (PAN) fibers, has proved to be an excellent matrix for small molecules, especially carbohydrates such as glucose (**11**), sorbitol (**40**), mannitol (**41**) and sucrose (**42**) (Chae et al., 2021). The matrix exhibited a high salt tolerance and high sensitivity in both positive ([M + Na]^+^ ions) and negative ([M – H]^‐^ ions) ionization modes. A linear response for sucrose was recorded over the range 0–500 pmol allowing quantitation. Other compounds that were successfully analysed included amino acids and synthetic polymers such as PEG.


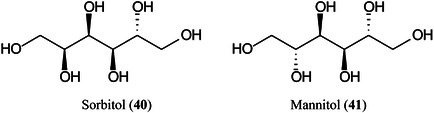



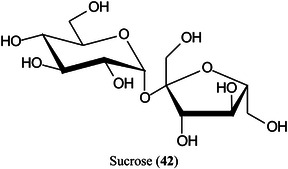


Nitrogen and boron codoped carbon nanofiber has also been reported as a good matrix for a range of compounds such as carbohydrates, amino acids, and polymers. This matrix showed high signal to noise ratio, excellent salt‐tolerance and homogeneous ion distribution and was reported to be superior to CHCA and to a C nanofiber matrix acting as a control (Zhao, Wang, Zhao, et al., 2021).

Highly curved onion‐like carbon nanoparticles have been synthesized from soot collected on a glass slide from the centre of a candle flame. The particles had a large surface area and good hydrophilicity. They exhibited superior performance for the detection of xylose (Xyl, **15**), glucose (**11**), maltose (**43**) monohydrate, and raffinose (**44**) pentahydrate in positive‐ion mode with low background noise, a homogeneous target, excellent reproducibility, good salt‐tolerance and high sensitivity compared to traditional matrices such as CHCA. Using the matrix, the authors developed a quantitative assay for glucose in rat serum (Zhao, Zhao, et al., 2022).


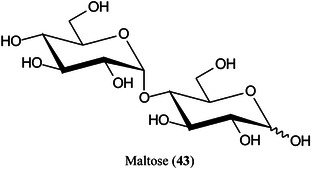



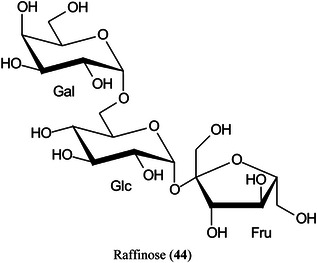


### 6.5 Nanoparticles and related substances

As these compounds, mainly consisting of metal and metal oxides, lack the organic structure of traditional matrices, they are useful for examination of small molecules. “Green metal” nanoparticles have been prepared from the leaves of *Cudrania tricuspidata* and silver nitrate and used as a MALDI matrix for various small molecules (MW<500 Da) such as glucose, lysine, sucrose (**42**) and glutamic acid (Sharma, Rejeeth, et al., 2021). A low detection limit (4–20 nmol) was reported with peaks of higher intensity than those obtained using conventional CHCA. Background noise was low. By using the matrix, the authors were able to detect 13 low molecular weight metabolites in human healthy serum samples and another distinct 18 low molecular weight compounds in pancreatic cancer serum samples.

Zhao, Ma, et al. (2022) have prepared sandwich‐like gold nanoparticles@mesoporous silica nanocomposite@silver nanoparticles (Au@MSN@Ag) by a layer‐by‐layer super‐assembly strategy as a novel matrix for the quantitative detection and enrichment of small biomolecules. The sandwich‐like nanospheres were said to form a unique plasma resonant cavity that effectively absorbed the laser energy, while the homogeneous mesoporous structure of the nanoparticles could lock the analyte. Compared to traditional matrices, Au@MSN@Ag produced a low background, a wide application range, high sensitivity, good high salt and protein tolerance, and good stability. As an example of its performance, the detection limit of glucose was 5 fmol, and showed a good linear relationship in the range of 1−750 μg/mL. Gold nanoparticles coated with 2‐mercaptopyridine‐3‐carboxylic acid (MPyCA, **45**) has also proved to be an effective matrix for small molecules, including glucose and has been reported to give stronger signals from this compound than when ionized by CHCA (Kakuta et al., 2022).


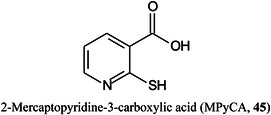


Palladium nanoparticles decorated thiol‐functionalized metal organic framework (MOF) nanocomposite (UiO‐66‐(SH)_2_@Pd NPs) has been synthesised as a matrix for analysis of di‐, tri‐ and tetra‐saccharides. The ionization efficiency was significantly improved over that of conventional matrices owning to the synergistic effect of MOF and Pd nanoparticles. By combining laser desorption‐LIFT‐TOF/TOF, 24 oligosaccharide isomers including disaccharides, trisaccharides and tetrasaccharides, were effectively differentiated. In addition, the relative quantification curves for isomeric oligosaccharides were established with good linear correlations. The method was successfully applied to the identification and quantification of sucrose and maltose in three batches of Asian and American ginseng respectively (Luo, Zhao, et al., 2022).

Among metal oxides, Fe_3_O_4_ nanoparticles have been reported as excellent matrices for a number of small molecules such as d,l‐pyroglutamic acid, d,l‐aspartic acid, l‐proline, l‐phenylalanine, sucrose, raffinose, and the triglycerides tripalmitin and triolein in positive ion mode (Zhao, Xu, Gong, et al., 2021). The matrix increased the MS peak strength and reduced the background noise compared with conventional matrices. The relative standard deviations in in‐spot and spot‐to‐spot repeatability were less than 3.2% and 6.0%, respectively and the linear correlation coefficients between MS peak intensity and concentrations were no less than 0.997 in the concentration range of 0.05–1.0 mg/mL.

TiO_2_ Nanoparticles have been reported to be a promising matrix for a variety of lipids including LacCer (**47**) (Peng, Zhang, et al., 2021). To prepare the target, the sample was mixed with the matrix solution in ethanol and NaCl was added if needed. The mixture was added to the target and allowed to evaporate. Strong signals were produced in both positive and negative ion modes with few interfering signals. P25 Titania, another TiO_2_ product has been shown to provide better ionization of small metabolites than either DHB or CHCA (Chen, Zhang, Wu, et al., 2022). Ten peaks were observed from a standard metabolite mixture consisting of glutamine acid, methionine, histidine, phenylalanine, taurine, aspartic acid, mannitol, and glucose whereas only two and five peaks were observed from DHB and CHCA respectively. Furthermore, the two matrices showed abundant matrix‐related peaks in the metabolite region. The method was used to examine metabolic patterns in membranous nephropathy. The material (Ti_3_C_2_(OH)_*x*_), synthesised from the new two‐dimensional material MXene, has also shown excellent properties as a matrix for small molecules such as mono‐ and disaccharides and amino acids. Furthermore, the material showed good storage properties and was stable for at least 8 months (Li, Ma, et al., 2022).


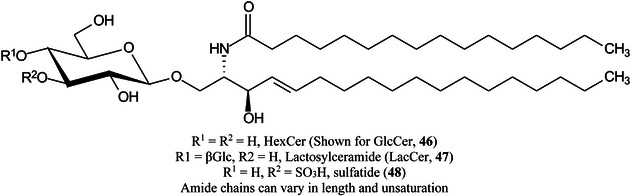


Of a series of MOFs synthesised by Ma, Yang, et al. (2022) the maltose‐functional MOF MIL‐101‐maltose has proved to be the best. Glucose was included in five test compounds and the matrix provided ultrahigh ionization efficiency, free of matrix background, uniform crystallization, good dispersibility, a short analysis time, strong salt tolerance (500 mM NaCl), and satisfactory reproducibility. The matrix was used for serum glucose determination and successfully identified diabetic patients from healthy controls.

### 6.6 Matrices for negative ion mode

Neutral compounds tend not to produce ions in negative ion mode with many traditional matrices although compounds such as norharmane (**49**) are effective. Acidic compounds such as carboxylic acids perform better but a number of more specialised matrices have been introduced. Among these are the deprotonating matrices 4‐dimethylaminobenzaldehyde (DMABA, **50**), *N,N*‐dimethylamino‐*p*‐phenylenediamine (DMAPA, **51**), and 3‐aminoquinoline (3‐AQ, **52**) (Krivosheina et al., 2021) which give limits of detection in the low ng/mL range with DMABA producing the strongest signals from acids and a number of neutral compounds.


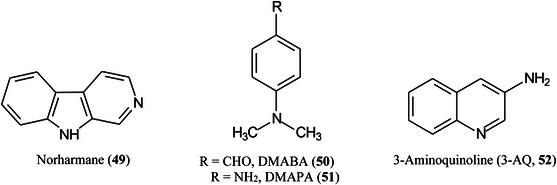


A disadvantage of metal‐containing nanoparticles is the unwanted appearance of metal adducts in positive ion mode. To overcome this disadvantage Tang et al. (2022) have investigated a bismuth oxide‐graphene oxide (Bi_2_O_3_‐GO) semiconductor nanomaterial for analysis of small molecules. The matrix was characterized using conventional methods and its performance for the detection of small molecules was compared with traditional matrices (e.g., CHCA, DHB, 9‐aminoacridine [9‐AA, **53**] and graphene oxide [GO]). The results showed that the negative ion spectra of small molecules were free of matrix‐related interferences, and possessed good signal intensity and repeatability. Application of Bi_2_O_3_@GO to the quantitative determination of glucose in human serum and soft drinks confirmed that the hybrid matrix could also be applied to complex samples. Conclusions drawn from the experimental results, computational chemistry calculations, and previous studies, suggesting that interfacial photogenerated thermal electron transfer and capture are key processes in the LDI mechanism. Other matrices for negative ion work (Veličković, Sharma, et al., 2022) are discussed in the section on MALDI imaging.


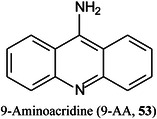


### 6.7 Matrices for dual‐polarity investigations

Most matrices for MALDI‐TOF MS of small‐molecules are only suitable for either positive or negative ion mode and, with the exception of carbon‐based nanomaterials, are not suitable for operation in dual‐ion mode. To achieve this property, two materials, poly *N*‐vinylcarbazole (PVK, **54**) and single‐layer graphene oxide (SLGO), have recently been combined to provide both positive‐ and negative‐ion‐mode spectra of amino acids, nucleic acid bases, environmental endocrine disruptors, antibiotics, and various small molecules such as sugars (Chen, Wang, Luo, et al., 2022). The lone‐pair electrons on the nitrogen atom of PVK can serve as a Lewis base with strong electron‐donation effects, which is favourable for production of negative ion spectra. The surface of SLGO, which contains many oxygen atoms in carboxyl and hydroxyl groups that act as Lewis acids provides favourable protonation sites for positive ion mode detection. The PVK/SLGO combined matrix was compared with PVK, SLGO, and the commercially available matrices 9‐AA and CHCA where the tested analytes were shown to give strong signals in both ion modes with the new matrix. Limits of detection ranged from 0.1 to 0.0001 and 0.01 to 0.0001 mg/mL in the positive and negative ion modes, respectively.


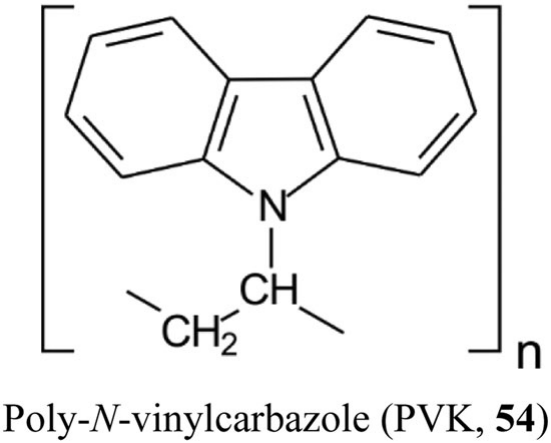


### 6.8 Combined matrices and derivatization agents

**Scheme 2:**
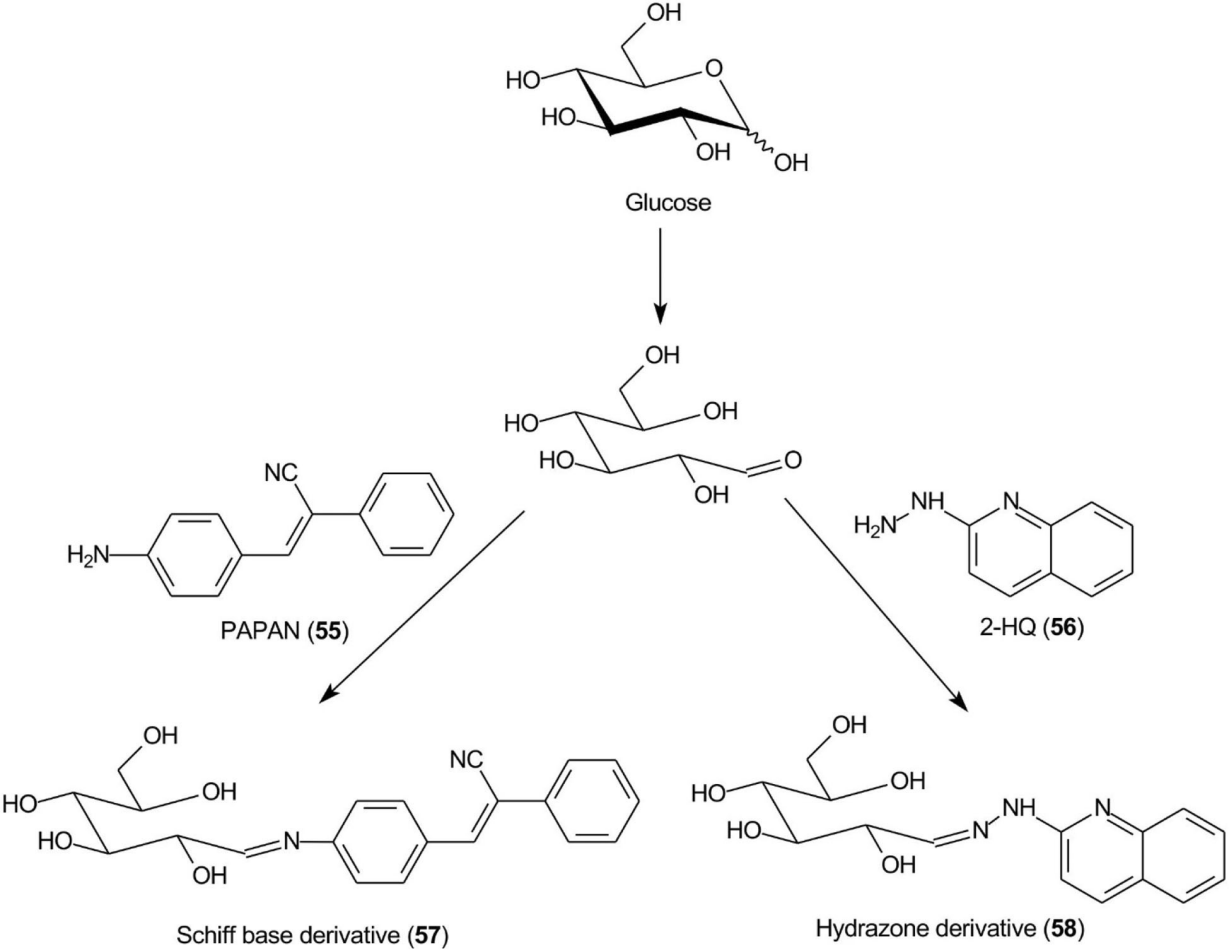
Derivatization of glucose with PAPAN (**55**, Schiff base) and 2‐HQ (**56**, hydrazone).

These compounds are used to form derivatives at the reducing end of the analyte molecules, sometimes directly on the MALDI plate before analysis. 2‐Phenyl‐3‐(*p*‐aminophenyl)acrylonitrile (PAPAN, **55**, Scheme 2) has been developed as one of these matrices (Ling et al., 2019). It forms a Schiff base (**57**) with the carbohydrates and the derivatives have been claimed to show increased ionization efficiency and reproducibility than DHB. Sample preparation involved mixing the acidified sample and PAPAN and heating at 60^o^C for 1 h and depositing the mixture directly onto the MALDI plate. The matrix was used to investigate maltooligosaccharides from beer (Ling, Jiang, et al., 2021).

2‐Hydrazinoquinoline (2‐HQ, **56**), forming a hydrazone derivative (**58**, Scheme 2), has also been used as a dual‐mode matrix. Samples were reacted with 2‐HQ in methanol containing 5% acetic acid for 10 min at 35^o^C, following which the solution was deposited onto the MALDI target and allowed to air‐dry. Use of the resulting glycan hydrazones were claimed to provide an enhancement in detection sensitivity of 10 and 100 fold over that provided by 3‐AQ or DHB respectively. The matrix worked in both positive and negative ion modes (neutral glycans as Cl^‐^ adducts) (Lin, Xiao, et al., 2021). 4‐Hydrazinoquinazoline (**59**), also introduced by the same research group (Ling, Yu, et al., 2021) and used in a similar fashion, was claimed to give a 100‐fold increase in sensitivity for maltoheptaose and a 30 fold improvement for the triantennary *N*‐glycan (**60**) compared with conventional matrices such as DHB. The matrix also formed homogeneous crystals and, thus, showed good shot‐to‐shot reproducibility. It was successfully applied to the analysis of *N*‐glycans released from ovalbumin, bovine fetuin and human serum.


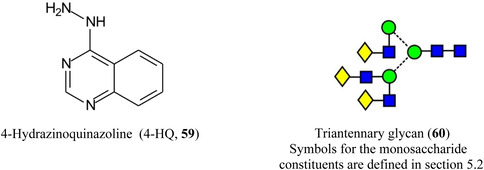


A mixture of 3‐AQ (**52**) and CHCA has been used to provide on‐target derivatization of various carbohydrates in an attempt to improve sensitivity (Wang, Zhao, Nie, et al., 2022). CHCA and 3‐AQ were mixed with ammonium dihydrogen phosphate and the carbohydrate (maltooligosaccharides and cyclodextrins), were deposited onto the MALDI plate and heated at 60^o^C for 1 h. MALDI‐TOF/TOF spectra were recorded and the sugars appeared as phosphate adducts in negative ion mode. Improved detection limits were achieved and the 3‐AQ derivatized glycans gave informative fragmentation spectra with A‐type cross‐ring cleavage ions providing useful linkage information.

Another combination of derivatization reagent and matrix is *O*‐benzylhydroxylamine (BOA, **61**) mixed with DHB and a small amount of a sodium salt (Barada & Hinou, 2022). Derivatization suppressed in‐ and post‐source fragments from the reducing end of the glycans and was reported to give excellent results from both *O*‐ and *N*‐linked glycans. MALDI targets were prepared simply by mixing the sample and reagents with sodium bicarbonate and spotting onto AnchorChipTM 400/384 TF plates.


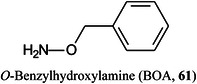


Other new matrices are described in the section on MALDI imaging.

### 6.9 Matrix‐free methods

**Scheme 3:**
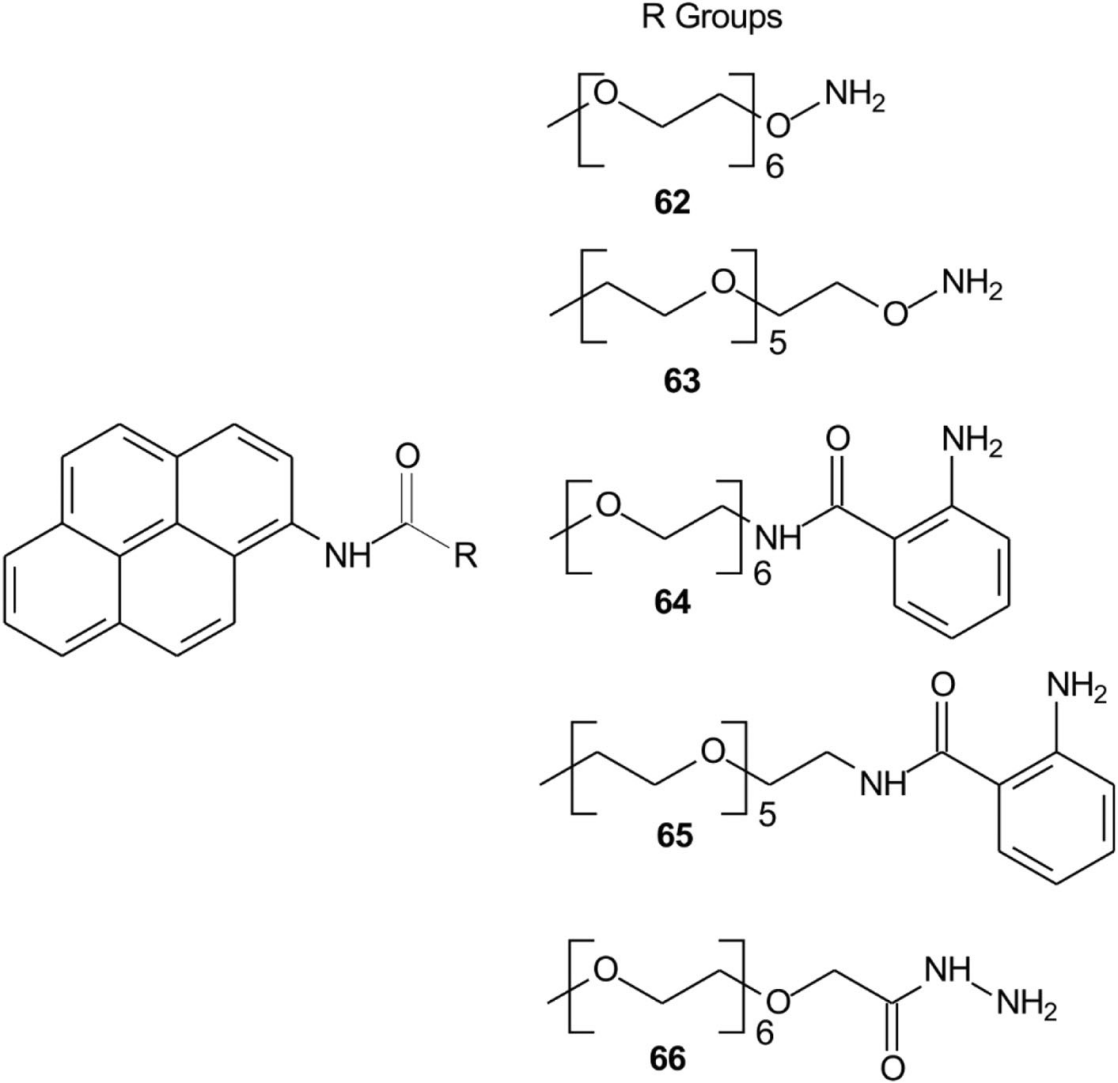
Pyrene derivatives for LALDI‐MS.

The absence of a matrix overcomes the problem of matrix ions masking ions produced by low molecular weight glycans. Hauser et al. (2021) have developed a technique, which they refer to as “label‐assisted laser desorption/ionization mass spectrometry” (LALDI‐MS) that dispenses with the traditional matrix. Sugars were tagged at the reducing terminal with pyrene‐based reagents (**62** – **66**, Scheme 3), which behaves in a similar way to the matrix by absorbing the laser energy. The labels were designed to avoid the laser‐induced loss of ketene inherent in earlier pyrene tags (Yoneda et al., 2016). In this way, only the labelled compounds in a mixture were detected. The method was demonstrated by detecting lactose (**30**) and extending it to its detection directly in cow's milk.

Electrochemical deposition of silver from silver trifluoroacetate at 10 V for 15 min has produced a surface that showed intense surface‐assisted laser desorption/ionization (SALDI)‐MS signals for standards from various classes of compounds including sugars, lipids, fatty acids and cyclitols at a concentration of 1 nmol/spot, with values of the signal‐to‐noise ratio greater than 50. The values of the limit of detection were 0.71 μM for adonitol (**67**), 2.08 μM for glucose and 0.39 μM for palmitic acid per spot (Arendowski et al., 2022). Using a through‐hole alumina membrane as an ionization‐assisting substrate, Fukuoka et al. (2021) have successfully analysed a series of mannosylerythritol biosurfactants (**68**) with molecular weights below about 750 Da.


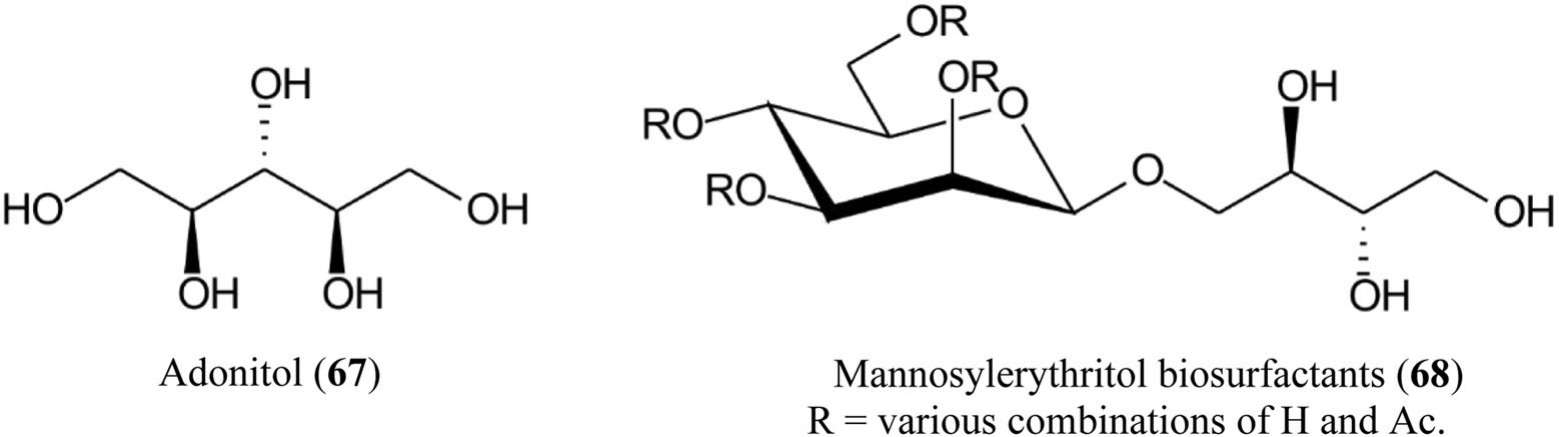


New matrices relevant to MALDI imaging are covered in Section 7.2.1.

## 7 MALDI IMAGING

MALDI imaging is possibly the fastest growing area in the use of MALDI ionization. New methods are constantly being developed with greater sensitivity and resolution. Many applications now involve enzymatic digestion of samples by spraying enzymes onto the material to be imaged and there are many new matrices being developed for specific purposes. Several reviews have been published over the 2‐year period covered by this review: These are summarized in Table 3.

**Table 3:** Reviews and general articles on matrix‐assisted laser desorption/ionization imaging.

Subject	Contents	Citations	References
Imaging of the human brain	Imaging in different disease states (cancer, Alzheimer's, epilepsy, etc.)	140	Ajith et al. (2021)
Mass spectrometry imaging for spatial chemical profiling of vegetative parts of plants	General review. Different types of imaging (MALDI, DESI, SIMS, LAESI). Applications–disease, etc.	163	Ajith et al. (2022)
MALDI Mass spectrometry imaging and glycomics	Discussion of glycan types: *N*‐glycans, GSLs, GAGs, glycosides, free glycans	117	Blaschke and Drake (2022)
Sample preparation of biological tissues for MALDI‐MSI	Embedding, storage, sectioning, FFPE samples, washing (lipids, glycans proteins) enzyme digestion (proteins, glycans), derivatization, matrix selection	101	Cillero‐Pastor and Cuypers (2022)
Exploring natural products through mass spectrometry imaging	Concentrates on recent progress with plants and microorganisms	133	Dong and Aharoni (2022)
Imaging mass spectrometry	General review with sections on different compound types, including *N*‐glycans	171	Drake et al. (2021)
Applications of stable isotopes in MALDI imaging	Application to measurements of UDP‐glucose and glucose phosphates in bovine lens	111	Grey et al. (2021)
On‐tissue chemical derivatization in mass spectrometry imaging	Covers ionization techniques. On‐tissue derivatization of various functional groups, reagent deposition, applications to glycomics, lipidomics and proteomics. Table of reagents	151	Harkin et al. (2022)
Mass spectrometry imaging of the brain glycome	Contains tables listing deglycosylation methods and MALDI matrices used for brain studies	190	Hasan et al. (2021)
Mass spectrometry imaging for direct visualization of components in plant tissues	General review, ionization methods, matrices, applications to compound type	115	Hu, Han, et al. (2021)
Recent advances in surface‑assisted laser desorption/ionization mass spectrometry and its imaging for small molecules	Discussion of different types of substrate and applications	132	Huang, Ouyang, et al. (2022)
Advanced applications of mass spectrometry imaging technology in quality control and safety assessments of traditional Chinese medicines	Covers topics such as sample preparation, matrix selection and various applications	123	Jiang, Zhang, et al. (2022)
An introduction to MALDI ionization mechanisms for users of mass spectrometry imaging	Covers laser ablation, plume pressure, temperature and velocity, laser spot size, ionization “lucky survivors”, thermal and non‐thermal ionization, metal surfaces, secondary ionization, matrix and analyte suppression	76	Knochenmuss (2021)
Molecular tissue profiling by MALDI imaging: Recent progress and applications in cancer research	Methods (instrumentation, matrices, matrix deposition, quantification), applications (identification of disease, biomarkers, drug distribution)	142	Lee, Yeoh, et al. (2021)
Mass spectrometry imaging of small molecules. Methods and protocols	Book	‐	Lee (2022)
Matrix‐assisted laser desorption/ionization mass spectrometry imaging for in situ analysis of endogenous small molecules in biological samples	General review, matrices with extensive table of matrices for various compounds, matrix coating methods, instrumentation, applications	192	Liu, Pan, et al. (2022)
Surface‐assisted laser desorption/ionization mass spectrometry imaging: A review	Definition of SALDI. Mechanisms. Strategies for SALDI imaging. Applications	274	Müller et al. (2022)
MALDI Mass spectrometry imaging in lipidomics (and glycolipidomics)	Sample preparation, MALDI matrices and application, identification of lipids by accurate mass measurements, MS/MS, ion mobility. Applications (cancer research, brain injury, liver disease), MALDI‐2, single cell analysis, use of stable isotopes	171	Mutuku and Ellis (2022)
Cell and tissue imaging by TOF‐SIMS and MALDI‐TOF: An overview for biological and pharmaceutical analysis	General review, methods, applications to cancer, toxicology, drug detection, combination with other methods	262	Noun et al. (2022)
Mass spectrometry‐based lipid analysis and imaging	General article on methods	189	Pathmasiri et al. (2021)
MALDI mass spectrometry imaging: The metabolomics visualization	Brief general review with applications to glycolipids,	48	Salviati et al. (2022)
Unravelling the local complexity of biological environments by MALDI mass spectrometry imaging	Reviews MALDI imaging for a wide range of compounds	114	Sgobba et al. (2021)
Introduction to spatial mapping of biomolecules by imaging mass spectrometry	Book, General coverage with chapters on methods and different compound types	‐	Shrestha (2021a)
Imaging mass spectrometry: Glycans	Brief general coverage	29	Shrestha (2021c)
Imaging mass spectrometry: Gangliosides in brain tissue	Book chapter, brief coverage	28	Shrestha (2021b)
Instrumentation for MALDI‐MSI – Part I. Ionization sources and design	Vacuum, intermediate and atmospheric pressure sources, special resolution, modes of illumination, postionization, MALDI‐2, MALDESI	70	Soltwisch (2022)
Quantitative mass spectrometry imaging of biological systems	Topics such as matrix effects on imaging, quant. of small molecules in tissues, addn. of standards, proteins	96	Unsihuay et al. (2021)
Research progress of derivatization methods in MALDI mass spectrometry imaging	Derivatives for various functional groups. Linkage‐specific sialic acid derivatization. In Chinese	95	Wang, Zhang, and Guo (2021)
Recent developments of novel matrices and on‐tissue chemical derivatization reagents for MALDI‐MSI	General review covering different compound types	94	Zhou et al. (2021)
Advances in MALDI mass spectrometry imaging single cell and tissues	General review on methods. Small section on *N*‐glycoproteomes	214	Zhu, Xu, et al. (2022)

### 7.1 Methods

Current matrix deposition methods face technical problems related to the inhomogeneous distribution of crystals and the low analyte extraction and cocrystallization efficiency prompting several investigators to develop techniques that are more efficient. In the approach adopted by Li, Wu, et al. (2022), an integrated matrix sublimation device with synchronous solvent nebulization has been developed. In operation, droplets of solvents were directly introduced into the chamber of the sublimator by using a miniaturized ultrasonic nebulizer unit and, at the same time, the matrix (DHB) was sublimed. Both synchronous and asynchronous modes of solvent nebulization and matrix sublimation were systematically investigated, but the synchronous technique was found to give the best results. Imaging of both protein (from 2,5‐dihydroxyacetophenone [DHA, **69**]) and small metabolites (e.g., sulfatide [**48**] in negative mode) was achieved in mouse brain tissue sections with clearly improved performance compared with those of conventional spray and sublimation methods. Luo, Song, Mao, et al. (2022) have overcome some of the problems related to matrix deposition by developing an automated heated sprayer system which they claim produces a more even matrix deposition and increases sensitivity by twofold to fivefold. To reduce analyte movement within the sample caused by matrix application, Nambiar et al. (2021) have developed a freeze‐spot method for matrix (DHB) application whereupon the matrix solution freezes on contact with the sample and the solvent dissipates by sublimation. The method was found to be particularly useful for small sample sections.


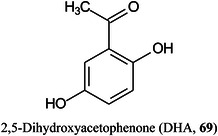


Three methods for matrix application have been evaluated by Deng, He, et al. (2021) for imaging of potato glycoalkaloids such as α‐solanine (**70**). Each method has advantages and disadvantages. Sublimation reduces analyte diffusion because there is no solvent sprayed directly onto the tissue. The main advantages are the small matrix crystals and the homogenous matrix layer that is formed. However, the sensitivity of the method is usually lower than matrix application by spraying. Airbrush spraying is relatively fast and simple but tends to generate matrix crystals that are too large for high spatial resolution imaging. The third method was a “two‐step matrix application” technique (Shimma et al., 2013), which combined matrix sublimation and airbrushing. By comparing these methods, ionization efficiencies were ranked according to the average ion signal intensity of four glycoalkaloids as follows: sublimation* < *airbrushing* < *sublimation & airbrushing resulting in the combination of sublimation and airbrushing being chosen as the matrix deposition method of choice.


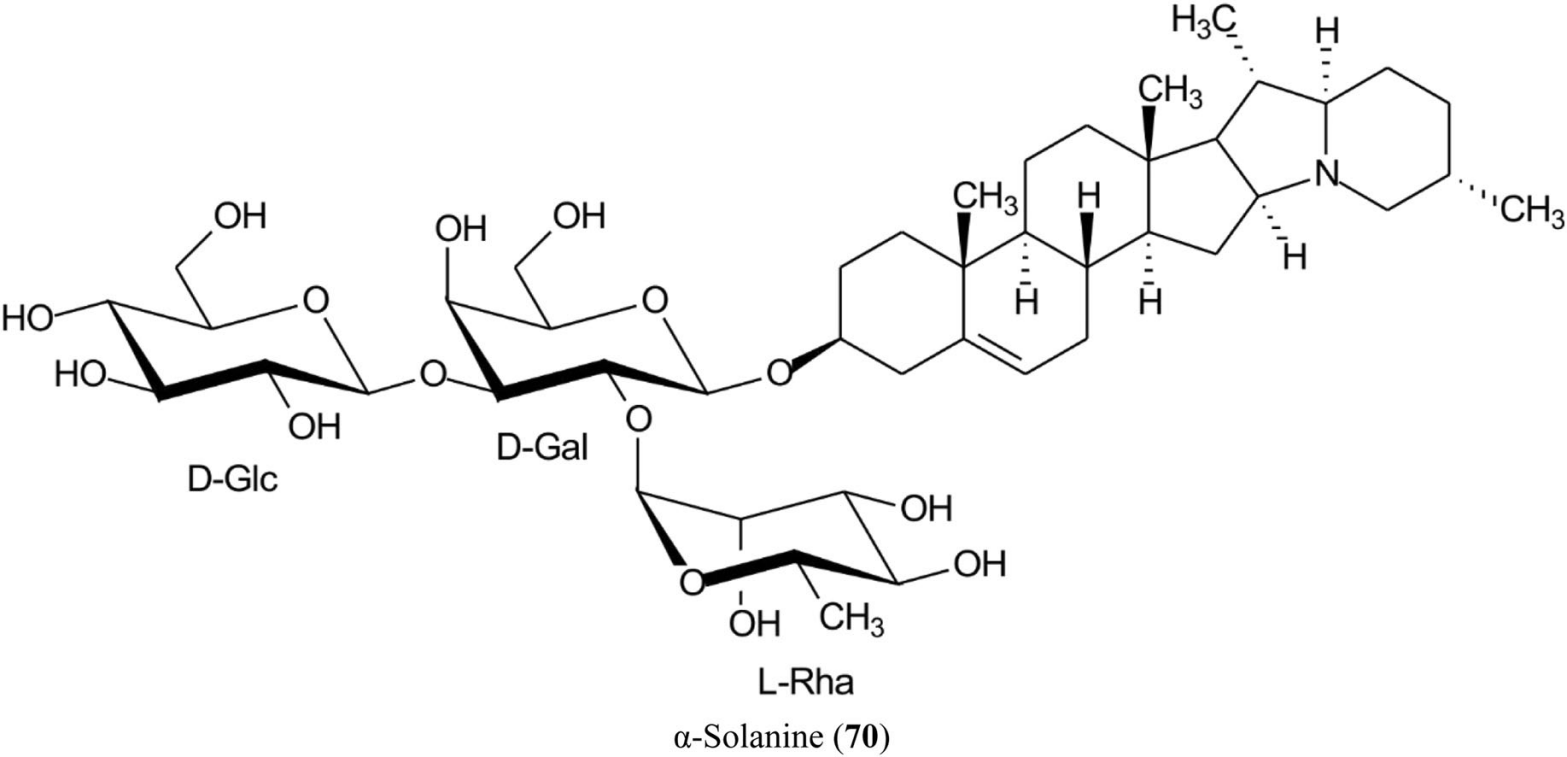


The size and distribution of matrix crystals deposited on the surface of a tissue section are critical for satisfactory imaging. Xie, Wu, et al. (2021) have achieved uniform distribution and a restricted size of matrix crystals by use of a homemade matrix sublimation device with a subzero controllable crystallization temperature, giving homogeneous matrix crystals with diameters <0.2 μm. The method was applied to endogenous and exogenous components in the tissues of strawberries, kidneys and mussels. Good reproducibility was achieved, and the quality of the ion images was significantly improved compared with the use of more traditional methods. Compounds such as pelargonidin‐3,5‐diglucoside (**71**), a previously undetected compound, were found in strawberries at −15^o^C illustrating the power of the technique.


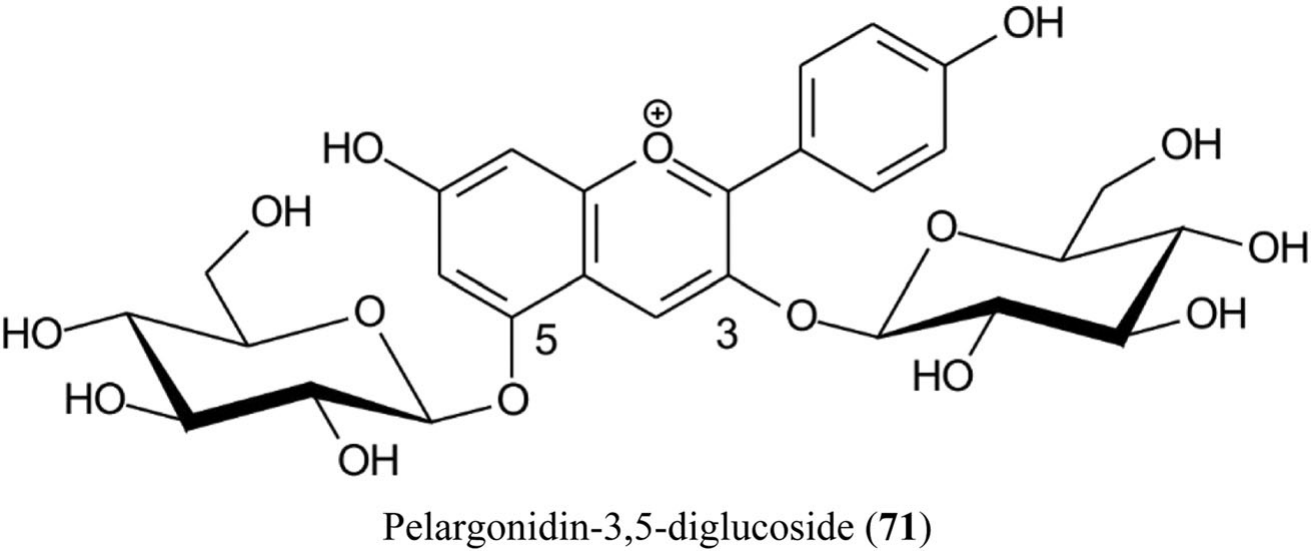


Rather than spraying the matrix on top of the sample, Xu, Deng, Ye, et al. (2021) have used prepared slides containing a single layer of the matrix graphene oxide film on indium‐tin oxide (ITO) slides, onto which the sample was placed, Using rat brain slices they imaged 60 kinds of lipids including HexCer (**46**), phospholipids, cyclic adenosine monophosphate, inosine, and cholesterol. The slides could be stored for over a month and their use avoided problems such as sprayer nozzles becoming blocked.

Lipids, including HexCer, have been imaged in mouse brain in both positive and negative modes using a dual polarity approach (Müller, Verdin, et al., 2021) on alternate pixels. Gold nanoparticles were used as the matrix in both polarities with an FT‐ICR instrument. Images from six accumulated laser shots were acquired from each pixel at a repetition rate of 60 Hz with Kendrick mass defect filtering used to aid lipid identification. Approximately 200 lipid species were identified. Blanc et al. (2021) have emphasised the advantages of using administered substrates incorporating the stable isotopes ^12^C and ^13^C for deconvolution of metabolic pathways and have also used the Kendrick mass defect method to analyse the data. Applications included a study of cancer metastases in mouse brain.

With the latest instrumental developments, where pixel sizes in the micrometre range can be obtained, investigations are becoming increasingly focused on single cell analysis. Traditional methods of matrix application at this scale can be problematical because of imperfections or inhomogeneities in the matrix layer. A solution is to use premanufactured, homogeneous ionization‐assisting devices such as a matrix‐free imaging technique called Desorption Ionization Using Through‐Hole Alumina Membrane (DIUTHAME) in which a premanufactured nanostructured membrane is deposited on top of a tissue section rather than by use of a spray coating of an organic matrix. By use of this method, Müller, Bhandari, et al. (2021) acquired spectra at atmospheric pressure and, compared to MALDI MSI, DIUTHAME MS images displayed higher signal homogeneities, higher contrast and reduced background signals, while signal intensities were reduced by about one order of magnitude. DIUTHAME membranes used on tissue sections thicker than 50 μm, were successful for mammal, insect and plant tissue with a high lateral resolution down to 5 μm.

Problems exist in the application of MALDI imaging to adipose tissues arising from poor matrix distribution and crystallization caused by excess liquid lipids on the tissue surface. The problem particularly affects lipid‐rich white adipose tissue. Wang, Sun, Kunzke, et al. (2022) have developed a simple and low‐cost preparation step which they refer to as “filter paper application” It consists of placing a filter paper onto the tissue before matrix application to remove the layer of excess liquid lipids. Thirty seconds was found to be optimal and the method resulted in a higher number of detected *m/z* species, including nucleotides, carbohydrates, and amino acids, and higher ion intensities than before the filter paper application.

McEwen et al. (2022) have developed a new liquid tissue sampling method which they call “poly‐synchronous surface extraction” (PSSE) that uses an omniphobic substrate patterned with hydrophilic surface energy traps (SETs) which, when wet with a solvent, form a dense microdroplet array. When in contact with a tissue sample, each microdroplet extracts analytes from the tissue surface, which can be analyzed by MALDI‐IMS. The method was used to examine glycosides, such as pelargonidin‐3‐*O*‐glucoside (see **71**), in slices of a strawberry (*Fragaria × ananassa*) and the method was shown to produce similar results to direct analysis and demonstrated the potential of the method to increase the speed of ambient MS tissue imaging techniques by decreasing the number of steps required for sample preparation.

As a method for increasing confidence of compound analysis, Rensner and Lee (2022) have used hydrogen/deuterium exchange (HDX) to provide information on the number of exchangeable hydrogen atoms for up to 17 labile hydrogens. HDX efficiency of 73%−85% were achieved by introducing D_2_O vapour into a heated MALDI source in combination with a deuterium labelled matrix (DHA). The D_2_O vapour was introduced directly into the ion funnel of an Orbitrap mass spectrometer by bubbling a stream of nitrogen through D_2_O. Complications arose because of the presence of ^13^C isotope peaks which needed a resolution of 280,000 for separation; higher than that of the Orbitrap. This problem was overcome by subtracting the contribution of the ^13^C isotope calculated from the number of carbon atoms in the compound's molecular formula. Applications were to the study of metabolites in sections of the fronds from *Lemna minor* (duckweed).

Dreisbach et al. (2021) have interfaced an autofocusing atmospheric pressure AP‐SMALDI AF high‐resolution MALDI imaging ion source to a Q Exactive HF Orbitrap mass spectrometer to obtain 3D images of cardiac glycosides produced by wounded leaves from the plant *Asclepias curassavica*. The ion source incorporated a diode‐pumped solid‐state laser operating at 343‐nm wavelength and at 100 Hz, irradiating the sample at 35° relative to the transfer capillary axis of the mass spectrometer. This system enabled the authors to keep the desorption/ionisation laser focus, fluence and ablation spot size constant across sample height differences by adjusting the sample stage position according to the sample height profile for each measurement spot. The instrument was operated at a resolution of 240,000 (at *m/z* 200) over a mass range of *m/z* 250 to 1000. The results showed an increased latex flow rate towards the point of leaf damage leading to an accumulation of defence substances in the affected area.

#### 7.1.1 Sample preparation

A report on optimization of sample preparation protocols for MALDI imaging of single cells has concentrated on washing, drying, chemical fixation, and matrix coating steps (Bien et al., 2021). Incubation of cells with formalin for about 5 min after isotonic washing and drying, resulted in a robust protocol that largely preserved not only cell morphologies, but also the molecular integrities of amine group‐containing cell membrane phospholipids. The method was demonstrated with four model cell lines, cultured directly on ITO‐coated glass slides. Transmission (t‐)mode MALDI‐2 gave a pixel size of 2 μm.

### 7.2 Matrices

Angerer et al. (2022), using an atmospheric pressure (AP‐) MALDI ion source coupled to an Orbitrap Elite mass spectrometer have evaluated six MALDI matrices and several protocols for analysis of lipids and glycolipids in mouse brain sections. Of the matrices CHCA, norharmane, DHB, 2,6‐dihydroxyacetophenone DHAP (**72**), 2,4,6‐trihydroxyacetophenone (THAP, (**73**), and DAN (**36**), the largest number of lipids were detected with CHCA and THAP, while THAP and DAN provided the best signal intensities. In negative‐ion mode, DAN showed the best lipid coverage and DHAP gave the best results for gangliosides. One hundred fifty‐five lipids were detected in positive ion mode with THAP and 137 in negative‐ion mode with DAN. The spatial resolution achievable with DAN was 10 μm and the overall results show that the performance of AP‐MALDI is comparable to that of vacuum MALDI.


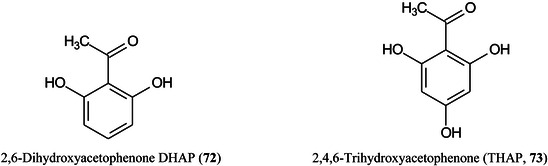


Treu and Römpp (2021) have advocated the use of cluster ions from common matrices as calibration standards for imaging experiments. DHB, for example, can form clusters with added NH_4_^+^ or added alkali metal ions of the type [aM + X^+^‐bH_2_O]^+^ (where X = added ion) with masses up to *m/z* 1378.19427. In negative ion mode, ions of the type [aM –bH +  (b‐1)X–cH_2_O]^‐^ or [aM–bH–H + (b‐1)X]^‐^ can be formed. CHCA, sinapinic acid, *trans*‐2‐[4‐*tert*‐butylphenyl‐2‐methylprop‐2‐enylidene]‐malonitrile (DCTB, **74**), 4‐nitroaniline (pNA, **75**), 1,5‐DAN and norharmane all formed both positive and negative ion clusters but THAP and 9‐AA worked best in negative ion mode.


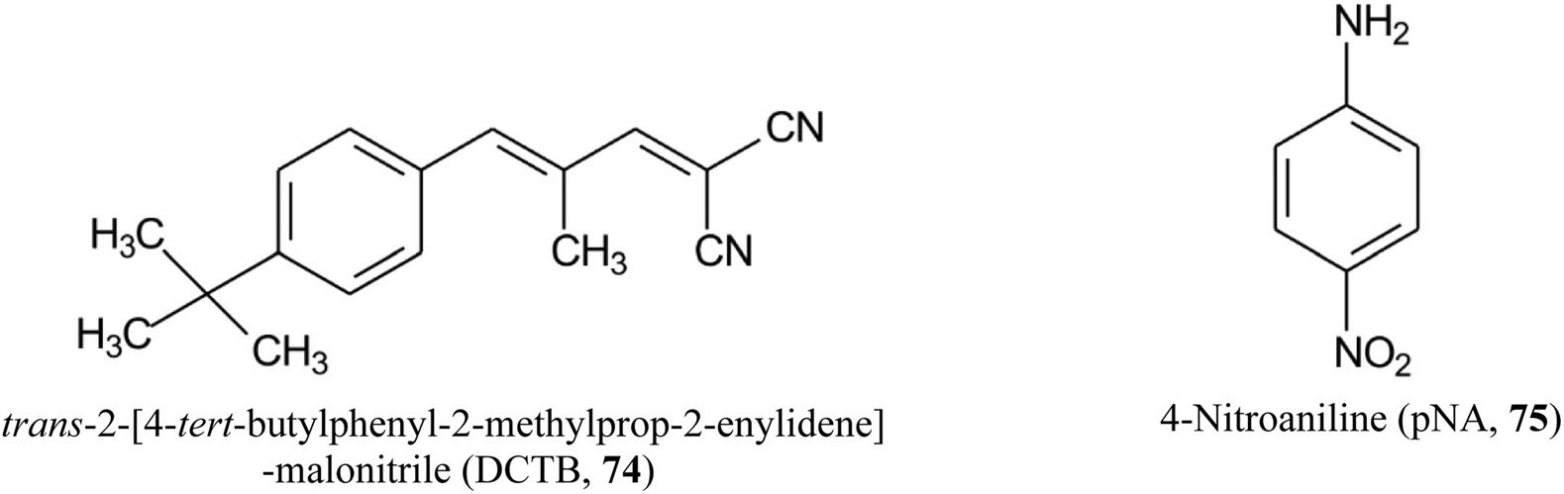


#### 7.2.1 New matrices

Several new matrices for MALDI imaging have been introduced during the review period.

##### 7.2.1.1 Organic matrices

Hydralazine (**76**) has been found to be a versatile and universal matrix for MALDI imaging of a wide range of endogenous compounds between 50.0 and 20,000.0 Da including glucosylceramides, galactosylceramides, sulfatides and gangliosides in both positive and negative ion modes. To improve its performance the matrix was doped with NH_4_OH or trifluoroacetic acid (TFA), resulting in superior performance for imaging biologically relevant compounds in the negative and positive‐ion modes, respectively. Compared with conventional matrices such as DHB, CHCA, and 9‐AA), hydralazine provided higher sensitivity, broader molecular coverage, and improved signal intensities and was applied successfully for the visualization of tissue‐specific distributions and changes of small molecules, lipids, and proteins in murine kidney and liver sections (Tang, Gordon, et al., 2021).


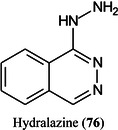


Gold nanoparticles (AuNPs) modified TiO_2_ nanospheres modified with gallic acid (**77**) to give TiO_2_@GA nanospheres have been used as a surface‐assisted (SALDI) substrate for imaging, They were sprayed onto ITO glass slides using a gas‐assisted electric sprayer and compared with matrices such as DHB, 2‐mercaptobenzothiazole (MBT, **78**), DAN, DHA, and 9‐AA. The nanospheres provide higher detection sensitivity, lower background interference, dual‐polarity detection and enhanced ionization efficiency of various endogenous molecules. Animal tissues (mouse brain, kidney, and liver) yielded mainly neutral lipids but plant tissues such as potato tubers additionally enabled glycoalkaloids to be mapped (Sun, Tang, et al., 2022).


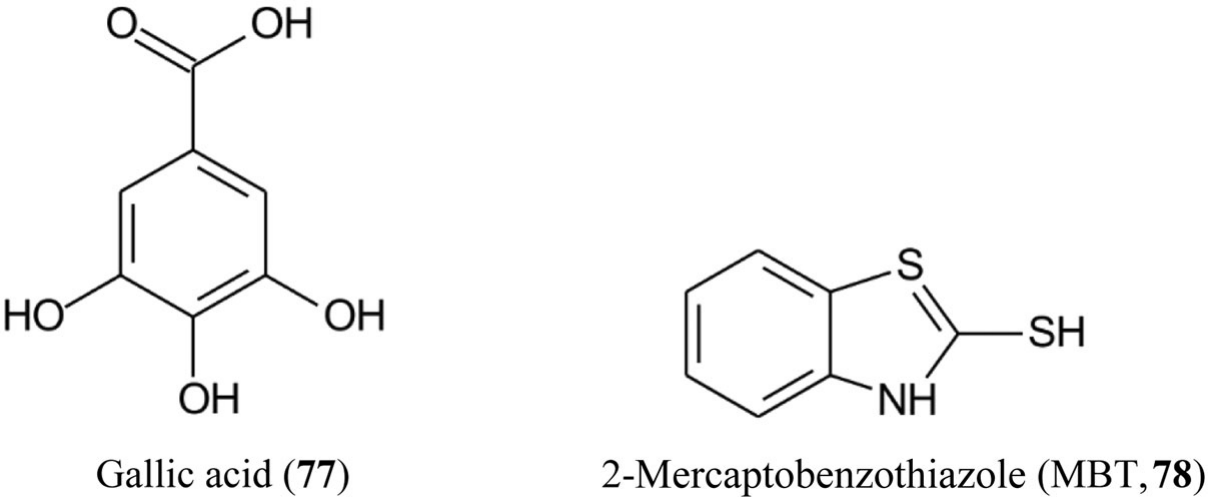


Another new matrix for small molecules consists of yolk‐shell Ni/NiO nanoparticles anchored onto nitrogen‐doped graphene (Ni/NiO/N‐Gr) and capable of analysing molecules in both ion modes (Zhao, Li, et al., 2022). The matrix showed the superior behaviour for the analysis of various small molecular metabolites such as carbohydrates amino acids, spermidine (**79**), creatinine (**80**), hippuric acid (**81**), dopamine (**82**), and ascorbic acid (**83**) with high sensitivity and excellent salt tolerance compared to the traditional CHCA and control substances (Ni/N‐Gr and NiO/N‐Gr). The matrix gave accurate quantitation of blood glucose in mice with a linearity concentration range of 0.2–7.5 mM and qualitative detection of various endogenous small molecular metabolites in murine serum and urine. Excellent spatial distribution of lipids in imaging the hippocampus region of mice brain was obtained.


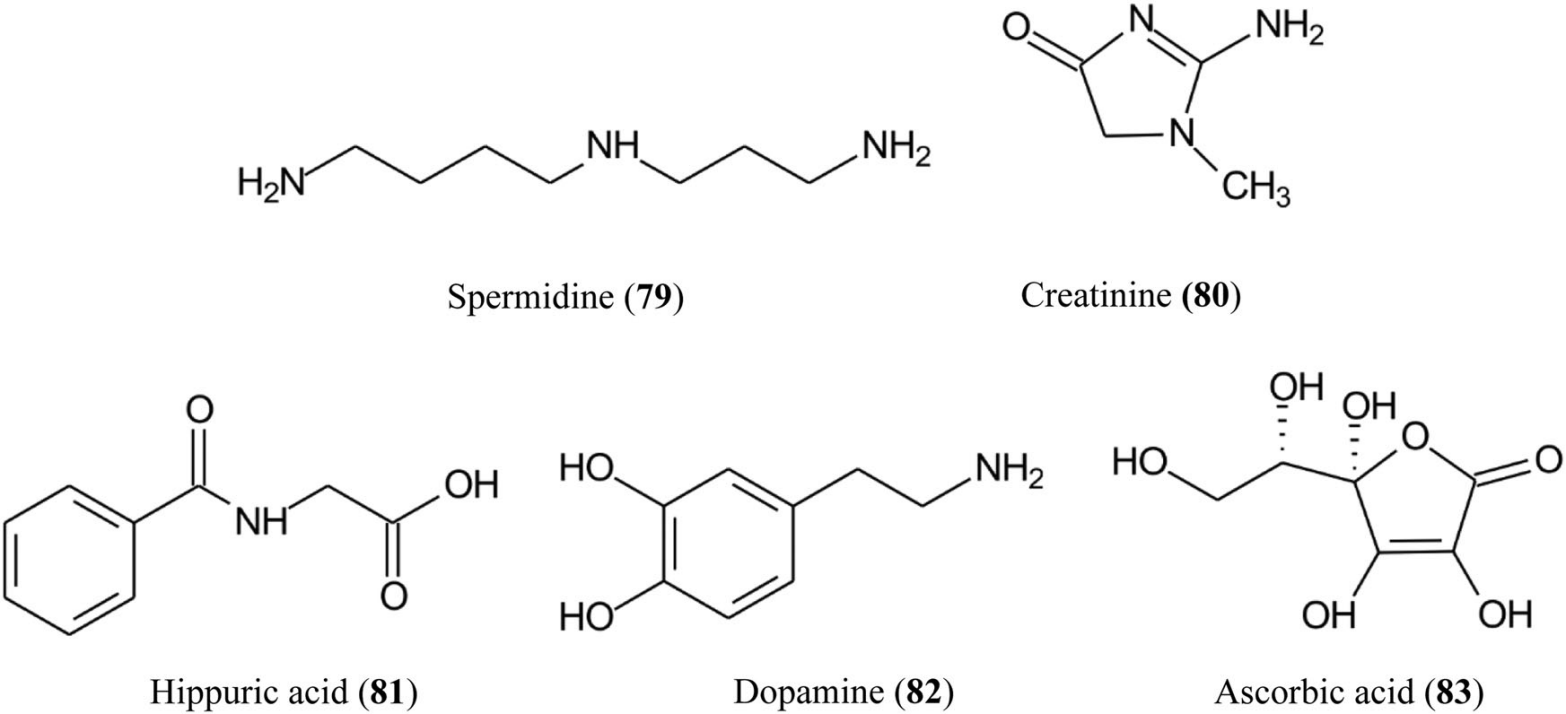


4‐Aminocinnoline‐3‐carboxamide (4‐AC, **84**) has been developed as a new dual‐polarity matrix and compared with traditionally matrices such as DHB and 9‐AA. It was reported to exhibit superior performance in UV absorption at 355 nm, better ion yields, low background interference and vacuum stability than the more traditional matrices. It was used to map many types of compound in mouse brain in a transgenic mouse model of Alzheimer's disease. Ninety‐three metabolites were shown to exhibit different levels of regional changes compared to the age‐matched controls (Chen, Hu, et al., 2022).


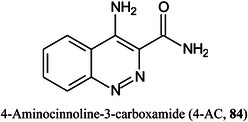


Several glycosylated matrices have been synthesised by combining glucose with common MALDI matrices such as 3‐AQ, 6‐AQ, and DAN. Compared with their parent matrices, the glycosylated matrices exhibited remarkably improved sensitivity and higher signal reproducibility in detecting small metabolites. Glucosylated 6‐AQ (6‐GAQ, **85**) exhibited the best performance with a detection limit for citric acid in the low fmol range. The matrix was used to image metabolites from mouse kidney sections, and showed higher sensitivity and lower background noise than the commonly used matrices. More importantly, this matrix could selectively detect hydrophilic metabolites, especially the hydrophilic lipids in the mouse kidney (Ma, Zhao, et al., 2022).


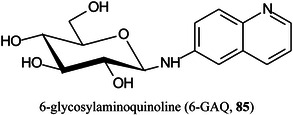


##### 7.2.1.2 Nanoparticles and quantum dots

To overcome problems such as low‐mass matrix peaks, various inorganic nanomaterials, such as gold nanoparticles, and metal oxides such as TiO_2_ have proved to be successful. TiO_2_, in particular has been preferred because of its favourable UV absorbing property, high chemical stability, and facile surface modification properties. Sun, Zhang, Tang, et al. (2022) have utilized this latter property to combine TiO_2_ submicron particles with various DHB isomers and have found that 3,4‐DHB (**86**)–TiO_2_ provides superior performance than the conventional matrices such as DHB or CHCA. The matrix exhibited low background noise and high detection sensitivity for the visualization of spatial distribution patterns of secondary metabolites such as flavonoids in the roots of the differently aged medicinal herb *Scutellaria baicalensis* Georgi (Chinese skullcap).


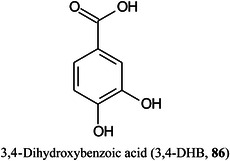


Nitrogen‐doped quantum dots, with their electron‐rich sites, promoted deprotonation and formation of negative ion spectra from compounds such as amino acids, carbohydrates and fatty acids. The matrices were highly salt‐tolerant and produced reproducible spectra as demonstrated by imaging of low molecular mass species in rat brain tissue (Jin, Liu, et al., 2022). Plasmonic Gold Nanoshell (SiO2@Au) is another type of new matrix that is stated to outperform that of conventional matrices and to be appropriate for a wide range of molecules such as carbohydrates, amino acids, peptides, drugs, nucleosides and dyestuffs (Du, Chen, et al., 2022). It has been used to image strawberry tissues at a pixel size of 100 μm without the presence of imaging artefacts and for mapping the lipid distribution within the whole‐body tissues of zebrafish (*Danio rerio*), honeybees (*Apis cerana*), and mouse brain tissues in a spatially resolved manner at pixel sizes of 55, 30, and 50 μm, respectively.

### 7.3 Surface‐assisted laser desorption/ionization mass spectrometry (SALDI‐MS)

SALDI‐MS, Because of its low background, has been successfully applied in the analysis of various small molecules. A new substrate, gold nanoparticles/thiol‐β‐cyclodextrin‐functionalized TiO_2_ nanowires (AuNPs/SH‐β‐CD‐TiO_2_ NWs) have been prepared on ITO glass slides and their performance compared with that of conventional organic matrices such as DHB (Wang & Li, 2022). The new substrate showed superior performances on detection sensitivity, repeatability and analyte coverage of various small molecules, such as carbohydrates, fatty acids, and bile acids in negative‐and positive ion mode and was used to profile several natural products in spearmint leaves and potato tubers. Magnetron‐sputtered niobium nanoparticles (a monoisotopic metal) has also been used as an alternative to expensive noble metals and found to work particularly well with GalCer and phospholipids (Pleskunov et al., 2022).

### 7.4 Nanopost arrays

MALDI using traditional matrices is relatively ineffective at ionizing neutral lipids and glycolipids, particularly in the presence of phospholipids. A recent innovation that improves the situation is to use silicon nanopost arrays (NAPA). Fincher et al. (2021) have produced NAPA wafers from low‐resistivity p‐type silicon wafers using UV projection photolithography followed by deep reactive ion etching to give arrays with a final dimensions of 1100 nm in height, 150 nm in diameter, and with a periodicity of 350 nm. Use of these arrays was then combined with trapped ion mobility imaging mass spectrometry (TIMS IMS) for examination of intact rat brain and kidney tissue which were placed directly on the arrays. The method provided enhanced ionization efficiency for neutral lipid species and provided complementary coverage to MALDI imaging. It enabled imaging of neutral lipid species at 20 μm spatial resolution and increased molecular coverage greater than twofold as the result of separation of molecular species, such as triglycerides, cholesteryl esters, HexCers and phospholipids, into distinct mobility‐*m/z* bands using gas‐phase ion mobility separations. In addition, the method allowed for the separation of isomeric species, including mobility resolved isomers of Cer(d42:2) (*m/z* 686.585).

Dufresne et al. (2021) have developed a precoated substrate that enables high spatial resolution of phospholipids, neutral lipids and glycolipids in positive ion mode as metal cation adducts. The substrates were constructed by depositing a layer of CHCA and potassium salts onto silicon nanopost arrays before tissue mounting. The matrix/salt precoated NAPA substrate was shown to significantly enhance all detected lipid signals allowing lipids to be detected at lower laser energies than could be obtained with bare NAPA. The method enabled ion images to be generated at 10 μm spatial resolution from samples such as rat retinal tissue. Signal intensity increases of at least 5.8 ± 0.1‐fold for phospholipids and 2.0 ± 0.1‐fold for neutral lipids compared to bare NAPA were obtained.

TiO_2_ Nanopillar arrays have been developed by Yamada et al. (2021) and shown to be effective at ionizing small amino acids, sugars, pesticides, peptides, and proteins with molecular weights of up to 24,000. A substrate with a lower surface density exhibited more intense signals for the detection of small (∼1.2 kDa) analytes as the result of more effective heat confinement. Wetting behaviour was another factor facilitating better performance for smaller molecules at lower surface density. On the other hand, the homogeneous adsorption of target molecules onto the nanopillared surface was thought to be a dominant factor for the detection of the larger proteins.

### 7.5 MALDI‐2 and related methods

With pixel sizes in the 5–20 μm range, the number of ions produced by each pixel is small resulting in low sensitivity. Detection of the sample molecules can also be compromised by ion suppression effects caused by ready ionization of major and easily ionisable constituents such as phosphatidylcholine (**87**). One way to alleviate the problem is to use a second laser to produce post‐ionization, a technique known as MALDI‐2, capable of boosting sensitivity by 2–3 orders of magnitude and well described in the protocol published by Dreisewerd et al. (2022). The authors state that two prerequisites are required for exploiting the MALDI‐2 effect, namely the use of ion sources that are operated under elevated pressure (a few mbar of nitrogen buffer gas) and the use of a pulsed UV post‐ionization laser. The wavelength of this laser should fall below the two‐photon threshold of the utilized MALDI matrix which, for aromatic matrices, such as, for example, DHB, CHCA or DHAP, is around 310 nm. Frequency‐quadrupled Q‐switched Nd:YAG lasers, which emit at 266 nm are suitable. Experiments with two pulse widths (28 ps and 6 ns at a wavelength of 266 nm) for the ionizing laser support a resonance enhanced two‐photon ionization (REMPI) of neutral matrix molecules desorbed by the MALDI laser from DHB or DHA (Potthoff et al., 2022). In a modification, known as transmission (t‐) mode MALDI, the target is illuminated from the back. Pixel sizes in the 1–2 μm range have been achieved and the method has been used, in combination with an optical microscope to image lipids and glycolipids in single cells and intercellular matrices at a pixel size of 2 μm (Bien et al., 2022).


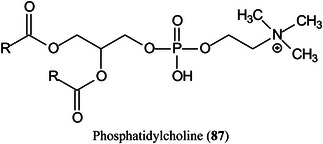


Postionization has also been implemented in an IR‐atmospheric ion source with improvements designed to overcome some of the disadvantages associated with AP sources (Schneemann et al., 2022). Ambient MS imaging comes with the advantage that visualizing biomolecules from tissues involves no or minimal sample preparation but it suffers from a pronounced bias towards either polar or nonpolar analytes. The improvements to the source devised by the authors involves use of an in‐capillary dielectric barrier discharge (DBD) module for postionization of neutrals desorbed by the IR‐MALDI) MSI source. The device was found to enhance the signal intensities of nonpolar compounds by up to 10^4^ compared to IR‐MALDI, without affecting transmission of IR‐MALDI ions. It was used to study mouse tissue and *Danaus plexippus* caterpillar tissue sections, visualizing the distribution of glycolipids, sterols, fatty acids, monoglycerides, and diglycerides that are not detected in IR‐MALDI MSI experiments and allowed mapping of nonpolar analytes with pixel resolutions down to 20 μm.

A related postionization technique involves irradiating the MALDI target with a series of nsec‐long UV laser pulses of 349 nm wavelength on a pixel‐by‐pixel basis, analogous to a classical MALDI‐MSI experiment. To induce secondary ionization in the MALDI plume, this material was irradiated by three RF‐Krypton discharge lamps operated at 13.560 MHz. Pulses of light were synchronized with the MALDI laser and the ion source was operated at about 10 mbar of N_2_ and dopant vapour (e.g., acetone) was introduced *via* a capillary system. Under these conditions, samples reacted with the dopant gas, and residual water vapour to give chemical ionization (CI)‐type reactions that were dissimilar to those seen in MALDI‐2. The technique was used to image lipids and glycolipids from animal tissues and it was reported that signal intensities could be boosted by up to 2–3 orders of magnitude (Bookmeyer et al., 2022).

Another technique involves plasma‐based postionization after the MALDI ion source using a commercially available “soft ionization by chemical reaction in transfer” (SICRIT) system interfaced to a trapped ion mobility mass spectrometer (Michael et al., 2022). The instrument worked particularly well for lipids, including glycosphingolipids and the ion mobility function was invaluable for separating isomers that were not resolved in the *m/z* dimension.

### 7.6 Formalin‐fixed and paraffin‐embedded (FFPE) samples

Applications in this area are particularly relevant to clinical studies. Sections of retina analyzed by imaging‐MS are typically fresh‐frozen. However, paraformaldehyde fixation facilitates the preservation of tissue morphology by forming methylene bridge crosslinks between formaldehyde and amine/thiols in biomolecules and would possibly be a better method for sample preparation for imaging. Consequently, Kotnala et al. (2021) have compared the molecular identity of lipids and glycolipids generated by MALDI‐IMS and LC–MS/MS for fixed and fresh‐frozen retina tissues in both positive and negative ion modes. More lipid signals were observed in fixed compared with fresh‐frozen retina. More potassium adducts were observed in fresh‐frozen tissues than in fixed tissues because the fixation process caused displacement of potassium adducts to protonated and sodiated species in ion positive ion mode. LC–MS/MS analysis showed an overall decrease in lipid signals due to fixation, particularly with glycerophospholipids and glycerolipids. However, the method largely conserved the signals from most sphingolipids and cholesteryl esters.

#### 7.6.1 Use of enzymes

For studies of compounds such as large polymers and *N*‐linked glycans from glycoproteins, methods, usually enzymatic, are needed to render them suitable for mass spectrometric analysis. Several investigators have used such methods for analysis of various compounds in FFPE tissues.

##### 7.6.1.1 Release of *N*‐linked glycans from glycoproteins with peptide‐N‐glycosidase F (PNGase F)

Unstandardized and uncontrolled incubation steps in the *N*‐glycan release step often cause significant delocalization of released *N*‐glycans, resulting in the inability to link given *N‐*glycan composition to a specific microanatomical region of the tissue. Veličković, Sharma, et al. (2022) have investigated this problem and have optimized the incubation step by use of methods to maintain constant relative humidity in the incubation chamber. They tested saturated solutions of various salts and showed that the best performance was achieved using a saturated solution of KNO_3_ that maintained an 89% relative humidity, Under these conditions, near maximal sensitivity was achieved with only minimal ion delocalization. The method was demonstrated at a 35 μm spatial resolution with a kidney nephrectomy tissue section. Another digestion device that controls humidity, this time by cyclic ventilation and heating of the slide holder and the chamber lid has been developed by Fülöp et al. (2022). The device was designed to enable controlled micro‐condensation on the slide and to stabilize and monitor the digestion process. It was used to study sagittal mouse brain sections and xenografted human U87 glioblastoma cells in CD1 nu/nu mouse brain.

Although many methods have been developed for examination of FFPE soft tissues, problems arise for hard tissues such as cartilage‐bone, tooth and whole mouse body. For example, there can be loss of morphology during the heat‐induced epitope retrieval step on commercially available conductive ITO slides. To overcome the problem, Lee, Briggs et al. (2021) have taken conductive ITO slides precoated with gelatin and chromium potassium sulfate dodecahydrate to improve the adherence of FFPE human osteoarthritic cartilage‐bone tissue sections for monitoring *N*‐glycans. Tissues were sprayed with PNGase F and incubated for at least 2 h at 37^o^C. A peptide calibration standard was added followed by the CHCA matrix, which was sprayed on with the same system as was used for the enzyme. Scanning was conducted with a TOF/TOF instrument. Use of the gelatin‐coated ITO slides resulted in overall higher *N*‐glycan signal intensity not only for FFPE osteoarthritic cartilage‐bone tissue but also for FFPE hard‐boiled egg white used as a quality control.

Pace et al. (2022) have reported the first use of the hybrid technique, IR‐MALDESI for imaging *N*‐glycans in FFPE samples. IR Ionization has the advantage of minimizing losses of sialic acids and avoids the necessity for derivatization. The method was demonstrated with FFPE embedded human prostate tissue, which was analyzed in negative ionization mode after pneumatic application of PNGase F to cleave the glycans. The mass spectrometer was an Orbitrap Exploris 240 with a resolving power of 240,000 (FWHM at 200 *m/z*). Fifty‐three *N*‐linked glycans were identified; more than 60% contained sialic acid residues.

Amidation with aniline of sialylated *N*‐glycans in FFPE tissue samples from human laryngeal cancer patients has been reported to provide increased detection sensitivity. After dewaxing, the sialic acids were amidated, the *N*‐glycans were released by spraying a solution of PNGase F and incubating at 37^o^C for 12 h and the glycans were examined by MALDI‐TOF after spraying with CHCA. Identification was by database matching (Zhang, Shi, et al., 2022).

##### 7.6.1.2 Use of other enzymes

Quantification of hyaluronic acid (HA, **88**) in human skin sections has been achieved following incubation with hyaluronidase (H1136). The enzyme was sprayed onto the tissue with a TM sprayer and the tissue was incubated at 37^o^C for 18 h. For MALDI analyses, the matrix was DHB/DMA, which was also sprayed onto the tissue. HA was detected in each skin section in negative ion mode by targeting its specific digestion fragment (6‐mer, **88**, *n* = 3) at *m/z* 1180.2900 ([M−3H+2Na]^−^ ion). The method was said to be better than existing methods based on Raman imaging or use of a biotinylated HA‐binding protein and was used to measure the HA concentration in the epidermis, upper dermis, and lower dermis following treatment with a cosmetic formulation (Legouffe et al., 2022).


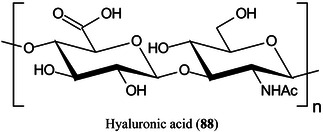


##### 7.6.1.3 Use of several enzymes

Serial treatment of FFPE tissue sections with several enzymes has been used to study constituents from the extracellular matrix from aortic valve sections (Clift, Drake, et al., 2021). For example, chondroitin sulfates were imaged after treatment with a chondroitinase, after which, treatment with PNGase F allowed *N*‐glycans to be studied. Peptides were then imaged by treatment of the tissues with elastase. Imaging was performed with an FT‐ICR instrument with CHCA as the matrix. A protocol for the method, aimed at fibrosis research has been published (Clift, Mehta, et al., 2021).

Denti et al. (2022) have developed a multiomics approach for visualizing lipids, *N*‐glycans and tryptic peptides on a single slide. The slides were first heated for 1 h at 60°C followed by two washes for 8 min each with toluene. The 9‐AA matrix was sprayed onto the surface with a TM‐Sprayer and Phosphorus Red, used as a calibration standard, was then spotted onto the slide, which was analysed by MALDI‐TOF. Next, the 9‐AA was removed and rehydration was performed with consecutive washes in 100% ethanol (1 × 3 min), 70% ethanol (1 × 3 min), and H_2_O (2 × 2 min). A citric acid antigen retrieval step was performed in a bath of citrate buffer (pH 5.9, 10 mM) at 97°C for 45 min before washes in H_2_O (20 min). PNGase F from *Elizabethkingia meningoseptica* was deposited using an iMatrixSpray and the slide was incubated overnight in a humidity chamber at 42°C. Finally, CHCA in 70% acetonitrile solution was sprayed using a TM‐Sprayer and MALDI‐TOF was again performed. The CHCA was then removed from the slides and rehydration was performed as above. Trypsin deposition (20 ng/μL) was performed using an iMatrixSpray and the slides were incubated in a humidity chamber overnight at 40°C. Finally, a solution of CHCA in 70% acetonitrile with 0.1% trifluoroacetic acid was applied with a TM‐Sprayer and the slide was examined by MALDI‐TOF. The method was applied to murine brain and renal carcinoma tissue providing complementary information that characterized different histological regions.

PNGase F/Endo F3 Glycan release, combined with neuraminidase digestion have provided a means to improve sensitivity and provide more information on chain branching, Enzymes were sprayed onto deparaffinised tissue slices and incubated for 2 h at 37^o^C (DelaCourt et al., 2022).

### 7.7 On‐tissue derivatization

On‐tissue derivatization has been used by several investigators to improve detection of specific compounds. The topic has been reviewed: “On‐tissue chemical derivatization reagents for matrix‐assisted laser desorption/ionization mass spectrometry imaging” (82 references) (Merdas et al., 2021). This is a general review but contains a small section on carbohydrates.

**Scheme 4:**
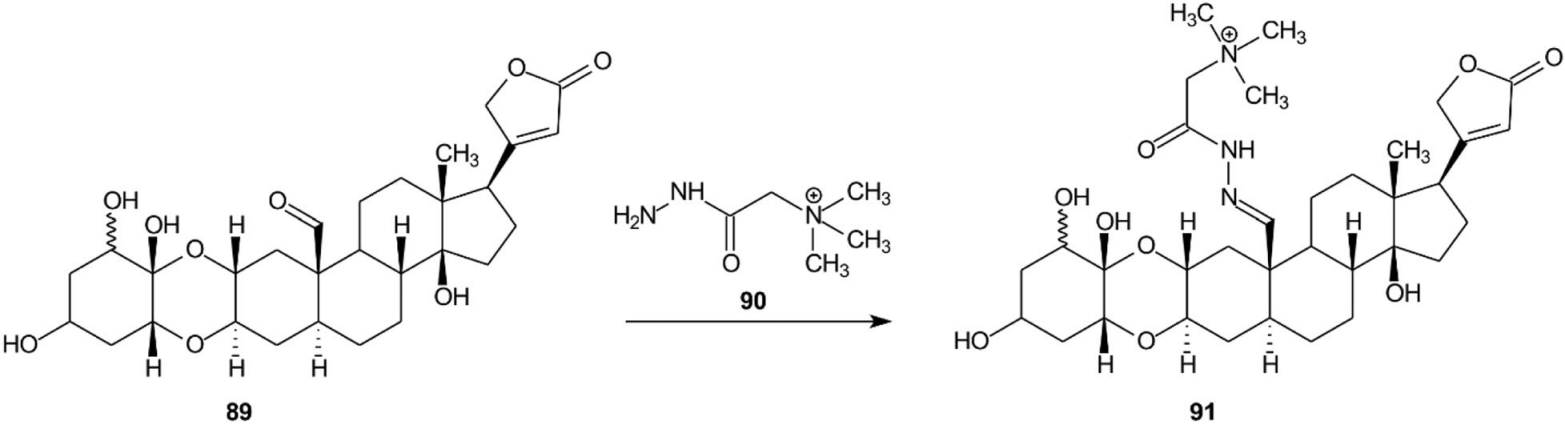
Derivatization of calotropin/calactin with Girard's T reagent (**90**).

A new method for analysis of steroid glycosides has been reported and used to improve the study of cardiac‐glycoside sequestration in *D. plexippus* (Dreisbach et al., 2022). The method involved derivatization of the 19‐oxo group of **89** with Girard's T reagent (Scheme 4) which gave an improvement of at least an order of magnitude over the use of underivatized samples.

**Figure 3:**
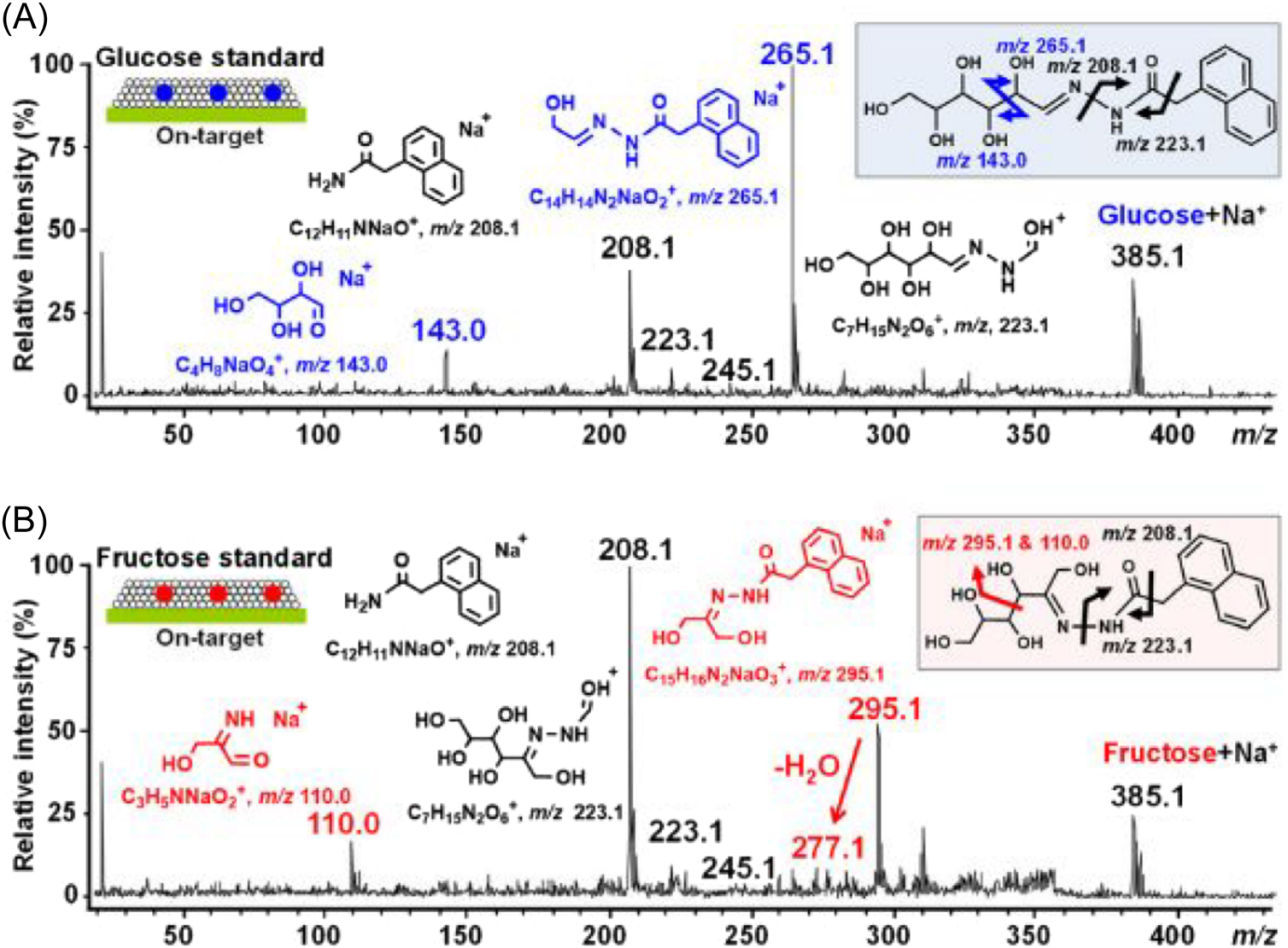
Formation of aldose‐ and ketose‐specific ions from the monosaccharides glucose and fructose following derivatization with NAH. From Han, Zhao, et al. (2022) with permission from Elsevier.


Han, Zhao, et al. (2022) have developed an on‐tissue derivatization method, to image and quantify the aldose and ketose isomers of monosaccharides in biological tissues. The new derivatization reagent, 1‐naphthaleneacethydrazide (NAH, **92**) was synthesized and was shown to significantly enhance the sensitivity of detection of the monosaccharides. In addition, the NAH‐derivatized aldose and ketose monosaccharides gave isomer‐specific diagnostic ions upon fragmentation (see Figure 3). Specifically, aldose carbohydrates, illustrated by glucose, gave *m/z* 265 and 143, whereas the keto‐monosaccharides, illustrated by fructose, gave *m/z* 295 and 119. For derivative formation, a solution of the reagent (0.5 mg/mL in acetonitrile/acetic acid (7:3 [v:v]), was sprayed onto the tissue and incubated for 2 h at 60^o^C. MALDI used 1,5‐DAN as the matrix. A quantitative method was also developed and applied to tissues from strawberry, carrot, mulberry, and burdock.

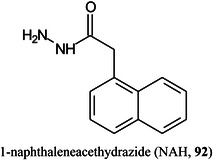


4‐(Dimethylamino)phenylboronic acid (DBA, **93**), applied with a TM sprayer, has been used to derivatize *cis*‐diol metabolites, including several carbohydrates, in cryo‐sectioned tissues from maize. The presence of the derivative improved the signal from the target compounds and identification was facilitated by use of the ^10^B/^11^B isotope pattern (Forsman et al., 2021).


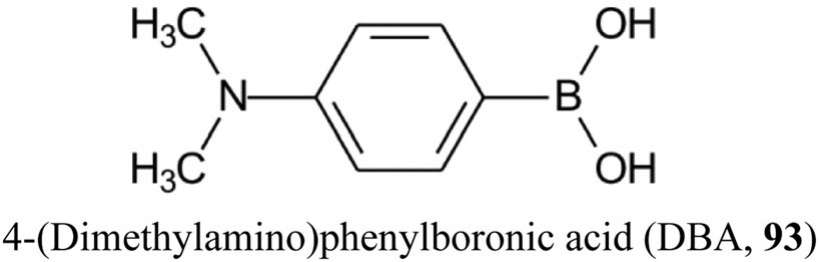


The use of multiple derivatization agents in parallel increases metabolite coverage even further but produces large and complex datasets that can be challenging to analyze. To address this problem, Larson et al. (2022) have developed “Metaspace,” for annotation of results from multiple derivatization experiments. Maize roots were used as a model system to obtain MSI data sets after parallel chemical derivatization with four different reagents, Girard's T (**90**, Scheme 4) and P (**94**) for carbonyl groups, coniferyl aldehyde (**95**) for primary amines, and 2‐picolylamine (**96**) for carboxylic acids. Using this method, 631 unique metabolites were identified compared with 256 from the underivatized data set. Analysis time was also shorter. An additional feature is a method to remove false derivatized annotations, which can clean 5%−25% of these annotations from the derivatized data.


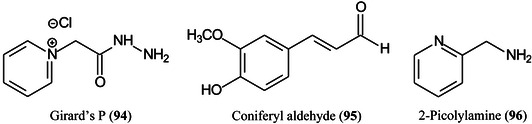


Protocols for various methods for MALDI imaging are listed in Table 4 and applications of MALDI imaging are listed in Table 5.

**Table 4:** Protocols for methods relating to matrix‐assisted laser desorption/ionization imaging.

Subject	References
Array‐based *N*‐glycan profiling of cells in culture	Angel, Mehta, et al. (2021)
Preparing ductal epithelial organoids for high‐spatial‐resolution molecular profiling	Bakker et al. (2022)
MALDI‐2 and t‐MALDI‐2 mass spectrometry imaging	Dreisewerd et al. (2022)
Matrix‐assisted laser desorption/ionization mass spectrometry imaging of glycogen in situ	Hawkinson and Sun (2022)
Ambient mass spectrometry imaging of small molecules from cells and tissues	Kim, Lim, et al. (2022)
Enhancing metabolite coverage for matrix‐assisted laser desorption/ionization mass spectrometry imaging through multiple on‐tissue chemical derivatizations	O'Neill, Dueñas, et al. (2022)
Single‐cell metabolomics with rapid determination of chemical formulas from isotopic fine structures	Samarah, Vertes, and Anderton (2022)
Mass spectrometry imaging of biological tissues by laser desorption ionization from silicon nanopost arrays	Samarah and Vertes (2022)
Sample preparation for imaging mass spectrometry	Shrestha (2021d)
MALDI Methods used in imaging, sample preparation, etc.	Shrestha (2021e)
Regional *N*‐glycan and lipid analysis from tissues using MALDI‐mass spectrometry imaging	Stanback et al. (2021)
MALDI Mass spectrometry imaging of lipids on free‐floating brain sections and immunohistochemically colocalized markers of neurodegeneration	Strnad et al. (2022)
TOF‐SIMS Imaging of biological tissue sections and structural determination using tandem MS	Van Nuffel and Brunelle (2022)
Optimization of multiple glycosidase and chemical stabilization strategies for *N*‐glycan isomer detection by mass spectrometry imaging in FFPE tissues	West, Lu, et al. (2021)

**Table 5:** Imaging.

Target compound	MALDI	Notes	References
Acylated anthocyanins in rat jejunum membranes (e.g., cyanidin glucoside [**97**])	TOF/TOF **(DHB**, ImagePrep sprayer)	Analysis of intestinal absorption of acylated anthocyanins in Sprague–Dawley rats	Hahm et al. (2021)
Alzheimer's brains (mouse and human)	PNGase F (TM sprayer), Q‐IM‐TOF (Waters Synapt G2‐Xs), (**CHCA**)	In situ spatial glycomic imaging. Changes in *N*‐linked glycosylation detected	Hawkinson, Clarke, et al. (2022)
Aminoglycoside and vancomycin antibiotics from mice and rabbit lung	FT‐ICR (**DHB**, spray)	Development of an optimized method for the detection and spatial distribution of aminoglycoside and vancomycin antibiotics	Wang, Dartois, et al. (2021)
Anthocyanins and carbohydrates from strawberries	TOF/TOF (**DHB**, TM sprayer)	Distribution of strawberry plant metabolites at different maturity stages	Wang, Yang, Chaurand, et al. (2021)
*Arabidopsis thaliana* leaves (glycolipids)	IR ablation atmospheric pressure photoionization, TOF (**ice**, sublimation)	Imaging at the single‐cell level	Hieta et al. (2021)
Arabinoxylans in developing wheat grain.	*R*‐TOF (**DHB**/DMA, nebulizing robot)	Spatial correlation of water distribution and fine structure of arabinoxylans	Fanuel et al. (2022)
Asperosaponin VI (glycoside, **98**) in *Dipsacus asperoides* roots	TOF/TOF (**DHB**, TM sprayer)	Jasmonic acid biosynthesis and signalling shown to be associated with the biosynthesis of asperosaponin VI in *D. asperoides*	Xu, Hu, et al. (2022)
Carbohydrates, amino acids, monoamines in banana pulp	TOF/TOF (**AuNPs**)	Spatially resolved metabolomics reveals variety‐specific metabolic changes during postharvest senescence	Yin, Dong, et al. (2022)
Carbohydrates, amino acids, and various metabolites from *Arctium lappa* L. (burdock) roots	TOF/TOF (**DHB**.**CHCA**, spray)	Distribution of components in roots. Carbohydrates mainly in centre	Li, Qiu, et al. (2022)
Cellooligosaccharides in plant cells	TOF (DMA/**DHB**, nebulyser)	Real‐time imaging of enzymatic degradation of cellulose in pretreated maize internodes reveals different cell types have different profiles	Leroy et al. (2022)
*Clausena lansium* (Lour.) skeels	IT‐TOF (**DHB**/TFA, airbrush)	Visualizing the spatial distribution of metabolites	Tang, Zhao, et al. (2021)
Defensive cardiac glycosides in *Asclepias curassavica*	Orbitrap with 3D AP‐MALDI ion source, (**DHB**, pneumatic sprayer)	3D‐Surface MALDI mass spectrometry imaging for visualising plant defensive cardiac glycosides	Dreisbach et al. (2021)
Disaccharide isomers in plant tissues	*R*‐TOF/TOF (**NEDC**, ImagePrep sprayer, ‐ve mode)	MALDI‐TOF/TOF tandem mass spectrometry imaging reveals nonuniform distribution of disaccharide isomers	Zhan et al. (2021)
Ellagitannins in *Fragaria *×* ananassa* (strawberry)	TOF/TOF (**DAN**, vapour deposition and spray), MS/MS, LC‐MS	Study of distribution in fruit	Enomoto (2021)
Fructans in stem and rhizome of *Agave tequilana* Weber var. azul	Q‐TOF (**CHCA**)	Higher DP fructans found toward the central section of the stem, lower DP fructans concentrated in the highly vascularized central core of rhizomes	Pérez‐López, et al. (2021)
Galactosylated glycerols and other defense‐related metabolites in *Triticum* spp.	AP‐MALDI, Orbitrap (**DHB**, spray)	For mapping the spatial distribution of defense‐related metabolites	Righetti et al. (2022)
Ginsenosides (e.g., **99**) and other metabolites from *Panax notoginseng*	TOF/TOF (**9‐AA**, **CHCA**, **DAN**, TM sprayer)	Visualizing the distributions and spatiotemporal changes of metabolites	Sun, Ma, et al. (2021)
Ginsenosides in *Panax notoginseng*	Q‐TOF (**DHB**, **CHCA** [+ve], **9‐AA** [‐ve] spray), MS/MS	For unveiling the transformations of ginsenosides during processing	Fan, Yang, Li, et al. (2022)
Glucose metabolites in bovine lens	FT‐ICR (**9‐AA**, **DAN**, **DHB**, **NEDC**, TM sprayer, ‐ve)	Development of method, NEDC matrix best	Zahraei et al. (2021)
Glucose in bovine lens cortex	FT‐ICR (**NEDC**, TM sprayer)	Mapping uptake, transport and metabolism	Zahraei et al. (2022)
Glycoalkaloids in potato tubers	QIT‐TOF (**DHB**, sublimation/spraying)	Distribution and changes of glycoalkaloids in potato tubers under different storage times	Deng, He, et al. (2021)
Glycogen and *N*‐glycans from human cancerous tissue (various)	PNGase F, IM‐Q‐TOF (**CHCA**, spray)	Imaging reveals heterogeneous glycogen stores in human normal and cancerous tissues	Young et al. (2022)
Glycosides and primary metabolites from bilberry (*Vaccinium myrtillus*) fruit	FT‐ICR (**THAP**, sublimation)	Determination of developmental distribution patterns	Dare et al. (2021)
Glycosides from *Gliricidia sepium* leaves	FT‐ICR (**DHB**, **CHCA**, **MBT**, nebulizer), LC	Optimization of imaging conditions and comparison with ESI	Pereira et al. (2022)
Glycosphingolipids from mouse retina	FT‐ICR (**DHAP**, sublimation, ‐ve)	Ganglioside GD3 synthase deletion shown to alter retinal structure and impair visual function in mice	Abreu et al. (2021)
Glycosphingolipids in ovarian cancer tissue	TOF/TOF (**DAN**, TM‐sprayer, +ve, ‐ve)	Glycosphingolipids shown to be mediators of cancer plasticity through independent signalling pathways	Cumin et al. (2022)
Glycosphingolipids in Gaucher disease mouse brain	TIMS‐TOF (**DHB**, sublimation)	Study of neuroinflammation in neuronopathic Gaucher disease	Boddupalli et al. (2022)
Lipids and glycolipids from postmortem human brain tissues	*R*‐TOF/TOF (**DHB**, +ve, **DAN**, ‐ve sublimation)	Regional lipid expression abnormalities shown to correspond to MRI‐defined white matter hyperintensities	Pinsky et al. (2021)
Lipids and glycolipids from rat brain	TOF/TOF (**s‐DHB**, **DHB**, **CHCA**, nebulizer)	To confirm Raman imaging study of posttraumatic stress injury	Chaichi et al. (2021)
Lipids and glycolipids from mouse brain	TOF/TOF (**CHCA**, spray)	Profiling changes in lipids over time under a high fat diet	Sighinolfi et al. (2021)
Lipids and sucrose in peanuts	TIMS‐TOF (**DHB**, TM‐sprayer)	Distribution of lipids	Wang, Chen, Liu, et al. (2022)
Lipids, including HexCer from human kidney	LTQ‐Orbitrap (**DAN**, ‐ve, sublimation)	High resolution imaging (10 μm)	Martín‐Saiz et al. (2021)
Lipids, including sulfatide, in cave‐dwelling fish	FT‐ICR (**DHB**/**AgNP**s, spray)	Study of lipid metabolic pathways underlying troglomorphic adaptations	Lam et al. (2022)
Lipids including sulfatide from mouse kidney	AP‐MALDI (Orbitrap), (**DHB** +ve, **norharmane**, ‐ve, spray)	In study of perfluorooctane sulfonate‐induced nephrotoxicity	Chen, Jiang, et al. (2022)
Maize tissue (metabolites)	LTQ‐Orbitrap (**DHB**, **CHCA**, **DAN**, TM sprayer)	Use of on‐tissue boronic acid derivatization for the analysis of *vicina‐*diol metabolites	Forsman et al. (2021)
Mannosylerythritol lipids in human skin cells	Ion trap	Studies of recovery effect on damaged skin cells	Kondo, Yasui, et al. (2022)
Metabolites such as gallotannins in *Paeonia suffruticosa* and *Paeonia lactiflora* roots	FT‐ICR (**DHB** and **DHB‐Li**, spray)	Spatial distribution of metabolites in roots	Li, Ge, et al. (2021)
Metabolites (e.g., rutin, **100**) from *Forsythia suspensa*	TOF/TOF (**DAN**, spray, ‐ve ion)	Spatial distribution of functional metabolites at different harvest stages	Jing et al. (2022)
Metabolites (various) in mouse kidney	FT‐ICR (**9‐AA**, **DAN**, **NEDC** [preferred])	Use of stable isotopes to monitor metabolic activity	Wang, Xing, et al. (2022)
Metabolites (various) in rat kidney	Orbitrap, TOF/TOF (**DAN**, TM sprayer)	Identification of tissue‐specific metabolic reprogramming	Wang, Fu, et al. (2021)
Metabolites in a transgenic mouse model of Alzheimer's disease	TOF/TOF (**4‐AC**, spray)	Use of 4‐AC as a new matrix	Chen, Hu, et al. (2022)
Metabolites from plant roots	FT‐ICR (**NEDC**, spray)	Elucidating drought‐tolerance mechanisms in plant roots	Honeker et al. (2022)
Metabolites (trehalose) from *Sphagnum* (peat moss)	FT‐ICR (**DHB/CHCA** (+ve), **NEDC** (‐ve), TM sprayer)	Novel metabolic interactions and environmental conditions shown to mediate the boreal peat moss‐cyanobacteria mutualism	Carrell et al. (2022)
Metabolites in continuously cropped *Salvia miltiorrhiza* Bge	TOF/TOF (**1,5‐DAN, BNDM**, TM sprayer)	Visualization of the spatial distribution and alteration of metabolite profiles	Sun, Cui, et al. (2022)
*N*‐Glycans from aortic valve tissue	PNGase F, FT‐ICR (**CHCA**, TM sprayer), *N*‐glycans	Spatial *N*‐glycomics of the human aortic valve in development and pediatric endstage congenital aortic valve stenosis	Angel, Drake, et al. (2021)
*N*‐Glycans in colorectal cancer	PNGase F, TOF/TOF (**CHCA**), *N*‐glycans (amide derivatization)	Cancer cells found to have higher levels of sialylation and high‐mannose glycans, together with less fucosylation and branching	Boyaval et al. (2021)
*N*‐Glycans in endometrial cancer tissue (FFPE preparation)	PNGase F (spray), *R*‐TOF/TOF (**CHCA**), *N*‐glycans	Detection of altered *N*‐linked glycosylation in endometrial cancer	Mittal et al. (2021)
*N*‐Glycans from alcoholic FFPE mouse liver	PNGase F, TOF/TOF (**DHB**, airbrush), *N*‐glycans, sialic acid benzylation	Investigation of benzylamidation of sialic acids.	Saito et al. (2021)
*N*‐Glycans from pancreatic cancer tissue	PNGase F or endo F3, FT‐ICR, Q‐TOF (**CHCA**, TM sprayer), amidation of sialic acids	Imaging of *N*‐glycans, high‐mannose, bi‐, tri‐, tetra‐antennary complex. Increased sialylation in cancer tissue	McDowell et al. (2021)
*N*‐Glycans from striatal neuroinflammation in the rodent brain	PNGase F, Q‐TOF (**CHCA**, spray)	Neuroinflammation caused a significant decrease in the abundance of sialylated and core fucosylated structures and an increase in high‐mannose glycans	Rebelo et al. (2021)
*N*‐Glycans from mouse brain	PNGase F, Q‐IM‐TOF, FT‐ICR (**CHCA**, spray)	Brain glycogen shown to serve as a critical glucosamine cache required for protein glycosylation	Sun, Young, et al. (2021)
*N*‐Glycans from 15 types of cancer tissue	PNGase F, TOF/TOF (**CHCA**, TM sprayer), high‐mannose *N*‐glycans	Re‐evaluation of previous data and re‐examination of tissues to evaluate contribution of high‐mannose *N*‐glycans to cancer	Chatterjee et al. (2021)
*N* Glycans from canine glioma	PNGase F, TOF (**DHB**)	Identification of biantennary glycan on haptoglobin	Malaker et al. (2022)
*N*‐Glycans in soybean root nodules	PNGase H, F, (FT‐ICR (**CHCA**, TM‐sprayer)	Spatial mapping provides insights into legume‐rhizobia symbiosis	Veličković et al. (2022)
*N*‑glycans in human knee osteoarthritis tissue	PNGase F, *R*‐TOF/TOF (**CHCA**, spray)	Identification of complex‑type *N*‑glycans as putative cartilage degradation markers	Lee, Briggs, et al. (2022)
Oligosaccharides from maize kernels	Orbitrap (**THAP**, spray)	Imaging following in situ enzymatic treatment	Granborg et al. (2022)
Oligosaccharides from FFPE slides from pancreatic ductal adenocarcinoma	FT‐ICR (**9‐AA**, spray), free glycans	Native glycan fragments shown to be independent prognostic factors of cancer	Sun, Trajkovic‑Arsic, et al. (2021)
Oligosaccharides, fatty acids, polyphenols from *Pisum sativum* seed coats	Q‐TOF (**DHB**, **THAP**)	Use of electronically driven micromanipulation and MALDI for analysis of seed coat layers in study of seed dormancy	Krejčí et al. (2022)
Planteose (**101**) in the parasitic weed *Orobanche minor*	iMScope TRIO **(DHB**, sublimation)	Study of the involvement of the enzyme in planteose hydrolysis during seed germination	Okazawa et al. (2022)
Polysaccharides in soybean root nodules	Laser desorption ionization from silicon nanopost arrays and MALDI, LTQ‐Orbitrap (**HABA, CHCA, 9‐AA, DHB**, nebulizer and airbrush)	Characterization of number and weight average molar mass, polydispersity, and oligomer size distributions across the tissue section. Comparison with MALDI	Samarah et al. (2021)
Prostate tissue	PNGase F, Q‐TOF (**CHCA**, spray), *N*‐glycans	Direct *N*‐glycan profiling	Blaschke, Hartig, et al. (2021)
Small molecules, including sugars, in brain tissues	TOF/TOF (**ZnO nanoparticles**, TM sprayer)	Use of zinc oxide nanoparticles as matrix	Chen, Laviolette, et al. (2021)
Steroidal glycosides in *Allium macrostemon* Bge. and *A. chinense* G. Don	TOF (**DHB**, TM sprayer)	Structural identification and structure/activity relationships	Duan et al. (2022)
Sucrose metabolite from *Vitis vinifera* L. (grapevine) infected with *Plasmopara viticola*	FT‐ICR (**DHB, CHCA, 9‐AA** [9‐AA poor])	Investigation of first moments of pathogen interaction	Maia et al. (2022)
Sugar phosphates and other metabolites from FFPE renal cancer tissue	FT‐ICR (**9‐AA**, spray), ‐ve mode	Identification of prognostic pathways and metabolites for renal cell carcinomas	Erlmeier et al. (2022)
Sulfatides in murine kidney	TOF (**9‐AA** [‐ve], **CHCA** [+ve] sublimation)	Identification of sulfatide with ceramide composed of phytosphingosine (t18:0) and 2‐hydroxy FAs in renal intercalated cells	Nakashima et al. (2022)
Sulfatides in rat brain	TOF (**9‐AA**, **DHB**, electrosprayed)	Imaging revealed sulfatide depletion in brain tissues of rats exposed in real air with high fine particulate matter	Diao et al. (2022)
Various endogenous compounds (mono‐, di‐saccharides, glycosides from Wolfberry fruit (*Lycium barbarum* L.)	QIT‐TOF (**DHB** [+ve], **9‐AA** [‐ve] sublimation)	Visualizing the spatial distribution of endogenous molecules at different development stages	Zhao, Zhang, Shi, et al. (2021)
Various from food	Orbitrap (**DHB**, pneumatic sprayer)	Demonstration of MALDI imaging for ingredients, contaminants and additives in processed food	Kokesch‐Himmelreich et al. (2022)


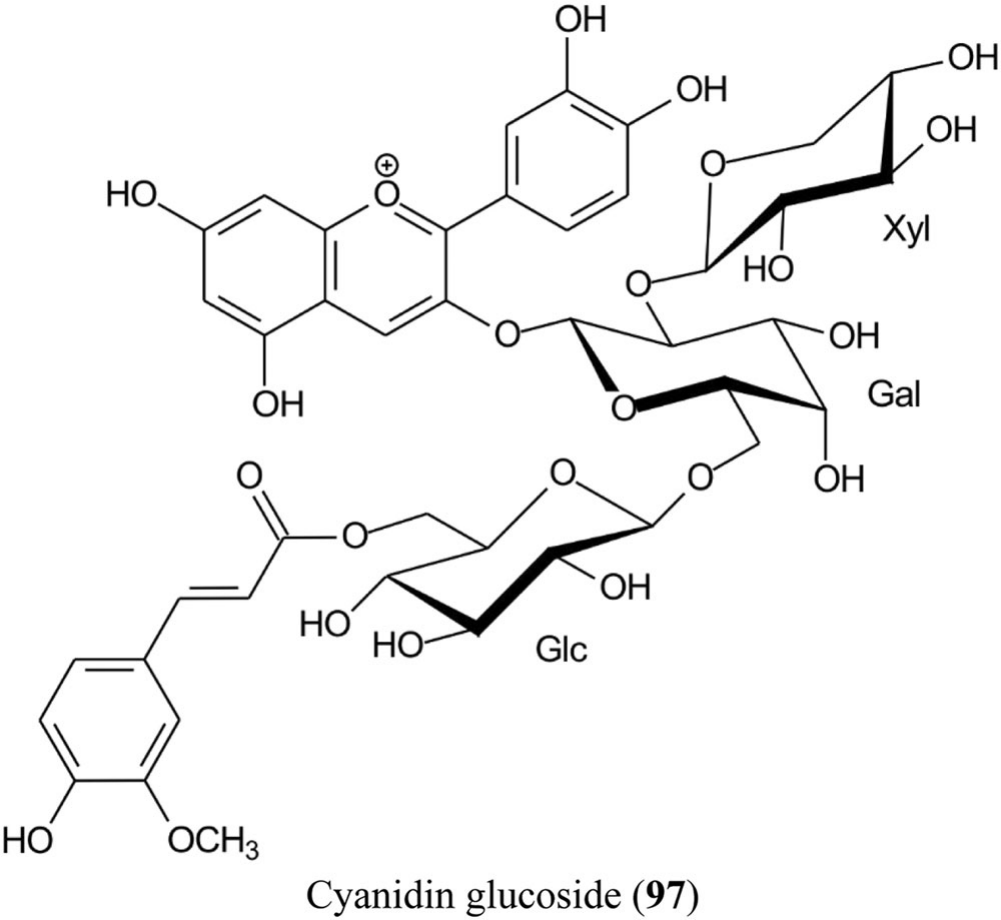



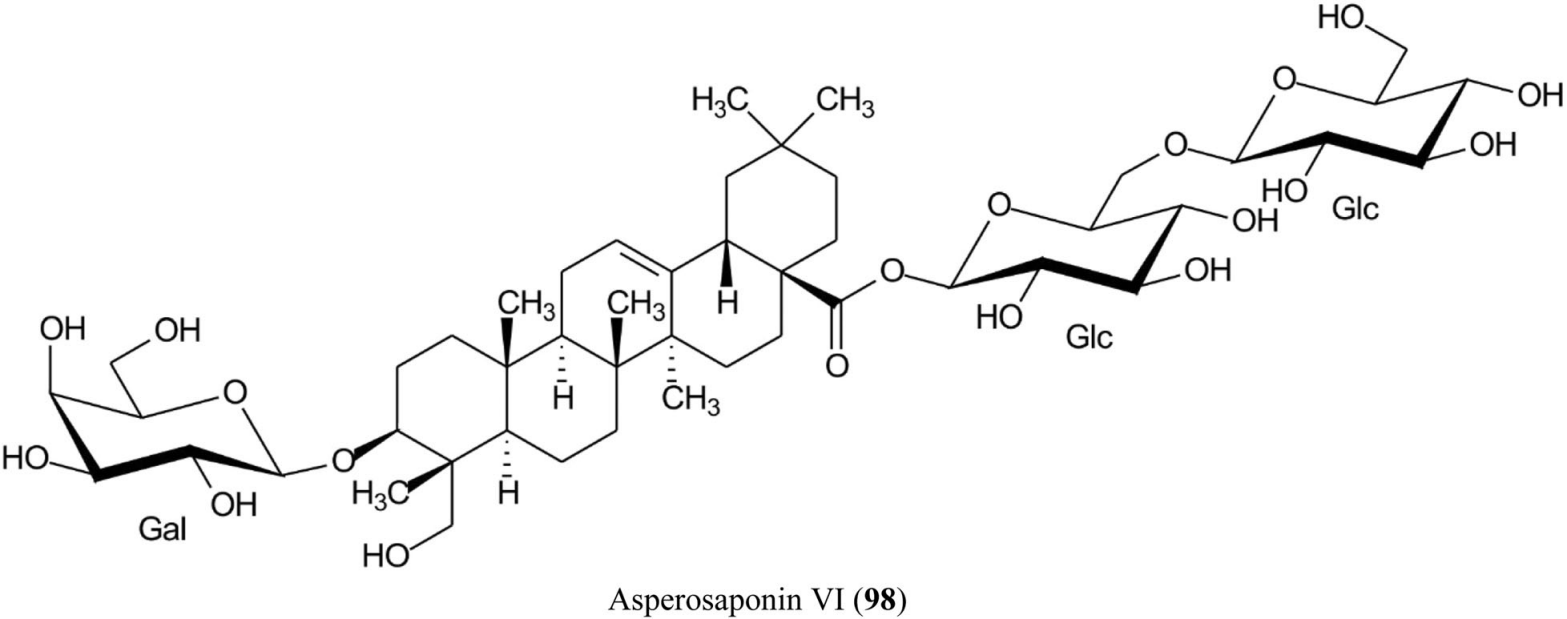



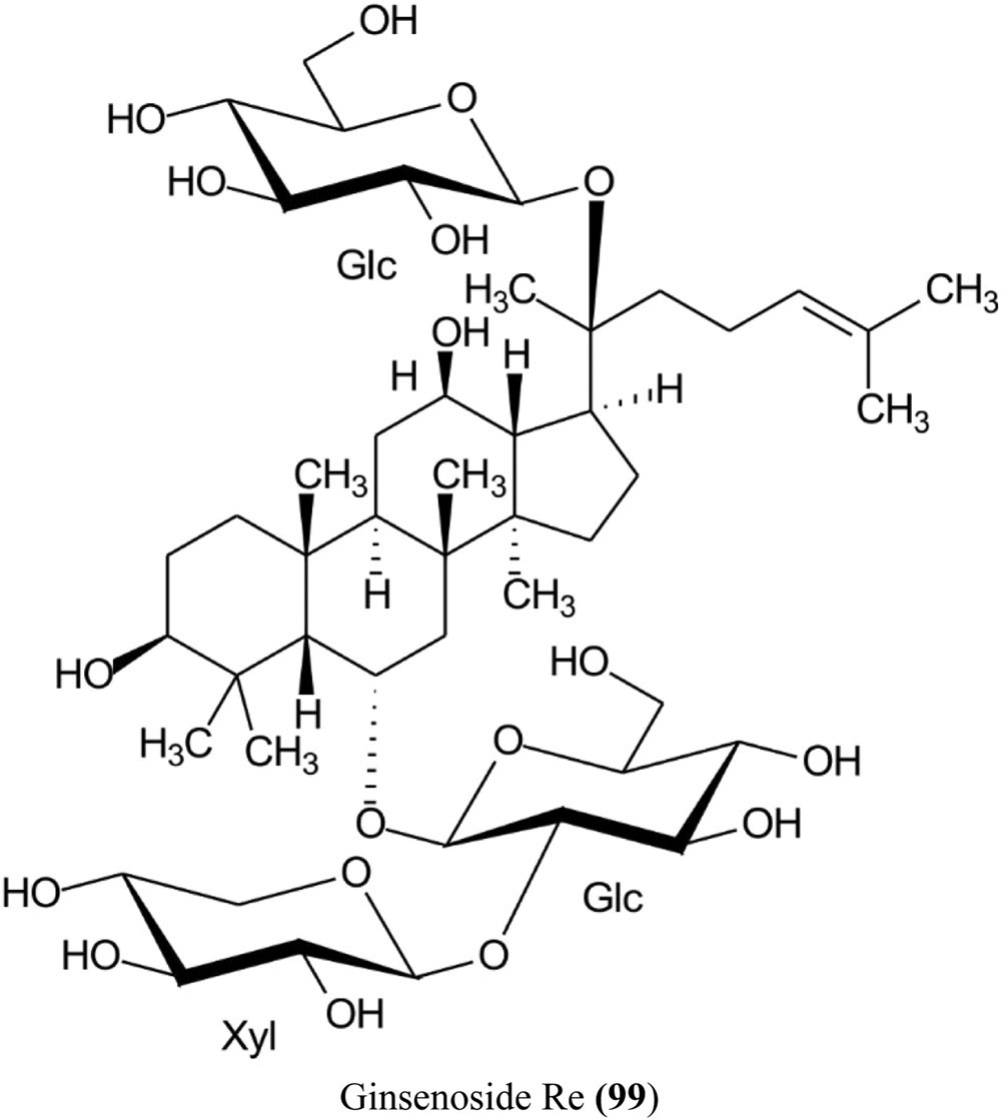



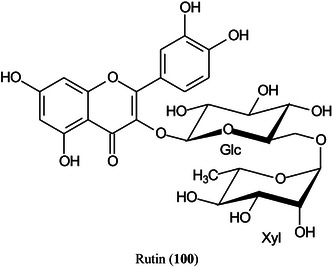



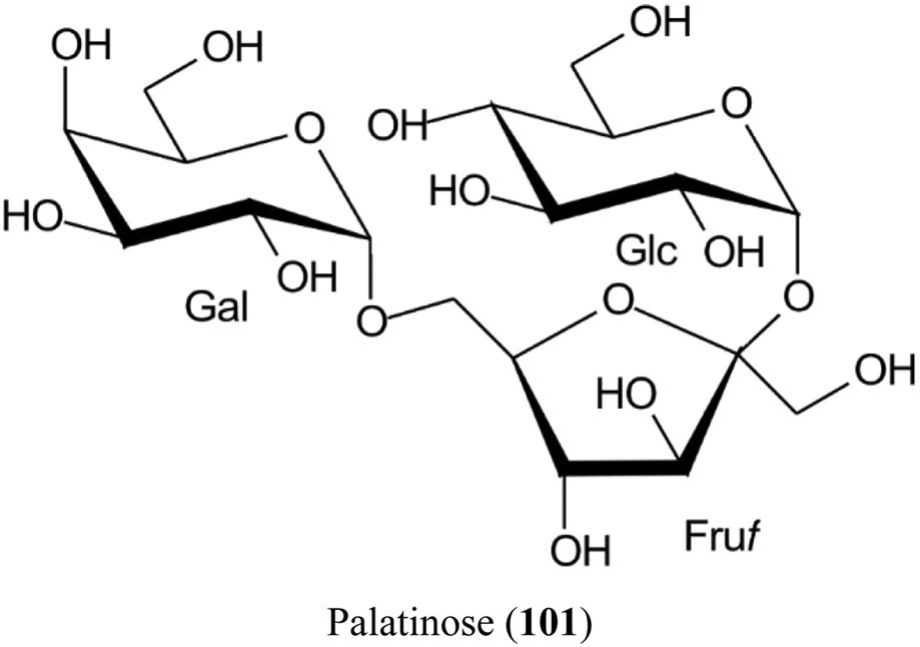


## 8 DERIVATIVES

Although reducing terminal derivatization is normally associated with the attachment of fluorescent derivatives for HPLC work, such derivatization methods can also attach moieties such as those containing a charge, that can enhance mass spectral performance. Several other derivatization methods such as permethylation and linkage‐specific derivatization of sialic acids are also used. Several reviews have appeared; these are listed in Table 6.

**Table 6:** General reviews on derivatives.

Subject	Comments	Citations	References
Recent advancements in glycoproteomic studies: Glycopeptide enrichment and derivatization	Brief; derivatization of glycopeptides, permethylation and derivatization of sialic acids	106	Pujić and Perreault (2022)
Targeting out of range biomolecules: Chemical labeling strategies for qualitative and quantitative MALDI MS‐based detection	Includes reductive amination reactions of carbohydrates	75	Sejalon‐Cipolla et al. (2021)
Chemical derivatization for mass spectrometric analysis of metabolite isomers	In Chinese, table and references in English	70	Wang, Li and Abliz (2021)
Derivatization of carbohydrates for analysis by liquid chromatography and capillary electrophoresis	Derivatization for various detection systems. Brief mention of MALDI	66	Yu, Dalman, et al. (2021)
Options of the main derivatization approaches for analytical ESI and MALDI mass spectrometry	Extensive review with large table showing derivative structures	410	Zaikin and Borisov (2022)

### 8.1 Reducing terminal derivatives

#### 8.1.1 Reducing terminal derivatives prepared by reductive amination

Reaction of the aldehyde group of the open chain form of reducing carbohydrates with an amine yields a Schiff base, which can be stabilized by reduction. Two protocols have been published for attachment of 2‐aminobenzamide (anthranilamide (2‐AB) **102**): “Profiling of *N*‐linked oligosaccharides of a glycoprotein by UPLC‐FLR‐ESI‐MS after derivatization with fluorescent anthranilamide” (Butré, Largy, & Delobel, 2021), and “Profiling, relative quantification, and identification of sialylated *N*‐linked oligosaccharides by UPLC‐FLR‐ESI/MS after derivatization with fluorescent anthranilamide” (Butré, Largy, Cantais, & Delobel, 2021). The 2‐AB reagent is used in excess and needs to be removed before mass spectral analysis. A method using a monolithic disc‐packed spin column has been reported (Yu, Dalman, et al., 2021). MonoSpin amide and MonoSpin‐NH_2_ columns showed the same efficiency as conventional solid‐phase extraction methods in the removal of the 2‐AB reagent and the recovery of the labelled glycans (Yui et al., 2022).


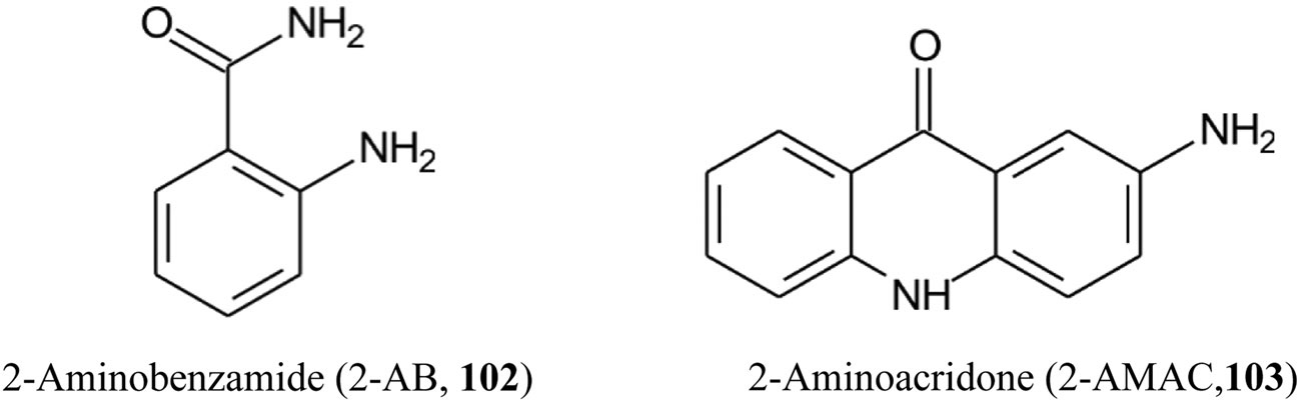


MALDI‐TOF/TOF has been used to characterize 2‐aminoacridone (2‐AMAC, **103**) derivatives of chitooligosaccharides in a study whereby they were encapsulated in alginate nanospheres to enhance bioavailability and antiliver fibrotic effects (Liu, Li, et al., 2022).

#### 8.1.2 Hydrazides

Benzenesulfonyl hydrazine (BSH, **104**) derivatives, whose identity was shown by MALDI‐TOF‐MS of their per‐methylated derivatives, have been shown to be suitable for glycan separation by 2D‐HPLC. Furthermore, the underivatized glycans could be recovered by heating at 70^o^C for 30 min. (Wang, Gao, et al., 2022).


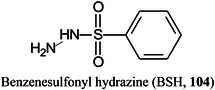


#### 8.1.3 Reducing‐terminal derivatives prepared by other methods

Reducing sugars have been shown to react with *N,O*‐dimethylhydroxylamine hydrochloride (DMHA, **105**) to form the substituted glycosylamine (**106**, shown for glucose) (Norberg et al., 2022). The derivatives gave good RP‐HPLC performance with a single peak for each sugar and enabled many isomers to be separated. The MALDI and ESI spectra were also reported to be excellent. Furthermore, as above, the free carbohydrates could be recovered quantitatively following mild acid hydrolysis with HCl or acetic acid.


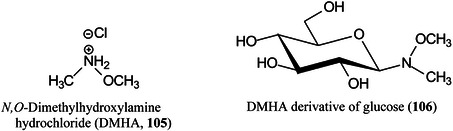


**Scheme 5:**
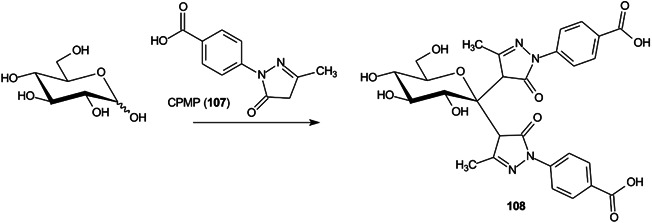
Formation of CPMP derivatives (**108**, shown for glucose).

1‐Phenyl‐3‐methyl‐5‐pyrazolone (PMP) has long been used as a derivatization reagent for carbohydrates. To produce high sensitivity, this reagent has now been modified (**107**, Scheme 5) to incorporate two carboxylic acids (CPMP) into the derivatized carbohydrate (**108**). The resulting derivatives gave exceptionally strong signals in negative ion mode. For derivatized disaccharides, the limits of detection (LODs) and limits of quantification (LOQ) ranged from 3.90 to 8.67 and 12.99 to 28.92 ng L^−1^, respectively (Ma, Chen, et al., 2022). Although analysis was by ESI, this derivative should work equally well by MALDI.

#### 8.1.4 Direct derivatization of PNGase F‐released glycans

**Scheme 6:**
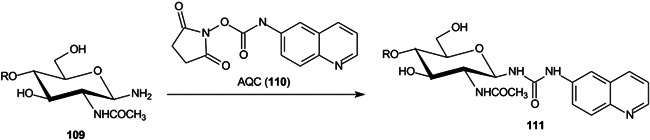
Derivatization of the reducing terminal GlcNAc residue of *N*‐glycans with AQC (**110**).

When glycoproteins are incubated with PNGase F, the *N*‐glycans are released as glycosylamines, which slowly hydrolyse to the native glycans. Some investigators have developed methods for derivatizing the glycosylamines directly and thus, are able to save time. One such reaction is with 6‐aminoquinolyl‐*N*‐hydroxysuccinimidyl carbamate (AQC, **110**) as shown in Scheme 6 (Wu, Zhang, Li, et al., 2022). The reaction is rapid and labelling was achieved in 20 min directly from the PNGase F incubation mixture.

#### 8.1.5 Derivative removal

**Scheme 7:**
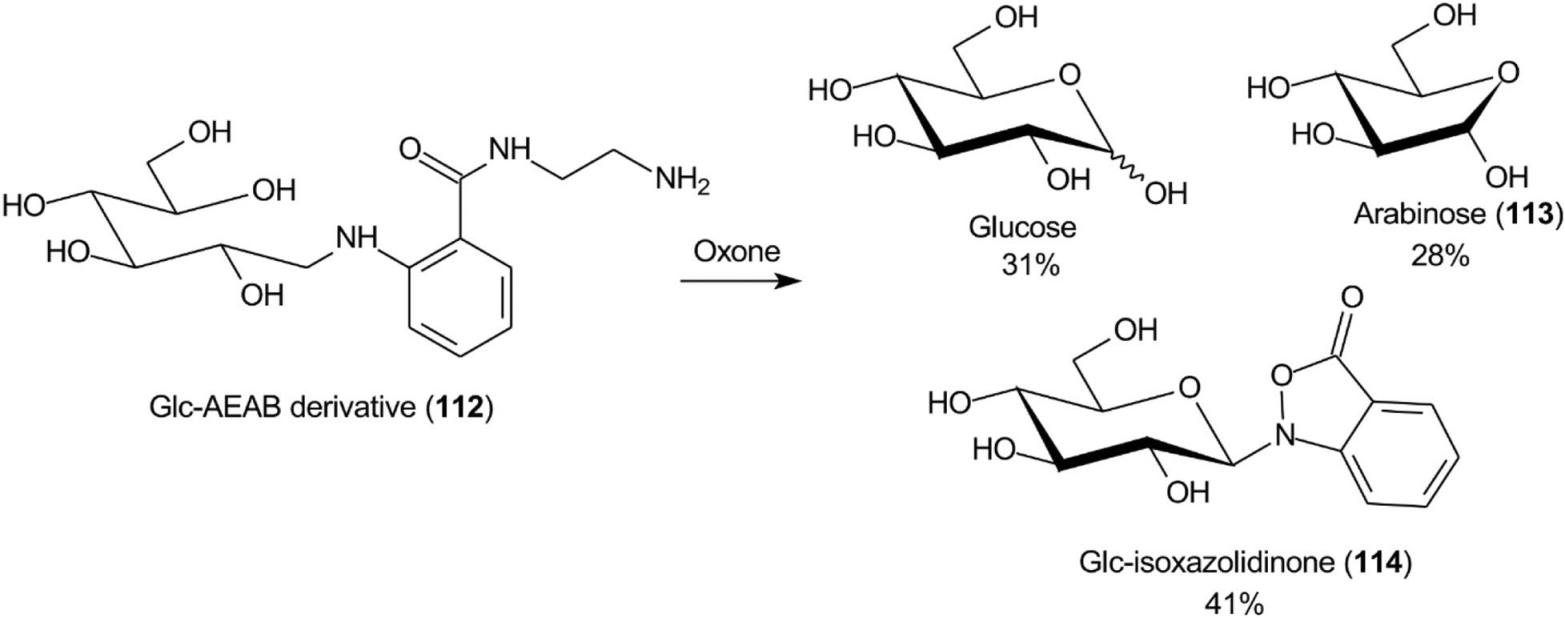
Derivative removal with Oxone.


A method for removal of a wide range of common florescent tags from glycans has been reported by Zhang, Wang, Li, et al. (2022). Glycans were incubated with 1% Oxone (trade name for 2KHSO_5_·KHSO_4_·K_2_SO_4_) containing 0.1% TFA for 0.5 h after which, the reaction mixture was purified with a RP‐C18 SPE cartridge. Unfortunately, reactions were not quantitative. Several products were generally produced as illustrated for glucose derivatized with 2‐amino(*N*‐aminoethyl) benzamide (AEAB (Scheme 7). Yields varied depending on the glycan and the derivative with glucose and lactose performing badly with only 31% and 27% of the required product for the AEAB derivative respectively, and 53% and 63% for the 2‐AB and 2‐aminobenzoic acid (2‐AA, **115**) derivatives respectively. *N*‐Acetylamino‐sugars, on the other hand gave high yields (e.g., 75% and 86% for GlcNAc [**117**] and GalNAc [**118**] respectively for the 2‐AA derivative. For aniline derivatives, the yield was 100% of the released glycan. 2‐aminopyridine (2‐AP, **116**) derivatives, however, failed to give a reaction, attributed to oxidation of the pyridine.

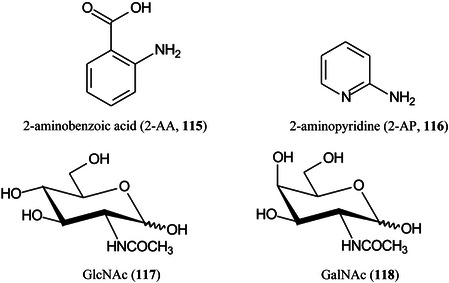


### 8.2 Derivatives of other sites

#### 8.2.1 Hydroxyl groups—Permethylation

Permethylation is possibly the oldest derivatization technique for carbohydrates and was originally used for gas‐liquid chromatographic (GLC) and combined GC/MS work. It featured prominently in a method for linkage analysis that is still used today. A tutorial on the technique has been published by Black et al. (2021). Later, it was used for fast‐atom bombardment (FAB) mass spectrometry and it still finds uses for improving sensitivity for MALDI analysis. Many examples can be found in Tables 12, 13, 15, 20, 21, 22, 33, 36, 39, 40, 43 and 50. Several methods of preparation have been published; an up‐to‐date one can be found in the paper by Cho, Banazadeh, et al. (2021).

Reaction conditions for solid‐phase permethylation have been optimized in another recent paper (Guan, Zhang, et al., 2021). The authors concluded that 10/100 (v/v) water/DMSO solvent gave the best results with 100 μL of iodomethane and 200 mg of sodium hydroxide beads and an incubation time of 10 min at room temperature. The method was said to minimize side reactions and inhibit the removal of *O*‐acetylation from sialic acids.

#### 8.2.2 Sialic acids

Sialic acids are generally unstable under MALDI conditions but they can be stabilized by ester (Powell & Harvey, 1996) or amide formation. Cheng, Shu, et al. (2022) have prepared four amides (with reagents **119**–**122**) of the carboxylic group of sialylated *N*‐glycans (characterized by MALDI‐TOF MS) and have used them to separate linkage isomers of several *N*‐glycans by microfluidic capillary electrophoresis‐MS. DMDT (**121**) Was chosen as the most satisfactory of the four reagents. As well as separating the linkage isomers, the migration times also revealed the number of sialic acids. Using this method, 52 sialylated *N*‐glycans were quantified in human serum within 10 min.


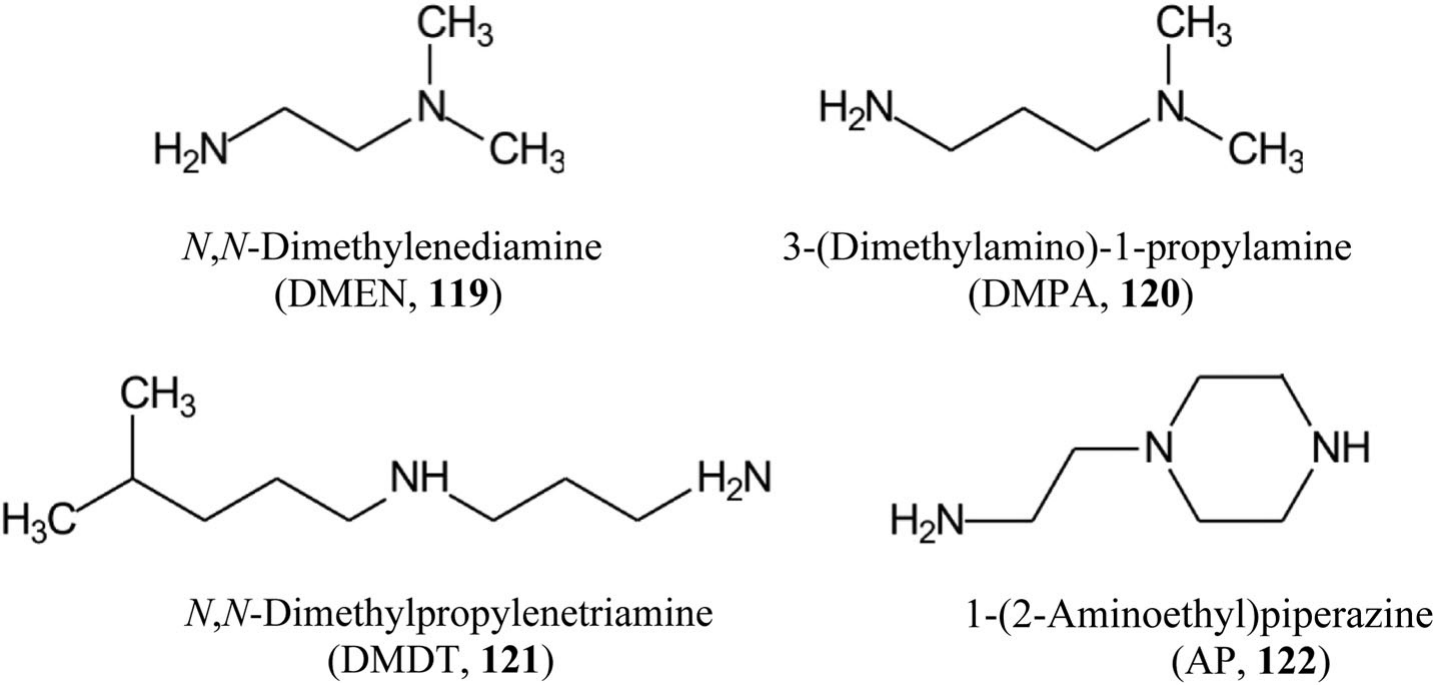


Amide formation is a popular alternative to ester formation. Wang, Zhang, Gao, et al. (2022) have used monomethylamides to study *N*‐glycans in cases of multiple myeloma, and Li, Zhang, et al. (2021) have employed this reaction in a study of *N*‐glycans in pediatric ulcerative colitis. Derivatives were prepared by mixing the sample with methylamine and (7‐azabenzotriazol‐1‐yloxy)*tris*‐pyrrolidinophosphonium hexa‐fluorophoshate (PyAOP, **123**) and allowing the mixture to incubate for 30 min at room temperature.


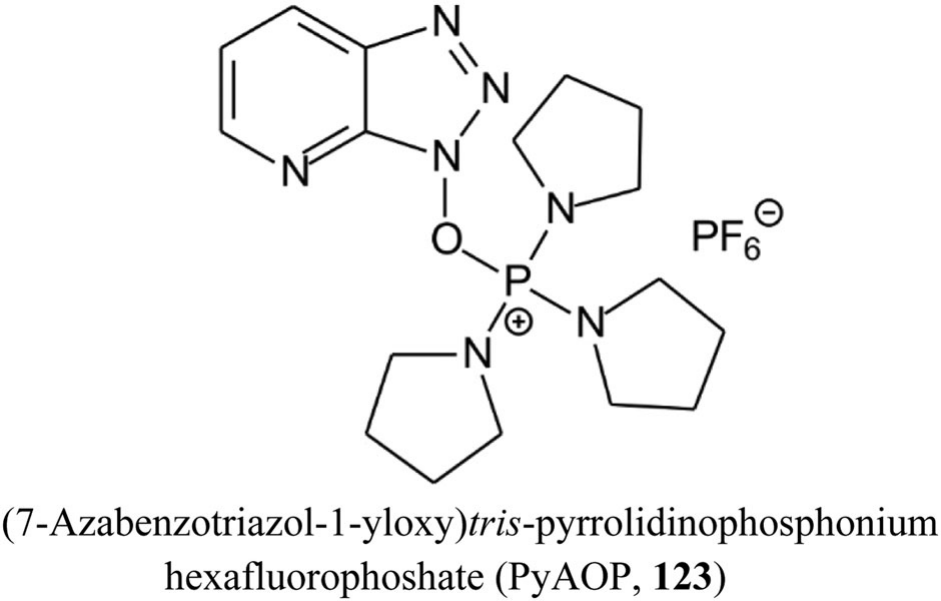


Jia et al. (2021) have used the same amides together with Girard's P derivatization to study changes in *N‐*glycans from bovine lactoferrin at different stages of lactation, and Cai, Ren, et al. (2022) have studied the urinary *N*‐glycome in diabetic kidney disease using similar methods. Rather than using PyAOP, Ret et al. (2022) have used the carboxylic acid activator 4‐(4,6‐dimethoxy‐1,2,3‐triazil‐2‐yl)‐4‐methylmorpholinium chloride (DMT‐MM, **124**) to form methylamides from *N*‐glycans. Benzylamidation has also been used in this context (Saito et al., 2021); the authors claim higher sensitivity than is produced by use of other amides.


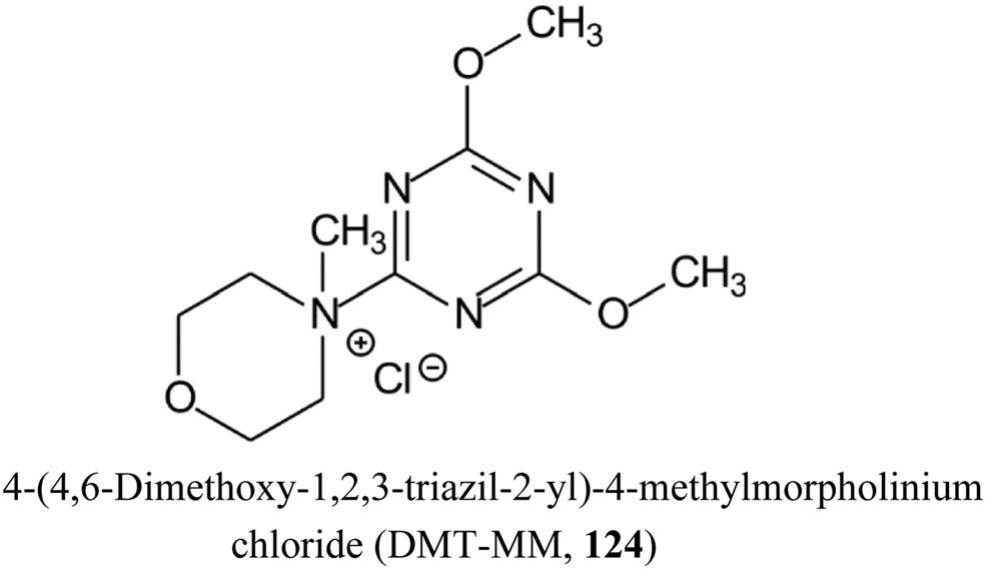


##### 8.2.2.1 Linkage‐specific derivatization

Under certain conditions, such as by the reaction with methanol in the presence of DMT‐MM, α2→6‐linked sialic acids form methyl esters, whereas the α2→3‐linked acids form lactones, The 32 unit mass difference allows the linkage to be determined by mass measurement (Wheeler et al., 2009). Smith, Millán‐Martín, Mittermayr, et al. (2021) have used this method to study sialylation of human serum glycoproteins. Rather than formation of methyl esters, investigators now prefer ethyl esters, prepared by use of 1‐ethyl‐3‐(3‐dimethylaminopropyl)carbodiimide (EDC, **125**) as a carboxylic acid activator and 1‐hydroxybenzotriazole (HOBt, **126**) as the catalyst (Aguedo et al., 2022; Cao, Zhang, et al., 2022; Van Coillie et al., 2022; Levink et al., 2022; Nummela et al., 2021; Pan, Zhang, Zhang, et al., 2021; Rubén et al., 2021; Xu, Jin, et al., 2022; Yaman, Kayili, et al., 2021; Zhang, Cao, et al., 2022; Zhang, Reiding, et al., 2021; Zhang, Wang, et al., 2021). *Iso*‐propyl alcohol has also been used for the α2→6‐esterification (Ohmi et al., 2021; Yang & Tian, 2022).


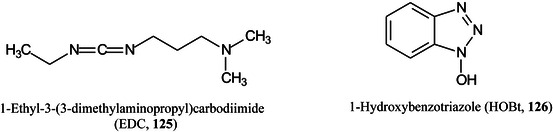


Several investigators have improved the original method, mainly by reacting the rather unstable lactone with a further amidation stage. An investigation of this latter reaction has confirmed that formation of the lactone is a prerequisite for amide formation and that the reaction involves direct amidation rather than prior hydrolysis of the lactone (Pongracz et al., 2021). The simplest reactions use the addition of ammonium hydroxide to convert the lactone from the α2→3‐linked acids to amides (Boyaval et al., 2022; Moran et al., 2022; Petralia, Santha, et al., 2022). Two publications have used *p*‐toluidine (**127**) as the second derivatization agent (Hyun et al., 2022; Wang, Wang, Zhang, et al., 2022). The α2→6 modification imparts a +28 amu tag whilst that of the *p*‐toluidine increases the molecular weight by 89 Da.


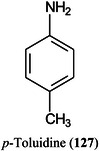


Amides have also been used in the first stage of the reaction as illustrated by the use of monomethyl‐ (Wang, Kałuża, et al., 2021) and dimethyl‐amides and ammonia (Alves et al., 2021; Zhu, Delbianco, et al., 2021). Ohmi et al. (2021), Yang and Tian (2022) have used *iso*‐propyl alcohol for the first esterification reaction and have followed it with methylamidation giving mass increases of 41.063 and 13.032 Da respectively, and the method has been adapted to allow it to be used with glycopeptides (Zhong, Huang, et al., 2021). The procedure also derivatized the COOH group of the peptide to give an approximately 4.6‐fold increase in signal intensity.

Many examples of these derivatization reactions employing use of several different alcohols for esterification and several amines for amidation, can be found in Tables 20, 21, 30, 36, 39 and 40. In a modification to allow α2→3‐linked acids to be specifically detected by electrophoresis (CE), the α2→6‐linked acids were amidated with methylamine and the α2→3‐linked acids were amidated with *N*,*N*‐dimethylethylenediamine (**128**) which could easily acquire a positive charge on the tertiary amine allowing it to separate from the isomer by CE. MALDI‐TOF was used to monitor the initial reaction with *N*‐glycans from fetuin (Cheng et al., 2021).


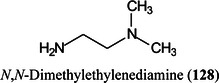


A high‐sensitivity method, termed derivatization of sialylated glycopeptides (DOSG), involves converting the α2→6‐ and α2→3‐linked acids in glycopeptides to *iso*‐propyl and methyl amides respectively. Carboxylic acid groups from amino acids are simultaneously converted into *iso*‐propyl amides (Zhong et al., 2021). An extension of this method, termed (DOSG+), which combines the linkage‐specific sialic acid derivatization with fixed charge derivatization has been developed (Zhong, Huang, et al., 2022). The sialic acids were reacted in a two‐stage process to convert the α2→6‐linked acids to *iso*‐propylamides and the α2→3‐linked acids to methyl amides. The acids were then reacted with sodium periodate to oxidize the side chain and the resulting aldehyde group was reacted with (2‐aminoethyl)trimethylammonium chloride hydrochloride (AETMA, **129**) by reductive amination to introduce a positive charge. Using a model glycopeptide, sensitivity increases of about 30% were reported.


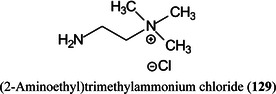


Jezková et al. (2022) have prepared alkyl esters of sialylated *N*‐glycans in a linkage‐specific fashion and then formed phenylhydrazone derivatives with phenylhydrazine (**130**). Under these conditions, the lactone ring was opened with the incorporation of a second phenylhydrazine moiety and phenylhydrazine was also added to the reducing terminal. Methyl rather than ethyl esterification was found to give the best results. The method was applied to the monitoring of sialylation in the serum of lung cancer patients.


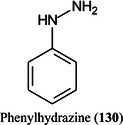


###### 8.2.2.1.1 Quantification

To avoid potential problems with quantification due to the fact that most of these methods convert the differently linked sialic acids into different compounds, Peng, Gu, et al. (2021) have used d_0_‐ and d_3_‐methylamide derivatization for the two stage reaction. Similar results were obtained with forward (d_0_‐ followed by d_3_) or backwards labelling. In a similar reaction, Jin, Li, et al. (2021) have used d_0_‐ and d_6_‐pyridine to label the α2→6‐ and α2→3‐linked sialic acids respectively. The *N*‐glycan solution was mixed with EDC, HOBt and d_0_‐aniline in DMSO and incubated at 60°C for 1 h. d_5_‐Aniline, together with additional EDC and HOBt in DMSO were then added and incubation was continued for a further hour. After purification, the glycans were derivatized with Girard's P reagent. Applications were to *N*‐glycans from glycoproteins in human colostrum and mature milk with a further study on human milk (Jin, Lu, et al., 2022).

### 8.3 Charged derivatives

Reagents with constitutive positive charges have been used to increase sensitivity in positive ion mode. Examples of the use of Girard's reagents T and P have been illustrated above and others are listed in Tables 15, 20, and 36. Another example is the imidazolium derivative (GITag, **131**) which has been synthesized and used to derivatize glycans by reductive amination (Zhang, Ghirardello, et al., 2021). Gains in sensitivity of up to 600 times over that provided by 2‐AB labelling were claimed for derivatized GlcNAc and lactose (**30**). The derivatization reaction could be conducted directly on the MALDI target following *N*‐glycan release with PNGase F and the reaction was demonstrated by examination of glycans released from human serum.


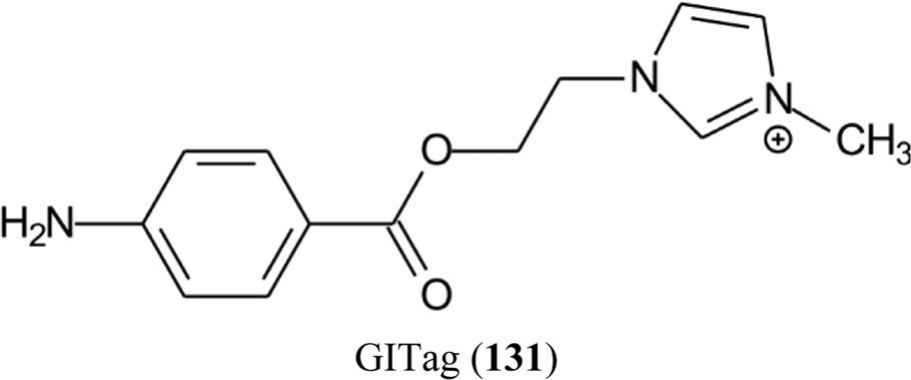


Although originally designed for CE studies (4‐hydrazidebutyl)triphenylphosphonium bromide (P_4_HZD, **132**), containing a permanent positive charge, has showed good MALDI performance. For example, the signals from derivatized maltodextrin were increased by an order of magnitude (Ma, Wang, et al., 2022). Larger oligomers were also detected when derivatized with this tag than were detected with the free sugars. Strong signals were also obtained from derivatized *N*‐glycans from therapeutic glycoproteins. Derivatization was said to increase the signal strength by more than the common labelling reagent, Girard's P, in both MALDI and ESI modes. Other examples of the use of Girard's P reagent are given in the section on linkage‐specific derivatization of sialic acids (Section *Linkage‐specific derivatization*).


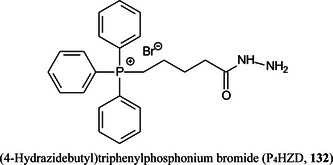


Constituents of ionic liquids, attached to various positions of the glycan molecules, including the reducing terminal, provide another method for attaching a fixed charge for high sensitivity. The subject has recently been reviewed (Ghirardello et al., 2022) and highlights tags such as **133**–**136**.


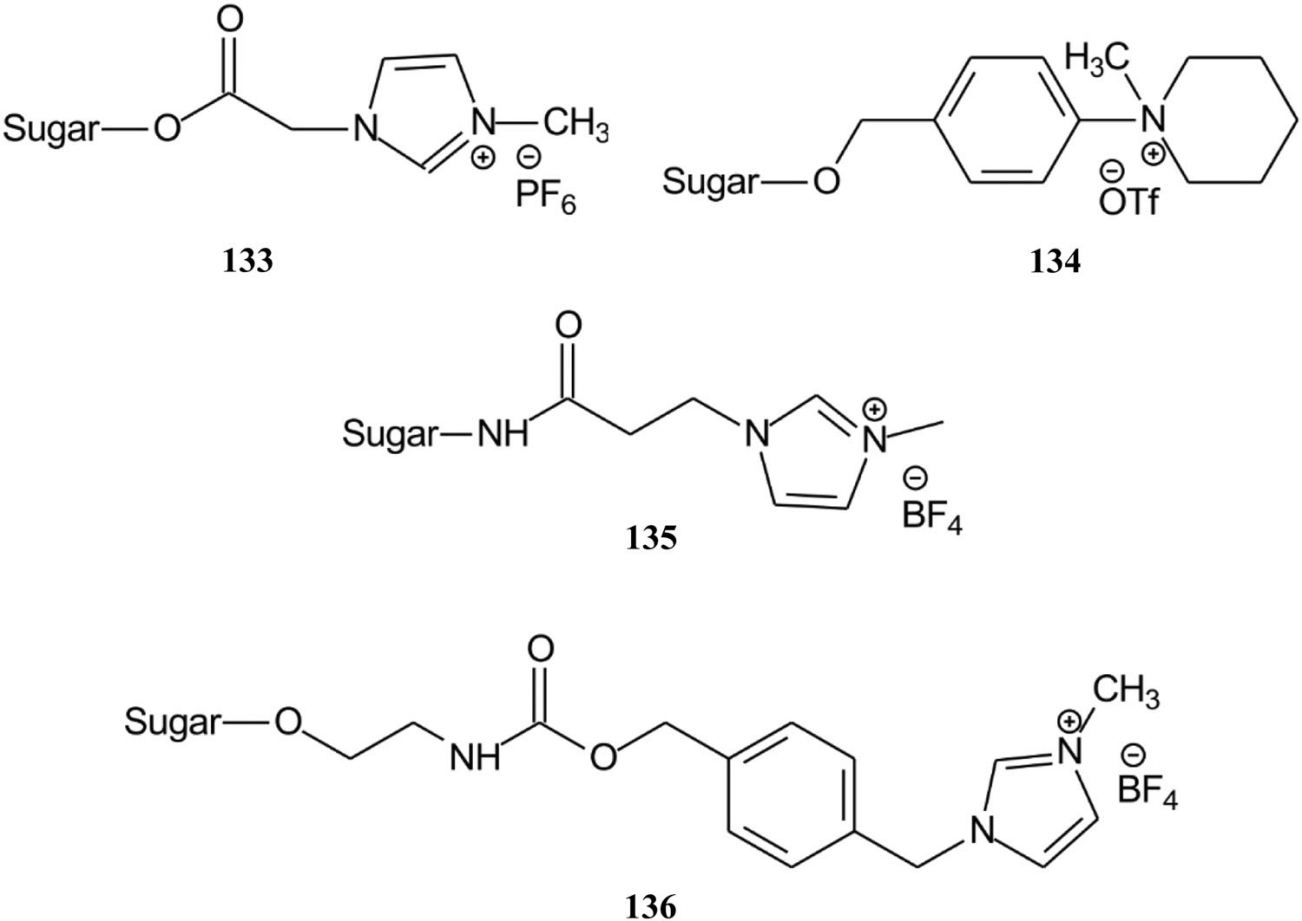


## 9 GLYCAN ARRAYS

A review with 138 references on glycan array technology has been published by Martinez et al. (2021). In a method to acquire *N*‐glycans for array construction Cao, Antonopoulos, et al., 2021) have used two‐dimensional hydrophilic interaction liquid chromatography and porous graphitized carbon chromatography to purify 31 *N*‐glycans from chicken ovalbumin. Purity of the glycans was estimated to be over 90% with identification by negative ion CID. The glycans were printed onto nitrocellulose‐coated glass slides and interrogated with wheat‐germ agglutinin, which mainly bound to hybrid‐type *N*‐glycans with a bisecting GlcNAc residue, compounds that were abundant in the released glycan mixture.

## 10 QUANTIFICATION

Reviews and general articles relating to quantitation of glycans and glycopeptides are listed in Table 7. “Methods to improve quantitative glycoprotein coverage from bottom‐up LC‐MS data” (Chang & Zaia, 2022) (136 references) is also of interest. Patabandige et al. (2022) have discussed quantitative clinical glycomics strategies and have concluded that there is no one best method and provide guidance on the best approach to take.

**Table 7:** Reviews and general articles on glycan quantitation.

Subject	Comments	Citations	References
Quantitative characterization of *O*‐GalNAc glycosylation	Summarizes the most common quantitative strategies and discusses benefits and limitations of the various approaches	51	Čaval et al. (2021)
Recent advances in analytical approaches for glycan and glycopeptide quantitation	Glycan and glycopeptide quantitation. Methods for isotope labelling, software.	208	Delafield and Li (2021)
Recent advances in qualitative and quantitative analysis of polysaccharides in natural medicines: A critical review	Discusses general and mass spectrometric methods	100	Li, Zhang, Han, et al. (2022)
Qualitative and quantitative methods for *N*‐glycans in *N*‐glycomics	Book chapter. General coverage of glycan analysis with some common quantitative methods	120	Ren and Lu (2022)
Isotope labeling strategies of glycans for mass spectrometry‐based quantitative glycomics	Mainly discusses isotope‐labelled derivatives	56	Yun et al. (2021)

### 10.1 *N*‐Glycopeptides

**Scheme 8:**
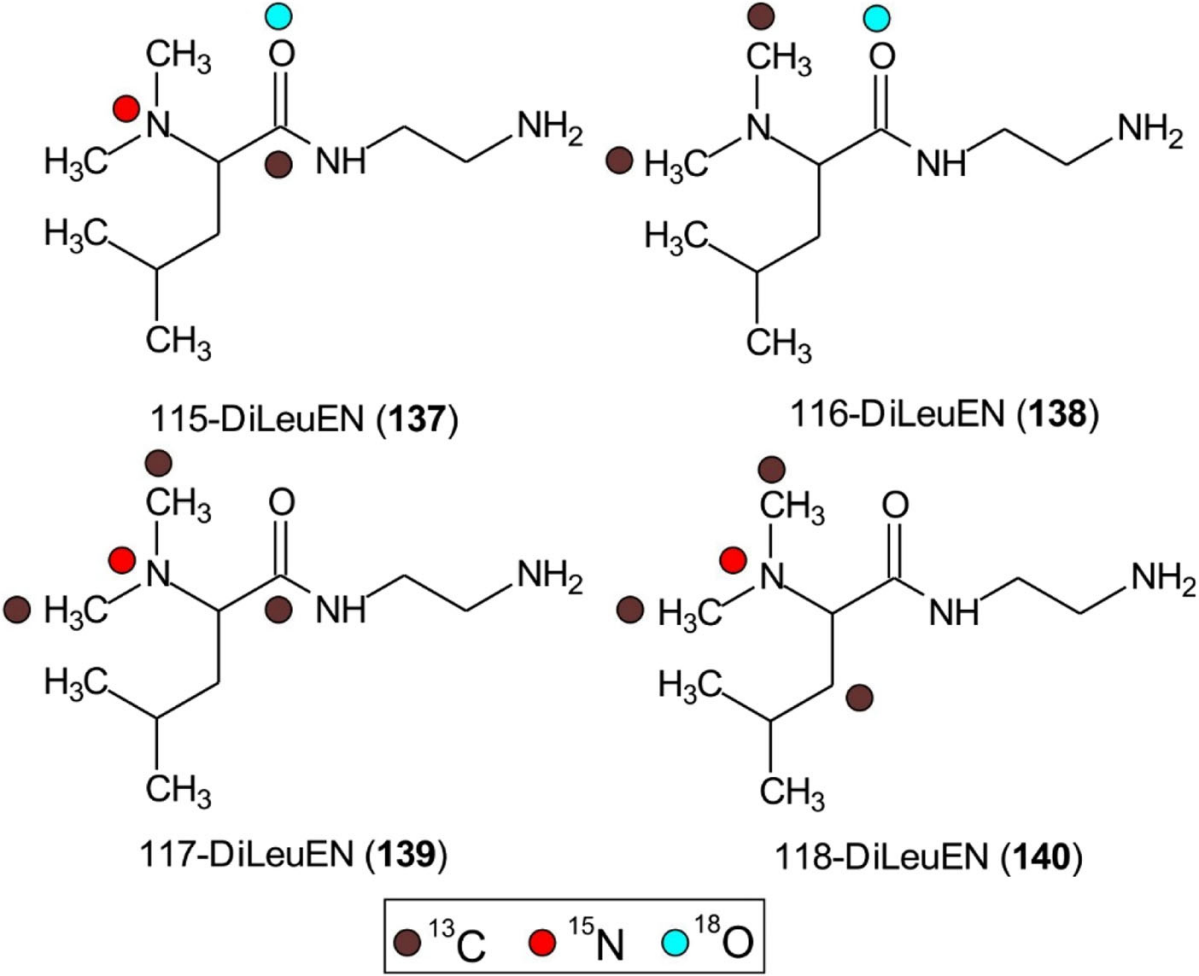
4‐Plex derivatives for glycopeptide quantification.

A 4‐plex method has been developed for quantification of glycopeptides. The reagents (DiLeuEN, Scheme 8) formed amides with the carboxylic acid groups and had the advantage of neutralizing the negative charge to improve sensitivity. The reporter ions allowed four samples to be quantified simultaneously (Li, Zhong, et al., 2022).

The lack of suitable standards for quantitation of glycopeptides from immunoglobulin G (IgG) has prompted the synthesis of fifteen such compounds (Wang, Liu, Qu, et al., 2021) by the attachment of ^13^C_6_‐fucose (Fuc, **14**), which introduced a 6 Da mass shift, to the core region of unfucosylated glycopeptides using the enzyme FUT8. The reference material was used to measure IgG glycopeptides in colon cancer sera.

### 10.2 *O*‐Glycans

**Scheme 9:**
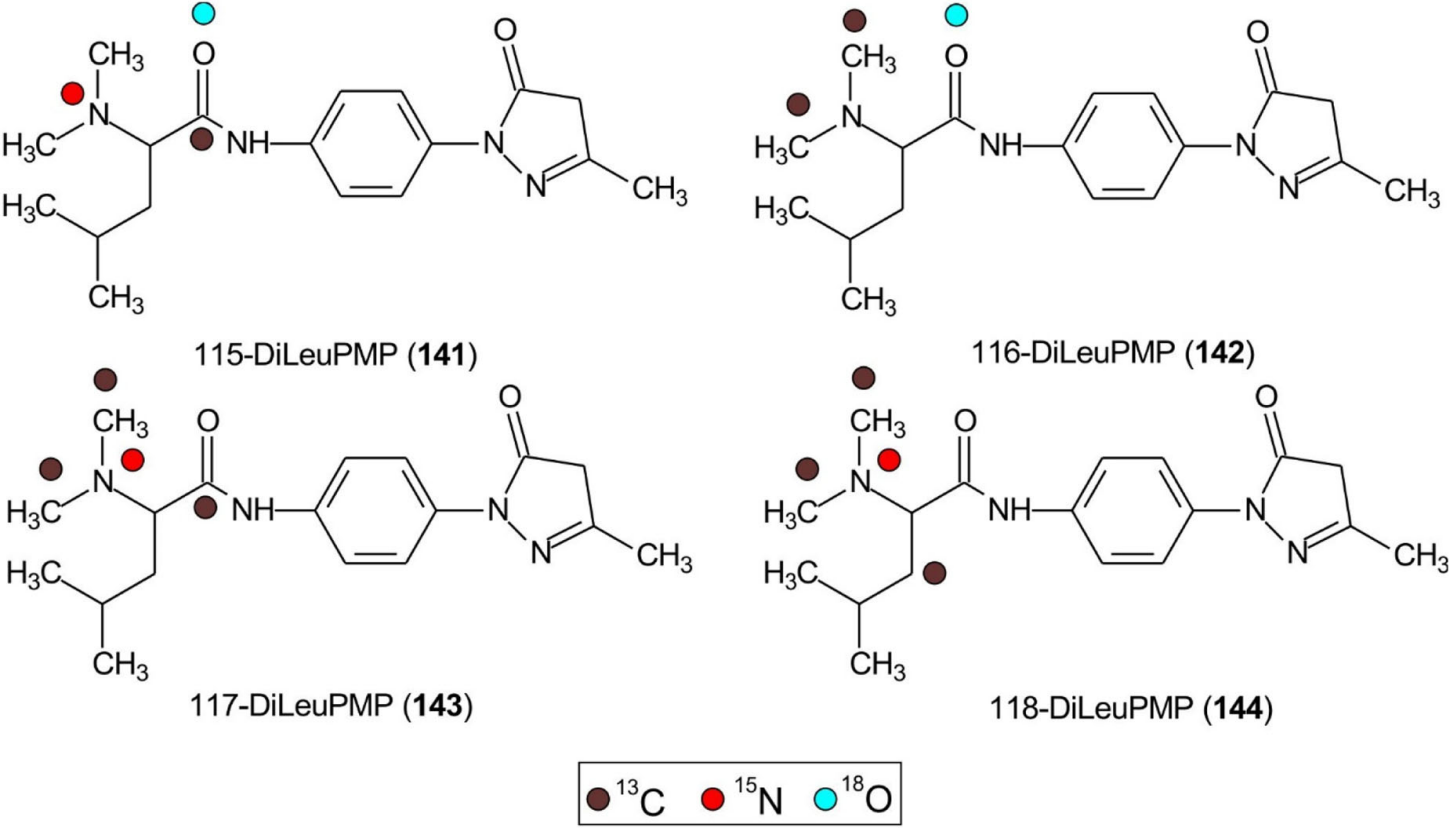
4‐Plex derivatives for *O*‐glycan quantification.

A 4‐plex method for O‐glycan quantification, using the same‐labelled leucine analogues (Scheme 9) as in the glycopeptide method described above, has been used for *O*‐glycan quatification (Li, Gu, et al., 2021). *O*‐glycan release using ammonium hydroxide and labelling to form the modified PMP derivatives (see Scheme 4) was achieved simultaneously by heating at 70^o^C for 24 h. Products were measured by MALDI‐MS or LC‐MS/MS. Applications were to core I *O*‐glycans from human serum.

## 11 FRAGMENTATION

Mechanisms leading to the formation of fragment ions in several carbohydrates continue to be a fruitful area for research with several methods for producing the ions being available. Nomenclature for the fragment ions follows that proposed by Domon and Costello (1988) (Scheme 1).

### 11.1 In‐source decay (ISD)

The [M + H]^+^ ion from α‐CD (as **27** but with six glucose rings) has been shown to exhibit two fragmentation pathways (Jang & Choi, 2021). After ring opening, the first pathway involves successive losses of glucose with the relative abundances of the fragments increasing as their mass decreases. The second series involved losses of OH and glucose units.

The disaccharide isomers gentiobiose (**145**), isomaltose (**146**), melibiose (**147**), lactose (**30**), maltose (**43**), cellobiose (**148**), and sucrose (**42**) have been ionized with a 349‐nm Nd:YLF UV laser from a graphene oxide matrix ([M + Na]^+^ ions) and shown to fragment in a manner that revealed differences between isomers (Lee, Kim, et al., 2021). Anomeric configurations of 1–6 and 1–4 linked isomers could be differentiated by comparing the peak intensity at *m*/*z* 267 with that at *m*/*z* 365. The α‐anomers (maltose [**43**], isomaltose [**146**], and melibiose [**147**] had *m*/*z* 267/*m*/*z* 365 ratios greater than 0.1, while those of the β‐anomers (cellobiose [**148**], lactose [**30**], and gentiobiose [**145**]) had ratios that were less than 0.1. Linkage isomers (1–4 and 1–6) were differentiated by the presence of a peak at *m*/*z* 275, which was only observed with 1–6 linked isomers such as gentiobiose, isomaltose, and melibiose.


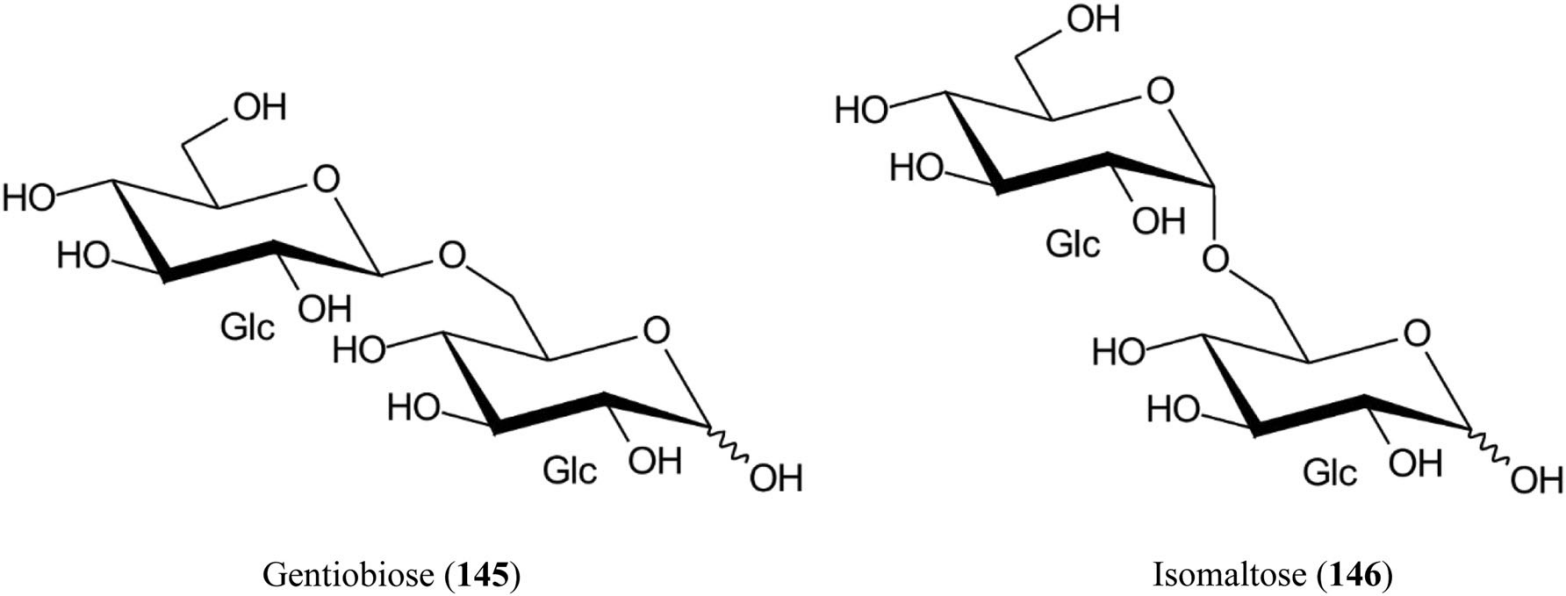



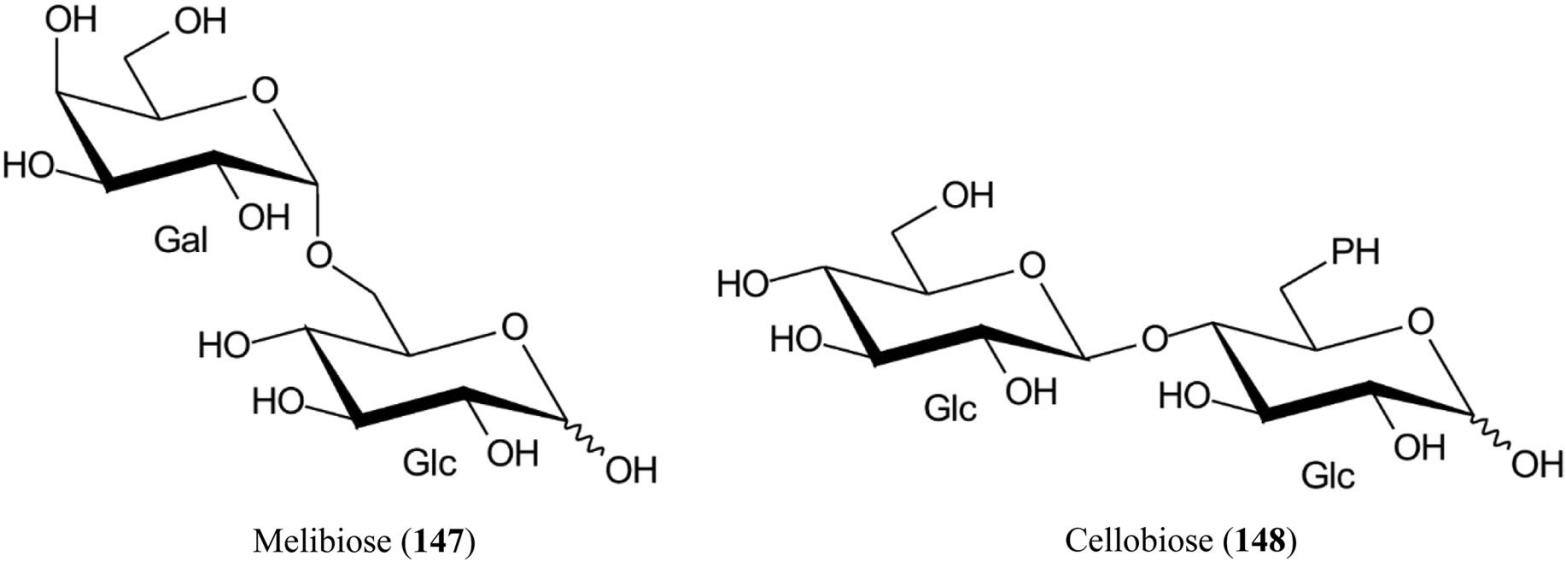


Liew, Chen, and Ni (2022) have studied ISD in electrospray ion sources and noted 0.2%–3% dissociation of neutral glycans and more than 50% dissociation when sialic acid is present. Dissociation rose with increasing temperatures and products of the larger glycans, which were similar to those observed by CID, were smaller glycans, some of which occurred naturally. The authors point out that this property could have adverse effects on the apparent compositions of glycan mixtures.

### 11.2 CID and higher‐energy collisional dissociation (HCD)

Nguan, Tsai, and Ni (2022) have used quantum chemical calculations and experimental measurements to elucidate the fragmentation mechanisms of the [M + Na]^+^ ions from cellobiose and maltose. Four mechanisms were studied. Dehydration mainly occurred through the transfer of a hydrogen atom to O1 of the sugar at the reducing end, followed by a C1−O1 bond cleavage. Cross‐ring dissociation started with a ring‐opening reaction, which occurred through the transfer of a hydrogen atom from O1 to O5 (ring oxygen) of the sugar at the reducing end. The third route, generation of B_1_ and Y_1_ ions, occurred through the transfer of an H atom from O3 (cellobiose) or O2 (maltose) to O1 of the sugar at the nonreducing end, followed by a glycosidic bond cleavage. The fourth pathway, production of C_1_−Z_1_ fragmentation, had two mechanisms: (1) the transfer of a hydrogen atom from O3 or O2 to O4 of the sugar at the reducing end to generate C ions in the ring form and (2) the transfer of a hydrogen atom from O3 of the sugar at the reducing end to O5 of the sugar at the nonreducing end to produce C ions in the linear form.

**Scheme 10:**
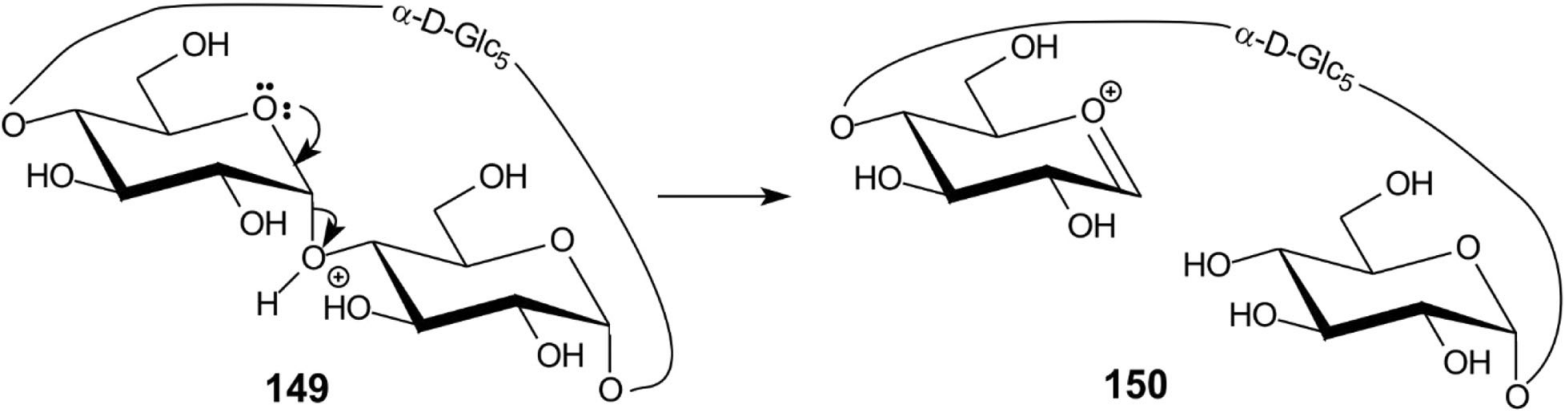
Proposed mechanism for ring opening of cyclodextrin rings.

Fragmentation of protonated β‐cyclodextrins (**27**) by CID and HCD, assisted by fragmentation of di‐ and tri‐methylated CD derivatives has shown initial glycosidic cleavage to open the ring (**150**, Scheme 10) followed by the elimination of glucose subunits and the subsequent release of water and formaldehyde moieties from the glucose monomer and dimer fragment ions (Bruni & Schürch, 2021).

**Scheme 11:**
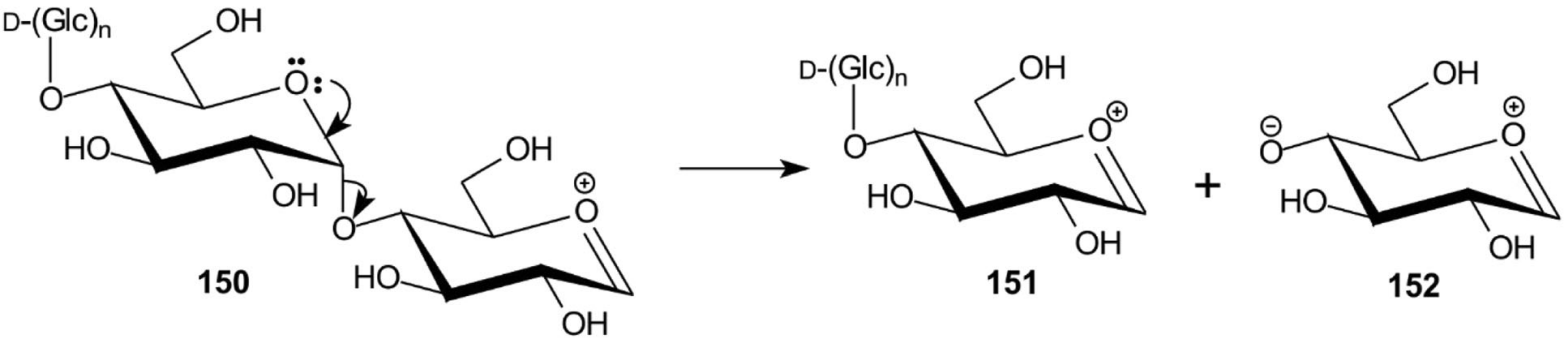
Proposed mechanism leading to elimination of a zwitterionic fragment from cyclodextrins.

**Scheme 12:**
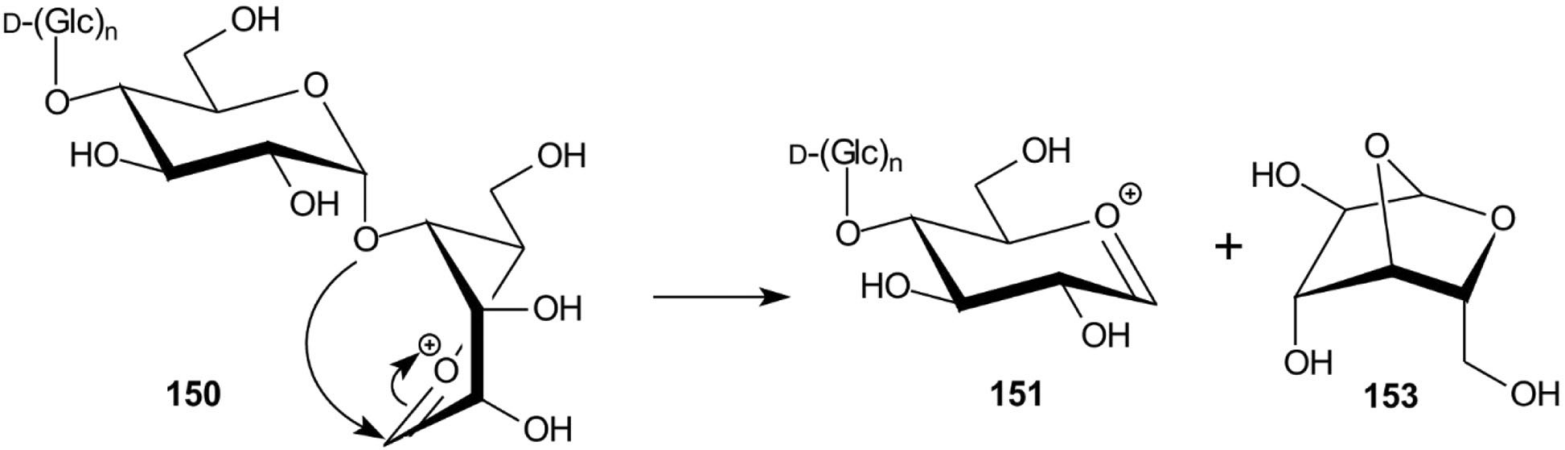
Proposed loss of a 1,4‐anhydroglucose moiety during fragmentation of cyclodextrins.

**Scheme 13:**
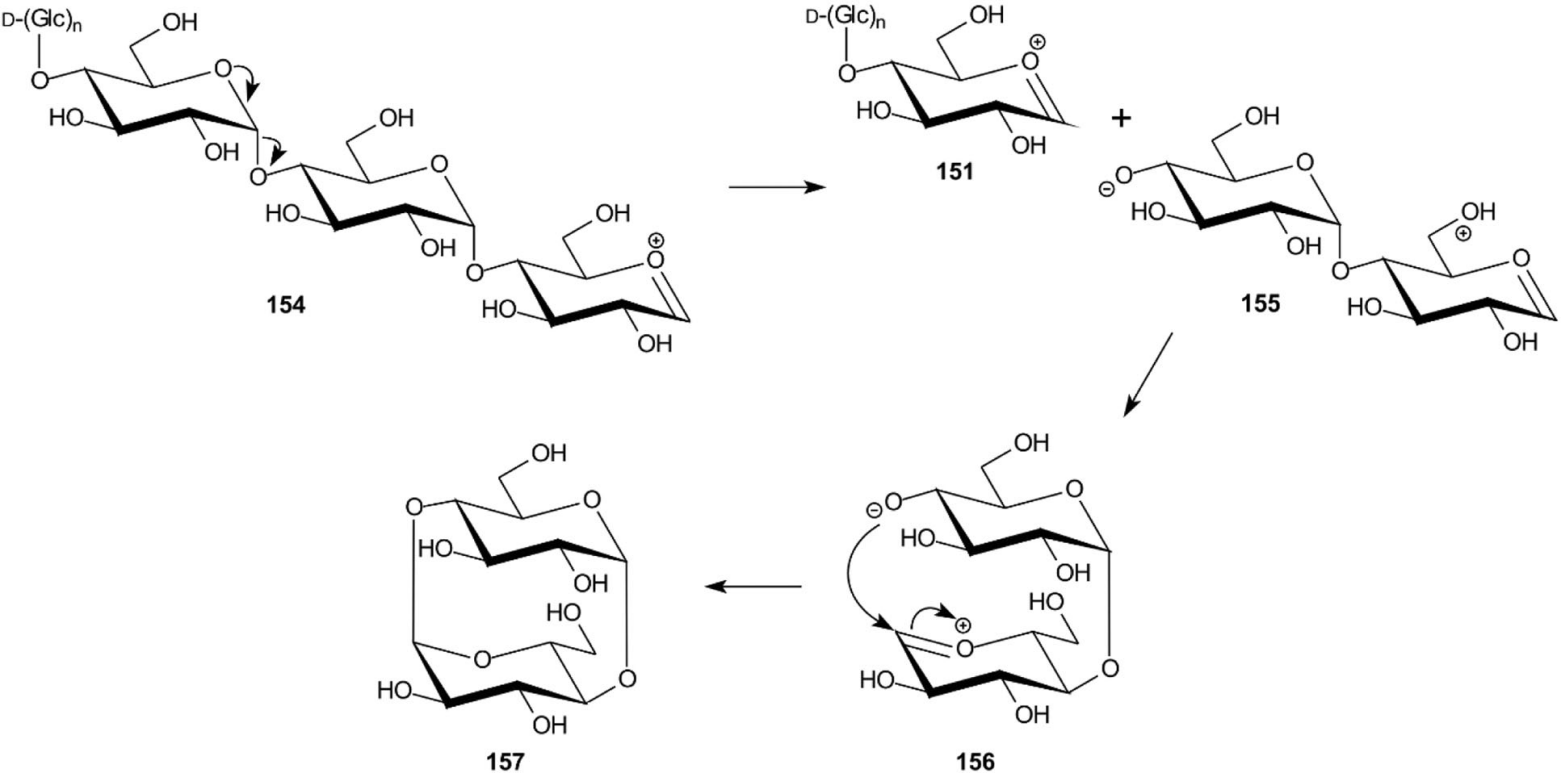
Proposed mechanism for elimination of a new macrocyclic structure during fragmentation of cyclodextrins.

The resulting linear structure further decomposes in charge‐independent processes forming either a zwitterionic fragment from the boat conformation of the reducing‐terminal ring (Scheme 11), elimination of a 1,4‐anhydroglucose moiety (Scheme 12), or loss of a new macrocyclic structure and an oxonium ion (Scheme 13).

**Scheme 14:**
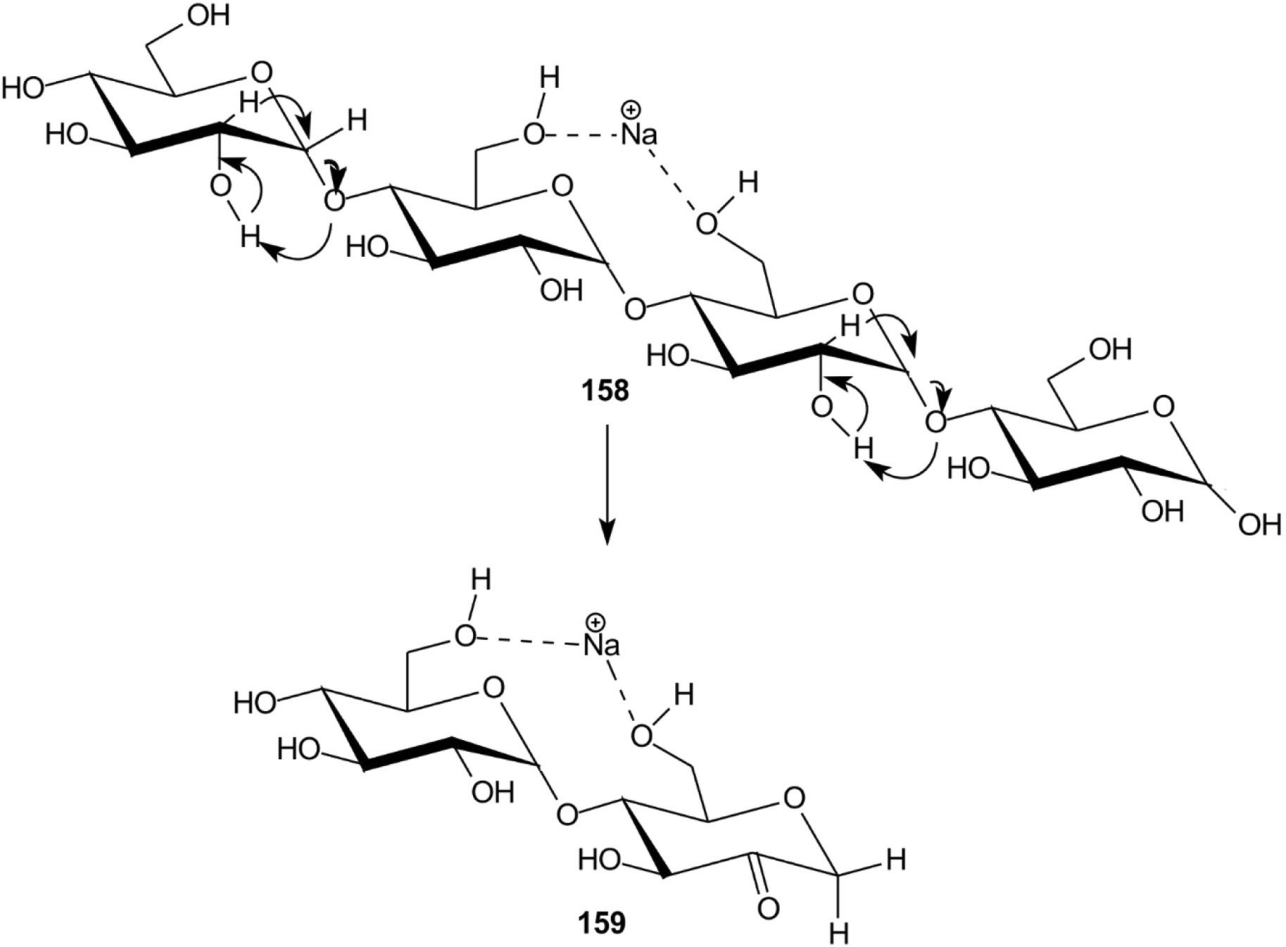
Proposed mechanism for the formation of a fragment with a 2‐ketone group. From Rabus et al. (2021).


Fragmentation of [M + Na]^+^ ions from β‐CD has been studied by Rabus et al. (2021) using ion mobility and cryogenic IR. Electronic structure calculations were consistent with formation of a fragment with a 2‐ketone group as shown in Scheme 14. Other B‐type fragments were formed similarly. The structures of three other proposed fragments (**160**–**162**) are shown below.

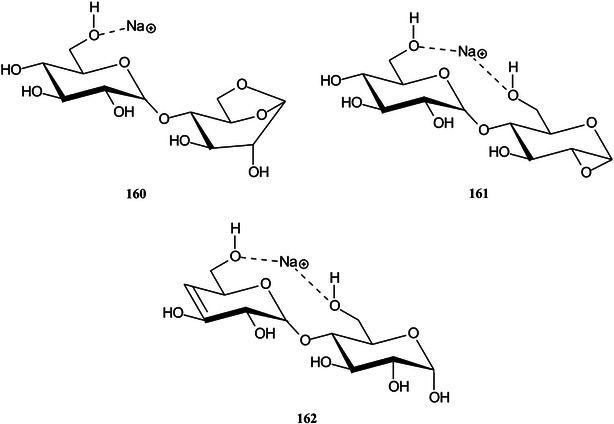


CID of the protonated ion from Lewis A trisaccharide (α‐l‐Fuc‐(1→4)‐[β‐d‐Gal‐(1→3)]‐d‐GlcNAc, **163**) and its methyl glycoside has shown that fragmentation from the reducing end of the ion plays a key role in the fragmentation process. The main product of the fragmentation are Y‐type fragment ions and a combination of Y‐type fragmentation and the loss of water at the reducing end instead of Z‐type fragmentation as proposed earlier. It appears that fragmentation only occurs with the aid of the mobile proton added during ionization (Iwan & Grotemeyer, 2021).


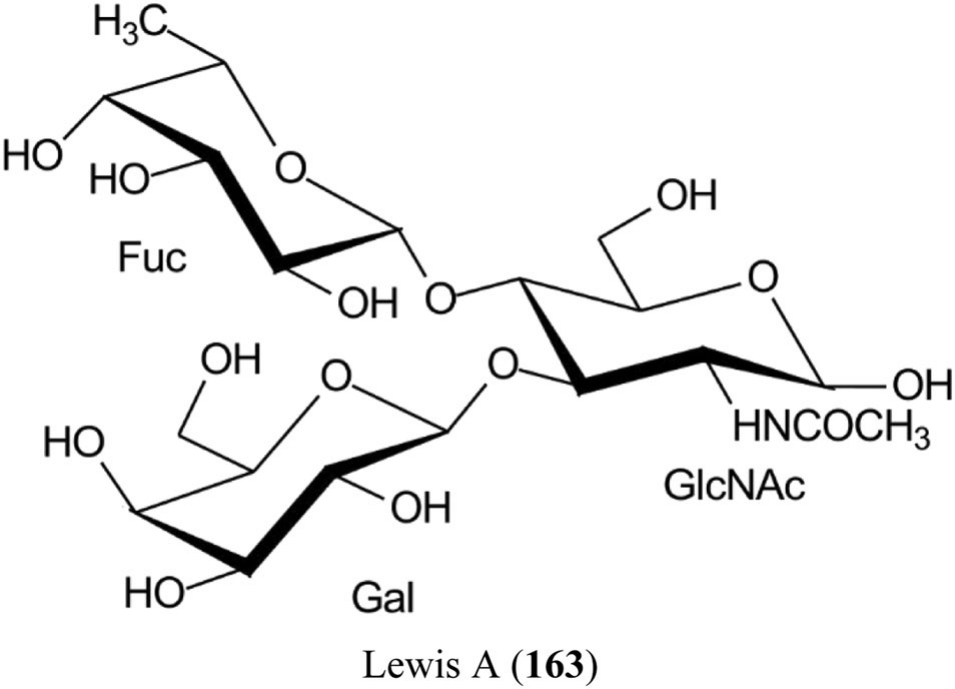


**Scheme 15:**
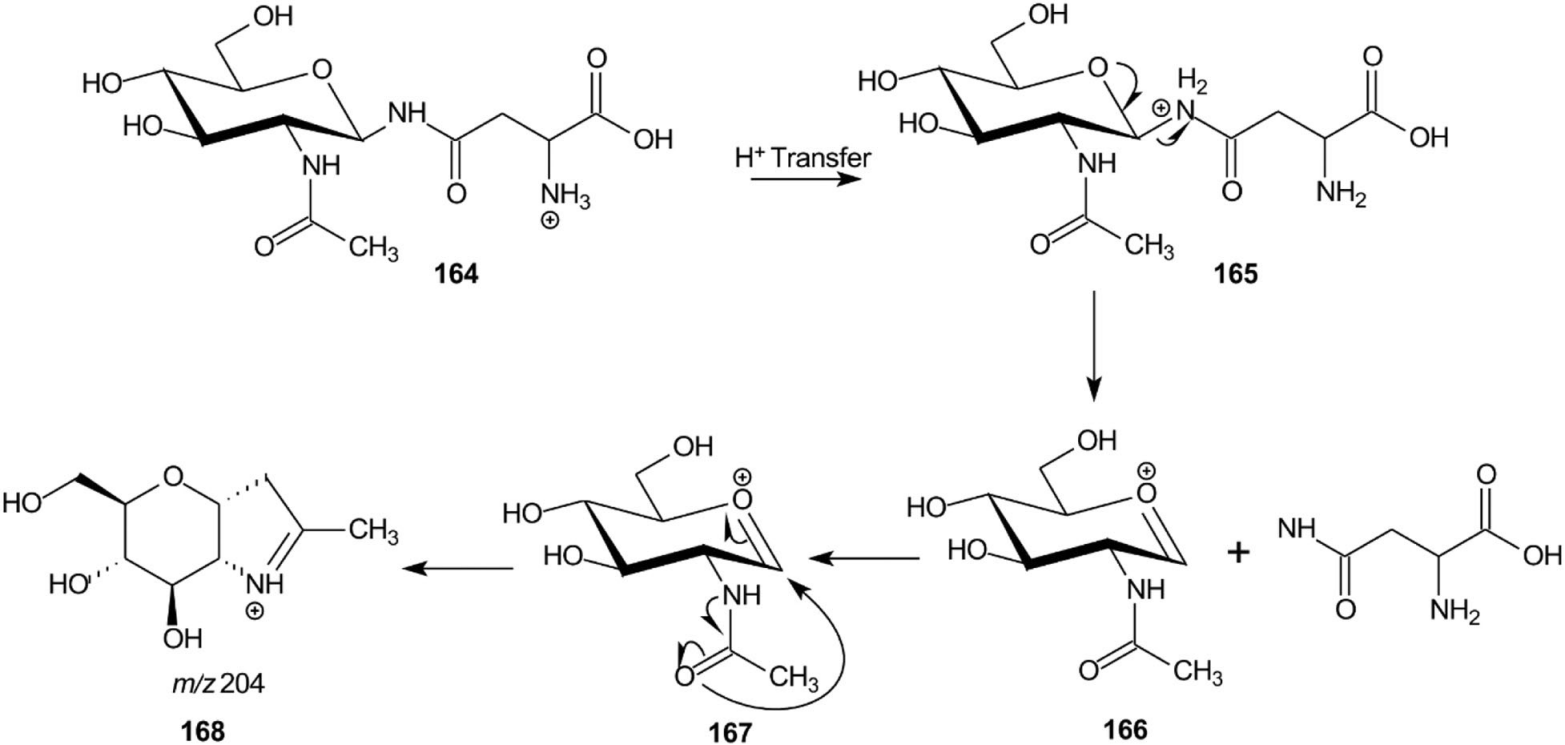
Formation of the ion at *m/z* 204 by the conventional mechanism. Shown for GlcNAc‐β‐1‐Asn + H^+^. From Guan and Bythell (2021).

A major fragmentation pathway for glycopeptides is glycosidic cleavage to give a B fragment with a conventional mechanism from the [M + H]^+^ ion from a monosaccharide giving *m/z* 204 (**168**) as shown in Scheme 15. MS^3^ experiments indicate that this ion fragments further to yield *m/z* 126 by loss of C_2_H_6_O_3_ but there appears to be no reasonable way in which this ion could be produced from the structure of *m/z* 204 shown in Scheme 15.

**Scheme 16:**
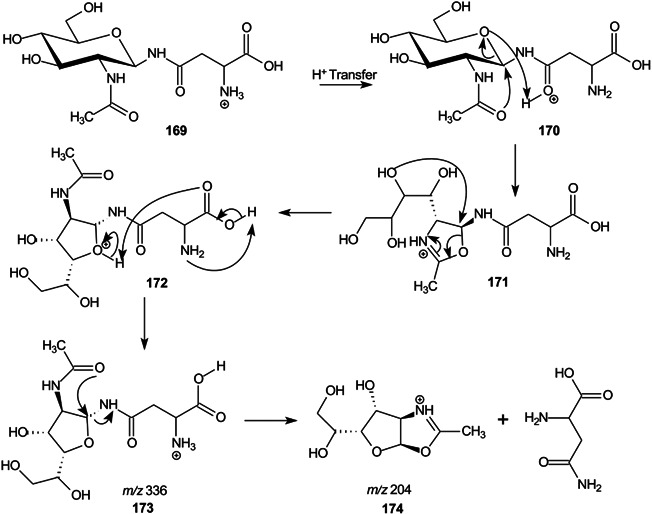
New proposal for formation of the ion at *m/z* 204 (**174**). Shown for GlcNAc‐β‐1‐Asn + H^+^.

Consequently, Guan and Bythell (2021) have investigated this fragmentation mechanism using hydrogen/deuterium exchange and energy calculations and have proposed that the reaction proceeds through a furanose form of the sugar as shown in Scheme 16.

**Scheme 17:**
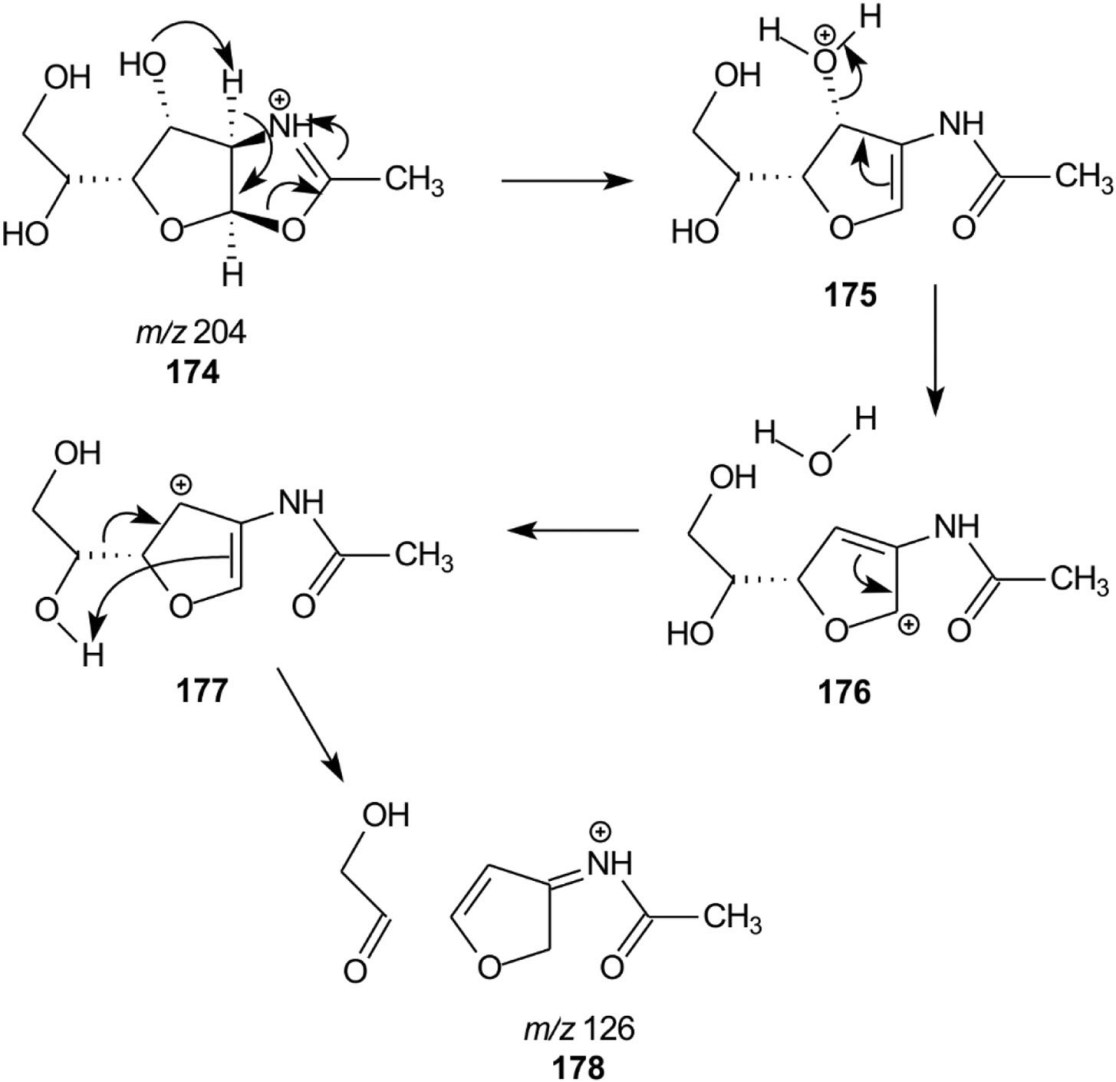
Proposed mechanism for formation of the ion at *m/z* 126 (**178**).

This ion is then proposed to decompose to *m/z* 126 by (**178**) the mechanism shown in Scheme 17.

Evidence supporting this proposed fragmentation mechanism has been supplied by IR action spectrometry (Rabus et al., 2022). This reaction, shown in Scheme 17, was found to occur irrespective of the glycosidic linkage stereochemistry (*α* or *β*) or the *N*‐acetylated hexose (GlcNAc or GalNAc). The authors comment that “Dissociation of the glycosidic and other bonds thus occur from the furanose isomer critically altering the reaction feasibility and product ion structures.”

**Scheme 18:**
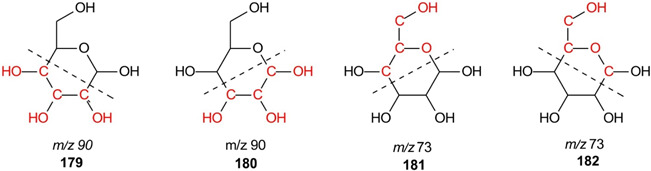
Formation of C3 fragments from d‐glucose. Atoms in the fragments are shown in red. From Rumiantseva et al. (2022).

Rumiantseva et al. (2022) have investigated ^16^O/^18^O and H/D exchange reactions in an attempt to gain more information on fragmentation reactions in general. Oxygen exchange was observed at the anomeric site of d‐glucose with a small amount occurring at the adjacent position as the result of aldose‐ketose reactions. The study showed that several of the cross‐ring fragment ions consisted of several species such as losses of C_3_ and C_4_ fragments from different parts of the molecule as shown for the C_3_ fragments in Scheme 18.

Reasons for the differences in the CID spectra of the two glucose dimers, Glcα1→4‐Glc (maltose, **43**) and Glcα1→6‐Glc (*iso*maltose, **146**) as sodium adducts have been studied using high‐level quantum chemistry calculations (Nguan & Ni, 2022). These calculations revealed that, although the two disaccharides had similar dissociation mechanisms, energy differences between the lowest transition states of various dissociation channels led to different fragmentation patterns. The dissociation barriers for dehydration and glycosidic bond cleavage were similar for the two disaccharides, but the cross‐ring dissociation, which has the lowest dissociation barrier, exhibited differences. The cross‐ring dissociation barrier for *α*‐maltose was only slightly lower than those for dehydration and glycosidic bond cleavage. However, the corresponding barrier for *α*‐*iso*maltose was substantially lower. Furthermore, most of the *α*‐*iso*maltose conformers that led to dehydration also led to cross‐ring dissociation, resulting in suppression of dehydration by cross‐ring dissociation. The findings can explain the low branching ratios for dehydration and glycosidic bond cleavage observed in the CID spectrum of *α*‐*iso*maltose CID spectra.

#### 11.2.1 CID of complexes

Differentiation of isomers has recently been facilitated by formation of complexes formed in the gas phase. For example, Chao and McLuckey (2021) have reacted deprotonated gangliosides with magnesium‐Terpy complex cations ([Mg(Terpy)_2_]^2+^, **183**) to form magnesium complexes and have demonstrated isomeric differentiation between GD1a (**184**) and GD1b (**185**) as [GD1−H+Mg]^+^ ions. In addition, isomeric identification among GT1a, GT1b, and GT1c was also achieved. The method was applied to ganglioside profiling in a porcine brain extract where 34 gangliosides were profiled among only 20 precursor ion *m/z* values.


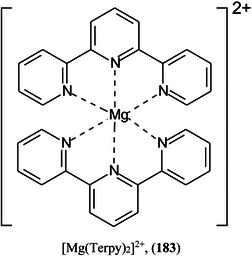



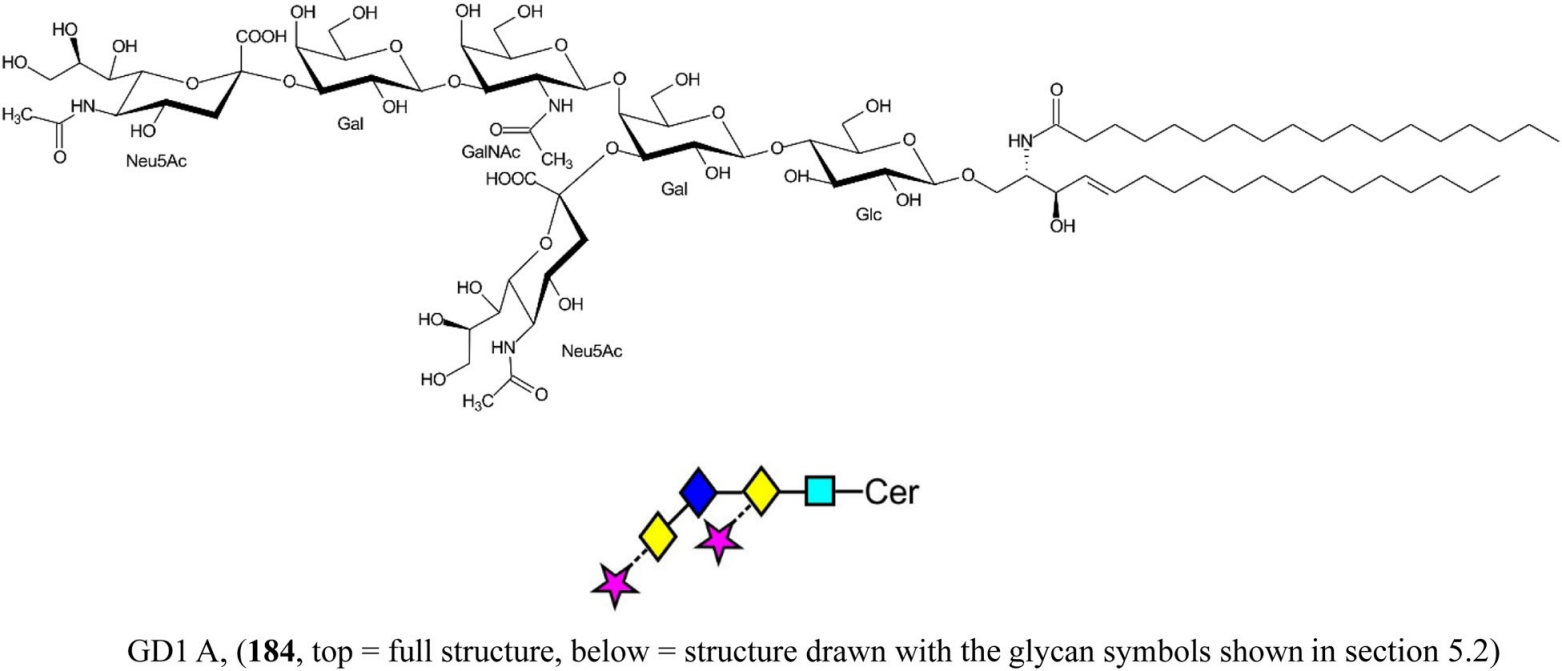



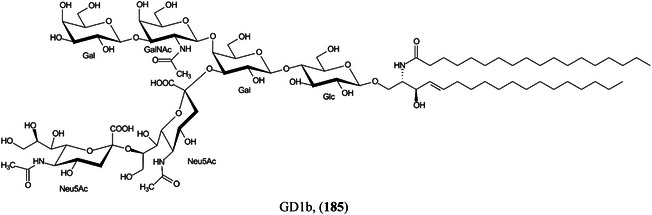


### 11.3 Electron‐transfer/higher‐energy collision dissociation (EThcD)

EThcD, triggered by HCD has been used to map *N*‐glycosylation on intact therapeutic antibodies (Li, Zhu, et al., 2022). The method was reported to provide higher quality spectra than use of EThcD alone and to differentiate between different *N*‐glycan classes such as high‐mannose, hybrid and complex.

### 11.4 Helium‐charge transfer dissociation (He‐CTD)

Using CTD with a modified ion trap instrument, Sasiene, Mendis, et al. (2021) have investigated the effect of Na/H exchange (sodium salt formation) on the fragmentation patterns of mannuronic acid oligomers (**186**). The conclusion was that the fewest possible number of Na/H exchanges will provide the most confident peak assignments and structural characterization.


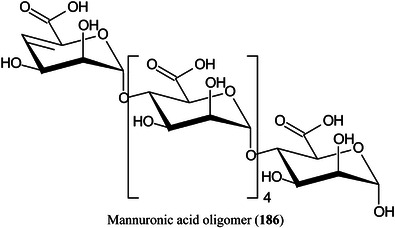


A comparison of the low‐energy CID and He‐CTD spectra for the branched xyloglucan (X_3_G_4_, **187**) has emphasised the superior results that can be obtained by the latter technique. The CID spectra of ions such as [M + Na]^+^ and [M + H + K]^2+^ contained numerous fragments produced by glycosidic cleavages but few cross‐ring cleavage ions and did not allow the 1→4 and 1→6 linkages of the glycan to be identified, He‐CTD, on the other hand, was able to identify the linkage. Different metal adducts (H^+^, Na^+^, K^+^, Ca^2+^, and Mg^2+^) were investigated but were found to have a negligible effect on the type of cross‐ring cleavages that were observed (Sasiene, Ropartz, et al., 2021).


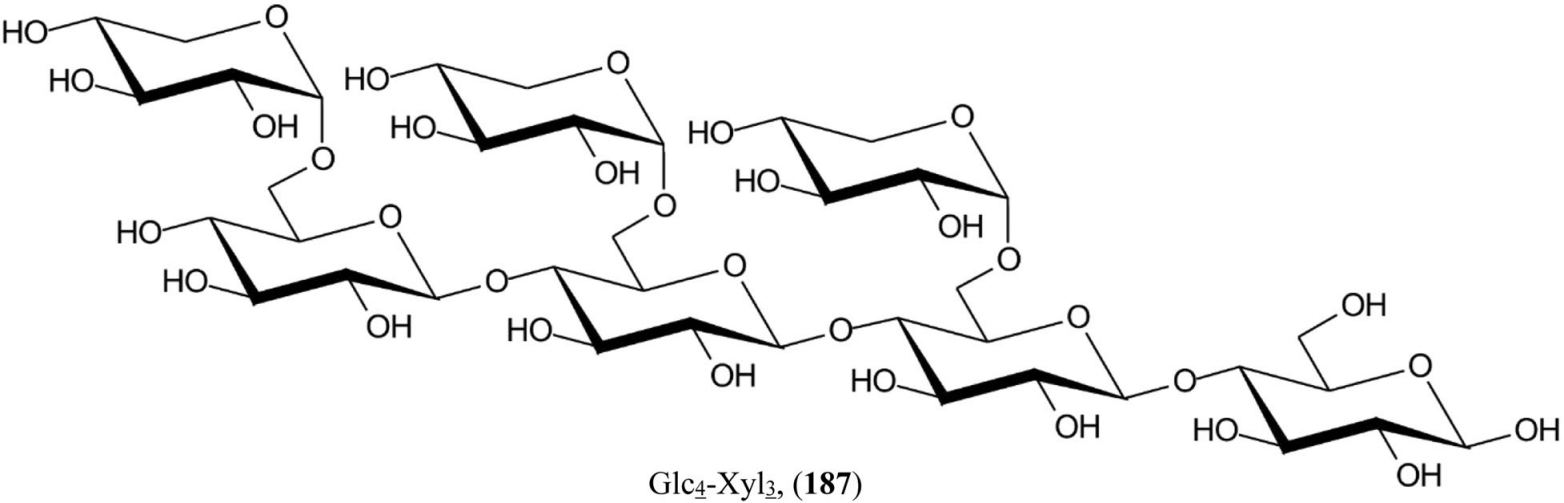


### 11.5 LIFT fragmentation

**Scheme 19:**
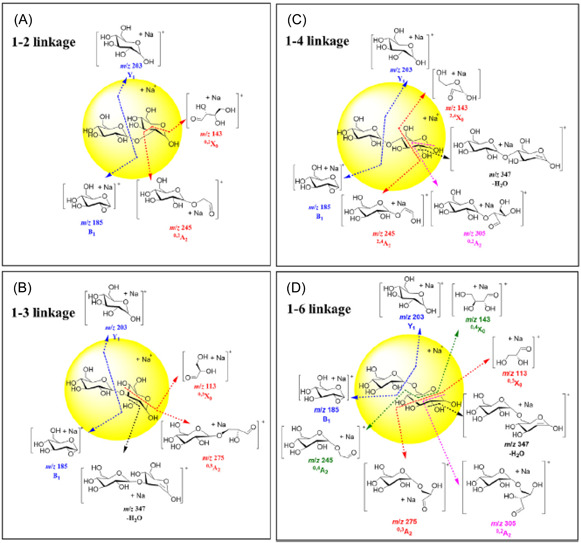
Differentiation of disaccharides with different linkages using LIFT‐MS/MS. From Luo, Zhao, et al. (2022).

Palladium nanoparticles decorated thiol‐functionalized metal organic framework nanocomposite (UiO‐66‐(SH)2@Pd NPs) have been prepared and shown to be an efficient MALDI matrix (Luo, Zhao, et al., 2022). By using this matrix combined with LIFT‐TOF/TOF, 24 oligosaccharide isomers including disaccharides, trisaccharides and tetrasaccharides, have been differentiated as shown in Scheme 19. Reducing and nonreducing disaccharides could be distinguished by the presence or absence of cross‐ring cleavage ions. Only B (*m/z* 185) and Y (*m/z* 203) fragment ions were observed in the MS/MS spectra of nonreducing sugars (trehalose and sucrose), whereas cross‐ring cleavage ions (*m/z* 305, 275, 245,143, and 113) were observed in the spectra of 10 reducing sugars to varying degrees. 1→4 and 1→6 linkage isomers produced *m/z* 305 whereas this ion was missing from the spectra of the 1→2 and 1→3 linkage isomers. The ion at *m/z* 275 appeared in the spectra of 1→6 and 1→3 linkage isomers but not in those from the 1→4 and 1→2 isomers. These and other diagnostic fragment ions are listed in Table 8.

**Table 8:** Diagnostic ions for differentiating linkage isomers from disaccharides (From Luo, Zhao, et al., 2022).

Linkage position	Ions (*m/z*)		Relative ion abundance
	Present	Absent	
1→2	245, 203, 185, 143	305, 275	α>15; β<5 or α>1>β
1→3	275, 203, 185, 113	305	α>1>β
1→4	305, 245, 203, 185	275	α>1>β
1→6	305, 275, 203, 185	‐	α>1>β

### 11.6 Photofragmentation

A method for differentiation of disaccharide isomers using a combination of IR and UV photodissociation mass spectrometry with an FT‐ICR instrument has enabled ten disaccharides, chosen for differences in connectivity, configuration, and/or composition, to be resolved (Du, Zhang, et al., 2022). The disaccharides were complexed with 3,5‐diiodo‐l‐tyrosine (**188**) by ESI to add UV absorption properties and irradiated successively with light from a double‐beam laser. The IR laser produced mainly glycosidic B/Y and C/Z ions, whereas the UV laser produced other complementary fragments. Fragments were formed from both parts of the complexes and were, therefore, not assigned the Domon and Costello nomenclature. Major fragments involved losses of water or monosaccharide residues.


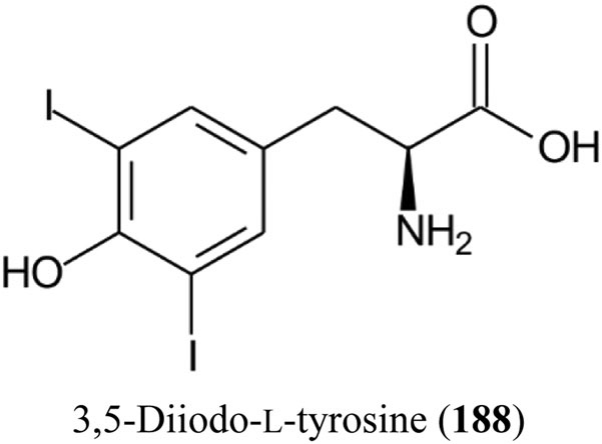


### 11.7 Multiple successive fragmentation (MS^*n*^)

Successive stages of fragmentation presents the analyst with multiple choices of which fragment ion to use for the next stage of fragmentation. This choice can become quite extensive after several stages. To simplify the analysis, Huang, Hsu, et al. (2021) and Ni et al. (2021) have developed a logically derived sequence (LODES) for galactose‐containing tri‐ and tetrasaccharides, but with the potential for it to be extended to other glycans. The method made use of ionic properties that differentiate between, for example, linear and branched compounds and their dissociation to disaccharides, which were compared to a database. The paper contains extensive figures explaining the recommended sequences. However, the success of the method for other glycans depends on the derived disaccharides being available in the database. The method has been extended to determine the structures of *N*‐glycan isomers and the paper contains a useful list of how the relative abundances of cross‐ring fragments relate to linkage (Liew, Yen, et al., 2021). The authors (Liew, Chan, et al., 2021) have also used the method to identify isomeric glycans derived from glycosphingolipids. In an application, Lin and Ni (2022) use the method to determine the structure of lichenin, a linear polymer with alternating β‐Glc‐(1 → 4)‐β‐Glc‐(1 → 4)‐β‐Glc‐(1 → 3)‐Glc, β‐Glc‐(1 → 4)‐β‐Glc‐(1 → 4)‐β‐Glc‐(1 → 4)‐β‐Glc‐(1→ 3)‐Glc repeats. A discussion as to what constitutes “good” (retaining much structural information) and “bad” fragment ions has also been published (Liew, Hsu, et al., 2022). The LODES/MS^*n*^ method also features in a chapter in the reference work “Comprehensive Glycoscience” (Ni et al., 2021).

### 11.8 Electron ionization

A new electron‐activated dissociation (EAD) device has been developed and coupled to a Q‐TOF mass spectrometer (Baba et al., 2021). It features a new electron beam optics design allowing high electron currents up to the space‐charge limit of 0.5 μA in the reaction cell, and enables fast and efficient dissociation of various analytes ranging from singly charged small molecules to multiply protonated proteins. The tuneable electron energy provided access to different fragmentation regimes: electron‐capture dissociation (ECD), hot ECD, and electron‐impact (EI) excitation of ions from organics (EIEIO). The system was evaluated for several compound classes including intact proteins and glycopeptides. Application of hot ECD for the analysis of glycopeptides resulted in rich fragmentation with predominantly peptide backbone fragments; but with additional glycan fragments attributed to the ECD process.

### 11.9 Negative ion fragmentation

Negative ion fragmentation of *N*‐glycans produces more diagnostic ions than positive ion fragmentation (Hykollari et al., 2022) and is frequently conducted using phosphate adducts to stabilize the ions (Harvey, 2020). Ruf et al. (2022) have now shown that adduction with phosphate considerably enhances sensitivity for mono‐ and oligosaccharides and forms more hydrogen bonds with the sugars than Cl^‐^, another popular adduct.

### 11.10 Comparison of methods

Fragmentation spectra of high‐mannose glycans, predominantly Man_5_GlcNAc_2_ (**189**), as [M + Mg]^2+^ and [M + Na_2_]^2+^ ions, induced by CID, ECD or electronic excitation dissociation (EED), have been compared (Wong, Chen, Wu, et al., 2022). CID produced mainly glycosidic cleavages, although more cross‐ring fragments could be obtained at higher intensities when [M + Mg]^2+^ ions were fragmented. ^0,2^A_3_, ^0,3^A_3_, and ^0,4^A_3_ ions (cleavage of the core branching mannose) provided structural information on the 3 → 1 and 6 → 1 linkages of the mannoses. Some internal fragment ions, such as ^2,4^A_5_/Y_3_β, were also produced in high abundance. ECD produced fewer fragments compared to the other dissociation methods when either of the metal ions were used as charge carriers. Cross‐ring fragments were produced in relatively high abundance, with the charge mainly retained on the nonreducing end. EED produced extensive glycosidic and cross‐ring cleavages with either metal charge carrier. More structural‐specific fragments were produced when Na^+^ was used as the charge carrier and this metal also provided higher fragmentation efficiency. Of the 31 possible cross‐ring cleavages, 25 were found, thus providing extensive linkage information. Many fragment ions were produced by all three dissociation methods when Mg^2+^ was used as the charge carrier. Best results were obtained with CID of [M + Mg]^2+^ ions and EED of sodiated glycans.


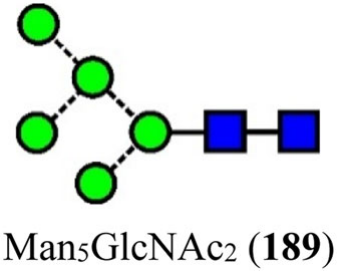


These three dissociation methods have also been compared for structural characterization of doubly charged *N*‐glycopeptides. CID produced distinctively different positive ion mass spectra for glycopeptides adducted with different charge carriers (hydrogen, sodium, magnesium). Protonated species produced mainly glycosidic cleavages in high abundance. Glycopeptides adducted with magnesium formed more cross‐ring cleavages, whereas doubly sodiated species produced cleavages at both glycan and peptide moieties. The effect of charge carriers on fragmentation in ECD and EED was lower than that in CID. ECD produced mainly peptide backbone cleavages but few cleavages of the glycan, whereas EED of glycopeptides resulted in extensive fragmentation regardless of the charge carrier. However, magnesiated species gave more cross‐ring cleavages than other charge carriers (Wong, Chen, Zhang, et al., 2022).

## 12 COMPUTER ANALYSIS OF SPECTRA

Several reviews on computer applications are listed in Table 9.

**Table 9:** Reviews and general articles on computer analysis of spectra.

Subject	Contents	Citations	References
Glycoinformatics in the artificial intelligence era	General review, discusses early failures, describes how the field has benefitted from lessons learned from areas such as proteomics, and makes predictions on the future	164	Bojar and Lisacek (2022)
Recent advances in software tools for more generic and precise intact glycopeptide analysis	Includes table describing different software	108	Cao, Liu, et al. (2021)
Glycobioinformatics in deciphering the mammalian glycocode: Recent advances	Comprehensive list of software tools	264	Datta and Sukhija (2021)
Glycobioinformatics	Short overview. Discussion of various systems	64	Gerwig (2021a)
Artificial intelligence in the analysis of glycosylation data	Use to gain mechanistic insights into glycosylation machinery and to predict models of glycosylation	78	Li, Chiang, et al. (2022)
Book chapter ‐ Analytical software and databases in *N*‐glycoproteomics	Mainly glycopeptide identification. States that latest GPSeeker reports additional structure‐specific information of monosaccharide sequences unavailable from older systems such as Byonic and GPQuest. Additionally, search engines that support *O*‐glycosylation identification are also briefly introduced.	74	Qin and Tian (2022)

A large amount of work has been devoted to designing software for analysing glycan spectra with the impression that lack of software is a serious problem for glycomics. Such attitudes must be viewed with caution because most of the software only gives pointers as to what a correct structure should be. Simply matching masses to structures in a database clearly is only a first step and obviously cannot assign a structure if it is not represented in the database and, of course, this method is incapable of identifying new structures. Also, taking a structure with the best fit out of several possibilities as the correct structure, cannot be accepted as good science; such putative structures must be confirmed with orthogonal techniques. In any case, a skilled analyst could probably identify a compound simply by looking at the spectrum and, in addition, spot the presence of additional compounds; something a software package would almost certainly miss.

Efforts to integrate various software packages for glycoinformatics have been summarised with emphasis on using consistent nomenclature across the various packages (Mariethoz et al., 2022) and various software packages have been compared with the identification of key variables that should guide future software developments and assist informatics decision‐making in glycoproteomics (Kawahara et al., 2021).

Several methods have been developed for converting glycan structures into computer‐readable formats. The major ones, International Union of Pure and Applied Chemistry (IUPAC), Linear Notation for Unique Description of Carbohydrate Sequences (LINUCS), Kyoto Encyclopedia of Genes and Genomes (KEGG), Chemical Function (KCF), GLYcan Data Exchange‐II (GLYDE‐II) and Glyco Connection Table (GlycoCT) have been summarised in a short review (Frey, 2022).

A glycosylation mapping tool, termed GlycoMaple, which visualizes and estimates glycan structures based on gene expression has been developed (Huang, Aoki, et al., 2021). Nine hundred and fifty genes involved in glycosylation and its regulation were selected and the expression profiles of these genes were mapped onto global glycan metabolic pathways to predict glycan structures. These structures were confirmed using glycomic analyses of *N*‐glycans in 40 knockout HEK293 cell lines. In addition, the glycan structures of 64 cell lines, 37 tissues, and primary colon tumor tissues were estimated and compared using publicly available databases. The authors point out that this is only a predictive tool for possible structures and that the structures of detected compounds must be confirmed by orthogonal methods. The code for GlycoMaple is available at https://glycosmos.org/glycomaples/index.

Zhou and Neelamegham (2022) have describes the development and usage of a package entitled “comparative Glycomics” (cGlyco) which is an open‐source program that can be used to compare data from multiple mass spectrometry runs. It has been used, for example, to compare differences in the metabolism of various human blood cell types and it may also be applied to different tissue types or to data collected from other mass spectrometers in the field.

There is currently much interest in increasing glycan data in bioinformatics databases such as ChEBI and PubChem, and connecting them to resources at the EMBL‐EBI and NCBI. Much material is available in databases such as GlyTouCan which contains glycans obtained primarily through batch upload from glycan repositories and individual laboratories and, as such, many glycan structures from such sources may not be fully defined. Databases like ChEBI and PubChem were designed to accommodate complete atomistic structures with well‐defined chemical linkages and, consequently, they cannot easily accommodate the structural ambiguity inherent in glycan databases. Therefore, there is a need to improve the organization of glycan data to enhance connectivity across the major NCBI, EMBL‐EBI and glycoscience databases. Navelkar et al. (2021) have developed a workflow in collaboration between GlyGen, ChEBI and PubChem to improve the connectivity of glycan data across these resources. GlyGen hosts a subset of glycans (∼29,000) from the GlyTouCan database and has submitted valuable glycan annotations to the PubChem database and integrated over 10,500 (including ambiguously defined) glycans into the ChEBI database. The current PubChem, ChEBI and GlyTouCan mappings can be downloaded from GlyGen (https://data.glygen.org).

### 12.1 Algorithms for analysing spectra

An addition to the Byonic software (Bern et al., 2012) that addresses the issues of finding glycopeptide spectra when they are a tiny fraction of the total spectra; assigning spectra with unanticipated glycans that are not in the initial glycan database; and finding, scoring, and labeling diagnostic peaks in tandem mass spectra has been developed (Roushan et al., 2021).

Claimed to be better than Byonic, the freely available package pGlyco3 (available at https://doi.org/10.1038/s41592-021-01306-0) is a “glycan‐first” application for analysing glycopeptides. It combines electron‐based (HCD, EDC) dissociation and provides site‐specific glycan localization. It is claimed to be 5‐40 times faster than other glycoproteomics search engines (Zeng, Cao, et al., 2021).

StrucGP is a new package for determination of *N*‐glycan structures from glycopeptides (Shen et al., 2021). It categorizes B and Y ions, produced by low‐energy MS/MS into three groups, core fragments, glycan subtype fragments and fragments from the antennae. Based on only these masses and LC data, it is claimed that complete structures can be determined. The method was tested satisfactorily with standard glycoproteins such as ovalbumin, fetuin, RNase B and IgG and then applied to mouse brain and other tissues. The detailed structures of 600 glycans were reported from mouse brain and 719 from five tissues. In another paper using StrucGP, 773 *N*‐glycans were identified in human seminal plasma (Xin, Xu, et al., 2022). Unfortunately, many of these structures do not conform to any reported biosynthetic pathways and the authors’ report that “It is worth to mention that the location of each branch structure (such as α−2,3 or α−2,6 mannose) can't actually be identified by StrucGP”. Thus, many of the reported structures are undoubtedly incorrect raising the question of how much reliance should be placed on the supposed identification using only computer algorithms such as this. It would appear that most “structures” can only be regarded as pointers to the correct structure and that these structures must be confirmed by orthogonal techniques as pointed out above. This conclusion is supported by statements in several other publications using computer algorithms such as “the structure with the highest score was taken to be correct.”, or the highest score within a given data set (Zhang, Peng, et al., 2022). Unfortunately, several publications have appeared in which StrucGP (Li, Zhao, et al., 2022; Xin, Xu, et al., 2022) and other computer software are the only methods used to “identify” *N*‐glycans with again, dubious structures being reported in many instances. Authors and reviewers of these papers must ensure that, at least, reported structures conform to the products of established biosynthetic pathways in the species being investigated. Thus, in publications where “identifications” have been made only by the use of computer algorithms and database matching with no follow‐up, results should be treated with caution. It is somewhat gratifying to see that at least one recent paper that used StrucGP to “identify” structures (of the Covid‐19 virus spike glycoprotein), that the authors only report the partial structures that the algorithm can correctly identify (Zhu, Chen, et al., 2022).

A software package called GlycanAnalyser has been developed for automatically interpreting the results of methods of glycan analysis that rely on HPLC separation of glycans and exoglycosidase digestion to determine the nonreducing terminal monosaccharide constituents. The paper describes a protocol for using the software and a table listing other software tools available to the glycobiologist (Walsh et al., 2022). The HPLC/exoglycosidase technique originated in Oxford in the 1990s and was developed in Dublin with the introduction of GlycoBase, a database containing retention data for many *N*‐linked glycans. Glycostore is a development of this work (Campbell et al., 2021). Its first release in October 2017) contained over 850 glycan entries accompanied by over 8500 retention times including data from HPLC, PGC interfaced with ESI‐MS/MS, and CE.

GAGrank is an algorithm that uses a bipartite graph model for sequencing GAGs from electron detachment dissociation (EDD) or negative electron transfer dissociation (NETD) tandem mass spectra. The process involves first assigning GAG product ions using the recently‐developed GAGfinder algorithm (Hogan et al., 2018) and secondly calculating every possible sequence for a given GAG composition. Sequences are given a higher ranking if they link to many important fragments. The system was optimized using ten training sequences and validated with three validation sequences. It was able to sequence isomeric mixtures using two mixtures at five different ratios (Hogan et al., 2021).

An algorithm for reconstruction of glycan structures, GlycoDeNovo, first reported in 2017 (Hong et al., 2017), has been upgraded to GlycoDeNovo2 with the inclusion of the calculation of glycan composition from precursor mass and the ability to calculate a p‐value from the predicted structures (Chen, Wei, et al., 2022).

One of the weaknesses of mass spectrometry for glycan analysis is the problem of identifying the nature of the constituent monosaccharides. An algorithm, termed HexNAcQuest has now been developed and is claimed to be able to differentiate GalNAc from GlcNAc with 97% accuracy (Li, Hou, et al., 2022). Essentially, the algorithm looks at the relative intensities of five fragment ions (HCD mode, Orbitrap) from oxonium ions derived from glycopeptides. Specifically, if the intensity of *m*/*z* 138 is much higher than that of *m*/*z* 144, the probability of GlcNAc is between 0.5 and 1, and if the ions at *m*/*z* 138 and 144 are of similar intensity, the corresponding probability will be between 0 and 0.5, indicating an *O*‐GalNAc modification.

The Toolbox Accelerating Glycomics (TAG) package for analysing MALDI spectra, was developed in 2020 (Miura et al., 2020) and consists of three units, “TAG List” which creates a glycan list that is used for database searching in “TAG Expression”; ‘TAG Expression’, which automatically annotates and quantifies glycan signals; and ‘TAG Pathway’ which maps the obtained expression information to biosynthetic pathways. This software has now been updated (Miura et al., 2022) to include some less common glycans such as those containing glucuronic acid and the linkage‐specific alkylamidation method (SALSA) (Hanamatsu et al., 2018, 2019; Nishikaze et al., 2017) for determining the linkage of sialic acids.

The web application “Glycoanalysis by the Three Axes of MS and Chromatography” (GALAXY) is a tool for assisting glycoprofiling by HPLC and MS data of 2‐AP‐derivatized glycans (Kato & Takahashi, 2009; Takahashi & Kato, 2003). A new version (3) has now appeared and includes new HPLC data on glucoronylated and sulfated glycans and an improved graphical user interface (Yagi et al., 2022).

### 12.2 Quantification

The “Individuality Normalization when Labeling with Isotopic Glycan Hydrazide Tags” (INLIGHT) strategy for glycan quantification uses hydrazide chemistry to derivatize the reducing end of *N*‐linked glycans and incorporates either a natural (NAT, ^12^C_6_) or a stable‐isotope label (SIL, ^13^C_6_) to enable relative quantification. GlycoHunter is software created in MATLAB that enables researchers to process quantitative glycomics data generated with but not limited to, INLIGHT. GlycoHunter accepts the commonly used data file formats imzML and mzXML and effectively identifies all peak pairs associated with NAT and SIL‐labeled *N*‐linked glycans using MS^1^ data. It also includes the ability to export the results for further analysis using Skyline or Excel. The software is available for no charge from the Web site https://glycohunter.wordpress.ncsu.edu/ (Kalmar et al., 2021). Ion mobility properties of INLIGHT derivatives of several *N*‐glycans released from commercial glycoproteins such as horseradish peroxidase (HRP) and fetuin have also been reported (Butler, Kalmar, et al., 2022).

gQuant, coded in Python, is another program for processing quantitative data from experiments using stable isotopes (Huang, Jiang, et al., 2021). In tests, reported quantitation ratios matched well with the experimental glycan mixture ratios ranging from 1:10 to 10:1. The application has a simple user interface and can easily be adapted by users for specific experimental designs, such as specific glycan databases or different derivatization types.

### 12.3 Databases

Five‐ reviews are relevant and are listed in Table 10.

**Table 10:** Reviews on glycan databases.

Subject	Contents	Citations	References
Analytical software and databases in *N*‐glycoproteomics	General overview of current analytical software and databases in *N*‐glycoproteomics	‐	Qin and Tian (2022)
Plant lipid databases	Overview of plant lipid databases focusing on nomenclature, structures as well as physical and chemical properties	18	Dörmann (2021)
Database search assisted *N*‐glycan structure identification	Concentrates on conventional glucose unit calculation, the virtual ladder approach and exoglycosidase glycan sequencing	49	Jarvas et al. (2021)
Databases and bioinformatic tools for glycobiology and glycoproteomics	Comprehensive review of different databases with comments on each	117	Li et al. (2020)
Glycosciences. De: Databases and tools to support research in glycomics and glycoproteomics	Overview of the individual databases and applications within Glycosciences. de, and their interconnections with each other and with external resources	40	Lütteke (2021)

GlycoPOST is a database that accepts MS data from glycomics experiments and issues an accession number to provide traceability for reuse and reanalysis of the data. This system is based on the jPOST repository system (Okuda et al., 2017), a stable MS data repository for proteomics. The GlycoPOST system has been designed to make it easy to input various metadata such as experimental conditions and instrument settings (ion source, ion transfer optics, etc.) specific to glycomics. GlycoPOST is a part of the GlyCosmos portal (Yamada et al., 2020), which also includes UniCarb‐DR and GlyTouCan (Aoki‐Kinoshita et al., 2016) as associated repository systems. Because of this relationship between UniCarb‐DR and GlycoPOST, the authors have implemented a combined user registration system that handles user information for both repositories (Watanabe et al., 2021). Metadata should comply with the MIRAGE guidelines (York et al., 2014). Several of these guidelines, covering techniques such as mass spectrometry (Kolarich et al., 2013) and sample preparation (Struwe et al., 2016) have been published. The latest covers capillary electrophoresis (Lageveen‐Kammeijer, Rapp, et al., 2022).

The Carbohydrate Structure Database (CSDB), which has been in place for some 15 years, aims to incorporate the best features of other databases while avoiding their problems. A recent paper (Toukach & Shirkovskaya, 2022) summarizes other databases and outlines the main features of CSDB. The project features free access, annual data deposition and updates, search and correction of errors (including those in publications), and regular announcement of new services.

### 12.4 Tools for displaying structures

A useful discussion of various software tools for annotating and displaying glycan structures has been published in the book “Glycosylation,” part of the *Methods in Molecular Biology* series (Mariethoz et al., 2022). Unfortunately, there is heavy emphasis on the SNFG method (Varki et al., 2015) for drawing structures which, in this reviewer's opinion, is inferior to the so called “Oxford” system used in this review (Harvey et al., 2009b); which is more logical, is equally useful in black and white, and allows the reader to easily depict the structure of many newly discovered monosaccharide constituents without having to invent new symbols. This is because the Oxford system uses different shapes, not color, for the basic monosaccharides and shows modifications to these structures by additions such as a full fill for the presence of an *N*‐acetyl group, and the addition of one dot or two to show the presence of one (as in fucose) or two absent hydroxyl groups respectively. An additional advantage is that many new symbols drawn in this way can simply be “read” directly without the reader having to refer to a table of symbols, many of which do not follow a logical pattern. Linkage in the Oxford system is shown by the angle of the lines connecting the symbols. Although an advantage to the original SNFG system, which relied on the linkage being written on the bond, the Oxford linkage system is now recommended for use by the SNFG system. The colors used by the SNFG system for the monosaccharides have been incorporated into the Oxford system to make it more understandable for those not familiar with it.

### 12.5 Tools for annotating and displaying spectra

The software package Sweet‐SEQer (Serang et al., 2013) for annotation of tandem mass spectra, written in Python, has been modified and improved using C++. The new version, C‐SEQer, produces the same output but is claimed to do so in approximately 15‐fold less time than the Python version (Burgoyne & Smith, 2021).

A software package, termed Glycopeptide Abundance Distribution Spectra (GADS) has been developed for simplifying the visualization of glycopeptides for specific peptides (Remoroza et al., 2021). The presentation is in the form of a mass spectrum with glycopeptide peaks labelled with their glycan composition. The displayed mass (in place of *m/z*) of each peak is that of the glycan mass, and its abundance corresponds to its relative abundance in the electrospray MS^1^ spectrum. The method is illustrated with glycopeptides from several glycoproteins, including SARS‐CoV‐t spike protein. The software has been applied to human milk proteins (Remoroza et al., 2022) where two varieties of mass spectral libraries were generated. One contains GADS spectra, whereas the other contains tandem mass spectra of the underlying glycopeptides.

Mass Spectrum Peptide Annotation (MS_Piano) is software developed for annotation of peaks in CID spectra of peptides or *N*‐glycopeptides for given peptide sequences, charge states, and positional modifications. The program annotates each peak in high or low resolution spectra with possible product ion(s) and the mass difference between the measured and theoretical *m/z* values. Spectral quality is measured by two major parameters: the ratio between the sum of unannotated versus all peak intensities in the top 20 peaks, and the intensity of the highest unannotated peak. The software is freely available in .exe and .dll formats for the Windows operating system (Yang, Neta, et al., 2021).

To address the absence of a high‐throughput tool for visualization and molecular annotation of *N*‐glycans in MSI data, Veličković et al. (2021) have developed NGlycDB, a public database of *N*‐glycans based on METASPACE, an open‐source cloud engine for molecular annotation of MSI data to automatically annotate, visualize, analyze, and interpret high resolution mass spectrometry‐based spatial *N*‐glycomics data. Its applicability was demonstrated by analyzing MALDI‐MSI data from FFPE human kidney and murine lung tissue sections.

## 13 STUDIES ON SPECIFIC CARBOHYDRATE TYPES

### 13.1 Polysaccharides

Reviews and general articles are listed in Table 11.

**Table 11:** reviews on the use of matrix‐assisted laser desorption/ionization for the analysis of polysaccharides.

Subject	Notes	Citations	References
A comprehensive review on mutan (a mixed linkage of α‐1‐3 and α‐1‐6 glucans) from bacterial sources	General review (history, transferases, biosynthesis, analysis (little on MALDI), function)	139	Boddapati and Gummadi (2021)
Agar oligosaccharides: A review of preparation, structures, bioactivities and application	General review, brief MALDI references. Mainly biological activity	60	Chen, Fu, et al. (2021)
Monosaccharide composition analysis	Mainly GC/MS with protocols. Brief mention of MALDI	26	Gerwig (2021d)
Carrageenan oligosaccharides: A comprehensive review of preparation, isolation, purification, structure, biological activities and applications	Production of oligosaccharides, separation methods, structural identification (MALDI, NMR), biological activity	186	Guo, Wei, et al. (2021)
Recent advances in qualitative and quantitative analysis of polysaccharides in natural medicines: A critical review	Emphasis given to depolymerisation of polysaccharides to oligosaccharides and their subsequent analysis	100	Li, Zhang, Han, et al. (2022)
Enzymatic synthesis and characterization of different families of chitooligosaccharides and their bioactive properties	Includes use of MALDI for characterization of chitooligosaccharides	87	Miguez et al. (2021)
Date (*Phoenix dactylifera* L.) polysaccharides: A review on chemical structure and nutritional properties	Structure, extraction, identification and biological effects	89	Noorbakhsh and Khorasgani (2022)
The cell wall of hornworts and liverworts: Innovations in early land plant evolution?	Presents an overview on shared and divergent polysaccharide features between these two groups of bryophytes and vascular plants.	175	Pfeifer et al. (2022)
Exploiting the amazing diversity of natural source‐derived polysaccharides: Modern procedures of isolation, engineering, and optimization of antiviral activities	Concentrates on sulfated polysaccharides. Comparison of extraction techniques. Few references to MALDI	230	Ray et al. (2021)
Plants arabinogalactans: From structures to physico‐chemical and biological properties	Structure, occurrence in different plant parts, extraction, purification, properties. Some discussion of MALDI	231	Saeidy et al. (2021)
Alginate derived functional oligosaccharides: Recent developments, barriers, and future outlooks	Compound separation, purification, analysis and biological properties	256	Vasudevan et al. (2021)
Recent advances in marine algae oligosaccharides: Structure, analysis, and potential prebiotic activities	Brief review, structure and activity. MALDI analysis listed in table	124	Xie and Cheong (2022)
Recent research advances in polysaccharides from *Undaria pinnatifida* (edible seaweed): Isolation, structures, bioactivities, and applications	Contains large tables citing references to isolation, structural characterization and biological activity	140	Zeng et al. (2022)

Two papers are highlighted. Using an FT‐ICR instrument in positive ion mode, Nicolardi et al. (2021) have recorded the MALDI spectra from super‐DHB (s‐DHB) of several large polysaccharides and confirmed their structure by observation of ISD fragments where both glycosiodic and cross‐ring fragments were observed. Polysaccharides included a 16‐mer chain with a [(→4)‐Rha‐α‐(1→3)‐Man‐β‐(1→) repeat ([M + Na]^+^, monoisotopic, *m/z* 9900.573 (calc. *m/z* 9900.543)), a 100‐mer linear polymannoside ([M + Na]^+^ major isotopic peak, *m/z* 16339.680 (calc. *m/z* 16339.397)) and a 151‐mer branched polymannoside (M + Na]^+^, average, *m/z* 24610.58)).

Depolymerization of plant polysaccharides with periodate at 121^o^C has been used to provide a unique MALDI‐TOF fingerprint profile of all investigated polysaccharides except xyloglycan (Pandeirada et al., 2022). The method was able to differentiate polysaccharides such as birch wood xylan vs wheat arabinoxylan vs rye arabinoxylan, and guar galactomannan vs locust bean galactomannan. Principal component analysis and hierarchical cluster analysis of the MALDI‐TOF MS data highlighted the structural heterogeneity of the polysaccharides.

Applications of MALDI to the analysis of polysaccharides in plants, animals and algae are listed in Table 12 and in lower organisms are in Table 13.

**Table 12:** Use of matrix‐assisted laser desorption/ionization‐mass spectrometry for examination of carbohydrate polymers from plants, animals and algae.

Species or glycan source	Carbohydrate	Methods^a^	Notes	References
*Agave angustifolia* Haw	Fructans	*R*‐TOF (**DHB**)	Identification and evaluation of the fermentation of acetylated agave fructans with *Saccharomyces boulardii* as a probiotic	Buitrago‐Arias et al. (2021)
*Agave tequilana* Weber var. azul	Fructans	TOF, IMS‐Q‐TOF, (**CHCA**) imaging	Localization and composition in stem and rhizome	Pérez‐López et al. (2021)
Agavin (fructan) from *Agave tequilana* Weber Var. Blue (commercial)	Fructan	TOF (**DHB**)	Study of the effect of dietary agavin supplementation in blood parameters and antioxidant enzymes of juvenile Nile tilapia (*Oreochromis niloticus*) under stress conditions	Flores‐Méndez et al. (2022)
*Alhagi pseudalhagi,* (camel thorn)	Hetero‐polysaccharide (14 residues)	*L*‐TOF/TOF (**DHB**)	Structural elucidation and osteogenic activity	Ye, Li, et al. (2021)
*Allium sativum* (garlic)	Oligosaccharide with Fru, Glc, GalA, Gal, Man, Ara, Rha	TOF	Preparation and structural characterization	Jiang, Ran, et al. (2022)
*Allium schoenoprasum*	Major polysaccharide with Ara, Gal, Glc, Fru (ratio 1:2:2:5)	*R*‐TOF/TOF (**CMBT**)	Purification and structural characterization	Zhang, Zheng, et al. (2021)
*Apium graveolens* (celery)	Rhamnogalacturonan‑II	TOF (**DHB**), ESI, GC/MS	Structural characterization	Barnes et al. (2021)
*Arabidopsis thaliana*	Xyloglucans	TOF/TOF (**CMBT**/**DHB**)	Study of the effect of *O*‐acetylation levels of cell wall xyloglucan on sensitivity to aluminium	Wu, Tao, et al. (2022)
*Aspergillus flavus* and *A. fumigatus*	Chito‐oligosaccharides	TOF/TOF (**DHB**)	For study on inhibitory activity and mechanism of chitosan oligosaccharides on *Aspergillus flavus* and *A. fumigatus*	Ke et al. (2022)
*Astragalus arbusculinus* gum	Carbohydrate with Glc, pinitol, and Ara (relative molar ratio of 4:1)	TOF (**CHCA**)	Isolation, characterization, and antioxidant activity	Ahmadi, Rezadoost, et al. (2022)
*Avena sativa* (oat, bran)	β ‐Glucan	TOF (**DHB**)	Structural studies of water‐insoluble β‐glucan and its effect on improving lipid metabolism in mice fed high‐fat diet	Yu, Wang, et al. (2021)
Birch and beech wood	Xylo‐oligosaccharides	TOF/TOF (**DHB**)	Hydrolysis of xylans catalyzed by xylanase from *Bacillus subtilis*	Wei et al. (2021)
*Bletilla formosana*	Glucomannans	*R*‐TOF/TOF	Structural determination of two glucomannans and their protective effect on inflammation *via* inhibiting NF‐κB pathway	Gu (2022)
Chicken jejunum	Oligosaccharides	TOF (**DHB**), (per‐Me)	Qualitative and quantitative profiles of jejunal oligosaccharides in broiler chickens receiving different dietary levels of fiber, protein and exogenous enzymes	Lin and Olukosi (2021)
*Coreopsis tinctoria* (Kunlun chrysanthemum flower tea)	Oligosaccharides	*L*‐TOF/TOF	Structural elucidation of three novel oligosaccharides and their bioactivities (hyperglycemia and neuroinflammation)	Yu, Chen, et al. (2021)
*Crassostrea hongkongensis* (oyster)	Polysaccharide (*α*‐(1→4) d‐linked Glc backbone and (→4,6)‐*α*‐d‐Glc‐(1→) branches every 4.7 residues	TOF/TOF	Oyster polysaccharides shown to ameliorate intestinal mucositis and improve metabolism in 5‐fluorouracil‐treated S180 tumour‐bearing mice	Baxa et al. (2020)
*Crataegus azarolus* (yellow hawthorn) fruit	Polysaccharides	TOF (**DHB**)	β‐(1 → 4)‐Linked glucose and mannose residues with monosaccharide branches of α‐(1 → 6) galactose and *O*‐acetyl substituents.	Bensaci et al. (2022)
*Cremastra appendiculata* (medicinal plant)	Mannoglucan	TOF, HPLC, NMR, GC/MS, IR	Structural characterization	Zhang, Bi, et al. (2021)
*Cyamopsis tetragonolobus* (guar)	Oligosaccharides	MALDI‐TOF	Characterization of resultant oligosaccharides from guar galactomannan upon depolymerization by nonspecific enzymes	Shobha et al. (2022)
*Cynara cardunculus* var. *scolymus* (artichoke)	Pectic oligosaccharides	TOF	Characterisation and virtual screening of prebiotic properties using *in silico* colonic fermentation	Sabater, Blanco‐Doval, Margolles, et al. (2021)
Digesta and excreta from broiler chicken	Arabinoxylo‐oligosaccharides	TOF/TOF (**DHB**)	Dietary endo‐xylanase shown to alter arabinoxylan utilization	Kouzounis et al. (2022)
*Evodia lepta* (Spreng) Merr.	Oligosaccharides	TOF (1‐(4‐cyanophenyl)‐4‐piperidinyl hydrazide (CPH) derivative)	Characterization, antioxidant and antitumor activities. Use of different extraction methods. Microwave‐assisted extraction best.	Xiong, Liang, et al. (2022)
*Glycine max* (soybean)	Polysaccharides	*L*‐TOF (**DHB**)	Study of the effect of microwave‐assisted acid extraction on the physicochemical properties and structure of soy hull polysaccharides	Cai, Zhang, et al. (2022)
*Glycine max* (soybean)	Polysaccharides	TOF (**DHB**)	Chemical composition and sugar spectroscopy of polysaccharides obtained by microwave‐assisted salt extraction	Li, Zhang, Cheng, et al. (2022)
*Hermetia illucens* (black soldier flies)	Chitosan	*R*‐TOF/TOF (**CHCA**, **DHB**)	Structure and enzymatic hydrolysis	Lee, Kim, Nam, et al. (2022)
*Hordeum vulgare* (spring barley)	Fructans	TOF/TOF (**DHB**)	Genome‐wide association study reveals the genetic complexity of fructan accumulation patterns in barley grain	Matros et al. (2021)
*Hordeum vulgare* (spring barley)	Fructose	IMS‐TOF (**DHB**), MS/MS, GC/MS	Structural determination and immunomodulatory properties	Lemieszek et al. (2022)
*Hordeum vulgare (*barley)	BF‐1 (Mixture of (arabinoxylan, yeast‐derived β‐glucan, barley‐derived β‐glucan, and type II arabinogalactan)	TOF/TOF (**DHB**), LC‐MS/MS	Structural identification of active moiety in antitumor metastatic polysaccharide purified from fermented barley	Son et al. (2022)
Horse gut (feces and stomach contents)	Cellulose	TOF (per‐Me)	Oxidation of cellulose by lytic polysaccharide monooxygenases	Liu, Yu, et al. (2022)
*Inula helenium* L.	Inulin (fructan)	*R*‐TOF (**DHB**)	Optimization of methods for inulin extraction	Ahmadi, Farimani, et al. (2022)
*Lignosus rhinocerotis* (Cooke) Ryvarden (fungus)	β‐Glucans	*R*‐TOF/TOF (**CHCA**)	Structural determination and effect on intestinal mucosal wound healing	Veeraperumal et al. (2021)
*Malus domestica* (apple, pomace)	Xyloglucans	TOF (**DHB/**DMA)	Analysis of xyloglucans for potential developments in industrial applications	Chen, Mac‐Béar, et al. (2022)
*Nyctanthesarbor‐tristis* leaves	Xylo‐oligosaccharides	*R*‐TOF (**DHB**), SEC, HPAEC, GC/MS	Production and identification of bioactive oligosaccharides by a combination of enzymatic, HPAEC and MALDI‐TOF‐MS techniques	Ali, Mukherjee, et al. (2021)
*Oryza sativa* (rice)	Oligosaccharides	*R*‐TOF/TOF (**DHB**, **CMBT**)	Poaceae‐specific cell wall‐derived oligosaccharides shown to activate plant immunity *via* OsCERK1 during *Magnaporthe oryzae* infection	Yang, Liu, et al. (2021)
*Oryza sativa* (rice), cell walls	Xylans	TOF (**DHB**)	Organically‐bound silicon shown to enhance resistance to enzymatic degradation	Pu et al. (2021)
*Oryza sativa* (rice)	Hydroxycinnamic acid‐modified xylan	TOF/TOF (**DHB**), (procainamide derivative)	Hydroxycinnamic acid‐modified xylan side chains and their cross‐linking products in rice cell walls are shown to be reduced in the xylosyl arabinosyl substitution of xylan 1 mutant	Feijao et al. (2022)
*Panax quinquefolius*, L (ginseng)	Polysaccharides	TOF/TOF (**DHB**)	Structural analysis of red ginseng polysaccharides	Jin, Oh, et al. (2021)
*Pennisetum glaucum* (pearl millet)	Oligosaccharide (Glc, Gal)	TOF/TOF (**DHB**), GC/MS, TLC, FTIR	Characterization and evaluation of their prebiotic potential	Mondal et al. (2022)
*Pinctada fucata* (pearl oyster), shells	Sulfated polysaccharide	TOF/TOF (**DHB**)	Sulfated polysaccharide shown to improve scopolamine‐induced memory impairment	Yamagami et al. (2021)
*Polygonatum cyrtonema*	Fructan and galactan	TOF/TOF (**DHB**)	Structures and their utilization by probiotic bacteria	Zhang, Chen, Luo, et al. (2021)
*Polygonatum cyrtonema* Hua	Fructo‐oligosaccharide	TOF (**DHB**)	Structural characterization and treatment of LPS‐induced peritonitis in mice	He et al. (2021)
*Polygonatum odoratum* (Mill.) Druce	Cell wall polysaccharides	TOF/TOF (**DHB**)	Structure and biological activities of cell wall polysaccharides in the rhizome, stem, and leaf	Li, Hsiung, et al. (2022)
Poplar sawdust (genus Populus)	Cello‐oligosaccharides	TOF (**DHB**)	Production of high‐yield short‐chain oligomers from cellulose *via* selective hydrolysis in molten salt hydrates	Ma, Lin, et al. (2022)
*Rhizobium radiobacter* ATCC 1333	Cyclic β‐1,2‐glucans	TOF (**DHB**)	Isolation and use for increasing solubility of curcumin by complexation	Wu, Zhang, Gao, et al. (2022)
Rosaceae family: Apple and sweet cherry	Xyloglucans	TOF/TOF (**DHB**/DMA)	Comparison of cell wall chemical evolution during the development of fruits	Lahaye et al. (2021)
*Sabia parviflora*	α‐Glucoside	TOF	Isolation, structure identification and hepatoprotective activity	Zhang, Li, et al. (2021)
*Sepioteuthis lessoniana* (squid)	Sulfated chitosan	*R*‐TOF (**CHCA**)	Sulfated chitosan converted to low molecular weight form with gamma radiation. Antituberculosis activity	Ramachandran et al. (2022)
*Solanum lycopersicum* (tomato)	Hemicellulose	Cutinase and endo‐1‐4‐ß‐d‐glucanase, TOF (**DHB**/DMA)	Investigation of cutin polymer matrix structure during fruit development	Reynoud et al. (2022)
*Taraxacum kok‐saghyz* Rodin	Inulin	*R*‐TOF (**DHB**)	Optimization of extraction by response surface methodology	Chen, Wang, Dong, et al. (2022)
*Tichocarpus crinitus* (red alga)	Kappa/beta‐carrageenan	*R*‐TOF/TOF (**DHB**), MS/MS, LC‐MS	Structural determination. Potential inhibitor of HIV‐1	Yermak et al. (2021)
*Ulva* sp.	Oligo‐ and polysaccharides	TOF	Effect on human skin fibroblasts	Fournière et al. (2021)
*Ulva fasciata* (green seaweed)	Ulvan, a water‐soluble polysaccharide (‐4‐β‐d‐Glc*p*A‐(1→4)‐α‐l‐Rha*p*‐(1→)‐ repeat	*L*‐TOF/TOF (**DHB**, ‐ve)	Ulvan shown to consist of rhamnose, rhamnose‐3‐sulfate, xylose and numerous uronic acid residues and to induce resistance in wheat against *Zymoseptoria tritici* without major alteration of leaf metabolome	de Borba et al. (2021)
*Vaccinium macrocarpon* (cranberry)	Oligosaccharides and proanthocyanidins	*R*‐TOF (**DHB**)	Proanthocyanidin‐enriched cranberry extract shown to induce resilient bacterial community dynamics in a gnotobiotic mouse model	Neto et al. (2021)
*Vaccinium* sect. *Cyanococcus* (bulberry)	Xyloglucan and pectin	*R*‐TOF/TOF (**DHB**)	Structure and composition of glycans that bind anthocyanins during fruit puree processing	Hotchkiss et al. (2021)
*Wolffiella repanda* (duckweed)	Rhamno‐galacturonan‑II	TOF (**DHB**), ESI, GC/MS	Structural characterization	Barnes et al. (2021)
Wood (oak, hornbeam, walnut)	Various oligosaccharides	TOF (**DHB**)	Destructive behaviour of wood by the white‐rot fungus *Fomes fomentarius*	Bari et al. (2021)
Commercial	Hyaluronic acid (low molecular weight)	TOF/TOF (**DHB**)	For study of rheological properties of hyaluronic acid diluted solutions as components of cosmetics	Saitarly et al. (2022)
Commercial	Chitin and chitosan	TOF (**DHB**)	Development of prediction models for adsorption properties of chitin and chitosan for micropollutants	Cho, Lim, et al. (2021)
Commercial	Chitosan	TOF (**CHCA** [peptides], **DHB** [glycans])	Structural characterization by MALDI	Jung, Lee, et al. (2021)

^a^ Format (not all items present): Depolymerization method, MALDI method (**matrix**), compounds run (derivative), other methods.

**Table 13:** Use of matrix‐assisted laser desorption/ionization‐mass spectrometry for examination of carbohydrate polymers from lower organisms.

Species	Carbohydrate	Techniques^a^	Notes	References
*Auricularia auricula‐judae*	Black fungus polysaccharide (β‐glucan)	TOF/TOF (**DHB**)	Investigation of anti‐hepatoma activity	Cai, Zhou, et al. (2021)
*Bacillus amyloliquefaciens* WX‐1	Konjac glucomannan	TOF (**DHB**)	Production, characterization, and prebiotic activity	Wan, Wei, et al. (2021)
*Colaconema formosanum*	Mannose	TOF	Investigation of bioactive compounds for industrial use	Lee, Huang, et al. (2022)
*Enterobacter soli*	Exopolysaccharides	TOF (**DHB**, +ve, ‐ve)	Shown to be effective at removing chromium from industrial effluent	Kailasam et al. (2022)
*Ganoderma lucidum*	β‐d‐glucan	TOF/TOF (**DHB**/TFA)	Microwave‐assisted degradation and the structural and immunoregulatory properties of oligosaccharide fractions	Qin, Ma, et al. (2022)
*Lactobacillus paraplantarum* KM1	Exopolysaccharides (EPS)	TOF (**DHB**)	Purification and characterization of novel exopolysaccharides produced from *L. paraplantarum* KM1 isolated from human milk and its cytotoxicity	Sharma, Sharma, et al. (2021)
*Mucilaginibacter* sp. ERMR7:07 (glacier bacterium)	Exopolysaccharides	TOF/TOF (**DHB**), GC/MS, NMR, UV, FTIR	Production, characterisation, and applications	Kumar et al. (2022)
*Mycobacterium bovis* (BCG)	Polysaccharides	TOF (per‐Me)	Structural chacterization	Luo, Song, Chang, et al. (2022)
*Nostoc commune*	Mannose	TOF	Investigation of bioactive compounds for industrial use	Lee, Huang, et al. (2022)
*Paenibacillus polymyxa* A 26	Exopolysaccharides	TOF (**DHB**)	Silica particles shown to trigger the EPS production of harsh environment isolates of growth‐promoting rhizobacteria and increase their ability to enhance wheat biomass in drought‐stressed soils	Fetsiukh et al. (2021)
*Pseudomonas aeruginosa*	Pel polysaccharide	*R*‐TOF (**DHB**), GC/MS, NMR	Structural determination (dimeric repeat of α−1,4 linked GalN and GalNAc)	Le Mauff et al. (2022)
*P. aeruginosa*	Malto‐oligosaccharides	TOF/TOF (**DHB**)	Trehalose and α‐glucan shown to mediate distinct abiotic stress responses	Woodcock et al. (2021)
*Saccharomyces cerevisiae* CNCM I‐3856	Hetero‐polysaccharides	*R*‐TOF/TOF (**DHB**), (per‐Me), GC/MS	Structural characterization and anti‐adhesive properties against *E. coli* associated with Crohn's disease	Sivignon et al. (2021)
*Sarcodia suae*	Mannose	TOF	Investigation of bioactive compounds for industrial use	Lee, Huang, et al. (2022)
*Sargassum muticum*	Heterofucoidans	TOF	Use of acetone precipitation to extract heterofucoidans from autohydrolysis extracts	Acevedo‑García et al. (2021)
*Sphaerotilus montanus* (sheath‐forming bacterium)	Sheath‐forming polysaccharide	*R*‐TOF (**2,3‐DHB**)	Structural characterization using thiopeptidoglycan lyase which recognizes the 1→4 linkage between α‐d‐GalN and β‐d‐GlcA	Kashiwabara et al. (2021)
*Spirulina platensis*	Oligosaccharide	TOF/TOF (**DHB**)	Structural characterization and its effect on the faecal microbiota *in vitro*	Cai, Yi, et al. (2022)
*Usnea* sp. (lichen)	Amide‑containing β‑glucan	*R*‐TOF/TOF (**DHB**)	Development of method to release the glucan	Fernandes et al. (2021)
*Xanthomonas* pathogens	Xyloglucans	TOF/TOF	Investigation of xyloglucan processing machinery and its role in the transcriptional activation of virulence factors	Vieira et al. (2021)
Zygnematophyceae (green algae), several species	Xyloglucan	TOF/TOF (**DHB**)	Ancient origin of fucosylated xyloglucan discovered in charophycean green algae	Mikkelsen et al. (2021)

^a^ Format (not all items present): MALDI method (**matrix**), (derivative), other methods.

### 13.2 Milk sugars

Four reviews are relevant and are listed in Table 14.

**Table 14:** Reviews and general articles on analysis of milk.

Subject	Comments	Citations	References
Oligosaccharides in human milk, achievements in analysis: A review	Short review	40	Belusko et al. (2022)
Human milk oligosaccharides: Structure and functions	Chapter from the 94th Nestlé Nutrition Institute workshop	24	Bode (2020)
Structural and functional aspects of milk oligosaccharides	Health benefits and analysis	‐	Debnath et al. (2022)
Evolution of milk oligosaccharides: Origin and selectivity of the ratio of milk oligosaccharides to lactose among mammals	Discusses structures of milk sugars in many different species	182	Urashima et al. (2022)

Liou et al. (2021) have used carbon‐dioxide supercritical fluid chromatography (SFC), coupled with both evaporative light scattering detectors and UV‐vis detectors to separate 18 human milk glycans attached to an azidohexyl linker ((CH_2_)_6_‐N_3_). The authors were able to separate regioisomers and connectivity isomers which is a major limitation currently associated with carbohydrate analysis. The oligomers, with compositions ranging from disaccharides to hexasaccharides were well separated within 10 min.

A new class of milk sugars containing a (Gal)_3_ chain without (**190**) and with (**191**) an additional fucose residue has been identified in human milk (Hanisch & Kunz, 2021). The glycans are thought to be the first to be observed with branching on the 6‐arm of the terminal galactose of the core galactosyl‐lactose moiety.


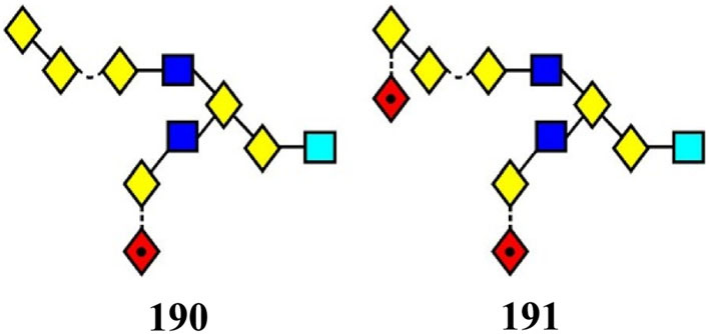


Other publications on the application of MALDI MS to the analysis of milk products are listed in Table 15.

**Table 15:** Use of matrix‐assisted laser desorption/ionization‐mass spectrometry for the characterization of carbohydrates from milk and milk products.

Source	Methods^a^	Notes	References
Human milk	*R*‐TOF/TOF (**CHCA**), (per‐Me)	Identification of new (Gal)_3_ and Fuc‐(Gal)_3_‐containing 6‐antennae (see text)	Hanisch and Kunz (2021)
Human milk	TOF, (**DHB**) (linkage‐specific amidation with d_5_‐aniline, Girard's P), LC‐MS	Identification of novel *α*2,3‐linked di‐/tri‐sialylated oligosaccharide isomers	Jin, Lu, et al. (2022)
Human colostrum and mature milk	PNGase F, TOF (**DHB**), (linkage‐specific amidation)	A preliminary study on isomer‐specific quantification of sialylated *N*‐glycans released from whey glycoproteins	Jin, Li, et al. (2021)
Human, cow, goat, sheep, and camel milk	*R*‐TOF/TOF (**DHB**), (per‐Me)	Identification and absolute quantification of milk oligosaccharides in different species	Shi, Han, et al. (2021)
Human milk	FT‐ICR, LTQ (MS/MS), (**DHB**)	Antibiofilm activity against multidrug resistant and susceptible isolates of *Acinetobacter baumannii*	Spicer et al. (2021)

^a^ Format (not all items present): MALDI method (**matrix**), (derivative), other methods.

### 13.3 Glycoproteins

MALDI has had a considerable impact on the analysis of glycoproteins and their attached glycans. Because of the complexity of these compounds, several processes are involved in their analyses. Glycoproteins with few glycosylation sites and a limited number of glycans at each site can now be resolved with high‐resolution instruments, but this method only gives a composition for the glycans following subtraction of the protein mass. Although not involving MALDI‐MS, the review on high‐resolution native mass spectrometry by Tamara et al. (2022), contains much useful information on the mass spectrometry of intact glycoproteins. The type (mainly *N*‐ and *O*‐linked) and attachment sites of the glycans are usually determined by analysis of derived glycopeptides following enzymatic hydrolysis, typically with trypsin, but structural analyses of the glycans themselves are usually determined following their release from the protein by chemical or, more commonly, enzymatic means. Such glycan analyses involves their composition and the determination of linkage and branching patterns between the constituent monosaccharides. Finally, all the individual pieces of information are combined to give the complete structure. General articles and reviews on glycoproteins and their analysis are listed in Table 16.

**Table 16:** Reviews and general articles on the analysis of glycoproteins.

Subject	Comments	Citations	References
Glycoproteomics	Glycoproteomic methods including sample selection; techniques for protein isolation, proteolytic digestion, glycopeptide enrichment and MS fragmentation	449	Bagdonaite et al. (2022)
Research progress in structure‐specific *N*‐glycoproteomics	Covers basic analytical procedures. In Chinese	57	Bi and Tian (2021)
The emerging role of cellular posttranslational modifications in modulating growth and productivity of recombinant Chinese hamster ovary cells	General review of glycosylation, phosphorylation and ubiquitination.	287	Bryan et al. (2021)
Site‐specific glycosylation of SARS‐CoV‐2: Big challenges in mass spectrometry analysis	*N*‐ and *O*‐glycans, Compares results from different analysis software. Little on MALDI	105	Campos et al. (2022)
Qualitative and quantitative analytical methods for intact glycopeptides	Book chapter – general overview	‐	Cao and Yang (2022)
Quantitative characterization of *O*‐GalNAc glycosylation	Short review several MS references but few that mention MALDI directly	51	Čaval et al. (2021)
Towards structure‐focused glycoproteomics	Covers literature for period 2018‐2020	254	Chernykh et al. (2021)
Seeing the forest through the trees: Characterizing the glycoproteome	Emphasises the importance of studying intact glycoprotein	102	Critcher et al. (2022)
Developments and perspectives in high‐throughput protein glycomics: Enabling the analysis of thousands of samples	Summary of current high‐throughput methods and some applications	92	de Haan, Pučić‐Baković, et al. (2022)
Advances in mass spectrometry‐based glycomics—An update covering the period 2017–2021	Glycan release, purification, derivatization, glycan separation, MS ionization, quantitation, bioinformatics	211	Donohoo et al. (2022)
Carbohydrate analysis of glycoconjugates	Short general review with protocols. Glycan release and analysis of glycopeptides	110	Gerwig (2021f)
Structural characterization of released glycans	*N*‐glycans (exoglycosidase digestion), *O*‐glycans, mucins, (with protocols)	34	Gerwig (2021e)
Analysis of sialic acids	General summary with protocols. Mass spectrometry but very brief on linkage‐specific methods.	32	Gerwig (2021h)
LC‐MS/MS in glycomics and lycoproteomics analyses	Derivatization including linkage‐specific methods. Separation methods. Software	202	Goli et al. (2021)
The glycosylation in SARS‐CoV‐2 and its receptor ACE2	Comprehensive review, *N*‐ and *O*‐glycosylation	477	Gong et al. (2021)
Glycosylation analysis	Chapter in book on monoclonal antibodies. Biological effects and analysis	152	Gstöttner, Kaur, et al. (2021)
*N*‐Glycosylation of milk proteins: A review spanning 2010–2022	Analysis protocol. Table of studies. Biological properties	107	Guan et al. (2022)
Glycomics and glycoproteomics: Approaches to address isomeric separation of glycans and glycopeptides	LC‐MS techniques, derivatization for sialic acid linkage. Table of methods	150	Gutierrez Reyes et al. (2021)
Advances in mass spectrometry‐based glycoproteomics: An update covering the period 2017‐2021	Metabolic labelling, enrichment, derivatization, quantification, ion mobility, bioinformatics	189	Gutierrez‐Reyes et al. (2022)
Glycan nanostructures of human coronaviruses	Comprehensive review with sections on each type of virus	119	Guo, Lakshminarayanan, et al. (2021)
Mass spectrometry‐based methods for immunoglobulin G *N*‐glycosylation analysis	Very comprehensive review. Mass spec, instrumentation, sample preparation, fragmentation, MALDI, LC/MS, CE	272	Habazin et al. (2021)
Calculating glycoprotein similarities from mass spectrometric data	Reviews analytical and statistical methods for determining glycoprotein molecular similarities from glycoproteomics data.	77	Hackett and Zaia (2021)
Automation of immunoglobulin glycosylation analysis	Mainly automation of methods for sample preparation	52	Hendel et al. (2021)
Negative‐mode mass spectrometry in the analysis of invertebrate, fungal, and protist *N*‐glycans	Emphasises some of the advantages of using negative ion MS for structural identification. Mainly MALDI applications	75	Hykollari et al. (2022)
Recent progress of analytical methods of proteomics based on mass spectrometry	Identification and quantitation. In Chinese	111	Ji, Fu, et al. (2021)
A mass spectrometry‐based glycotype‐centric cellular glycomics is the more fruitful way forward to see the forest for the trees	General review with comments. Native glycans, sialic acid derivatization, MS^n^ reactions	120	Khoo (2021)
Recent advances and trends in sample preparation and chemical modification for glycan analysis	Comprehensive review of glycan release, glycan enrichment, derivatization, use of stable isotopes	189	Kinoshita and Yamada (2021)
High sensitivity glycomics in biomedicine	Glycan sample preparation, clean‐up, analysis (CE‐MS, LC‐MS, PGC‐LC‐MS, ion mobility, MALDI), analysis of glycopeptides and glycoproteins	191ü	Lageveen‐Kammeijer, Küster, et al. (2022)
Analysis of glycosylation of IgG using mass spectrometry and its application	Contains discussion of several mass spectrometry methods (in Chinese)	146	Lai, Zhou, et al. (2021)
Mass spectrometry‐based analysis of IgG glycosylation and its applications	Intact IgG, glycopeptides and released glycans	132	Liu, Sun, et al. (2022)
Analytical and biochemical perspectives of protein *O*‑GlcNAcylation	Research over 35 years. Protein characterization. MS methods (ionization and fragmentation), enzyme characterization	680	Ma, Wu, et al. (2021)
Protein glycosylation in extracellular vesicles: Structural characterization and biological functions	General review. Many MALDI papers listed in tabular form	258	Macedo‐da‐Silva et al. (2021)
The hitchhiker's guide to glycoproteomics	General review (glycopeptide preparation, purification, MS fragmentation)	215	Oliveira et al. (2021)
A perspective on the Protein Data Bank's impact on the field of glycobiology	Discusses under‐representation of structures containing glycans	68	Prestegard (2021)
SARS‐CoV‐2	Combined with review on glycopeptide enrichment and derivatization	106	Pujić and Perreault (2022)
Progresses in mass spectrometry‐based plant and algae *N*‐glycomics and *N*‐glycoproteomics	Contains tables listing details of methods used and results	90	Qin, Qin, et al. (2022)
Qualitative and quantitative methods for *N*‐glycans in *N*‐glycomics	Covers ample pretreatment protocols *N*‐glycan release, purification and enrichment, separation, derivatization and some common qualitative and quantitative analysis strategies	120	Ren and Lu (2022)
*N*‐Glycoproteins in plant cell walls: A survey	Brief review covering structure of *N*‐glycans, overview of analytical methods, role of *N*‐glycans	80	San Clemente and Jamet (2022)
Quantitative methods for *N*‐glycosite containing peptides in *N*‐glycoproteomics	Book chapter – Reviews recently developed quantitative approaches in glycoproteomics	‐	Sun, Zhang and Lu, (2022)
High‐throughput glycomic methods	Comprehensive review. Historical overview. Discussion of various techniques including sample preparation for each one (HPLC, CE, MS, lectin microarrays), data processing, applications	438	Trbojević‐Akmačić et al. (2022)
mAbs *N*‐glycosylation: Implications for biotechnology and analytics	Small section on analytical methods	181	Wang, Liu and Voglmeir, et al. (2022)
Glycomics, glycoproteomics, and glycogenomics: An inter‐taxa evolutionary perspective	Discusses current glycomic, glycoproteomic, and glycogenomic methods to characterize protein glycosylation in less‐well‐studied eukaryotes	126	West, Malzl, et al. (2021)
The role of data‐independent acquisition for glycoproteomics	*N*‐ and *O*‐glycosylation, oxonium ions and limitations of the technique	78	Ye and Vakhrushev (2021)

#### 13.3.1 Isolation and concentration of glycoproteins and glycopeptides

Enrichment of glycoproteins, glycopeptides and purification of released glycans is an essential aspect of a successful structural analysis of these compounds and many methods have been devised; Table 17 lists a number of reviews. A few of the materials exhibit novel properties but most rely on established methods. Formation of boronate esters with compounds bearing *cis*‐diol groups is a popular method with the advantage that the boronate rings can easily be cleaved to release the free glycan. Other methods include hydrophilic attachment where many materials have been used. Of particular significance are zwitterionic materials, which are particularly useful for glycopeptides. Metal‐organic frameworks, with their high surface area, are also useful. Cotton has also been used, particularly for purification of *N*‐glycans and lectins provide a method for fractionating different types of these glycans. A protocol: “Enrichment of intact glycopeptides using strong anion exchange and electrostatic repulsion hydrophilic interaction chromatography” has been published (Bermudez & Pitteri, 2021).

**Table 17:** Reviews on methods for glycoprotein and glycan enrichment.

Subject	Notes	Citations	References
Recent strategies for using monolithic materials in glycoprotein and glycopeptide analysis	Use in chromatographic methods and for glycopeptide enrichment	158	Alla and Stine (2022)
Advances in enrichment methods for mass spectrometry‐based proteomics analysis of posttranslational modifications	Covers glycosylation and other PTMs such as phosphorylation and acetylation	117	Brandi et al. (2022)
Improving the study of protein glycosylation with new tools for glycopeptide enrichment	Materials used for glycan and glycopeptide enrichment classified by type	53	Chen, Dupard, et al. (2021)
Application of magnetic solid phase extraction in separation and enrichment of glycoproteins and glycopeptides	Covers categories of magnetic adsorbents and applications to human body fluids	83	Qi et al. (2021)
A review on recent advances in the enrichment of glycopeptides and glycoproteins by liquid chromatographic methods: 2016‐Present.	Nanoparticles, chromatographic methods, lectin affinity, metal‐organic frameworks	128	Kumari and Tetala (2022)
Recent progress and application of boronate affinity materials in bioanalysis	Uses in sample preparation and guidance for designing for specific requirements	207	Li, He, et al. (2021)
Advances in glycopeptide enrichment methods for the analysis of protein glycosylation over the past decade	Biological roles of glycosylation, analytical workflows, boronate affinity, *O*‐glycosylation	143	Li, Zhang, Xu, et al. (2022)
Methods for enrichment and assignment of *N*‐acetylglucosamine modification sites	Use of lectins, labelling methods, immunoprecipitation, MS analysis and software	56	Maynard and Chalkley (2021)
Recent advancements in glycoproteomic studies: Glycopeptide enrichment and derivatization	Briefly discusses different types of enrichment materials (combined with derivatization and SARS‐CoV‐2 glycosylation)	106	Pujić and Perreault (2022)
A guide to enrichment strategies for mass spectrometry–based glycoproteomics	Use of glycosidases, metal affinity chromatography, hydrophilic interaction chromatography, use of PGC and chemical coupling methods	512	Riley et al. (2021)
Simultaneous application of nanomaterials to separation of phosphorylated and glycosylated proteins	Book chapter, discusses methods such as immobilized metal affinity chromatography and metal oxide affinity chromatography	27	Sun, Deng and Shen (2021a)
Application of nanomaterials to separation of glycosylated proteins	Book chapter, discusses different carbohydrates and different chemical types (amino acids etc.) as functional groups, boronate affinity materials	233	Sun, Deng and Shen (2021b)
Selective enrichment methods for *N*‐glycosite containing peptides in *N*‐glycoproteomics	Current representative methods for glycoproteins/glycopeptides enrichment are summarized with discussion of advantages and limitations	94	Wang, Zhang and Lu (2022)
Advances in proteomic sample preparation and enrichment for phosphorylation and glycosylation analysis	Categorises enrichment methods by type of adsorbent	134	Xie, Feng, Zhang, et al. (2022)
Chemistry of magnetic covalent organic frameworks (MagCOFs): From synthesis to separation applications	General review with section on use for glycopeptide enrichment	179	Yadav et al. (2022)

A method for sialoglycopeptide enrichment, but which modifies the glycan, involves periodate oxidation, coupling with an alkyn‐containing hydrazide and click chemistry was employed to link the derivatized glycopeptides to Dde‐Azide or PEG‐azide resin (Li, Huang, et al., 2022). After centrifugation to isolate the resin‐bound glycopeptides, the resin was removed by incubation with hydrazine. The derivatized glycopeptides could then be examined by MALDI‐TOF MS or LC/MS.

Cai, Ren, et al. (2022) have developed a method which they call Ultrafast Glycoprotein Immobilization for Glycan extraction (UltraGIG) in which proteins are captured with NHS‐activated agarose resin *via* amide linkages. Contaminating compounds, salts, and so forth could then easily be removed and the glycans recovered by enzymatic cleavage. The method was used to study urinary *N*‐glycans in patients with diabetic kidney disease.

Table 18 lists 88 of the other materials that have been reported for purification and isolation procedures during the review period.

**Table 18:** Materials and methods used for the enrichment of carbohydrates, glycoproteins and glycopeptides.

Method	MALDI^a^	Materials	References
**Boronate‐based methods**			
Boronoisophthalic acid	*L*‐TOF (**SA**)	Glycoproteins, human milk	Ali, Hussain et al. (2021)
Phenylboronate functionalized magnetic nanoparticles	TOF/TOF (**CHCA**)	Low molecular weight glycoproteins	Dou et al. (2021)
Encapsulated magnetic nanoparticles with a polymer containing boronic acid groups	TOF	HRP Glycoprotein	Gharaghoushi et al. (2022)
Boronic acid‐functionalized mesoporous graphene−silica composites	TOF/TOF (**DHB**), LC/MS	*N*‐ and *O*‑linked glycopeptides (IgG, human serum)	Kong et al. (2021)
6‐Aminopyridine‐3‐boronic acid functionalized magnetic nanoparticles	MEKC	*Cis*‐diol‐containing biomolecules (HRP, human urine). Compounds not specified	Li and Dong (2021)
Boronate‐immobilized cellulose nanofiber‐reinforced cellulose microspheres (pH‐dependent)	UV	Glycoproteins (ovalbumin)	Li, Qiao, et al. (2022)
Boronate affinity sorbents based on thiol‐functionalized polysiloxane‐polymethacrylate composite materials	TOF/TOF (**CHCA**)	Glycopeptides (HRP, human serum)	Mompó‐Roselló et al. (2021)
Boric acid‐functionalized metal–organic frameworks	TOF	Glycopeptides (HRP), serum of cervical cancer patients	Rao et al. (2022)
Boric acid imprinted magnetic nanoparticles	TOF	Glycoproteins (HRP, ovalbumin)	Wang, Duan, et al. (2021)
Boric acid–functionalized magnetic covalent organic framework	TOF (**DHB**)	*N*‐Glycopeptides (HRP, human saliva)	Wang, Liu, Yan, et al. (2021)
Covalent organic framework material rich in boronic acid sites	TOF (**DHB**)	Glycopeptides (HRP, human saliva and serum)	Xie, Yan, et al. (2022)
Electrochemical sensor with surface imprinted boric acid	Electrochemical	P‐Glycoproteins	Yang, Song, et al. (2022)
Hollow MnFe_2_O_4_@C@APBA nanospheres	TOF	Glycopeptides	Zhang, Jin, et al. (2021)
**Hydrazide‐based methods**			
Chemical oxidation and reversible hydrazide chemistry	TOF/TOF (**DHB**), LC‐MS/MS	*O*‑GlcNAc Glycopeptides	Chen, Qin,et al. (2021)
**Graphite and carbon‐based methods**			
Magnetic porous carbon‐dependent platform	TOF	*N*‐Glycans (ovalbumin, urinary exosomes)	Wu, Zhang, et al. (2021)
**Carbohydrate‐functionalized materials**			
Bi‐amino acid functionalized biomimetic honeycomb chitosan membrane	TOF, LC‐MS/MS	*N*‐Glycopeptides (HRP), nasopharyngeal carcinoma serum	Fu et al. (2022)
Carrageenan functionalized magnetic carbon‐based framework	TOF (**DHB**)	*N*‐Glycopeptides from human saliva (IgG)	Jin, Zhu, et al. (2021)
Glycosyl imprinted mesoporous microspheres	LC/MS	Glycopeptide antibiotics	Tan et al. (2021)
Hydrophilic glucose functionalized quantum dots	TOF (**DHB**)	Glycopeptides (HRP, diabetic serum)	Xie, Feng, Fang, et al. (2022)
Absorbent cotton	LC‐MS/MS	Glycopeptides (mouse brain, seminal plasma)	Xin, You, et al. (2022)
**Amino acid and peptide‐functionalized materials**			
Magnetic binary metal oxide composites with hydrophilic tripeptide	TOF	Glycopeptides (HRP)	Chu et al. (2022)
Amino acid–functionalized zinc sulphide quantum dots	*R*‐TOF (**DHB**)	*N*‐Glycopeptides (HRP, human saliva)	Feng et al. (2022)
Glutathione‐functionalized two‐dimensional cobalt sulphide nanosheets	*R*‐TOF/TOF (**DHB**/H_3_PO_4_)	*N*‐Glycopeptides (HRP, IgG, human serum)	Gao, Bai, et al. (2021)
Covalent organic frameworks with glutathione and cysteine (denoted as COF‐S@Au@GC)	TOF/TOF (**DHB**)	Glycopeptides (HRP), glycopeptides from serum exosomes	Hua et al. (2022)
Asparagine immobilized cellulose/polymer nanohybrid	TOF/TOF (**DHB**)	*N*‐Glycans (ovalbumin, IgG, human serum)	Sajid, Saleem, Jabeen, Saleem, et al. (2022)
*O*‐Phospho‐l‐serine‐poly(glycidyl methacrylate‐co‐ethylene dimethacrylate) microspheres	TOF/TOF (**DHB**)	*N*‐Glycopeptides (IgG, HeLa glycoproteins) and phosphopeptides	Tang, Yu, et al. (2021)
Dandelion‐like silica nanoparticles modified with l‐glutathione	TOF/TOF (**DHB**)	*N*‐Glycopeptides (IgG, human serum)	Tian et al. (2021)
Tannic acid and l‐cysteine functionalized magnetic composites	TOF/TOF (**DHB**)	*N*‐glycopeptides (HRP, human serum)	Wang, Xu, et al. (2022)
Glutathione‐functionalized magnetic thioether‐covalent organic frameworks	TOF	Glycopeptides (HRP, exosomes)	Xiong, Jia, et al. (2022)
β‑Amyloid peptide 1−42‐conjugated magnetic nanoparticles	LC‐MS/MS	Glycoproteins (egg white)	Zhen et al. (2021)
Hydrophilic arginine‐functionalized mesoporous polydopamine‐graphene oxide composites	*R*‐TOF (**DHB**)	Glycopeptides (IgG)	Zheng, Pu, et al. (2022)
Dipeptide‐based polymeric material	TOF	Glycoproteins (IgG, HRP)	Zheng, Zhang, et al. (2022)
Graphene functionalized with structurally complementary amino acids	TOF, LC‐MS/MS	*N*‐Glycopeptides (HRP, human saliva and serum)	Zhu, Wu, et al. (2021)
**Metal‐organic frameworks**			
Melamine foam assisted in‐tip packed amine‐functionalized titanium metal–organic framework	TOF (**CHCA**, **SA**)	Glycopeptides (HRP, ovalbumin, IgG, human saliva)	Ali, Zhu, Wang, et al. (2021)
Metal‐organic framework (MF@PDA@UiO‐66‐NH_2_ composite)	TOF/TOF (**CHCA**)	Glycopeptides (HRP)	Ali, Zhu, Hussain, et al. (2021)
Gold nanoparticle‐glutathione functionalized metal‐organic frameworks	*R*‐TOF (**DHB**)	Glycopeptides (HRP, human saliva and serum)	Wu, Jin, et al. (2021)
Hydrophilic hollow zirconium organic frameworks	TOF	Glycopeptides (HRP)	He, Zheng, et al. (2022)
Zwitterionic dual‐functional metal‐organic framework nanocomposite.(In Chinese)	TOF (**DHB**)	Glycopeptides (HRP)	Li, Xie, et al. (2021)
Graphene oxide/chitosan foam incorporated with metal–organic framework	TOF/TOF (**DHB**)	Glycopeptides (HRP)	Liu, Gao, et al. (2022)
Bifunctional magnetic covalent organic framework	TOF (**DHB**)	Glycopeptides (IgG, rat liver)	Luo et al. (2021)
Fe_3_O_4_@SiO_2_@(ZreTi‐MOF)_10_‐NH_2_ Dual‐functionalized magnetic bimetallic metal‐organic framework composite	TOF/TOF (**DHB**), ESI‐MS/MS	Glycopeptides (IgG, human serum)	Pan, Zhang, Xiao, et al. (2021)
Magnetic dual‐hydrophilic metal organic framework	TOF (**DHB**)	*N*‐glycopeptides (HRP)	Su, Wang, et al. (2021)
Glutathione functionalized magnetic covalent organic frameworks	TOF (**CHCA**)	Glycopeptides (HRP)	Su et al. (2022)
Hydrophilic MOFs‐303‐functionalized magnetic probe	TOF **(DHB**)	Glycopeptides (HRP)	Wang, Wang, Li, et al. (2022)
Gold nanoparticle‐glutathione functionalized MOFs	TOF (**DHB**)	Glycopeptides (HRP)	Wu, Jin, et al. (2021)
Hydrophilic hierarchical porous metal‐organic frameworks	TOF (**DHB**), LC‐MS/MS	Glycopeptides (IgG)	Zhu, Gu, et al. (2022)
**Lectins**			
α‐Mannose‐specific *Burkholderia cenocepacia* lectin A	LC‐MS/MS	*C*‑ and *O*‑mannosylated peptides	Hütte et al. (2022)
**Zwitterionic materials**			
Zwitterionic polymer modified graphene oxide	TOF/TOF (**DHB**)	Glycopeptides from urine of healthy subjects and patients with lung adenocarcinoma	Bai et al. (2022)
Zwitterionic HILIC with exposed choline group	LC‐MS/MS	Sialoglycopeptides	Chen, Yen, et al. (2021)
ZIC‐cHILIC functionalized magnetic nanoparticle	*L*‐TOF/TOF (**CHCA**)	Glycopeptides (HRP, fetuin)	Pradita et al. (2021)
Zwitterionic carboxybetaine‐based hypercrosslinked polymers	TOF/TOF	Glycopeptides (IgG)	Sun, Xu, et al. (2022)
Zwitterionic sulfobetaine vinylimidazole‐based monoliths	TOF/TOF	Glycopeptides (IgG)	Wang, Sun, Wu, et al. (2022)
Zwitterionic microspheres (HILIC mode)	TOF (**DHB**), LC‐MS/MS	*N*‑Glycopeptides (IgG, human serum)	Wu, Tang, et al. (2021)
Zwitterionic‐HILIC (ZIC‐HILIC) nanosphere (Fe_3_O_4_‐CG)	TOF **(DHB**)	Glycopeptides (HRP, Alzheimer's disease patients’ serum)	Yi, Shao, et al. (2022)
**Other methods**			
MXene cartridge (Ti_3_C_2_)	TOF/TOF (**DHB**)	*N*‐Glycans	Aguedo et al. (2022)
Mesoporous covalent organic framework microspheres	TOF/TOF	Glycopeptides (IgG)	Ba et al. (2022)
TiO_2_	LC‐Q‐TOF	Glycopeptides, simultaneous enrichment, on‐line deglycosylation	Chen, Zhang, Dong, et al. (2021)
PAMAM dendrimer‐assisted 3‐carboxybenzoboroxole‐functionalized magnetic nanoparticles	TOF/TOF	Glycoproteins (HRP, human saliva)	Fan, Yang, Huang, et al. (2022)
Three hydrophilic poly(glycidyl methacrylate‐co‐ethylene glycol dimethacrylated macroporous adsorbent resins	TOF, LC‐MS	*N*‐Glycopeptides (IgG, human serum)	Gao, Tang, et al. (2021)
Highly crosslinking core–shell magnetic (Fe_3_O_4_) nanocomposites	SDS, UV	Glycoproteins (IgG)	Guo, Yao, et al. (2022)
Dual‐functional Ti(IV)‐IMAC material	TOF/TOF (**DHB**)	Glycopeptides (RNase B, mouse lung)	Huang, Liu, et al. (2021)
EDMA‐co‐VPBA‐co‐VPA) monolith)	TOF	Glycopeptides (HRP, human serum)	Huang, Zheng, et al. (2021)
Hydrophilic hydrogel with a 3D network structure (Zn_2_+/SAP)	TOF/TOF (**DHB**)	*N*‐glycopeptides (HRP)	Jin, Gao, et al. (2022)
Magnetic polyaniline nanomaterial (Fe_3_O_4_@PANI)	FT‐ICR (**DHB**)	*N*‐Glycopeptides (ovine fetuin, transferrin, haptoglobin)	Lai, Zhang, et al. (2021)
Nitrogen‐rich linear porous organic polymers	TOF/TOF (**DHB**), LC‐MS/MS	Glycopeptides (IgG)	Li, Xu, et al. (2022)
Titanium (IV) ion affinity chromatography materials	ESI‐Q‐TOF	*O*‐Glycopeptides (fetuin)	Li, Dong, et al. (2022)
Hydrophilic magnetic mesoporous silica microspheres	LC/MS	*N*‐Glycopeptides, *N*‐glycans (HRP, human serum)	Liu, Ma, He, et al. (2021)
HILIC HPLC, automated method	TOF/TOF (**DHB**), LC‐MS	*N*‐Glycopeptides (IgG, human serum)	Liu, Zhu, et al. (2021)
Cyclen‐containing hydrophilic polymeric monolithic materials	TOF/TOF, LC‐MS/MS	*N*‐Glycopeptides (IgG, human serum)	Ma, Tang, et al. (2021)
(Thio)urea and crown ether polymer	TOF (**DHB**)	Sialylated glycopeptides (bovine fetuin)	Mavliutova et al. (2021)
Iminodiacetic acid (IDA)‑generated mesoporous nanopolymer	TOF/TOF (**DHB**), LC‐MS/MS	Glycopeptides (HRP, IgG, human serum)	Sajid et al. (2021)
Methyl methacrylate/ethylene glycol dimethacrylate/1,2‐epoxy‐5‐hexene polymer plus cysteic acid	TOF/TOF (**DHB**), LC‐MS/MS	*N*‐glycopeptides (HRP, serum glycoproteins)	Sajid, Saleem, Jabeen, Najam‑ul‑Haq, et al. (2022)
Zirconium modified adenosine triphosphate functionalized monolith	CE‐LIF	*N*‐Glycans (RNase B)	Shao et al. (2021)
Al^3+^‐doped‐TiO_2_ monodisperse microspheres	TOF/TOF (**DHB**)	Glycopeptides (IgG, α‐casein, human serum, nonfat milk)	Sheng, Xue, et al. (2021)
Hydrophilic graphene oxide‐dopamine‐cationic cellulose composites	TOF/TOF (**DHB**)	Glycopeptides (IgG, human serum)	Sheng, Li, et al. (2021)
Nanoparticle biomolecular corona‐based enrichment	LC‐MS/MS	Glycoproteins (fibrinogen)	Trinh et al. (2022)
Dopamine/graphene oxide linked to trypsin for hydrolysis and enrichment	LC‐MS/MS	Glycopeptides	Wang, Zhang, Wei, et al. (2022)
Core‐shell microporous organic polymer‐coated silica microspheres	TOF, LC‐MS/MS	*N*‐Glycopeptides	Wang, Tang, et al. (2022)
Thiazolidine modified magnetic nanoparticles	TOF	Glycated peptides	Wu, Fei, et al. (2022)
Fluorescent molecular imprinted polymers	Fluorescence	Glycoproteins (ovalbumin)	Xie, Li, et al. (2022)
Strongly hydrophilic mesoporous silica (Fe_3_O_4_@mSiO_2_‐TSG)	*R*‐TOF/TOF	Glycopeptides (HRP)	Xu, Wu, et al. (2021)
Hydrophilic mesoporous channel coupled with metal oxide, Fe_3_O_4_@TiO 2@mSiO_2_ ‐TSG nanomaterial	TOF	Glycopeptides (HRP, IgG, salivary glycopeptides)	Xu, Wu, et al. (2022)
Polyoxometalate‐covalent organic framework conjugate	SDS‐PAGE	Glycoproteins, phosphoproteins	Xu, Cao, et al. (2022)
Bowl‐like mesoporous polydopamine	TOF/TOF (**DHB**)	Glycopeptides (HRP)	Yan et al. (2021)
Hydrophilic nano‐floral inter‐polymeric material	TOF (**DHB**)	Glycopeptides (HRP)	Yang, Gao, et al. (2022)
Bifunctional super‐hydrophilic mesoporous nanocomposite (mTiO_2_ @AuCG)	TOF	Glycopeptide (HRP), phosphopeptides	Yi, Fu, et al. (2022)
Immobilized metal ion affinity chromatography	LC‐MS/MS	*O*‑GalNAc glycopeptides (human serum)	Yue et al. (2021)

^a^ Format (not all items present): MALDI method (matrix), other methods.

#### 13.3.2 Problems encountered during sample preparation

Morgenstern et al. (2022) have commented on the fact that boronate methods of glycan enrichment often suffer from poor performance. On investigation, they found that the choice of buffer made a major difference to the method's performance. By eliminating amine‐containing buffers, glycan yields could be improved by as much as 10‐fold.

Despite its widespread use, hydrophilic enrichment methods are associated with several problems including the need for relatively large amounts of starting materials, potential introduction of chemical artefacts such as formylation when high concentrations of formic acid are used, and biasing or under‐sampling of specific classes of compound such as *O*‐linked glycopeptides. Izaham et al. (2021) have investigated these shortcomings for the study of bacterial glycoproteomes using three *Burkholderia* species (*B. cenocepacia*, *B. Dolosa*, and *B. ubonensis*), confirming that short aliphatic *O*‐linked glycopeptides are typically absent from hydrophilic interaction liquid chromatographic (HILIC) enrichments, yet are readily identified in whole proteome samples. Using high‐field asymmetric waveform ion mobility spectrometry (FAIMS) fractionation, they showed that at high compensation voltages, these compounds can be enriched from complex samples, providing an alternative method to HILIC enrichment.

Glycans and glycoproteins have been observed to develop an artefactual compound that produces a peak 28 mass units above that of the target compounds, which slowly increases in abundance when the samples were stored at −20^o^C. The peak was not observed in samples stored at room temperature, +4^o^C, −80^o^C, or −196^o^C. A corresponding reaction was not observed with acetic of trifluoroacetic solutions. The cause of the peak was traced to formylation of one of the hydroxyl groups on the glycan but it was not clear why the reaction was only observed at −20^o^C (Zhi et al., 2022).

The position of acetyl groups on the 7, 8 or 9 positions of sialic acids (**192**) influences the extent to which some pathogenic viruses bind. It has recently been reported (Oh et al., 2022) that acetyl groups can migrate between these positions in the presence of base making it important to control the conditions during sample preparation if determination of the position of such acetylation is important.


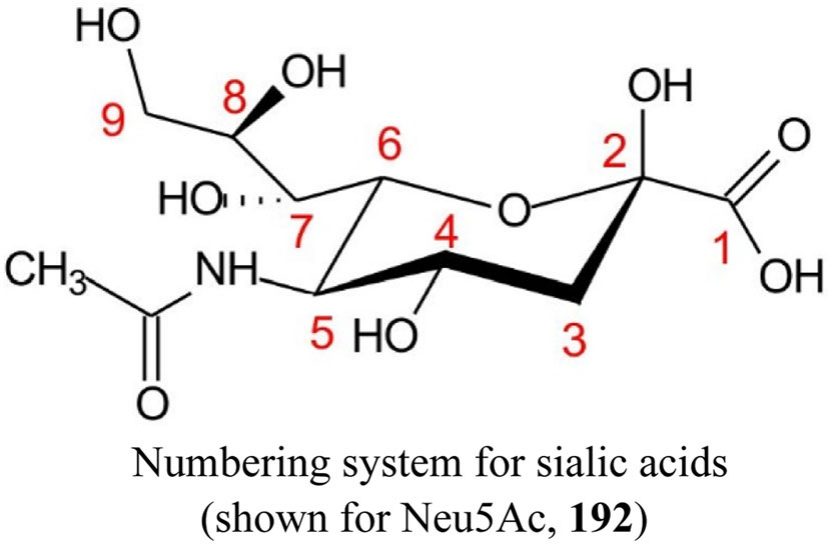


#### 13.3.3 N‐glycans

##### 13.3.3.1 Analysis of intact glycoproteins

**Figure 4:**
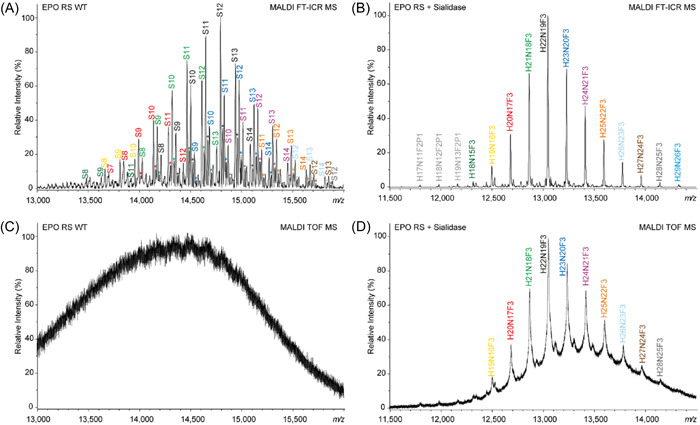
(A) MALDI‐FT‐ICR spectra of the doubly charged ion from EPO. (B) The spectrum of the glycans following desialylation. Spectra C and D are the corresponding MALDI‐TOF sprectra. From Lippold et al. (2021) with permission from Elsevier.

The high resolution capabilities of FT‐ICR instruments are now sufficient to resolve glycoforms of many glycoproteins as shown in Figure 4 for erythropoietin (EPO), a glycoprotein with three *N*‐glycosylation sites occupied mainly by bi‐, tri‐, and tetra‐antennary complex glycans (Lippold et al., 2021). The MALDI‐FT‐ICR spectra were obtained from 2,5‐DHAP, a matrix which minimizes loss of sialic acid and the spectrum of the doubly charged ion before and after incubation with sialidase is shown in Figure 4A. Figure 4B shows the corresponding MALDI‐TOF spectrum illustrating the much poorer resolution.

###### 13.3.3.1.1 Use of mass spectrometry to detect glycosylation of proteins

Detection of possible glycosylation of glycoproteins can simply be made by measuring their mass before and after incubation with a suitable endoglucosidase such as PNFase F. Thus, for the soluble complement receptor 1 investigated by Wymann et al. (2021), the difference between the mass of the intact glycoprotein (169,251‐178,056 ± 50 Da) and the product of PNGase F digestion (148,165 ± 50 Da) was interpreted as showing the presence of *N*‐glycans. Similarly, laccase from *Madurella mycetomatis* gave a mass of 67.4 kDa, which reduced to 62.0 following deglycosylation with Endo H. The, result indicated 8.8% glycosylation (Tülek et al., 2021).

###### 13.3.3.1.2 Detection of glycosylation sites and site occupancy

*N*‐Glycosylation occurs at asparagine (Asn) residues in a Asn‐Xxx‐Ser(Thr) motif where Xxx is any amino acid except proline. Not all sites are fully occupied and detection of glycosylation and its extent can be evaluated by the conversion of Asn to aspartic acid (Asp) giving a mass change of +1 Da following deglycosylation with PNGase F. An example of where this method has been used is the study by Dittner‐Moormann et al. (2021) on the transferrin biomarker for the congenital disorders of glycosylation (CDG) disease PMM2‐CDG where glycosylation with biantennary glycans is deficient. Transferrin has two *N*‐glycosylation sites and the results of PNGase F digestion of the glycoprotein from patients showed that the deficiency of biantennary glycosylation occurred equally at both sites.

##### 13.3.3.2 Analysis of N‐glycans

Detailed structural analysis of the *N*‐linked glycans is most commonly performed following their release from the glycoprotein.

###### 13.3.3.2.1 N‐Glycan release

Two types of method are commonly employed; chemical or enzymatic digestion.

####### 13.3.3.2.1.1 Chemical release

Hydrazinolysis has been used extensively to remove both *N*‐ and *O*‐linked glycans in the past, but is now rarely used because of its associated hazards and the accompanying complete degradation of the protein. However, some other chemical procedures are still being investigated. Thus, for example, Diaz et al. (2022) have advocated the use of sodium hypochlorite for releasing Man_9_GlcNAc_2_ (**193**) from *Phaseolus lunatus* beans for use as a standard reference material. Some decomposition was observed at the reducing terminal GlcNAc residue, caused by the chlorination of intermediate imines.


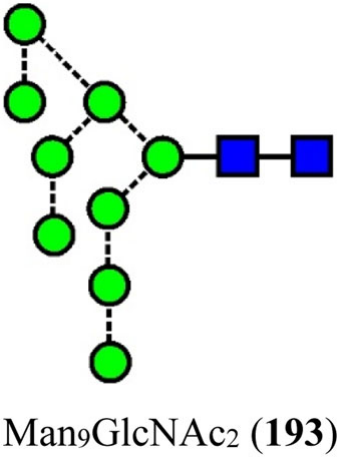


Because of the expense of enzymatic release, chemical release, such as with ammonia, is used when large quantities of *N*‐glycans are required. It has been reported, however, that the high pH of this reaction can cause epimerization of the reducing‐terminal GlcNAc (**117**) residue to ManNAc (**194**) in relatively large quantities (Liew, Chen, Tsai, & Ni, 2022). CID spectra can be used to differentiate the two isomers: Thus, for *N*‐glycans with GlcNAc at the reducing end, the intensity of the fragment ion produced through dehydration (loss of neutral *m *= 18 Da) is smaller than the intensity (50%) of the fragment ion produced through glycosidic bond cleavage (loss of neutral *m *= 221). By contrast, for *N*‐glycans with ManNAc at the reducing end, the intensity of this fragment ion is larger than 80% of the intensity of the fragment ion produced through glycosidic bond cleavage.


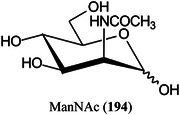


####### 13.3.3.2.1.2 Enzymatic release

Enzymatic release has now almost completely replaced chemical release and new enzymes are frequently being discovered. The most popular endoglycosidase for releasing *N*‐glycans is peptide *N*‐glycosidase F (PNGase F). However, this enzyme has some drawbacks. In particular, it operates optimally only in the neutral to slightly acidic pH range, suffers from steric inhibition, and its activity is severely compromised in the presence of reducing and denaturing substances. A new PNGase, isolated from the Gram‐negative bacterium *Rudaea cellulosilytica* (PNGase Rc) has been shown to overcome many of these disadvantages. It demonstrated broad substrate specificity for *N*‐glycan release from multiply occupied and natively folded proteins and is more tolerant to low pH conditions and to the presence of reagents such as urea and guanidinium chloride than PNGase F, making it suitable for use in hydrogen‐deuterium exchange experiments (Gramlich et al., 2022; Guo, Zhang, et al., 2022).

A recent study has compared the effects of detergents used to denature glycoproteins during release of *N*‐glycans with PNGase F (Kayili, Sakhta, et al., 2022). The released *N*‐glycans were labeled with procainamide (**195**), purified using cellulose‐containing solid‐phase extraction cartridges and analyzed by HPLC with fluorescence detection. The results showed that sodium dodecyl sulfate (SDS) and sodium deoxycholate (SDS + SDC) detergent combination provided the highest average fluorescence signal areas and intensities suggesting the most efficient release. It was also found that the average signal intensities of the detected *N*‐glycans were reduced when SDS and SDC were used with 1,4‐dithiothreitol (DTT) reducing agents. Profiles reflected the relative abundance of the released glycans rather than their compositions. A mixture of SDS, SDC and DTT produced a profile from human plasma consisting mainly of larger glycans (bi‐ and tri‐antennary) whereas the profile produced by SDS contained much more abundant low mass glycans (high‐mannose and degalactosylated biantennary). The authors also report the results of another relevant paper (Vilaj et al., 2020) in which it was reported that glycan profiles produced by PNGase F enzymes made by different manufacturers also differed.


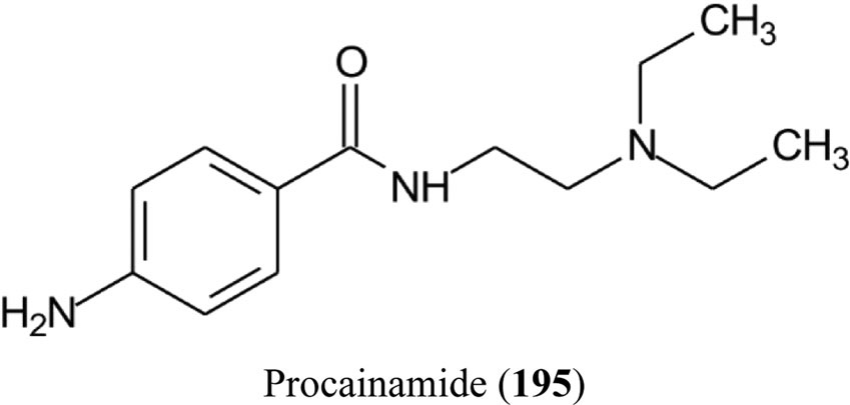


Release of glycans with PNGase F followed by removal of the deglycosylated proteins by use of C18 cartridges is a popular method for *N*‐glycan analysis. However, it is not so applicable to large scale samples. Consequently, Wang, Peng, et al. (2022) have developed an alternative method in which the proteins are precipitated with acetone. The yield of *N*‐glycans was tested with the standard glycoprotein samples, bovine fetuin and human serum. Compared to the amounts of *N*‐glycans from the use of C18 cartridges, most of the sialylated *N*‐glycans from human serum were detected with higher abundance after acetone precipitation. However, C18 showed a slightly higher efficiency for protein removal. Using the unfiltered human serum, around 97.7% of the proteins were removed by acetone precipitation, while more than 99.9% of the proteins were removed by C18 cartridges.

###### 13.3.3.2.2 Extraction and purification of released glycans

A comparative study of different methods for *N*‐glycan purification strategies, including filter‐aided sample preparation, de‐*N*‐glycosylated protein precipitation, and trypsin digestion followed by reversed phase‐based solid‐phase extraction (RP‐SPE) has concluded that the RP‐SPE method produced the best results (Guan, Zhang, Wang, et al., 2021). Glycans were permethylated using an optimized method (see Section 8.2.1.) and the method was used for examination of *N*‐glycans from the monoclonal antibodies trastuzumab and adalimumab.

Some methods, such as negative ion fragmentation, work best with neutral glycans and require a method for sialic acid removal. This can be accomplished enzymatically or by acid treatment. TFA is frequently used and has prompted Dong, Liu, et al. (2022) to study the reaction. It was found that, although most of the sialic acids were removed after heating at 75^o^C for 1 hour, it took 4 h for complete removal. Alternatively, when the concentration of TFA was raised to 5%, complete removal could be achieved in 1 h. However, the increased reaction times or TFA concentrations had adverse effects on peptides if the reactions were performed with glycopeptides.

###### 13.3.3.2.3 Analysis of released glycans

Use of HPLC with exoglycosidase digestion of fluorescently labelled glycans has been a popular method for structural analysis of *N*‐glycans but suffers from the disadvantage that exoglycosidases are not available for identification of all structural features, one consequence of which is that new structures are difficult to analyse. Nevertheless, protocols continue to be published, such as that from McLeod et al. (2021). The article covers HPLC, LC‐MS, and CE, with glycans labelled with, for example, procainamide or 2‐AB. Products of exoglycosidase digestions can, of course, be monitored by mass spectrometry with the advantage that the measured mass leads directly to the composition of the glycan. MALDI is particularly useful because of its property of producing mainly single ions from each glycan. Techniques such as ESI tend to produce ions in different charge states, particularly for the larger glycans.

####### 13.3.3.2.3.1 Methods to identify core‐fucosylated glycans

Abnormal expression of cell‐surface glycans with core fucosylation has been frequently observed in various cancers such as liver, colorectal, ovarian, prostate, and breast cancer, and has been associated with promotion of tumor growth, invasion, and metastasis. Consequently, there is much interest in methods that enable core from antenna fucosylation to be determined.

A method for achieving this distinction using low energy HCD claims to overcome problems caused by fucose migration leading to false positives (Chen, Shen, et al., 2022). The method involved observing the ratio of the Y_1_ + Fuc to the Y_1_ ions. If the ratio was greater than 0.1, the glycopeptide was considered to be core‐fucosylated, whereas, if the Y_1_F/Y_1_ ratio was less than 0.1, the glycopeptide was considered as solely antenna fucosylated. The method was tested with glycoproteins from human IgG (core fucosylation) and haptoglobin (antenna fucosylation). All 1026 core fucosylated glycopeptides from IgG were correctly identified whereas 156 of the 159 antenna‐fucosylated glycopeptides from haptoglobin were identified.

Another method has been developed by Tian, Wang, et al. (2022) with MALDI‐TOF being used to identify the final fucosylated glycopeptide. The enzyme Endo‐F3 from *Elizabethkingia meningoseptica* was used to cleave between the two GlcNAc residues of the core region of core‐fucosylated bi‐ and tri‐antennary glycans leaving Fuc‐GlcNAc attached to the protein. The, M‐endo‐F3 was used to attach the biotinylated probe **196** to the Fuc‐GlcNAc group effectively giving a biotinylated biantennary glycan attached to the protein. These labelled glycoproteins were cleaved with trypsin and the resulting fucosylated glycopeptides were captured with streptavidin beads. Cleavage with endo F3 left glycopeptides with attached Fuc‐GlcNAc which were identified by MALDI‐TOF and LC‐MS/MS.


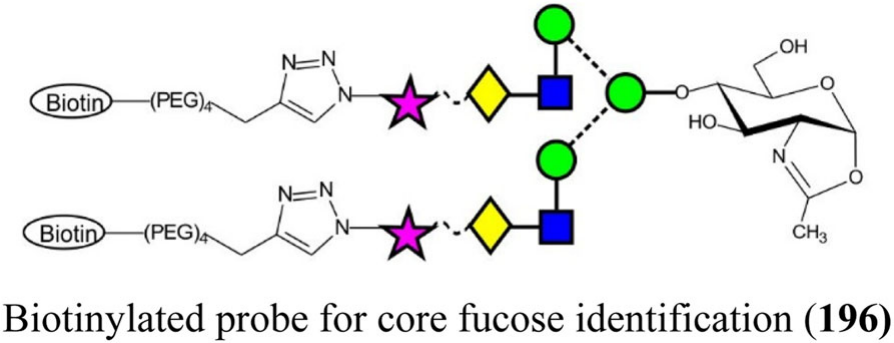


##### 13.3.3.3 Total methods for glycoprotein structure

A review with 91 references on sample preparation methods for *N*‐glycomics which covers glycan release, purification and enrichment, fluorescence labelling, permethylation and sialic acid derivatization has been published by Kayili, Atakay, et al. (2022). Protocols for various aspects of *N*‐glycan analysis are listed in Table 19.

**Table 19:** Reviews and general articles on *N*‐glycan protocols.

Subject	References
Site‐specific *N*‐glycosylation analysis of recombinant proteins by LC/MS^E^	Canis et al. (2021)
Analysis of intact glycoproteins by matrix‐assisted laser desorption/ionization time‐of‐flight mass spectrometry	Giménez et al. (2021)
Profiling of cellular glycoproteins and GSLs by Glycoblotting	Hanamatsu and Furukawa (2022)
Analysis of the biosynthesis and degradation of *N*‐glycan precursors in mammalian cells	Harada et al. (2021)
Analysis of monoclonal antibodies	Nmagu et al. (2021)

An integrated on‐line deglycosylation, labeling and purification method for *N*‐glycan analysis is believed by the authors (Wu, Zhang, Li, et al., 2022) to be the first time that such a simplified method has been developed. The method consists of an on‐line immobilized enzyme reactor for PNGase F release of the *N*‐glycans, direct labeling of released *N*‐glycosylamines (see Section 8.1.4) with 6‐aminoquinolyl‐*N*‐hydroxysuccinimidyl carbamate (AQC) (Scheme 6) and purification of the derivatives on a microfluidic chip. The process could be completed in within about 30 min. Good reproducibility and stability were achieved with the relative standard deviation (RSD) less than 10%. Intermediate stages were monitored by MALDI‐TOF but the method itself was designed for HPLC monitoring.

Another integrated method that claims to reduce glycan release and labelling from 2 days to 2.5 h involves use of Stage Tips, prepared in pipette tips containing 3 mm of cotton wool. The glycoprotein (IgG) was added, followed by PNGase F and appropriate buffers and the tips were incubated for 1 h at 45^o^C. The released glycans were then labelled with procainamide by incubation at 70^o^C for 1 h. The Stage Tips were centrifuged and washed with aqueous acetonitrile and TFA and the labelled glycans were eluted with water, purified with solid‐phase extraction cartridges for analysis by MALDI with DHB. The tips were also used to produce glycopeptides by incubation with trypsin (Kayili, Ragoubi, et al., 2022).

A method for obtaining *N*‐glycans from human milk has involved acetone precipitation of the glycoproteins, removal of the glycans with PNGase F, methylation of the sialic acids and use of the glycoblotting technique (aoWR [**197**] labelling) and analysis by MALDI‐TOF/TOF from DHB. To enhance sensitivity, glycans were also permethylated for MALDI analysis. *N*‐Glycans from human milk were the normal range of high‐mannose, hybrid and bi‐, tri‐, and tetra‐antennary compounds. Bovine milk contained similar compounds but in different proportions such as less core fucosylation and more high‐mannose and bisected structures (Wang, Zhao, Tao, et al., 2021).


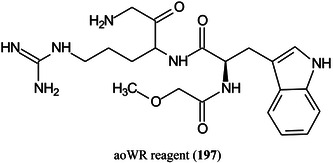


##### 13.3.3.4 Identification of new *N*‐linked glycan structures

Several novel *N*‐glycan structures have been identified during the review period. These compounds are usually produced by “lower organisms” and those synthesised by *Caenorhabditis elegans* have been reviewed (Paschinger et al., 2021).

Methyl hexoses feature in several of these new structures. Thus, Man_3_GlcNAc_2_ with 3 pentoses, each carrying 0 or 1 Me group (e.g., **198**) have been found in green algae (*Chlorella* species) (Choi et al., 2021).


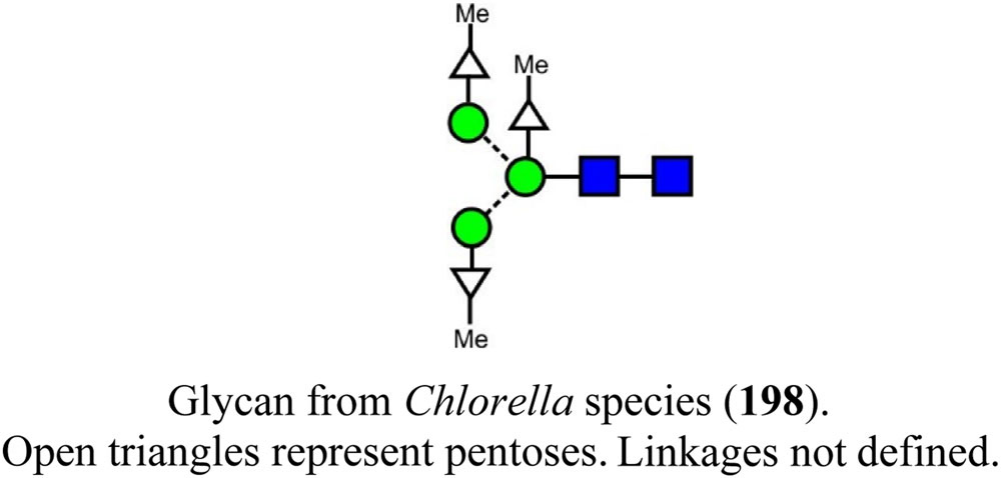


Mosses have been shown to contain the normal plant paucimannosidic glycans such as **199** and some of them, such a *Funaria hygrometrica* and *Plagiomnium undulatum* contain methylated constituents such as 2,6‐dimethyl‐mannose comprising the 6‐antenna (**200**, **201**) (Stenitzer & Altmann, 2022; Stenitzer, Mócsai, et al., 2022). Antennae with Lewis A termini (e.g., **202**) are also common.


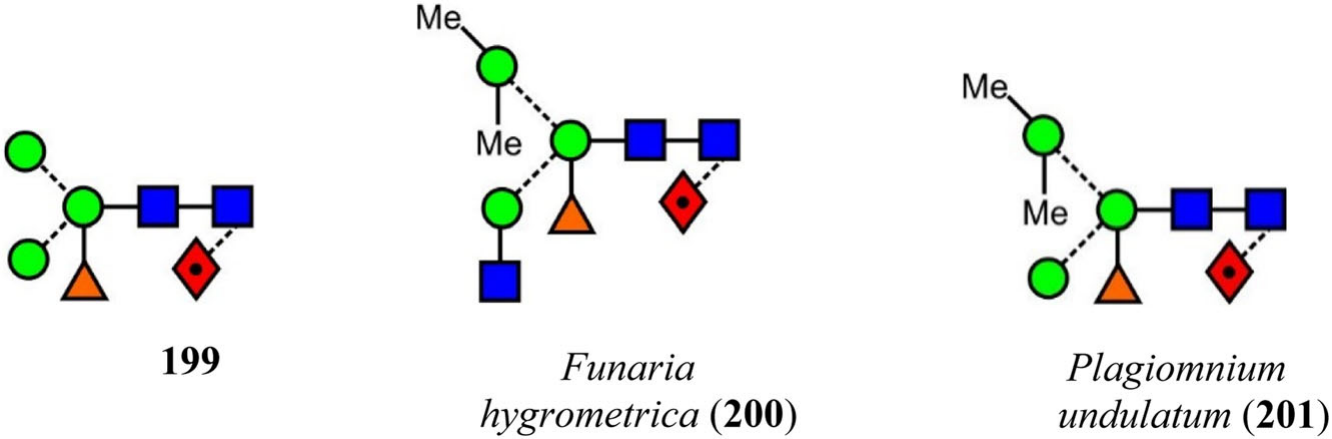



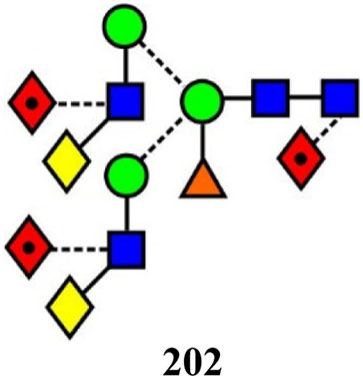


A new set of hybrid glycans with substituted bisecting GlcNAc residues (e.g., **203** and **204**) has been detected in human brain (Helm et al., 2021).


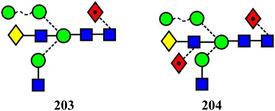


High‐mannose glycans with additional substituents also feature in some species. Thus, Man_3‐5_GlcNAc_2_ with one additional (unidentified) pentose and Man_2‐8_GlcNAc_2_ with one deoxy‐hexose have been found in the semi‑terrestrial microalga *Thorsmoerkia curvula* gen. et spec. nov. (Trebouxiophyceae, Chlorophyta) from Iceland. Glycans bearing both substituents were not found (Nicoletti et al., 2021). High mannose glycans with an additional galactose residue has been found attached to invertase expressed in the industrial yeast Y*arrowia lipolytica* (Szymański et al., 2022).

Among other structures, *N*‐glycans with GlcA (**205**) and phosphorylcholine (**206**) substitutions have been found in *N*‐glycans from the filarial nematode *Brugia malayi* (Petralia, van Diepen, et al., 2022).


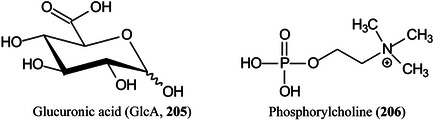


The *N*‐linked glycans from chloroviruses (family *Phycodnaviridae*) are considerably different from those of most other species and lack the normal trimannosyl chitobiose core. A recent review (Speciale, Notaro, et al., 2022) discusses their structure and biosynthesis. A new study on glycans from *Paramecium bursaria* chlorella virus MA‐1D by MALDI‐TOF/TOF‐MS (from DHB) and NMR has revealed three structures (**207**–**209**) that share several features with those of the other chloroviruses examined earlier except that they lack a distal xylose residue that was believed to be part of a conserved core structure for all the chloroviruses (Speciale, Di Lorenzo, et al., 2022). The authors believe that this result requires a reconsideration of the core structure for all chloroviruses.


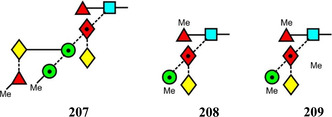


Further applications on the use of MALDI MS to the analysis of *N*‐glycans in specific glycoproteins and tissues are listed in Tables 20 and 21 respectively.

**Table 20:** Use of matrix‐assisted laser desorption/ionization‐mass spectrometry for examination of *N*‐glycans from specific glycoproteins.

Glycoprotein	Methods^a^	Notes	References
4‐1BB receptor (human)	PNGase F, TOF/TOF (per‐Me)	Structural determination and demonstration that *N*‐glycosylation facilitates 4‐1BB membrane localization by avoiding its multimerization	Sun, Kim, et al. (2022)
Alkaline phosphatase from *Neurospora crassa*	PNGase F, TOF (per‐Me), GC/MS	Characterization the *N. crassa* DFG‐5 α−1,6‐mannanase and demonstration of binding to the α−1,6‐mannose backbone of an *N*‐linked galactomannan found on cell wall glycoproteins.	Patel et al. (2022)
Alpha‐1‐acid glycoprotein (AGP)	PNGase F, TOF/TOF (**DHB**), (per‐Me)	Different glycoforms of AGP shown to contribute to its functional alterations in platelets and neutrophils	Sumanth et al. (2021)
Apo‐H (beta‐2‐glycoprotein)	PNGase F, TOF (**s‐DHB**), (Et esters)	Structural characterization (bi‐, tri‐antennary complex)	Javeed et al. (2021)
Alpha fetoprotein	PNGase F, MALDI (**DHB**), (Girard's P)	In development of a dual‐modal ratiometric immunoassay for diagnosis of hepatocellular carcinoma	Li, Pang, et al. (2022)
Asialofetuin	PNGase F, TOF, (+ve, ‐ve), (per‐Me)	Use of a novel lamprey antibody to characterize 3‐*O*‐sulfation	McKitrick et al. (2021)
Bilirubin oxidase	PNGase F, endo‐F1, TOF/TOF (**DHB**), (Bz oximes, Glycoblotting)	Effects on direct electron transfer‐type bioelectrocatalysis (high mannose)	Suzuki, Itoh, et al. (2022)
Bovine fetuin	PNGase F, TOF (**CHCA**) (amidation with aniline)	Control experiment on aniline derivatization of sialic acids for increased sensitivity for tissue imaging	Zhang, Shi, et al. (2022)
Bovine lactoferrin	PNGase F TOF (**DHB**), (Et ester, Girard's P)	Detection of *N‐*glycan changes of bovine lactoferrin at different stages of lactation	Jia et al. (2021)
Dynactin‑associated protein	PNGase F, Spiral‐TOF (**CHCA**), (3‐AQ)	Glycosylation of T/S cluster region (anomalous behaviour in SDS‐PAGE analysis)	Yin, Konishi, et al. (2022)
Erythropoietin	FT‐ICR (**DHAP**, **Cl‐CCA, CHCA, SA**) glycoprotein	Glycoform analysis of intact erythropoietin	Lippold et al. (2021)
Glycoprotein from *Abelmoschus esculentus* L. Moench (okra)	PNGase A, TOF (**DHB**)	Structural determination and antioxidant activity	Zhao, Xu, et al. (2021)
H11 protein from *Haemonchus contortus*	PNGase F, TOF/TOF (**DHB**), (per‐Me)	H11‐induced immunoprotection shown to be predominantly linked to *N*‐glycan moieties during infection	Wang, Liu, et al. (2022)
Human ACE2/IgG1‐Fc domain (ACE2‐Fc)	PNGase F, *R*‐TOF/TOF (**DHB**), (reduction, per‐Me)	SARS‐CoV‐2 spike protein variant binding affinity	Matthews et al. (2022)
IgG	PNGase F, IT‐TOF (**DHB**), (per‐Me)	Cytokines in the immune microenvironment shown to change the glycosylation of IgG by regulating intracellular glycosyltransferases	Cao, Song, et al. (2022)
IgG	PNGase F, *R*‐TOF/TOF (**DHB**), (Per‐Me and Me ester)	Enzyme ST6Gal1 shown not to be required for IgG sialylation	Oswald et al. (2022)
IgG, (surface variable domain)	PNGase F, TOF/TOF (s‐**DHB**), (Et ester)	Surface IgG glycosylation shown to affect autoantigen binding and acts as threshold for human autoreactive B cell activation	Kissel et al. (2022)
IgG (monoclonal against aflatoxin B1)	PNGase F, TOF (**DHB**)	As part of detailed structural analysis	Xing et al. (2022)
IgG from rhesus macaque	PNGase F, TOF/TOF (**DHB**), (2‐AA, Et ester/amide, linkage‐specific)	Alteration of *N*‑glycome during infection with the human parasitic filarial nematode *Brugia malayi*	Petralia, Santha, et al. (2022)
IgG1 and FcgRIIIa (158F and 158V allotypes)	PNGase F, *R*‐TOF (**s‐DHB**), (Et esters, linkage‐specific)	Study of the role of *N*‐glycosylation in FcgRIIIa interaction with IgG	Van Coillie et al. (2022)
Invertase glycoforms from *Saccharomyces cerevisiae*	PNGase F, *R*‐TOF/TOF (**DHB**)	To obtain optimum glycosylation for use as synthetic enzymes for methyl β‐d‐fructofuranoside	Andjelković et al. (2021)
Lactoperoxidase	EndoBI‐1, TOF (**DHB**), (2‐AA)	Model compound for evaluation of immobilized bifidobacterial endo‐ß‐*N*‐acetylglucosaminidase to generate bioactive compounds for food industry	Pekdemir et al. (2022)
Palivizumab (SynagisR), (monoclonal antibody)	PNGase F, *R*‐TOF/TOF (**SA**), (2‐AB), UHPLC, database structural interpretation	Structural determination mainly by HPLC (Man_5_GlcNAc_2_, biantennary complex)	Sran et al. (2022)
Prolyl‐alanyl‐specific endoprotease endopro from *Aspergillus niger*	PNGase F, FT‐ICR (s‐**DHB**), (2‐AA)	Structural determination and functional proteoform characterization (high‐mannose, site analysis)	van Schaick et al. (2021)
Prostate‐specific antigen (PSA)	Trypsin, FT‐ICR (**s‐DHB**), (sialic acid amidation)	Glycopeptide profiling of PSA from seminal plasma by MALDI‐MS	Wang, Kałuża, et al. (2021)
SARS‐CoV‐2, Spike protein S1 subunit RBD (Arg319‐Phe541)	PNGase F, TOF/TOF (**DHB**), (per‐Me), MS/MS	Site‐specific analysis (bi‐, tri‐antennary complex)	Antonopoulos et al. (2021)
SARS‐CoV‑2 Spike glycoprotein (from HEK293 and baculovirus‐insect cells)	PNGase F, TOF/TOF (**DHB**)	Site‐specific analysis (high‐mannose, bi‐, tri‐antennary complex)	Wang, Wu, et al. (2021)
SARS‐CoV‑2 nucleocapsid protein in HEK293 cells	PNGase F, TOF/TOF (**DHB**), per‐Me	Structural identification, (high‐mannose, bi‐antennary complex)	Supekar et al. (2021)
SARS‐CoV‑2 spike glycoprotein in CHO and HEK293 cells	PNGase F, TOF/TOF (di‐Me amide/amide derivs)	Use of a linkage‐specific sialic acid labeling strategy to define site‐specific glycosylation patterns	Wang, Wang, et al. (2021)
SARS‐CoV‐2 spike protein	PNGase F, QIT‐TOF (**DHB**), (per‐Me)	Study of the effect of *N*‐glycosylation of SARS‐CoV‐2 spike protein on the virus interaction with the host cell ACE2 receptor	Huang, Tan, et al. (2021)
Shark‐derived IgG new antigen receptor	PNGase F, TOF/TOF (**DHB**), BlotGlyco method	Identification of *N*‐glycans (high‐mannose, hybrid, di‐, tri‐ and tetra‐antennary complex) and production of monoclonal IgG from CHO cells	Enatsu et al. (2021)

^a^ Format (not all items present): Glycan release method and/or protease, MALDI method (**matrix**), (derivative), other methods.

**Table 21:** Use of matrix‐assisted laser desorption/ionization‐mass spectrometry for examination of *N*‐glycans from intact organisms, tissues or glycoprotein mixtures.

Source	Methods^a^	Notes	References
*Abelmoschus esculentus* L. Moench (okra)	PNGase A, TOF (**DHB**)	Structural characterisation and antioxidant activity of a novel *N*‐linked glycoprotein	Zhao, Xu, et al. (2021)
Amniotic membrane (human)	PNGase F *R*‐TOF/TOF (**DHB**), (per‐Me)	Glycoproteins in amniotic membrane shown to contain bisected complex *N*‐glycans	Chen, Zhang, Zhang, et al. (2022)
*Arabidopsis thaliana*	PNGase F, TOF	Changes of protein *N*‐glycosylation in the growth of *A. thaliana* and effects of enzymatic deglycosylation on root development (in Chinese)	Wang, Yang, Zhao, et al. (2021)
B3GAT1 and mCherry A549 cells	PNGase F, *R*‐TOF/TOF (**DHB**), (per‐Me)	Inhibition of sialyltransferase prevents infection by influenza respiratory viruses	Trimarco et al. (2022)
*Bombus terrestris*, (bumblebee, queen)	PNGase F, QIT‐TOF (**DHB**)	Hex_5_HexNAc_3_dHex_2_, Hex_3_HexNAc_3_dHex_2_, and Hex_4_HexNAc_3_Pen_1_ identified in study of the role of GAGs in aged rats	Ahn et al. (2021)
*Brugia malayi* (filarial nematode)	PNGase F, TOF/TOF (**DHB**), (2‐AA), exoglycosidase	Identification of high‐mannose glycans and complex glycans with GlcA and phosphatidylchloline	Petralia, van Diepen, et al. (2022)
Caveolin‑1 knockout mouse serum	PNGase F, QIT‐TOF (per‐Me)	Caveolin‐1 (protein) shown to influence *N*‐glycosylation	Chen, Wang, Wu, et al. (2022)
*Chlorella vulgaris* UTEX395	PNGase A, TOF/TOF	Structural analysis of secretome and *N*‐glycosylation, Man_3_GlcNAc_2_Pen_3_(Me)_2 or 3_	Choi et al. (2021)
CHO Cell lines (CHO‐K1, CHO‐S, and CHO‐Pro5)	PNGase F, *R*‐TOF/TOF	Structural determination. Differences in sialylation and fucosylation	Wang, Wang, Wu, et al. (2022)
EXT1 k.d. cells (ER membranes)	PNGase F, TOF/TOF (per‐Me)	Alternative glycosylation shown to control endoplasmic reticulum (ER) dynamics and tubular extension in mammalian cells	Kerselidou et al. (2021)
Extracellular vesicles secreted from *Plasmodium falciparum*‐infected red blood cells	PNGase F, TOF/TOF (**s‐DHB**), (per‐Me)	Sialylated *N*‐glycans shown to mediate monocyte uptake of the extracellular vesicles	Pilo et al. (2022)
Fish cell lines (6 species)	PNGase F, *R*‐TOF/TOF, (Me ester)	High‐mannose, hybrid, bi‐, tri‐antennary complex glycans. For predicting cellular receptors to the nervous necrosis virus	Gye and Nishizawa (2022)
*Funaria hygrometrica* (moss)	PNGase A, TOF (**DHB**), LC‐MS/MS, GC‐MS, (reduced)	Identification of plant‐like glycans (Man_3_GlcNAc_2_Fuc_1_Xyl_1_) and glycans with 2,6‐di‐Me‐Man at 6‐antenna. Also methylated glycans from other mosses. (see text)	Stenitzer, Mócsai, Zechmeister, et al. (2022)
*Haemonchus* (parasitic nematode)	PNGase A, F, TOF/TOF (**DHB**), (per‐Me)	Structural characterization, high‐mannose, paucimannosidic	Wang, Gao, et al. (2021)
HCT116 CRC cell line	PNGase F, TOF/TOF (**s‐DHB**), (Et ester)	Experiments to determine best lectin for detecting core fucose	Rubén et al. (2021)
HepG2 Cells	PNGase F, TOF/TOF **(DHB**), (aoWR derivatives), exoglycosidase	To evaluate effect of swainsonine on *N*‐glycosylation and its contribution to toxicosis in livestock grazing swainsonine‐producing plants	Morikawa et al. (2022)
HepG2 Cells	PNGase F, *R*‐TOF/TOF (**DHB**), (per‐Me)	Study of the origin of cytoplasmic GDP‐fucose used for glycan assembly	Sosicka et al. (2022)
HepG2 cells (plasma glycoproteins)	PNGase F, TOF/TOF (**DHB**), (2‐AB), HPLC, LC‐MS	DNA Hypomethylation shown to upregulate expression of the *MGAT3* gene and lead to changes in *N*‐glycosylation of secreted glycoproteins	Klasić et al. (2022)
HL‐60 promyelocytes	PNGase F, TOF/TOF (**DHB**), (per‐Me)	In study of inhibition of *O*‐glycan biosynthesis using the hexosamine analog Ac5GalNTGc	Wang, del Solar, et al. (2021)
HL‐60 cells	PNGase F, TOF/TOF (**DHB**/DMA), (Et ester, *p*‐toluidine)	Demonstration of altered sialidase expression in human myeloid cells undergoing apoptosis and differentiation	Hyun et al. (2022)
Human brain	*R*‐TOF, LC/MS, CID	Bisecting Lewis X in hybrid‐type glycans identified (see text)	Helm et al. (2021)
Human cervicovaginal fluid	PNGase F, TOF/TOF (**DABP**), (per‐Me)	Structural characterization. Found to reflect microbial community, immune activity, and pregnancy status	Wu, Grassi, et al. (2022)
Human dermal endothelial cells	PNGase F, TOF/TOF (**DHB**), (per‐Me)	Sialoglycans on lymphatic endothelial cells shown to augment interactions with Siglec‐1 (CD169) of lymph node macrophages	D'Addio et al. (2021)
Human erythrocytes	PNGase F, TOF/TOF (**CMBT**), (per‐Me	Structural determination, high‐mannose, hybrid, bi‐, tri‐antennary complex, bisects, *N*‐acetyl‐lactosamine extensions	Bua et al. (2021)
Human serum and cerebrospinal fluid	PNGase F, *R*‐TOF/TOF, (aoWR), exoglycosidase (2‐AB)	Detection of novel low‐molecular‐weight blood group‐specific glycans in serum and cerebrospinal fluid	Furukawa et al. (2021)
Human umbellar vein endothelial cells	PNGase F, TOF (per‐Me)	To assess adhesion of cells to PET woven fabrics used in medicine. No significant change, unlike *O*‐glycans	Hu, Sheng, et al. (2022)
*Leptinotarsa decemlineata* (Colorado potato beetle), peritrophic membrane	PNGase A, FT‐ICR (**DABP**), (per‐Me)	Study of changes to peritrophic membrane in *mannosidase‐Ia* silenced insects. Accumulation of high‐mannose glycans	Liu, De Schutter, et al. (2022)
Madin‐Darby canine kidney (MDCK) cells and humanized MDCK cell line	PNGase F, TOF/TOF (**DHB**), (per‐Me)	Analysis of sialylated and sulfated glycans in humanized cell line	Byrd‑Leotis et al. (2022)
MDCK sialic acid knockout cells	PNGase F, QIT‐TOF (**DHB**), (linkage‐specific amidation, 2‐AA)	Investigation of influenza A virus agnostic receptor tropism with terminal sialic acid knockout cells	Kamiki et al. (2022)
Middle silk gland of silkworm (*Bombyx mori*)	Hydrazinolysis, *R*‐TOF (**DHB**), (2‐AP)	Temporal analysis of *N*‐acetylglucosamine extension of *N*‐glycans	Kajiura et al. (2022)
Mouse liver	PNGase F, TOF (**DHB**), (per‐Me)	Nuclear receptors (farnesoid X receptor and small heterodimer partner) found to regulate protein *N*‐glycan modifications in the liver	Mathur et al. (2021)
Mouse serum	PNGase F, TOF/TOF (**DHB**), (per‐Me)	Identification of glycans separated by molecular matrix electrophoresis	Liu, Liu, Li, et al. (2021)
Mouse serum	PNGase F, TOF/TOF (**CHCA**), (Me ester, BOA)	Study of anxiety‐related behaviors in single versus group‐housed male mice	Abou‐Elnaga et al. (2021)
Mouse brain tissue	PNGase F, TOF/TOF (**DHB**), (per‐Me)	Brain glycoproteins shown to exhibit diminished glycan complexity compared to other tissues	Williams et al. (2022)
Mouse peritoneal macrophage sub‐populations	PNGase F, TOF/TOF (**DHB**), (per‐Me), MS/MS	Resident and elicited murine macrophages shown to differ in expression of their glycomes and glycan‐binding proteins	Park, Chen, et al. (2021)
Mouse primary mesangial cell	PNGase F, FT‐ICR (**CHCA**, TM sprayer on glass slides)	Increased sialylation in lupus	Sundararaj et al. (2021)
NK Cells	PNGase F, TOF	Glycoengineering of NK cells with glycan ligands of CD22 and selectins for B‐Cell lymphoma therapy	Hong et al. (2021)
*Oryzias latipes*, (Japanese medaka, fish)	Hydrazine, TOF (**DHB**), (2‐AP)	Exposure of silver nanocolloids from environmental pollution shown to cause glycosylation disorders and embryonic deformities in medaka fish	Shimizu et al. (2021)
*Oryzias latipes* (Japanese medaka, fish)	Hydrazine, QIT‐TOF (**DHB**), (2‐AP)	Exposure of TiO_2_ nanoparticles from environmental pollution shown to cause glycosylation disorders and embryonic deformities in medaka fish	Horiuchi et al. (2021)
*Pelagia noctiluca* (jellyfish), mucus	PNGase F, *R*‐TOF/TOF (**DHB**), glycoblotting	High‐mannose. Use for accumulation of nanoparticles	Patwa et al. (2022)
*Phaseolus lunatus* beans	*R*‐TOF/TOF (**DHB**), (procainamide)	Investigation of the use of sodium hypochlorite to release Man_9_GlcNAc_2_	Diaz et al. (2022)
Porcine bladder urothelial cells	PNGase F, FT‐ICR (**DHB**), (per‐Me)	Structural identification (high‐mannose, hybrid, di‐, tri‐, tetra‐antennary complex)	Wang, Bergström, et al. (2022)
Porcine endometrium	PNGase F, TOF/TOF (**DHB**)	Glycomics reveal that ST6GAL1‐mediated sialylation regulates uterine lumen closure during implantation	Han, Wang, et al. (2022)
*Thorsmoerkia curvula* gen. et spec. nov. (semi‑terrestrial microalga from Iceland	Pepsin, PNGase A, TOF (**DHB**), (per‐Me)	High‐mannose (Man_2‐9_) plus series with one deoxy‐hexose of one pentose.	Nicoletti et al. (2021)
Umbilical mesenchymal stem cells	Trypsin (release method not stated), QIT‐TOF (**DHB**), (per‐Me), database analysis	Proteomics and posttranslational modifications analysis during aging (Dubious high mannose structures)	Wang, Zhao, Chen, et al. (2022)

^a^ Format (not all items present): Glycan release method and/or protease, MALDI method (**matrix**), (derivative), other methods.

#### 13.3.4 *O*‐linked glycans

Analysis of *O*‐linked glycans has received much less attention than that of *N*‐glycans. Although generally smaller, they do not have the conserved core of *N*‐glycans and enzymatic release suffers from a lack of suitable enzymes. β‐Elimination is, thus, the preferred method for their release. Two recent reviews are of interest “Recent advances in demystifying *O*‐glycosylation in health and disease” (116 references) (Li, Guo, et al., 2022) and “Quantitative characterization of *O*‐GalNAc glycosylation” (51 references) (Čaval et al., 2021).

Recently, several *O*‐glycan‐specific endoproteases that can cleave the protein adjacent to the appended glycan have been described and used for the analysis of these compounds. To date, use of most of these enzymes suffer from problems such as inefficient cleavage of glycoproteins bearing sialylated *O*‐glycans, high selectivity for certain types of glycoproteins, or protein sequence bias. Vainauskas et al. (2022) have investigated a new immunomodulating metalloprotease from *Pseudomonas aeruginosa* using an array of synthetic peptides and their glycoforms. They showed that the enzyme has no specific residue preference and can tolerate most amino acids, except aspartic acid, at the position adjacent to the glycosylation site on the amino‐terminal side of the peptide. The enzyme was found not to cleave between two adjacent *O*‐glycosites. Glycopeptides with as few as two amino acids on either side of an *O*‐glycosite could be cleaved and the enzyme efficiently cleaved peptides and proteins carrying sialylated and asialylated *O*‐glycans.

A new method for identification of *O*‐GlcNAc‐modified proteins involves intracellular expression of a soluble GalNAc transferase, which then labels the GlcNAc residues with GalNAc. The resulting disaccharides can be detected by *Wisteria japonica* agglutinin, which is specific for this disaccharide, or by MALDI‐TOF MS (Abo et al., 2022).

##### 13.3.4.1 Site analysis

Two protocols for site analysis of *O*‐glycans have been published: “Mapping *O*‐glycosylation sites using the *O*‐specific protease OpeRATOR and LC‐MS” (Nordgren et al., 2021) and “Site‐specific *O*‐glycosylation analysis by liquid chromatography–mass spectrometry with electron‐transfer/higher‐energy collisional dissociation” (Hashii & Suzuki, 2021).

##### 13.3.4.2 Other studies

A protocol named Cellular *O*‐Glycome Reporter/Amplification (CORA) for studying mucin‐type *O*‐glycans of living cells has been developed by Kudelka et al. (2021). It involves incubation of cells with peracetylated Bn‐α‐GalNAc or N_3_‐Bn‐α‐GalNAc where cytosolic esterases generate the deacylated Bn‐α‐GalNAc or N_3_‐Bn‐α‐GalNAc. These glycans are transported into the Golgi apparatus and modified by native *O*‐glycosyltransferases which are secreted into the medium, purified, and then analyzed by MALDI‐TOF MS (from DHB) for Bn‐*O*‐glycans or derivatized with fluorescent tag 2‐amino‐*N*‐(prop‐2‐yn‐1‐yl)benzamide (PYAB, **210**) followed by HPLC separation. The consequent amplification and secretion of the *O*‐glycome products was claimed to greatly facilitate their analysis and to aid functional studies.


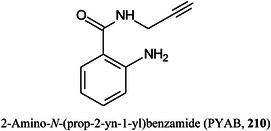


Further applications of the use of MALDI for the analysis of *O*‐glycans in specific glycoproteins and tissues are listed in Tables 22 and 23 respectively.

**Table 22:** Use of matrix‐assisted laser desorption/ionization‐mass spectrometry for examination of *O*‐glycans from specific glycopeptides.

Glycoprotein and source	Methods^a^	Notes	References
Vλ6 light chain mutant Wil, recombinant in *Pichia pastoris*	Trypsin, TOF/TOF (**SA**), glycopeptides	Effect of *O*‐glycosylation on amyloid fibril formation of the variable domain	Abe et al. (2021)
Apolipoprotein CIII	FT‐ICR (**CHCA, SA**)	Structural characterization	Demus et al. (2021)
Asprosin (from serum)	TOF/TOF (**CHCA**), (per‐Me)	Asprosin detection in clinical samples reveals serum/saliva correlation and indicates cartilage as source for serum asprosin	Morcos et al. (2022)
SARS‐CoV‑2 Receptor‐binding domain (RDB)	TOF (**s‐DHB**)	Structural and functional characterization of SARS‐CoV‑2 RBD domains produced in mammalian cells	Gstöttner, Zhang, et al. (2021)
Major peptide from the male ejaculatory duct of *Rosophila melanogaster*	*R*‐TOF/TOF (**DHB**)	Unspecified structure with one hexose, HexNAc and phosphoethanolamine	Sturm et al. (2021)
MUC2 from mouse small intestine and colon	β‐Elimination, TOF/TOF, (per‐Me)	The role of the mucin‐glycan foraging *Ruminococcus gnavus* in communication between the gut and the brain. Changes in sialylation detected	Coletto et al. (2022)
Mucins from murine submandibular glands	β‐Elimination, *R*‐TOF/TOF, QIT‐TOF (**DHB**), (per‐Me)	Study of the effect of aging on mucins	Kameyama, Tin, et al. (2021)
NOTCH1 EGF repeat fragments	*L*‐TOF/TOF (**DHB**), (glycoprotein)	Study of *O*‐GlcNAcylation of NOTCH1	Tsukamoto et al. (2022)
Porcine gastric mucin	β‐Elimination, TOF (per‐Me)	The human gut symbiont *Ruminococcus gnavus* shows specificity to blood group A antigen during mucin glycan foraging	Wu, Crost, et al. (2021)
SARSCoV‑2 Spike glycoprotein (HEK293 and baculovirus‐insect cells)	β‐Elimination, TOF/TOF (**DHB**), (per‐Me)	Site‐specific analysis	Wang, Wu, et al. (2021)
SARS‐CoV‑2 nucleocapsid protein in HEK293 cells	β‐Elimination, TOF/TOF (**DHB**), (per‐Me)	Structural characterization	Supekar et al. (2021)
Thrombospondin‐1	TOF, glycoprotein	*O*‐Fucosylation shown to stabilize the TSR3 motif in thrombospondin‐1 by interacting with nearby amino acids and protecting a disulfide bond	Berardinelli et al. (2022)
Various glycoproteins	β‐Elimination, TOF/TOF, (per‐Me)	To identify *O*‐glycans in new method for on‐line LC‐MS/MS identification of *O*‐glycosites by EThcD	Yang, Wang, et al. (2021)
Visgun (protein) from *Drosophila melanogaster* S2R+ cells	β‐Elimination, TOF/TOF (**DHB**)	Identification of Visgun as a Tc toxin receptor	Xu, Viswanatha, et al. (2022)

^a^ Format (not all items present): Glycan release method and/or protease, MALDI method (**matrix**), compounds run (derivative).

**Table 23:** Use of matrix‐assisted laser desorption/ionization‐mass spectrometry for examination of *O*‐glycans from intact organisms or tissues.

Organism	Methods^a^	Notes	References
Bovine submaxillary mucin	β‐Elimination, FT‐ICR (**s‐DHB**), (per‐Me)	As reference for development of automated method	Kotsias et al. (2021)
CHO Cell lines (CHO‐K1, CHO‐S, and CHO‐Pro5)	β‐Elimination, *R*‐TOF/TOF, (per‐Me)	Structural determination. Differences in sialylation and fucosylation	Wang, Wang, Wu, Lin, et al. (2022)
Human dermal endothelial cells	Glycans from cell medium, TOF/TOF (**DHB**), (per‐Me)	Sialoglycans on lymphatic endothelial cells shown to augment interactions with Siglec‐1 (CD169) of lymph node macrophages	D'Addio et al. (2021)
Human (serum and cerebrospinal fluid)	β‐Elimination, *R*‐TOF/TOF	Detection of novel low‐molecular‐weight blood group‐specific glycans in serum and cerebrospinal fluid	Furukawa et al. (2021)
Human umbellar vein endothelial cells	TOF (per‐Me)	To assess adhesion of cells to PET woven fabrics used in medicine. Profile changed, unlike *N*‐glycans	Hu, Sheng, et al. (2022)
Mouse brain	β‐Elimination, QIT‐TOF (**DHB**), amidation of sialic acids	Majority of α−2,6‐sialylated glycans in the adult mouse brain shown to exist in *O*‐glycans	Ohmi et al. (2021)
Mouse bain tissue	β‐Elimination, TOF/TOF (**DHB**), (per‐Me)	Brain glycoproteins shown to exhibit diminished glycan complexity compared to other tissues (most unbranched)	Williams et al. (2022)
Mouse serum	β‐Elimination, TOF/TOF (**DHB**), (per‐Me)	Identification of glycans separated by supported molecular matrix electrophoresis. α2,8‐Sialylated *O*‐glycans detected	Liu, Liu, Li, et al. (2021)
Mouse (submandibular gland)	β‐Elimination, *R*‐TOF/TOF, QIT‐TOF (**DHB**), (per‐Me)	Protein Bmi‐1 shown to regulate mucin levels and mucin *O*‐glycosylation	Kameyama, Nishijima, et al. (2021)
Mouse embryonic stem cell	Trypsin (TOF/TOF)	Identification of *O*‐GlcNAcylation of proteasome activator subunit 3 (Psme3) protein	Pecori et al. (2021)
Mouse peritoneal macrophage subpopulations	β‐Elimination, TOF/TOF (**DHB**), (per‐Me), MS/MS	Resident and elicited murine macrophages shown to differ in expression of their glycomes and glycan‐binding proteins	Park, Chen, et al. (2021)
*Pelagia noctiluca* (jellyfish), mucus	*R*‐TOF/TOF (**DHB**), glycoblotting	Use for accumulation of nanoparticles	Patwa et al. (2022)
Porcine bladder urothelial cells	β‐Elimination, FT‐ICR (**DHB**), (per‐Me)	Structural identification	Wang, Bergström, et al. (2022)
*Pseudomonas aeruginosa*	Non‐reductive β‐elimination, *R*‐TOF/TOF, (per‐Me)	Mucin glycans shown to signal through the sensor kinase RetS to inhibit virulence‐associated traits	Wang, Wheeler, et al. (2021)
*Schmidtea mediterranea* (flatworm)	β‐Elimination, TOF/TOF (**s‐DHB**), (per‐Me and free)	Structural determination of mucin‐type *O*‐glycans	Subramanian et al. (2022)
*Tribolium castaneum* (Insect)	β‐Elimination, FT‐ICR (**s‐DHB**), (per‐Me)	Pentasaccharide mucin‐type *O*‐glycans shown to be linked with pupation	Li, De Schutter, et al. (2022)

^a^ Format (not all items present): Glycan release method and/or protease, MALDI method (**matrix**), (derivative), other methods.

### 13.4 Glycated proteins (nonenzymatic attachment of sugars)

**Scheme 20:**
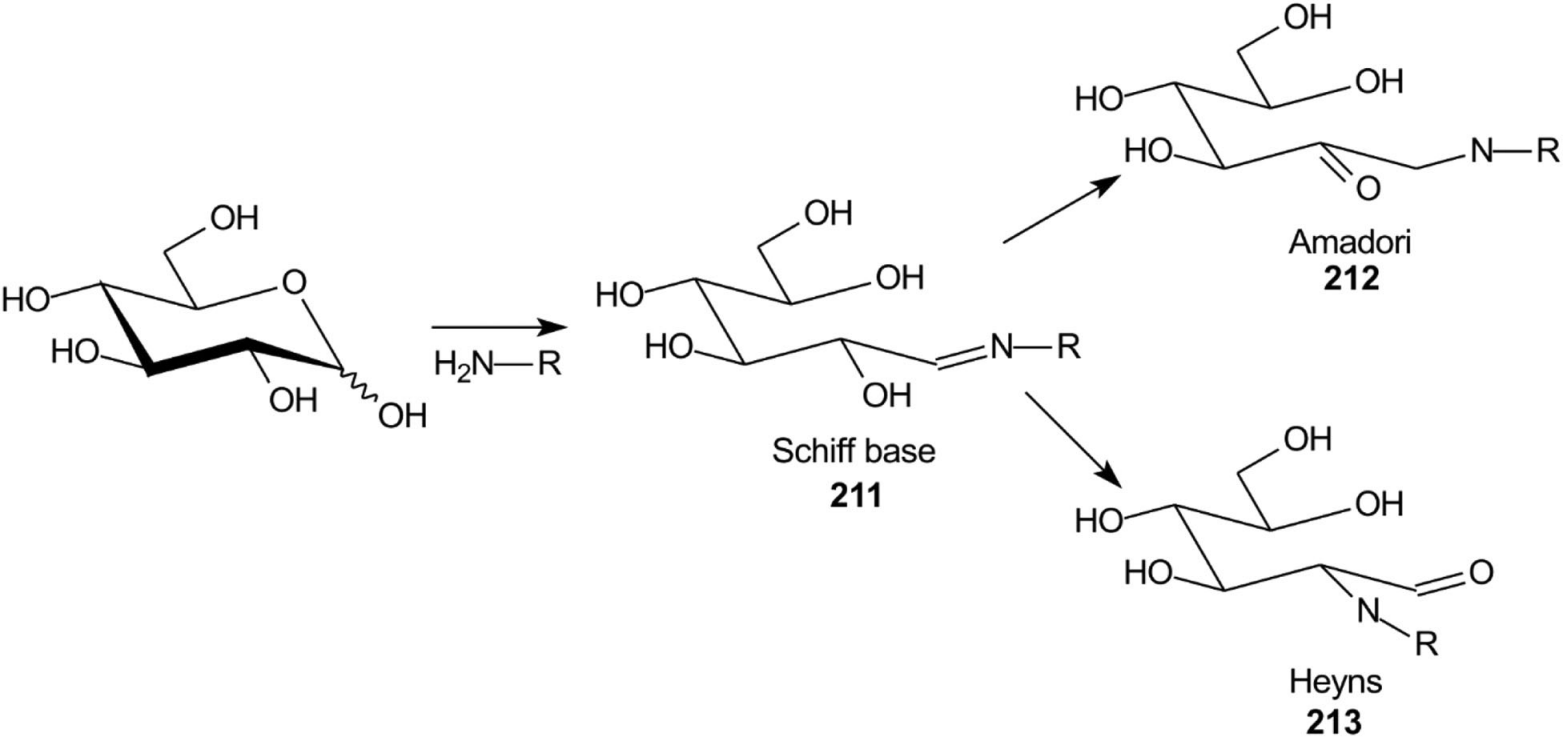
Formation of products resulting from glycation of proteins.

Review: “Enrichment and analysis of glycated proteins” (Cho, Duong, et al., 2022), 126 references

d‐Glucose and d‐fructose react with amino groups in proteins to form Schiff bases that rearrange to more stable Amadori and Heyns products, respectively (Scheme 20). These compounds are difficult to differentiate by mass spectrometry because of their identical molecular masses and similar fragmentation patterns. However, it has now been shown that separation can be achieved by RP‐HPLC with phosphate‐buffered eluants providing the best separation (Schmutzler & Hoffmann, 2022).

Glycated hemoglobin (HbA1c) is used to monitor patients with diabetes but results can differ depending on the method used. Song, Xu, et al. (2022) have compared LC/MS and MALDI‐TOF to monitor this glycated protein and generally found good agreement between the two techniques. Variations were, however, found where variant hemoglobin was encountered.

Other applications of MALDI to the analysis if glycated proteins are listed in Table 24.

**Table 24:** Use of matrix‐assisted laser desorption/ionization‐mass spectrometry for the investigation of glycated proteins.

Protein/amino acid and sugar	Methods^a^	Notes	References
Alpha‐synuclein, methylglyoxal	TOF/TOF (**DHA**, NH_4_Cit)	Glycation shown to modulate alpha‐synuclein fibrillization kinetics	Farzadfard et al. (2022)
BSA, glucose	MALDI (**DHB**/**SA**)	Development of a benzothiazole‐phenothiazine conjugate‐based molecular probe for the differential detection of glycated albumin	Kumar et al. (2021)
BSA, glucose, fructose, methylglyoxal	TOF/TOF (**SA**)	*In vitro* chronic glycation shown to induce AGEs accumulation reducing insulin‐stimulated glucose uptake and increasing glucagon‐like peptide 1 (GLP1R) in adipocytes	Chilelli et al. (2021)
Hemoglobin, methylglyoxal	TOF	Methylglyoxal‐derived hemoglobin advanced glycation end products shown to induce apoptosis and oxidative stress in human umbilical vein endothelial cells	Lee, Samsuzzaman, et al. (2021)
HSA, d‐glucose	TOF	Effects of glycation in the binding of bioactive flavonoid 6‐hydroxyflavone by HSA	Sarmah et al. (2022)
HSA, d‐glucose	TOF/TOF (**SA**, **CHCA**, **DHB**)	Investigation of the effects of glycation on drug binding to HSA	Ghosh and Kishore (2022)
Murine cardiac proteins, fructose	TOF	Curcumin shown to prevent glycation of tricarboxylic acid cycle and cell respiration proteins in the hearts of mice fed with a high‐fructose diet	León‐García et al. (2022)
Myoglobin, glyoxal	TOF/TOF (**CHCA**)	Long‐term incubation of myoglobin with glyoxal shown to induce amyloid like aggregation of the heme protein	Banerjee (2021b)
Myoglobin, melibiose	MALDI	The melibiose‑derived glycation product shown to mimic a unique epitope present in human and animal tissues	Staniszewska et al. (2021)
Myoglobin, methylglyoxal	TOF/TOF (**CHCA**)	Role of advanced glycation end products in inducing protein structural alterations	Banerjee (2021a)

^a^ Format (not all items present): MALDI method (**matrix**).

### 13.5 Peptidoglycans

Peptidoglycans are found in the cell walls of most bacteria and are composed of chains of GlcNAc‐β‐(1→4)‐MurNAc (where MurNAc = **214**) cross‐linked by small peptides, commonly l‐Ala‐γ‐d‐Glu‐meso‐A_2_pm(or l‐Lys)‐d‐Ala‐. Their analysis usually involves hydrolysis to amino acids, peptides and amino sugars or enzymatic digestion of the glycan chain to muropeptides (disaccharide plus peptide).


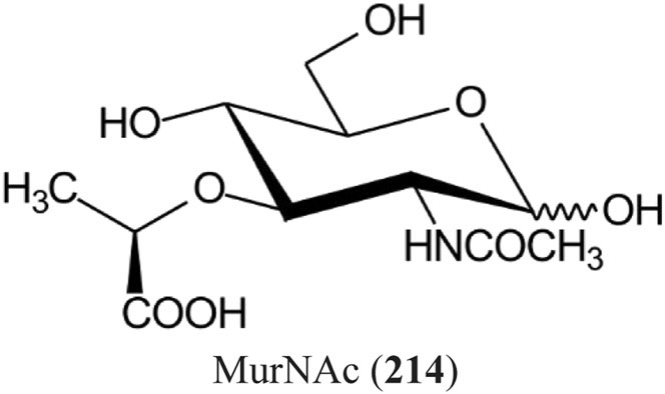


Papers describing work with these compounds are listed in Table 25.

**Table 25:** Use of matrix‐assisted laser desorption/ionization‐mass spectrometry for examination of bacterial peptidoglycans and muropeptides.

Species	Peptidoglycan	Methods	Notes	References
*Acinetobacter baumannii*	Muropeptides	TOF	The bacterium is shown to be able to survive with an outer membrane lacking lipooligosaccharide due to structural support from elongasome peptidoglycan synthesis	Simpson et al. (2021)
*Bacillus subtilis mreB* mutants	Muropeptides	TOF	Magnesium shown to restore the rod shape of *Bacillus subtilis mreB* mutants through its inhibitory effect on peptidoglycan hydrolases	Tesson et al. (2022)
*Vibrio cholerae*	D‐Met‐and D‐Arg‐muropeptides	TOF/TOF	Binding of noncanonical peptidoglycan shown to control *V. cholerae* broad spectrum racemase activity	Espaillat et al. (2021)

### 13.6 Glycosaminoglycans (GAGS)

Reviews and general articles on the analysis of glycosaminoglycans are listed in Table 26.

**Table 26:** Reviews and general articles on the analysis of glycosaminoglycans.

Subject	Comments	Citations	References
Developments in mass spectrometry for glycosaminoglycan analysis	Covers sample preparation, composition analysis, sequencing (fragmentation), software and applications	165	Pepi, Sanderson, et al. (2021)
Insights into structure, affinity, specificity, and function of GAG‐protein interactions through the chemoenzymatic preparation of defined sulfated oligohyaluronans	Useful short section on analysis of GAGs by mass spectrometry	32	Schiller et al. (2021)
Analysis of the glycosaminoglycan chains of proteoglycans	Fairly comprehensive. Sample preparation, MS (mainly LC‐MS, NMR, hyphenated techniques).	107	Song et al. (2021)
State‐of‐the‐art glycosaminoglycan characterization	Comprehensive review covering structure, function, sample preparation, chromatographic and mass spectrometric analytical methods, ion mobility, IR spectroscopy	352	Zappe et al. (2022)

A problem with analysis of these compounds by MALDI is loss of sulfate. A new method, reported Krüger et al. (2022) involves on‐target derivatization with 3‐hydrazinobenzoic acid (3‐HBA, **215**) by heating at 70^o^C for 10 min. MALDI‐TOF/TOF was performed in negative ion mode. Disaccharides from dermatan (**216**) and chondroitin sulfates (**217**) were examined and MS/MS spectra allowed sulfation patterns to be resolved.


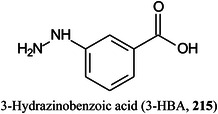



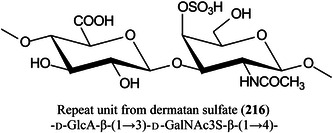



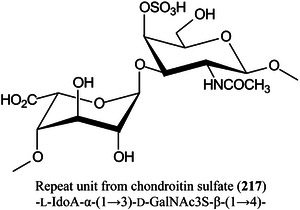


A detailed study of the photofragmentation of chondroitin sulfate isomers has shown promising results, particularly with differentiating isomers involving C‐5 uronic acid stereochemistry (Pepi, Leach, et al., 2021).

### 13.7 Glycolipids

Several types of glycolipid can be identified. They include lipopolysaccharides (LPS) found in the cell membranes of Gram negative bacteria, glycosphingolipids (GSLs) and a range of assorted structures, usually found in bacteria. Several general reviews are of interest. A review of recommendations for good practice in MS‐based lipidomics, while not providing a step‐by‐step protocol for best practice, nevertheless provides the reader with links to original publications concerning the state‐of‐the‐art practices in the field (Köfeler et al., 2021). MALDI methods, however, are specifically excluded because the authors state that the technique is overwhelmingly used for MALDI imaging which is beyond the scope of the review.

“Imaging lipids in biological samples with surface‐assisted laser desorption/ionization mass spectrometry” (SALDI‐MSI) with 169 references, is a concise review of work published during the last decade (Müller, De Pauw, et al., 2021) (**169**). The review describes the advantages of SALDI‐MSI for lipid analysis, such as the ability to perform analyses in both ionization modes with the same nanosubstrate, and the detection of lipids that exhibit low ionization efficiency in MALDI‐MS. The complementarity of SALDI and MALDI‐MSI is also discussed. The review contains a very comprehensive list of the use of SALDI in lipid and glycolipid analysis.

The third review “A new update of MALDI‐TOF mass spectrometry in lipid research” (361 references) (Engel et al., 2022), is a general review of lipids and glycolipids covering work over the past 10 years, with emphasis on glycerophospholipids. Particular attention is given to quantitative aspects of MALDI MS since this is widely considered as the most serious drawback of the technique. The choice of the MALDI matrix is shown to be crucial to be able to detect all lipid classes. MALDI imaging and the combination of MALDI with TLC are given special attention.

Other reviews are listed in Table 27.

**Table 27:** Reviews on glycolipids.

Subject	Notes	Citations	References
A journey from structure to function of bacterial lipopolysaccharides	General review. Structural identification of LPS. Comments on unusual monosaccharides	284	Di Lorenzo et al. (2022)
Lipopolysaccharide lipid A: A promising molecule for new immunity‐based therapies and antibiotics	Emphasises the dominance of MALDI as the best analytical method for lipid A with examples	240	Garcia‐Vello, Di Lorenzo, et al. (2022)
History of colistin resistance mechanisms in bacteria: Progress and challenges	Short section on MALDI analysis of lipid A	110	Hamel et al. (2021)
A comprehensive review on natural occurrence, synthesis and biological activities of glycolipids	Many types of glycolipid. Few references to MALDI	318	Jala et al. (2022)
Integrated mass spectrometry‐based multi‐omics for elucidating mechanisms of bacterial virulence	Mainly proteomics but section on lipid A	276	Man et al. (2021)
Solving the structural puzzle of the bacterial glycome	Short review, mass spectrometry, NMR, bioinformatics	52	Marchetti et al. (2021)
Structures and functions of the gut microbial lipidome	Covers several structural types such as glycoglycerolipids, sphingolopids, lipid A and steroidal glycolipids	157	Morozumi et al. (2022)
Modern techniques for separation, mass spectrometric detection, and characterization of glycolipids	Extraction and purification, TLC, CZE and ion mobility	89	Sarbu and Zamfir (2021)

#### 13.7.1 LPS and lipooligosaccharides (LOS)

These compounds are composed of lipid A, a glycolipid containing two glucosamine molecules attached to up to six fatty acyl chains and decorated with various groups such as phosphate (see structure **219** below for an example), a core region and usually a long carbohydrate chain consisting of repeat units. The term LOS is usually used for the smaller molecules. A protocol “Dissecting lipopolysaccharide composition and structure by GC‐MS and MALDI spectrometry” in *Methods in Molecular Biology* (Garcia‐Vello, Speciale, et al., 2022) describes methods for analysis of these compounds.

Lipid profiles as determined by MALDI‐MS in negative ion mode, combined with a machine‐learning algorithm have proved useful in discriminating between *Escherichia coli*, *Shigella flexneri*, and *S. sonnei*. The three species showed different profiles for cardiolipins (**218**) and bisphosphoryl lipid A with the *Shigella* species demonstrating higher mass peaks for lipid A (Pizzato et al., 2022).

**Figure 5:**
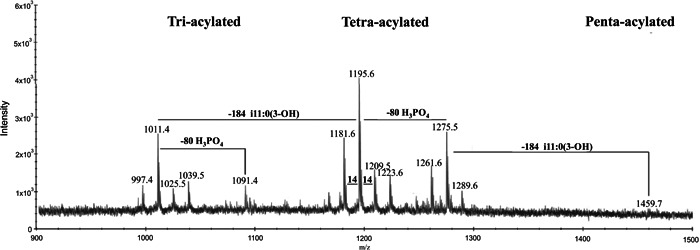
Negative ion MALDI‐TOF spectrum of the LOS from *Idiomarina zobellii* KMM 231^T^. From Kokoulin et al. (2022) with permission from MDPI).


Intact lipooligosaccharide from the deep‐sea marine bacterium *Idiomarina zobellii* KMM 231^T^, isolated at a depth of 4000 m (Kokoulin et al., 2022) has been analysed and shown to consist of only five sugar rings, two of which comprise the lipid A portion (**219**). The negative ion MALDI‐TOF spectrum (Figure 5) resolved three groups of peaks corresponding to tri‐, tetra‐, and penta‐acylated forms with acyl chains of different length. Deacylated LOS was studied by NMR and monosaccharide identification was performed by GC/MS. The lipid A portion of the molecule was isolated and each acylated form was studied by MALDI‐MS/MS.

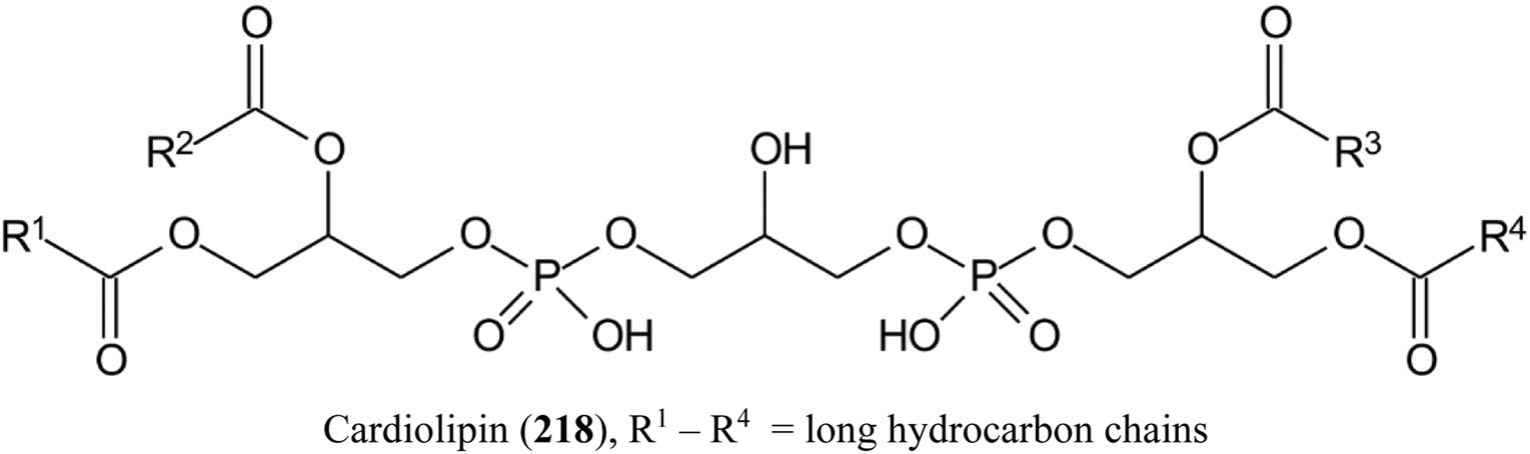



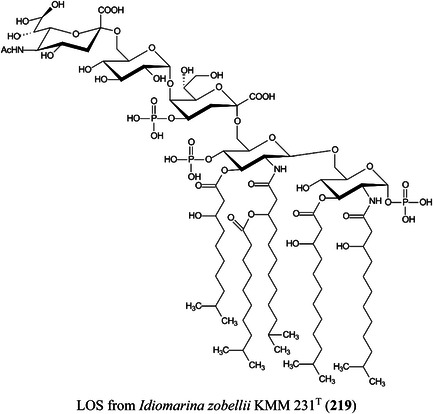


##### 13.7.1.1 Lipid A

**Scheme 21:**
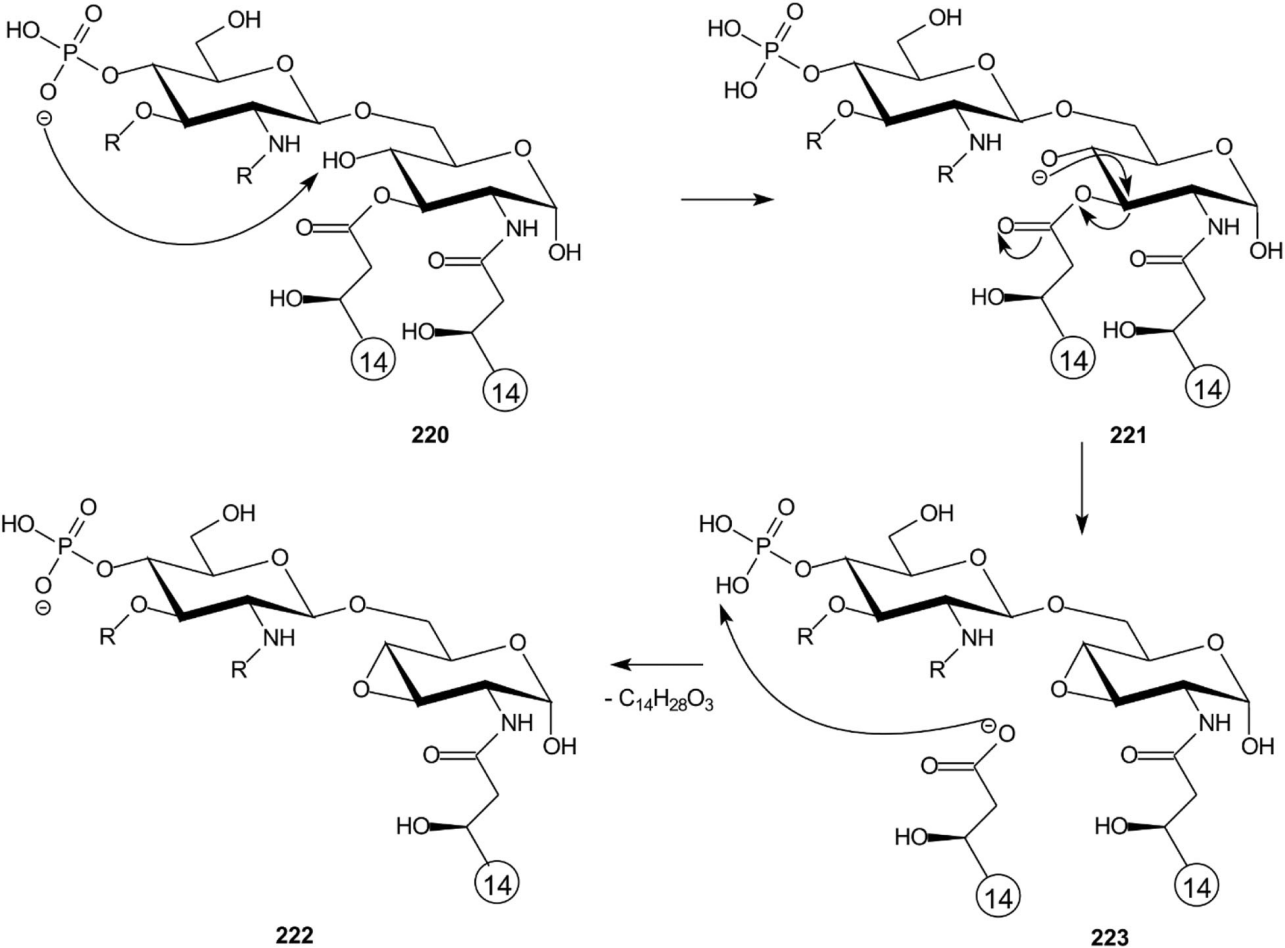
Formation of an epoxide by loss of the C3 primary acid group from the [M – H]^‐^ ion of lipid A.

**Scheme 22:**
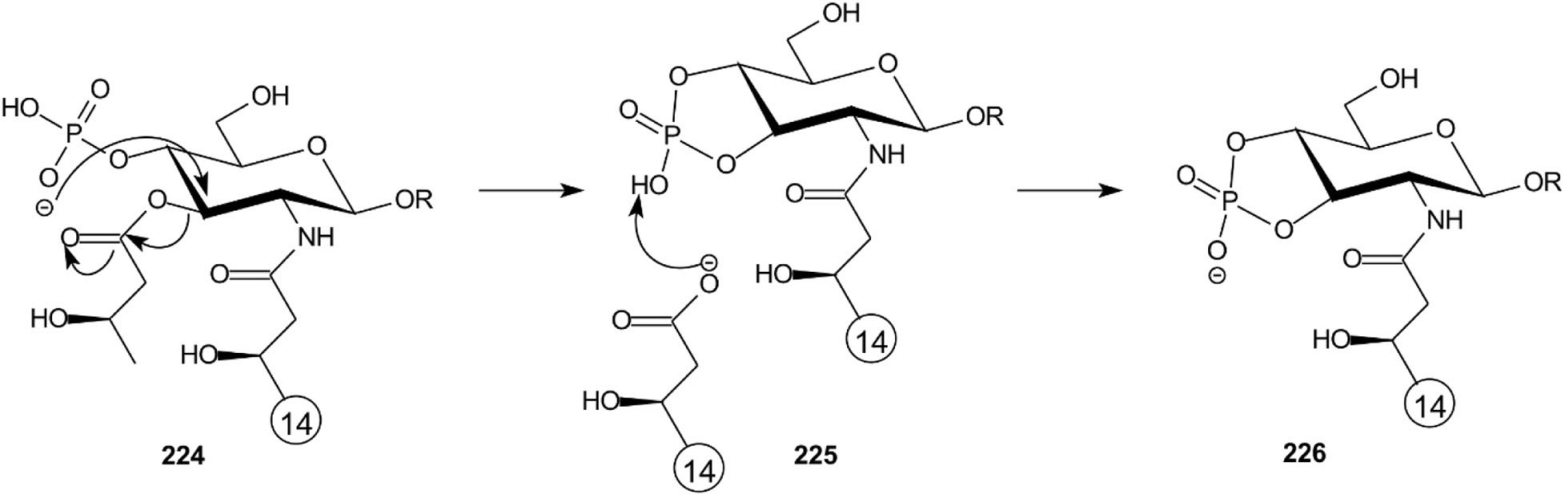
Formation of a cyclic phosphate by loss of the C3’ primary acid group from the [M – H]^‐^ ion of lipid A.

**Scheme 23:**
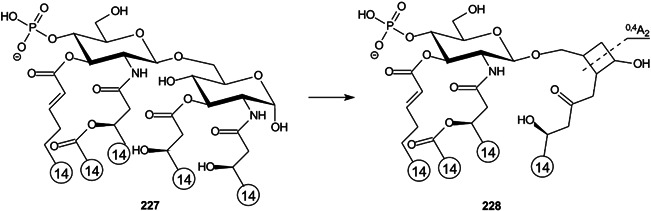
Formation of an oxetanose ring.

Two detailed protocols for analysis of 4‐monophosphoryl lipid A by MALDI‐TOF have been published (Larrouy‐Maumus, 2021; Micoli et al., 2022). Most work was done in negative ion mode because of the anionic groups attached to the GlcN residues. Aissa et al. (2021) have studied the negative ion (deprotonated molecule) CID spectra of lipid A and their findings can be summarised as follows: (i) cleavage of the C‐3 primary fatty acid to leave an epoxide group attached to the reducing sugar (Scheme 21); (ii) cleavage of the C‐3’ primary fatty acid (as an acid) which generates a cyclic phosphate connected to the nonreducing sugar (Scheme 22); (iii) cleavage of the C‐2’ secondary fatty acid which is observed to occur both in acid and ketene forms; (iv) the C‐2 and C‐2’ primary fatty acids are eliminated as an amide and ketene, respectively; (v) the ^0,2^A_2_ cross‐ring fragment from the reducing terminal ring contains a four‐membered ring (oxetanose, Scheme 23); (vi) the ^0,4^A_2_ ion is formed from this ion; and (vii) formations of H_2_PO_4_^‐^ and PO_3_^‐^ ions are associated with the formation of sugar epoxide.

Yang, Smith, Chandler, et al. (2022) have developed a tandem MS version of the earlier fast lipid analysis technique (FLAT) method termed FLAT^*n*^ and used it to directly examine lipid A from a single bacterial colony of *E. coli*. Washed bacteria were deposited onto an indium tin oxide (ITO) slide, treated with citrate buffer and heated at 110^o^C for 30 min and examined with a timsTOF instrument. Detailed spectra of the lipid A, including cross‐ring fragments, were obtained. The method was developed into a direct‐from‐urine diagnostic for Gram‐negative pathogens (Yang, Smith, Sumner, et al., 2022).

The phosphate groups on lipid A are targeted by cationic antimicrobial peptides, such as polymyxins. However, resistance can develop because the bacteria are able to neutralize the negative charges by adding neutral groups such as aminoarabinose (AraN, **229**) and ethanolamine (EtN). These modifications can be detected by MALDI‐TOF but only semiquantitatively. To overcome this disadvantage Sherman et al. (2022) have developed a GC/MS assay for the individual components of lipid A involving hydrolysis and derivatization as methyloxime/trimethylsilyl (TMS) derivatives. Using the method, increase in the abundance of AraN and EtN modifications were observed when resistant *Enterobacter* and *E. coli* strains were grown in the presence of colistin (polymyxin E). Because lipid A modifications serve as indicators of polymyxin resistance in Gram‐negative bacteria, this GC/MS method was claimed to provide an excellent method to monitor polymyxin resistance.


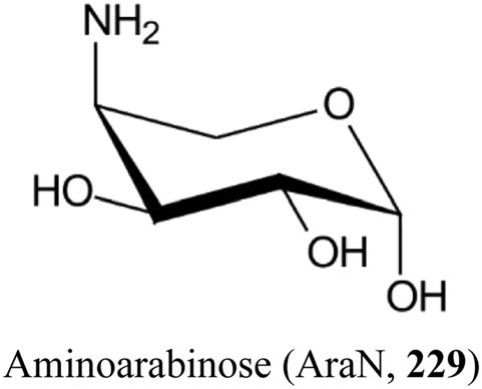


##### 13.7.1.2 O‐Chain

A brief study of the fragmentation pattern of the O‐specific polysaccharide from *Vibrio cholera* O139 has been reported. In negative mode, the predominant fragmentation pathway was loss of neutral monosaccharide residues (Pančík, Pakanová, Mečárová, et al., 2022). A rapid method for measurement of the O‐chain by MALDI‐TOF directly from cells involves heating with HCl for 10 min at 90^o^C (Urakami & Hinou, 2022a) (paper in Japanese).

Table 28 lists work that has been reported with these compounds. Much is targeted in understanding mechanisms of antibiotic resistance and has resulted in the observation of increases phosphorylation of lipid A making it more acidic. As with lipid A, most of the MALDI work has been in negative ion mode with THAP and norharmane as the favoured matrices.

**Table 28:** Use of matrix‐assisted laser desorption/ionization‐mass spectrometry for examination of glycolipids from Gram‐negative bacteria.

Species	Type	Methods^a^	Notes	References
*Acinetobacter baumannii*	Lipid A	*R*‐TOF/TOF (**CMBT‐**EDTA)	Colistin dependence in extensively drug‐resistant *A. baumannii* strain shown to be associated with IS*Ajo*2 and IS*Aba13* insertions and multiple cellular responses	Chamoun et al. (2021)
*A. baumannii*	Lipid A	*R*‐TOF (**CMBT**)	Alteration of lipooligosaccharide structure during cold stress	Herrera et al. (2021)
*A. baumannii*	Lipid A	TOF (**norharmane**, ‐ve)	Overcoming addition of phosphoethanolamine to lipid A mediated colistin resistance in clinical isolates with colistin–sulbactam combination therapy	Srisakul et al. (2022)
*A. baumannii*	Lipid A	TOF (**DHB**)	Phenotypic modification (PEtN incorporation and loss of a C_12_ acyl chain) of lipid A in clinical isolates	Kim, Yun, et al. (2022)
*Aeromonas hydrophila*	Lipid A	TOF (‐ve)	Investigation of colistin resistance	Liu, Xiao, et al. (2021)
*Aeromonas salmonicida*	Lipid A	TOF (‐ve)	Mobile colistin resistance enzyme MCR‐3 shown to facilitate bacterial evasion of host phagocytosis	Yin, Ling, et al. (2021)
*Aeromonas veronii* bv*. sobria* Strain K133	LPS	Q‐TOF (**THAP**)	Structural characterization	Dworaczek et al. (2021)
*Alcaligenes faecalis*	LPS, Lipid A	TOF/TOF (**THAP**)	Complete structure characterization and chemical synthesis of its lipid A	Shimoyama et al. (2021)
*Bacteroides thetaiotaomicron*	LPS	TOF/TOF (**DHB**)	Structural characterization and immunological activity	Pither, Illiano, et al. (2022)
Cattle (rumen microbiome)	Lipid A	*R*‐TOF/TOF (**THAP**, ‐ve)	Lipid A acetylation pattern shown to differ between cows fed on different diets	Sarmikasoglou et al. (2021)
*Caulobacter crescentus*	Lipid A	TOF	lipid A shown to be conditionally dispensable in the absence of ferric uptake regulator *fur* and in the presence of anionic sphingolipids	Zik et al. (2022)
*Echinicola pacifica* KMM 6172^T^ and *E. vietnamensis* KMM 6221^T^	Lipid A	TOF/TOF (**THAP**, **DHB, ‐ve**), MS/MS, GC/MS	Incorporation of GalA and modifications to acyl chains in response to survival in a marine environment	Pither, Mantova, et al. (2021)
*Edwardsiella tarda* PCM 1155	O‐polysaccharide	TOF/TOF (**DHB**)	Structural determination shows the presence of unique β‐l‐Rha*p*NAc3NAc derivative	Kaszowska et al. (2021)
*Enterobacter cloacae*	Lipid A	TOF (**DHB**)	Presence of 2‐hydroxymyristate on endotoxins shown to be associated with death in neonates with *E. cloacae* complex septic shock	Augusto et al. (2021)
*E. cloacae*	Lipid A	TOF (**DHB**, ‐ve)	Characterization of resistance mechanisms of *E. cloacae* complex co‐resistant to carbapenem and colistin	Liu, Fang, et al. (2021)
*Enterobacter* species	Lipid A	TOF (**norharmane**, ‐ve)	Development of a MALDI‐TOF assay for the rapid detection of colistin‐resistant enterobacter species	Smith, McElheny, et al. (2022)
*Escherichia coli*	Lipid A	TOF (**DHB**)	Investigation of colistin resistance	Wan, Xu, et al. (2021)
*E. coli*	Lipid A	TOF (**ATT**, **DHB**)	Diacylglycerol kinase A shown to be essential for polymyxin resistance provided by EptA, MCR‐1, and other lipid A phosphoethanolamine transferases	Purcell et al. (2022)
*E. coli* R1 and K12	Entero‐bacterial common antigen	TOF/TOF (**THAP**, **DHB**)	Structural identification (repeats of →3)‐α‐d‐Fuc*p*4NAc‐(1→4)‐β‐d‐Man*p*NAcA‐(1→4)‐α‐d‐Glc*p*NAc‐(1→ linear and cyclic forms	Gozdziewicz et al. (2021)
*E. coli* harbouring the mcr‐8 (nmcr‐2) mutants	Lipid A	TOF	Characterization of NMCR‐2, a new nonmobile colistin resistance enzyme:	Ullah et al. (2021)
*E. coli* BL21 carrying Ah762 (functional variant of MCR‐3)	Lipid A	TOF	The MCR‐3 inside linker appears as a facilitator of colistin resistance	Xu, Chen, et al. (2021)
*E. coli*	O25B Polysaccharide	Q‐TOF (**DHB**)	Development and characterization of an *E. coli* O25B bioconjugate vaccine	Kowarik et al. (2021)
*E. coli* NK5449.	Lipid A	TOF/TOF (**DHB**)	Study of genes in hospital wastewater breaking through the defence line of last‐resort antibiotics	Zhu, Shuai, et al. (2022)
*Fusobacterium nucleatum* ATCC 51191	Lipid A	*R*‐TOF/TOF (**THAP**, ‐ve)	Structural characterization (and O‐antigen by NMR)	Garcia‐Vello et al. (2021)
*Granulibacter bethesdensis*	Lipid A	*R*‐TOF/TOF (**THAP**, ‐ve)	Bacterium shown to produce a penta‐acylated hypostimulatory glycero‐d‐talo‐oct‐2‐ulosonic acid–lipid A glycolipid (Ko‐lipid A)	Muszyński et al. (2021)
*Herbaspirillum sp*. Root189, isolated from the roots of *Arabidopsis thaliana*	O‐Antigen	FT‐ICR (**s‐DHB**)	LPS O‐antigen molecular and supramolecular modifications of plant root microbiota shown to be pivotal for host recognition	Vanacore et al. (2022)
*Klebsiella pneumoniae*	Lipid A	*L*‐TOF/TOF (**CMBT/**NH_4_‐Cit)	A *K. pneumoniae* DedA family membrane protein shown to be required for colistin resistance and for virulence in wax moth larvae	Tiwari et al. (2021)
*K. pneumoniae*	Lipid A	*R*‐TOF/TOF (**DHB**, –ve)	Pharmacodynamic and immunomodulatory effects of polymyxin B in combination with fosfomycin against KPC‐2‐producing *K. pneumoniae*	Sharma, Garcia, et al. (2022)
*K. pneumoniae*	LPS	TOF	Shown to induce host metabolic stress that promotes tolerance to pulmonary infection	Lung et al. (2022)
*K. pneumonia* and *Acinetobacter baumannii*	Lipid A	TOF (**norharmane**)	Benzimidazole isosteres of salicylanilides shown to be highly active colistin adjuvants. Changes to lipid A monitored by MALDI.	Li, Mattingly, et al. (2021)
*Mycobacterium smegmatis*	LOS	TOF/TOF (**DHB**)	Elimination of enzymes PknL and MSMEG_4242 in *M. smegmati*s shown to alter the character of the outer cell envelope	Báez‐Ramírez et al. (2021)
*Neisseria gonorrhoeae*	LOS/Lipid A	Q‐TOF (**THAP**/nitro‐cellulose)	Investigation of novel small molecules that increase the susceptibility of *N. gonorrhoeae* to cationic antimicrobial peptides by inhibiting lipid A phosphoethanolamine transferase	Mullally et al. (2022)
*Pandoraea pulmonicola*	Lipid A	TOF/TOF (**THAP**, **DHB**, ‐ve)	Chronic strain of the cystic fibrosis pathogen *P. pulmonicola* shown to express a heterogenous hypo‐acylated lipid A	Pither, McClean, et al. (2021)
*Pseudoalteromonas nigrifaciens* Sq02‐Rifr	LOS (**230**)	TOF/TOF (**THAP**), NMR	Complete structural characterization and study of its immunomodulatory activity	Di Guida et al. (2021)
*Pseudomonas aeruginosa*	Lipid A	TOF, (**norharmane**)	Detection of colistin resistance in *Pseudomonas aeruginosa* using the MALDIxin test on the routine MALDI Biotyper Sirius mass spectrometer	Jeannot et al. (2021)
*P. aeruginosa*	Lipid A	TOF (**norharmane**, ‐ve)	Loss of resistance‐nodulation‐division‐type multidrug efflux pumps shown to trigger iron starvation and lipid A modifications	Adamiak et al. (2021)
*P. aeruginosa*	Lipid A	*R*‐TOF (**norharmane**)	Genomic characterization of lytic bacteriophages targeting genetically diverse *P. aeruginosa* clinical isolates	Nordstrom et al. (2022)
*Pseudomonas syringae* pv. *phaseolicola*	Lipid A	TOF (**norharmane**)	Remodelling of lipid A *in vitro*	Gerster et al. (2022)
*Rickettsia* (4 species)	Lipid A	TOF (**norharmane**, ‐ve)	Structural characterization	Guillotte et al. (2021)
*Salmonella enterica* subsp. *enterica* serovar Liverpool	Lipid A	TIMS‐TOF **(9‐AA, ‐**ve)	Coculture with *Acinetobacter johnsonii* shown to enhance resistance to benzalkonium chloride disinfectant by triggering lipid A modifications to reduce net negative charge	Wilson, Fegan, et al. (2022)
*Shigella sonnei*	Lipid A	*L*‐TOF/TOF (**s‐DHB**, ‐ve)	Investigation of the contribution of O‑antigen and proteins to the immunogenicity of *Shigella sonnei* generalized modules for membrane antigens	Mancini et al. (2021)
*S. sonnei*	Entero‐bacterial common antigen	TOF/TOF (**THAP**, **DHB**)	Structural identification (repeats of →3)‐α‐d‐Fuc*p*4NAc‐(1→4)‐β‐d‐Man*p*NAcA‐(1→4)‐α‐d‐Glc*p*NAc‐(1→ linear and cyclic forms	Gozdziewicz et al. (2021)
*Yersinia pestis*	Lipid A	TOF (**nor**harmane, ‐ve)	Optimization of RG1‐VLP vaccine performance in mice with novel TLR4 agonists	Zacharia et al. (2021)
*Zunongwangia profunda* SM‑A87	Lipid A	*R*‐TOF/TOF (**THAP**)	Structural characterization	Pither, Sun, et al. (2022)

^a^ Format (not all items present): MALDI method (**matrix**), other methods.


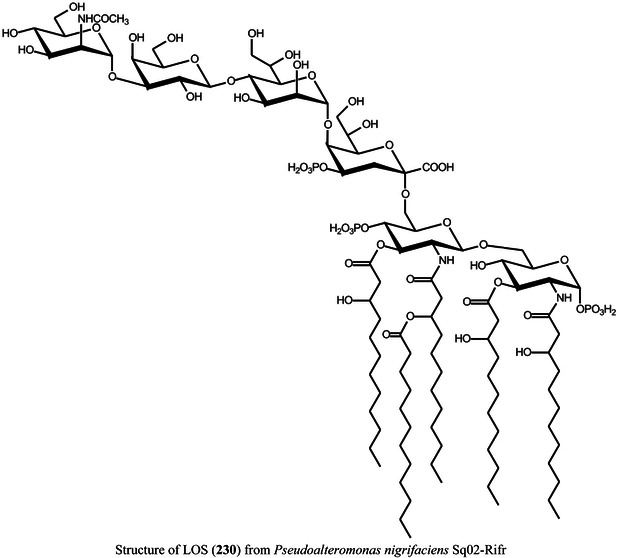


#### 13.7.2 Glycosphingolipids (GSLs)

These compounds consist of the amino alcohol, sphingosine (**231**) in which the amino group is amidated with a long chain saturated or unsaturated fatty acid giving ceramide (Cer) and the primary hydroxyl group is connected to an oligosaccharide chain (see structures **184** and **185**). Du, Yu, et al. (2021) have published a protocol for analysis of glycosphingolipid glycans by lectin microarrays and MALDI‐TOF mass spectrometry. The review “Developments and applications of separation and microfluidics methods coupled to electrospray mass spectrometry in glycomics of nervous system gangliosides” (Sarbu, Ica, & Zamfir, 2021) is relevant and contains references to ion mobility and a few to MALDI. Other reviews are listed in Table 29.

**Table 29:** Reviews and general articles on the analysis of glycosphingolipids (GSLs).

Subject	Comments	Citations	References
Recent progress in *O*‐acetylated gangliosides analysis and functions in cancer	Short discussion on analysis of acylated GSLs by MALDI	88	Groux‐Degroote et al. (2021)
Recognition and avoidance of ion source‐generated artefacts in lipidomics analysis	Fragments generated by ISD. Mainly glycerolipids but some glycosphingolipids mentioned	98	Hu, Luo, et al. (2022)
Glycolipids being viewed *in vivo* or *in vitro*	Review of analytical methods. Small section on MALDI	35	Yilmaz (2021)


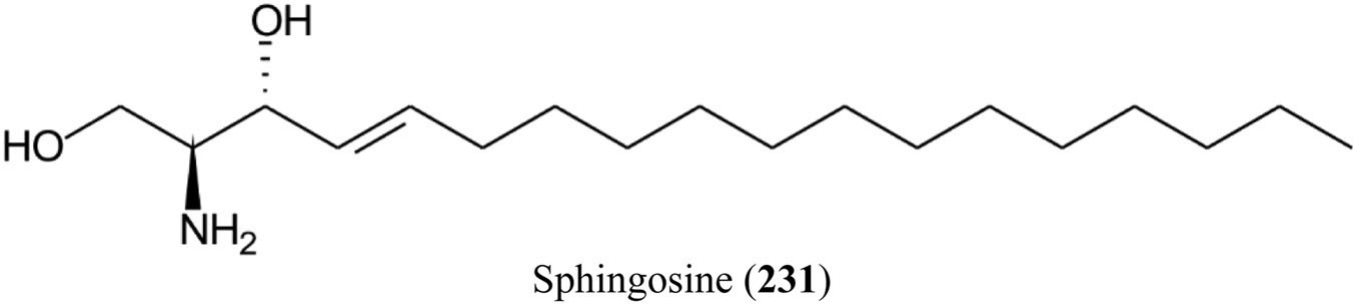


##### 13.7.2.1 Analysis of intact molecules

A simple method for separating GSLs from other lipids, including phospholipids and cholesterol, using zirconium dioxide (zirconia, ZrO_2_) has been developed (Nagasawa et al., 2022). The lipid mixture consisting of GSLs, cholesterol and phospholipids was loaded onto a ZrO_2_ column where cholesterol did not bind. The column was eluted with DHB in methanol when GSLs but not phospholipids were recovered; leaving the phospholipids bound to the ZrO_2_ particles. This method worked well for GSLs such as triglycosylceramides, tetraglycosylceramides and some pentaglycosylceramides, sulfatide (**48**) and GM3 (**232**) located in the lower phase of a Folch's partition, where significant amounts of phospholipids, cholesterol and neutral lipids were found along with GSLs.


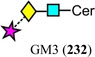


A method involving AP‐MALDI interfaced to an Orbitrap mass spectrometer with a THAP matrix spiked with lithium salts has given improved detection of lipids, particularly HexCer from enveloped viruses (Tran, Monreal, et al., 2021). Use of the method resulted in the identification of over 130 lipids from influenza A virions.

Analysis of sphingo‐ and glycosphingo‐lipids in complex mixtures is greatly facilitated by using basic hydrolysis to remove contaminating glycero‐ and phospho‐lipids. KOH is traditionally used for this purpose leading to the lipids being detected as potassium adducts. Tran, Wan, et al. (2021) have reported that LiOH hydrolysis gives better detection of ceramides and glycoceramides and results in Li adducts of the lipids in the resulting MALDI spectrum. They have, consequently, developed a method using LiOH and have found that THAP provides the best signals. The method was applied to sphingolipid detection from a high‐fat‐induced obesity mouse model.

Use of thin‐layer chromatography (TLC) plates with or without blotting onto hydrophilic polyvinylidene fluoride (PVDF) membranes is frequently used in work with these compounds. A problem can arise from background peaks in the MALDI spectrum that often mask those from the sample. Matsushita et al. (2021) have addressed this problem by pre‐washing the plates with 1,2‐dichloroethane before development and found that the background peaks could successfully be removed.

Positive ion spectra of gangliosides are often weak because of the presence of acidic groups. As with *N*‐glycans, this situation has been reversed by derivatization to block the negative charge sites. Liu, Yang, Li, et al. (2022) have used 1,1‐dimethylethylenediamine (DMEN, **119** above) as the derivatizing reagent which, not only formed amides with the carboxylic acid groups, but also provided a site that was easy to protonate for high sensitivity, The detection of gangliosides was reported to be improved by at least four fold. By using DMEN derivatization, 45 glycosphingolipids were identified from human plasma, including 30 gangliosides and 15 neutral glycosphingolipids.

##### 13.7.2.2 Analysis of released glycans

Because of the heterogeneity of the ceramide, some investigators remove this part of the molecule to study the glycan portion. Endoglycoceramidase I is a suitable enzyme and was employed by Furukawa et al. (2021) in a study of blood group‐specific antigens in serum/plasma and cerebrospinal fluid (CSF). The results suggest that blood group‐specific antigens are predominantly present on GSLs and lipoproteins rather than on glycoproteins. Petralia, van Diepen, et al. (2022) have used the enzyme in a study of the glycome from the filarial nematode *B. malayi* where it was shown that the GSLs contained both unusual glucuronic acid and phosphorylcholine (PC).

Other applications are listed in Table 30.

**Table 30:** Use of matrix‐assisted laser desorption/ionization‐mass spectrometry for examination of glycosphingolipids.

Source	Methods^a^	Notes	References
Human serum and cerebrospinal fluid	Endoglucoceramidase, *R*‐TOF/TOF, Me‐ester	Detection of novel low‐molecular‐weight blood group‐specific glycans in serum and cerebrospinal fluid	Furukawa et al. (2021)
Mouse kidney	Endoglucoceramidase, *R*‐TOF/TOF (aoWR)	GM3 shown to prevent albuminuria and podocytopathy induced by anti‑nephrin antibody	Kawashima et al. (2022)
*Brugia malayi* (filarial nematode)	Endoglycoceramidase, TOF/TOF (**DHB**), (2‐AA), exoglycosidase	Identification of glycans with GlcA and phosphatidylchloline	Petralia, van Diepen, et al. (2022)
Human cervical cancer cells	*R*‐TOF/TOF (**DHB**)	GRASP55 protein shown to regulate intra‐Golgi localization of glycosylation enzymes to control glycosphingolipid biosynthesis	Pothukuchi et al. (2021)
Cervical and prostate cancer cells	TOF/TOF (**DHB**), intact GSLs	Golgi maturation‐dependent glycoenzyme recycling shown to control GSL biosynthesis and cell growth *via* the Golgi‐localised oncoprotein GOLPH3	Rizzo et al. (2021)
*Aspergillus fumigatus* glycoinositol‐phosphoceramides	*R*‐TOF/TOF (**ATT**, ‐ve)	Characterization of a gene cluster involved in *A. fumigatus* zwitterionic glycosphingolipid synthesis	Seegers et al. (2022)

^a^ Format (not all items present): Glycan cleavage, MALDI method (**matrix**), compounds studied, (derivative) other methods.

#### 13.7.3 Bacterial glycolipids

A number of diverse structures are included under this heading and relevant papers are listed in Table 31.

**Table 31:** Use of matrix‐assisted laser desorption/ionization‐mass spectrometry for examination of glycolipids from bacteria, plants and similar organisms.

Source	Glycolipid	Methods^a^	Notes	References
*Bifidobacterium animalis* subsp. lactis BPL1	Lipoteichoic acid (**233**)	*R*‐TOF/TOF (**CHCA**)	Shown to reduce fat deposition *via* the IGF‐1 pathway	Balaguer et al. (2022)
Castor oil	Mannosylerythritol lipids (e.g., **234**)	TOF/TOF	Structural characterization	Beck et al. (2021)
*Mycobacterium abscessus*	Glycopeptidolipids	*L*‐TOF (**s‐DHB**)	An improved method for rapid detection of *M. abscessus* complex based on species‐specific lipid fingerprint by routine MALDI‐TOF	Khor et al. (2021)
*Apilactobacillus kosoi* 10H^T^, *Lactiplantibacillus plantarum* JCM1149^T^ and *Lacticaseibacillus rhamnosus* GG	Lipoteichoic acid anchor region	*R*‐TOF/TOF (**DHB**)	Investigation of the role of lipoteichoic acid from the genus *Apilactobacillus* in inducing a strong IgA response	Matsuzaki et al. (2022)
*E. coli*	Membrane protein integrase glycolipid (**235**)	TOF	Summary of discovery, structure, synthesis and biological activity	Fujikawa et al. (2019)
*Halorientalis salina sp. nov., H. marina sp. nov.,* and *H. litorea sp. nov*.	Sulfated mannosyl glucosyl diethers	TOF (‐ve ion)	Results indicated three novel species.	Wang, Sun, Wu, Zheng et al. (2022)
*Lactococcus cremoris* 3107	Cell‐wall polysaccharide	TOF/TOF (**DHB**)	Investigation of mechanism for lactococcal phage TP901‐1 infection. Glycan modification not implicated	Ruiz‐Cruz et al. (2022)
*Mycobacterium abscessus*	Glycopeptidolipid	QIT‐TOF (**DHB**), MS/MS	Glycopeptidolipid glycosylation shown to control surface properties and pathogenicity	Daher et al. (2022)
*Mycobacterium bovis* BCG	Trehalose dimycolate (**236**)	TOF	The liposome of trehalose dimycolate extracted from *M. bovis* BCG shown to induce antitumor immunity *via* the activation of dendritic cells and CD8^+^ T cells	Shiga et al. (2021)
*Pseudomonas aeruginosa*	Rhamonolipids (e.g., **237**)	TOF/TOF (**CHCA**)	The rhamnolipids shown to promote methane hydrates formation in fixed bed silica gel medium	Arora et al. (2021)
*P. aeruginosa*	Rhamonolipids	*R*‐TOF/TOF	Characterization and cytotoxicity of rhamnolipids against breast cancer MDA‐MB‐231 cell line	Mishra et al. (2021)

^a^ Format (not all items present): MALDI method (**matrix**), other methods.


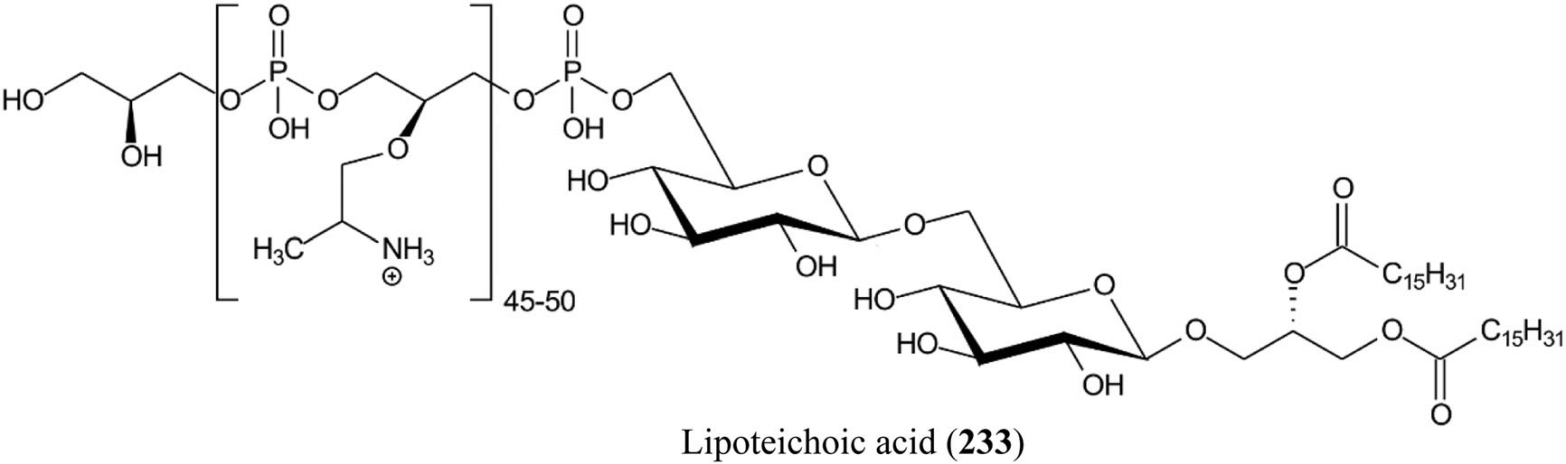



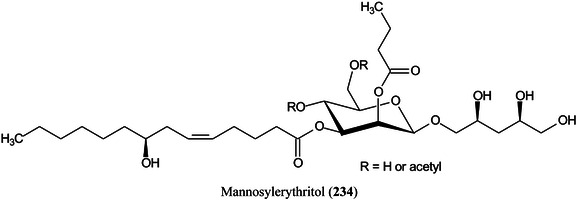



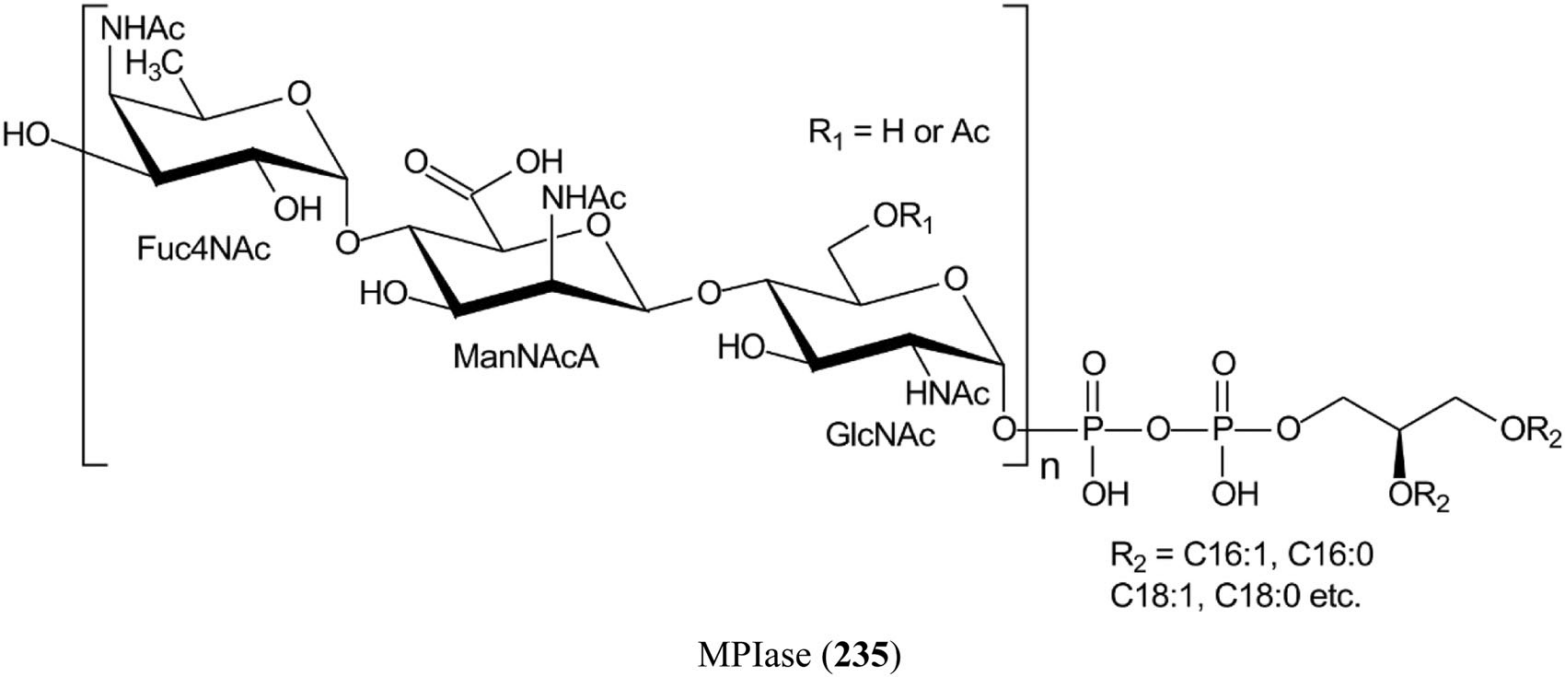



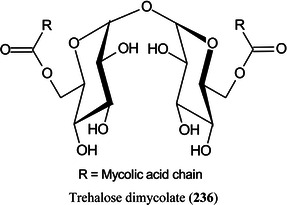



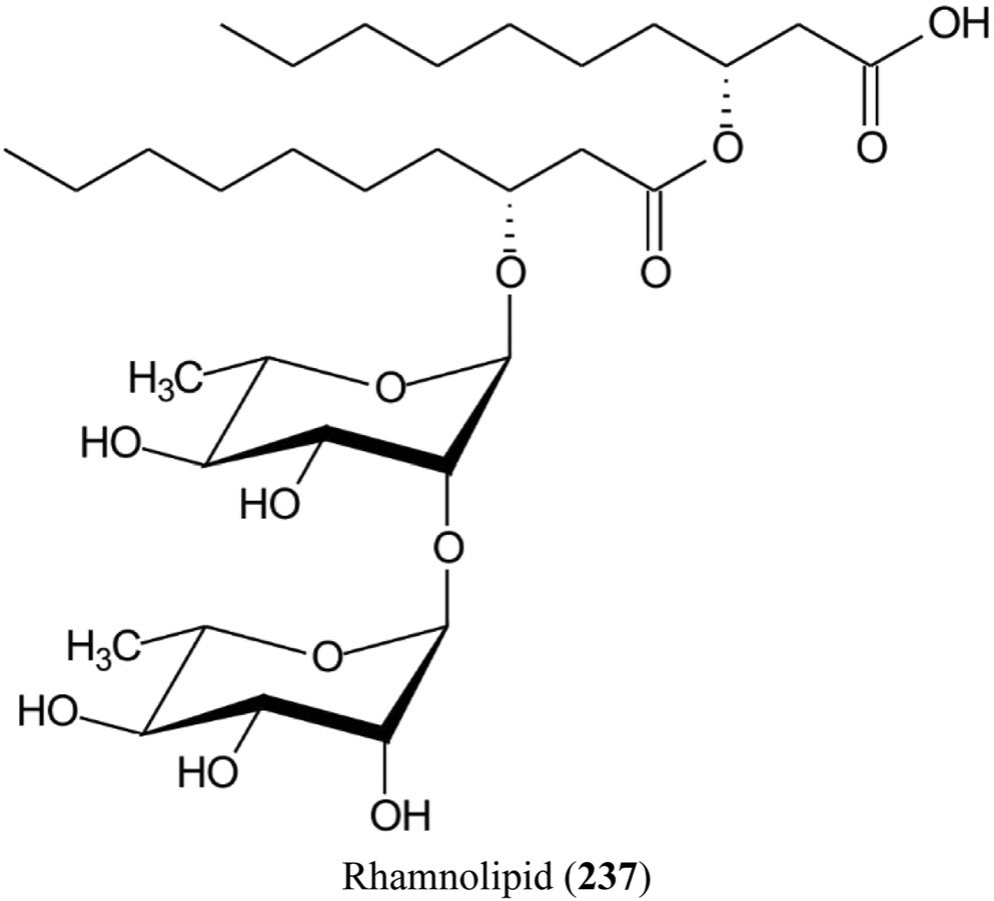


#### 13.7.4 Glycosides ‐ Natural Products

Although identification of natural products, mainly glycosides from plants, is a very active field, most mass spectrometric work appears to be conducted with ESI and LC‐MS/MS techniques. However, MALDI features in several publications and relevant reviews relating to the technique are summarised in Table 32.

**Table 32:** Reviews and general articles on the analysis miscellaneous natural products.

Subject	Notes	Citations	References
Current knowledge of intestinal absorption of anthocyanins	Short overview including MALDI imaging	59	Hahm et al. (2022)
Chemical constituents and chemical analysis of *Ginkgo biloba* leaf, extract, and phytopharmaceuticals	Extensive list of flavonoids and glycosides, discussion of analytical methods and quantification	122	Liu, Wang, Zhang, et al. (2021)
Extraction, purification, structural characteristics and biological properties of the polysaccharides from *Codonopsis pilosula*	Includes table listing use of MALDI to investigate structures of the glycans	136	Luan et al. (2021)
Application of MS‐based metabolomic approaches in analysis of starfish and sea cucumber bioactive compounds	Contains table with details of analysis of starfish polar steroids and sea cucumber triterpene glycosides	183	Popov et al. (2022)
*Holothuria* triterpene glycosides: A comprehensive guide for their structure elucidation and critical appraisal of reported compounds	Comprehensive review. Table of reported structures. Mass spectral fragmentation	93	Puspitasari et al. (2022)
Bioactive polysaccharides and oligosaccharides from garlic (*Allium sativum* L.): Production, physicochemical and biological properties, and structure–function relationships	Comprehensive review. Occurrence, production and extraction of sugars. Modifications such as sulfation. Characterization (MALDI). Metabolism, biological activity	433	Qiu et al. (2022)
Separation procedures for complicated mixtures of sea cucumber triterpene glycosides	With several references to MALDI analysis and MALDI imaging	89	Silchenko et al. (2022)

A method claiming to provide improved coverage of plant metabolites involves examination of powder derived from dried plant fragments rather than the products of liquid extraction (Islam et al., 2022). Ground plant powder was fixed to a metal plate using double‐sided adhesive tape and interrogated directly with the laser in an FT‐ICR instrument. No matrix was required, various compounds in the powder were presumed to provide this function. The method required a smaller amount of sample (~200 μg) compared with traditional methods. By employing the powder method using *Centella asiatica* leaves, a higher number of reproducible molecular formulae (*>*5000) and metabolites (*>*650) were obtained than with the conventional methods. Flavonoids, phenolic acids, xanthones, lipids, carbohydrates, terpenoids and alkaloids were all detected from leaves, stems and roots of the plant.

Yamagaki et al. (2022) have shown that the postsource decay (PSD) fragmentation spectra of the [M + Na]^+^ ions of 4′‐*O*‐galloylpaeoniflorin (**238**) and 4‐*O*‐galloylalbiflorin (**239**) are different even though they are positional isomers. In particular, 4’‐galloylpaeoniflorin tended to eliminate a galloyl group to produce major ions whereas 4‐*O*‐galloylalbiflorin eliminated the sugar residue.


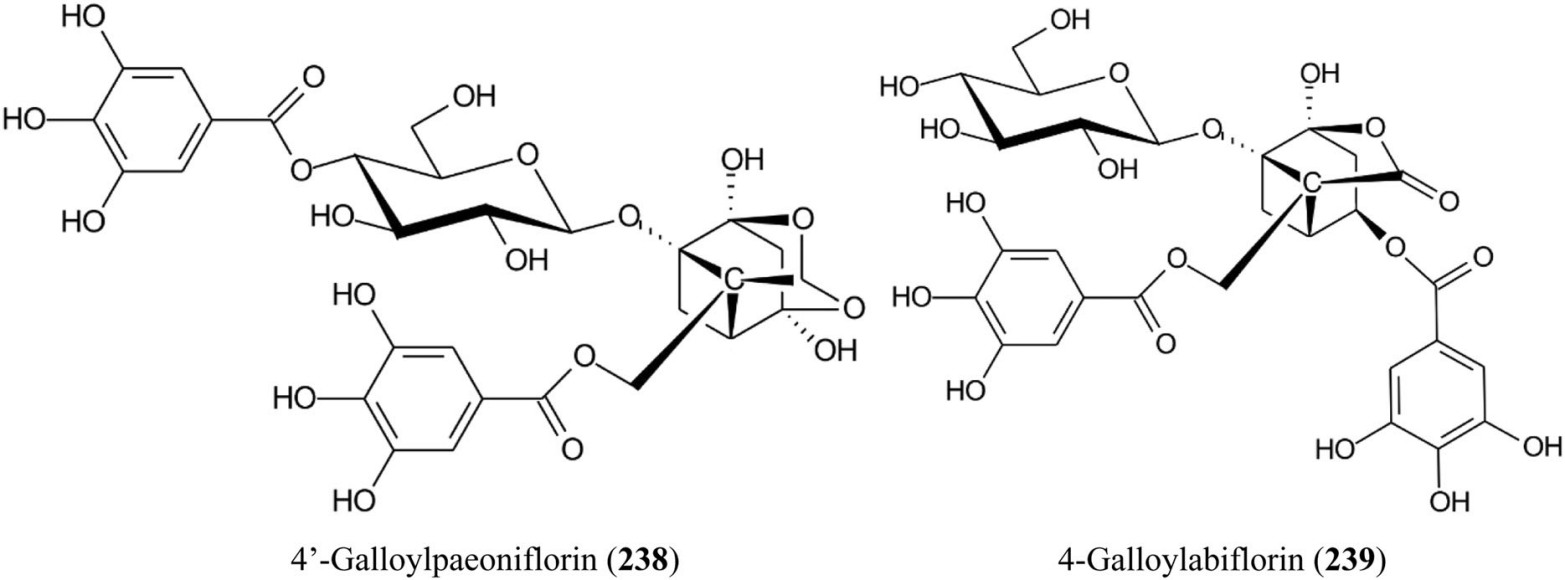


Investigations of enzyme‐assisted extraction of various compounds from plant material, with the aim of maximizing the quality of the extracts, have been made by Rafińska et al. (2022). Pectinase was shown to be particularly efficient in obtaining high quality extracts with a low content of interfering compounds using *Medicago sativa* L. as a test plant. The types of compounds that were investigated included carbohydrates, flavonoids and phenolic acids.

Other publications are listed in Table 33.

**Table 33:** Use of matrix‐assisted laser desorption/ionization mass spectrometry for the study of natural products.

Source	Compound	Methods^a^	Notes	References
**Carbohydrates**				
*Beta vulgaris* L. (red beet)	Mono‐ and poly‐saccharides	*R*‐TOF/TOF (**DHB**)	Structural characterization	Hotchkiss et al. (2022)
*Fucus vesiculosus*	Fucoidan (**240**)	TOF/TOF (**DHB** (native), **CHCA** (per‐Me))	In study of competitive inhibition of gastrointestinal norovirus binding	Hanisch, Aydogan, and Schroten (2021)
*Sargassum horneri*	Sulfated polysaccharides	TOF/TOF (**DHB**), MS/MS	Structural characteristics and immune‐enhancing activity	Kim, Hwang, et al. (2022)
**Glycosides**				
*Agave marmorata* Roezl	Flavonoids, phenolics, steroidal glycosides	*R*‐TOF/TOF (**DHB**)	Study of the micropropagation of seed‐derived clonal lines of this endangered plant and their compatibility with endophytes	Martinez‐Rodriguez et al. (2022)
*Albizia julibrissin* (Chinese medicinal herb)	Oleanane‐type glycoside (**241**)	*R*‐TOF/TOF	Structural determination and cytotoxic activity	Han et al. (2021)
*Calicotome spinosa* (Gorse)	Isoflavonoid glucosides (**242**)	MALDI	Extraction and identification of bioactive compounds	Mustafa et al. (2022)
*Deschampsia antarctica*	Various glycosides, e.g. orientin 2"‐*O‐β*‐arabino‐pyranoside (**243**) and isoswertiajaponin 2"‐*O‐β*‐arabinopyranoside	TOF/TOF (CHCA)	Chromatographic and mass spectrometric analysis of secondary metabolites of *D. antarctica* from Galindez Island, Argentine Islands	Ivannikov et al. (2022)
*Holothuria poli* (sea cucumber)	Triterpene glycosides (e.g. holothurin A, **244**)	TOF (‐ve)	Characterization and investigation of anti‐proliferation in tumor cell lines	Mert‐Ozupek et al. (2022)
*Holothuria scabra* (viscera)	Hemolytic saponins (structures similar to holothurin A)	Q‐TOF (**DHB**/**DMA**)	Structural characterization, desulfation with microwaves to determine toxic fraction	Savarino et al. (2022)
*Khaya ivorensis* (African mahogany)	Quercetin‐7‐*O*‐hydroxybenzoic acid‐3‐*O*‐hexoside	TOF	Identification of phenolic compounds	Athomo et al. (2021)
*Pinus pumila*	Triterpene glycoside (**245**)	TOF	Structural characterization	Liu, Liu, Tao, et al. (2021)
*Prosopis* species	Glycosides and other compounds	TOF, TOF/TOF	Mainly review of various compounds	Picariello et al. (2022)
*Quillaja saponaria* (commercial)	Triterpenoid glycosides	TOF/TOF (**DHB**)	For development of low‐cost cage‐like particles to formulate veterinary vaccines	Lupi et al. (2022)
*Scutellaria brevibracteata* subsp. *subvelutina*	Iridoid glycosides (8, e.g., epiloganic acid (**246**), phenylethanoid glycoside (martynoside, **247**)	TOF	Identification of secondary metabolites and their *in vitro* anti‐inflammatory activities	Erdoğan et al. (2021)
**Glycolipids**				
*Natrinema halophilum* sp. nov., *Natrinema salinisoli* sp. nov., *Natrinema amylolyticum* sp. nov.	Sulfated mannosyl glucosyl diethers	TOF/TOF (**9‐AA**)	Detection as part of description of species	Bao et al. (2022)
*Haloterrigena alkaliphila* sp.nov.	Sulfated mannosyl glucosyl diethers	TOF/TOF (**9‐AA**)	Detection as part of description of species	Bao et al. (2022)
*Perilla frutescens* (L.)	Glycoglycero‐lipids	TOF/TOF (**DHB**)	Structural characterization and anti‐inflammatory activities	Zi et al. (2021)
*Quillaja lancifolia* (*Q. brasiliensis*)	*Quillaja* saponins	TOF (**DHB**)	Investigation of nanoparticles formed from saponins	Cibulski et al. (2022)
*Rohdea chinensis*	Steroidal sapogenins (2)	Spiral‐TOF	Identification of two new steroidal sapogenins from rhizomes and their antifungal activity	Yao et al. (2022)
*Streptomyces* sp.	Tunicamycin	MALDI	Effect of acyl chain on activity	Price et al. (2021)
**Glycopeptides, etc**.				
*Micromonospora chersina* strain DSM 44154	Lipoglyco‐depsipeptide Chersinamycin (antibiotic)	TOF/TOF	Discovery of six ramoplanin family gene clusters and the antibiotic	Morgan et al. (2021)
Bee pollen from rape (*Brassica napus* L.)	Reversibly glycosylated polypeptide‐2	TOF, LC‐MS	Purification and characterization	Zhang, Sun, et al. (2021)
Major component of the venom of the ant *Myrmecia gulosa*.	*O*‐Linked glycopeptide (Mg7a)	*L*‐TOF/TOF (**CHCA**)	Identification and synthesis	Robinson et al. (2021)
**Other compounds**				
*Actinokineospora spheciospongiae*	Polyene macrolides (natamycin, luconsomycin, kineosporicin (**248**)	TOF	Sterol sponge mechanism of fungicidal action is shown to be conserved.	Guo, Zhang, et al. (2021)
*Dolichos lablab* L. hull	Pectin‐glucuronoxylan complex	*L*‐TOF/TOF (**DHB**)	Structural characterization	Liu, Tang, et al. (2022)
*Haloprofundus salilacus* sp. nov., *H. halobius* sp. nov. and *H. salinisoli* sp. nov.:	Various including sulfated mannosyl glucosyl diether, mannosyl glucosyl diether‐phosphatidic acid and sulfated mannosyl glucosyl diether‐phosphatidic acid	TOF/TOF (**9‐AA**, ‐ve)	Structural characterization	Li, Xin, et al. (2022)
*Halosolutus amylolyticus* gen. nov., sp. nov., *H. halophilus* sp. nov. and *H. gelatinilyticus* sp. nov.	Polar lipids and glycolipids	TOF	Description of species	Sun, Wang, et al. (2022)
Rice (*Oryza sativa*) straw	Soluble polysaccharides conjugated with *p‐*coumaric acid, ferulic acid, vanillic acid, and vanillin	*R*‐TOF/TOF	Structural characterization. Shown to alleviate ethanol fermentation stresses in *Saccharomyces cerevisiae*	Wang, Zheng, et al. (2022)
*Rosa roxburghii*	Ellagitannins	QIT‐TOF	Structural identification and suppression of poly(I:C)‑induced IL‑8 production in human keratinocytes	Takayama et al. (2021)
*Tabernaemontana contorta* Stapf.	Glyco‐cerebrosides (**249**)	TOF	Identification of new glycocerebrosides from the trunk and their antibacterial activity	Ebede et al. (2022)
Wood (oak, hornbeam, walnut)	Glycosylated conyferyl alcohols (**250**)	TOF (**DHB**)	Destructive behaviour of wood by the white‐rot fungus *Fomes fomentarius*	Bari et al. (2021)

a Format (not all items present): MALDI method (**matrix**), other methods.


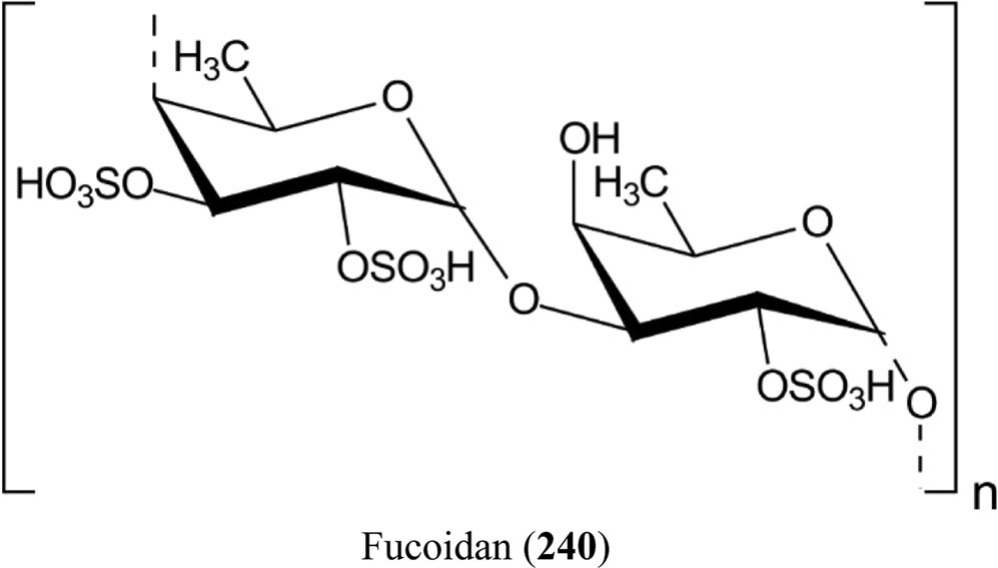



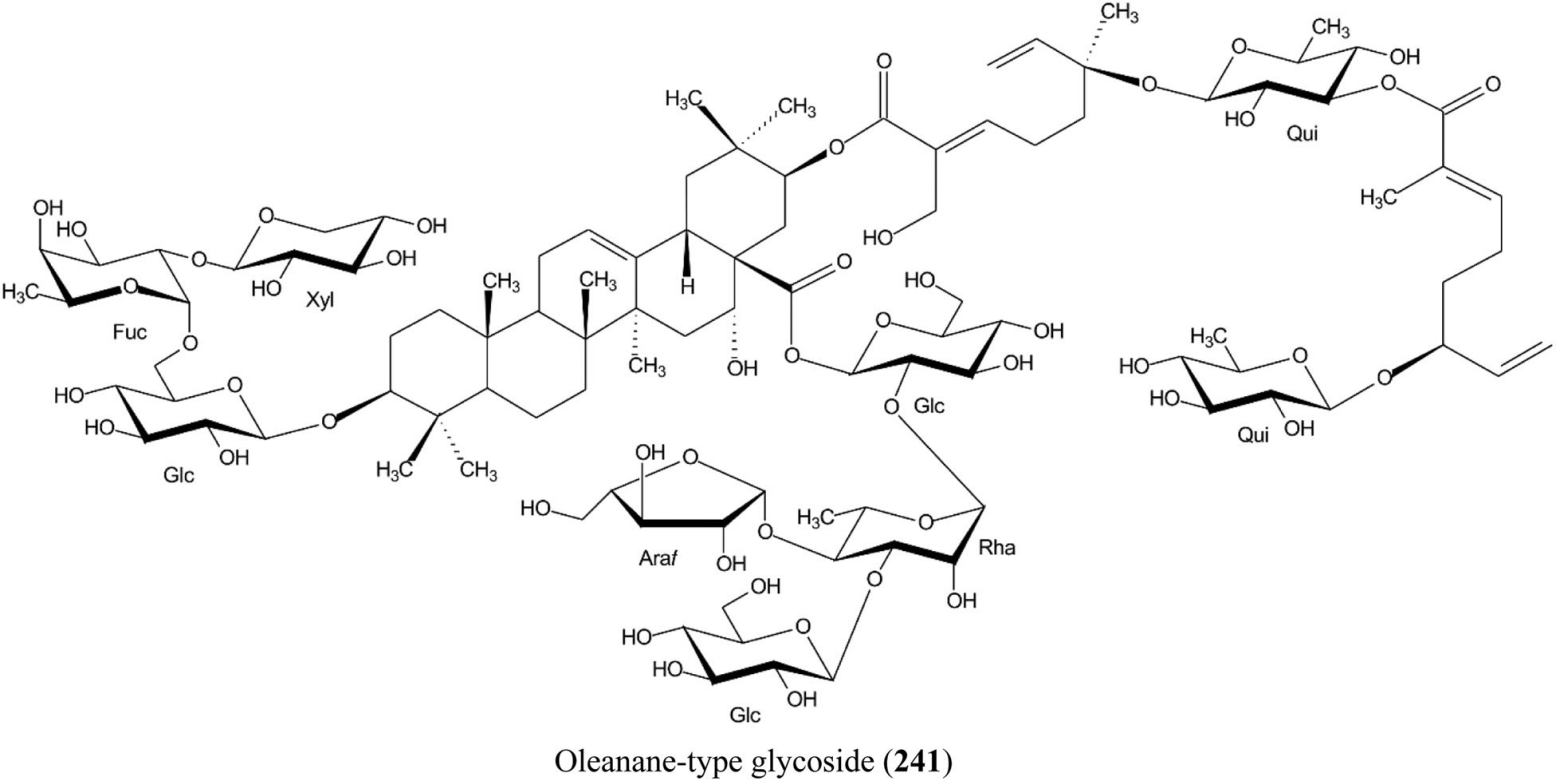



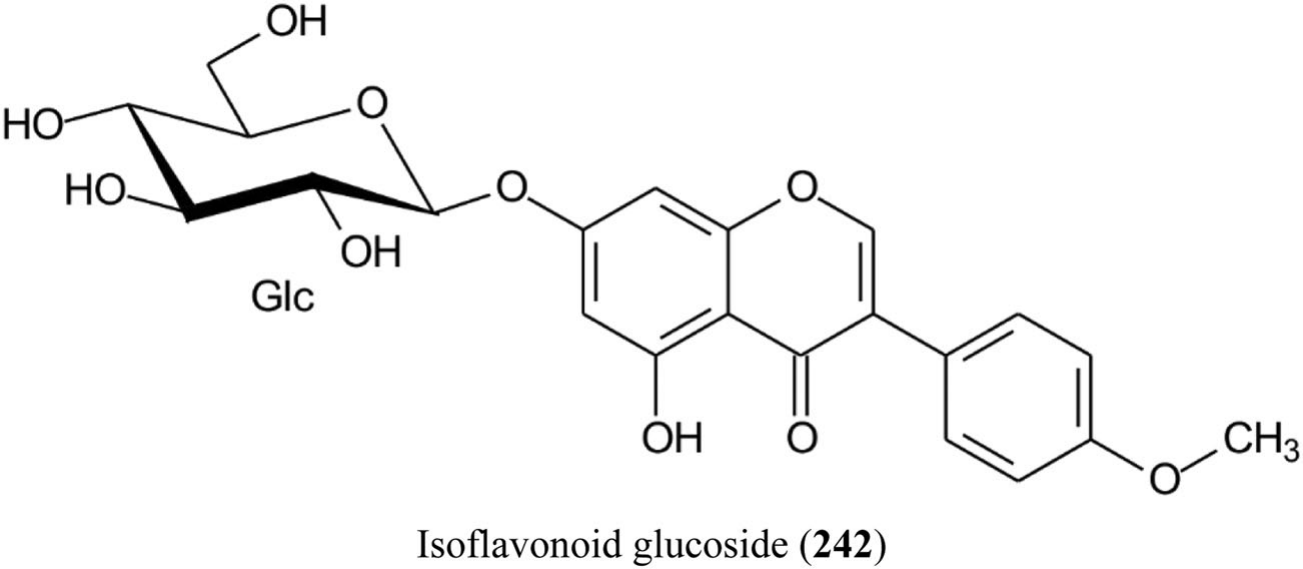



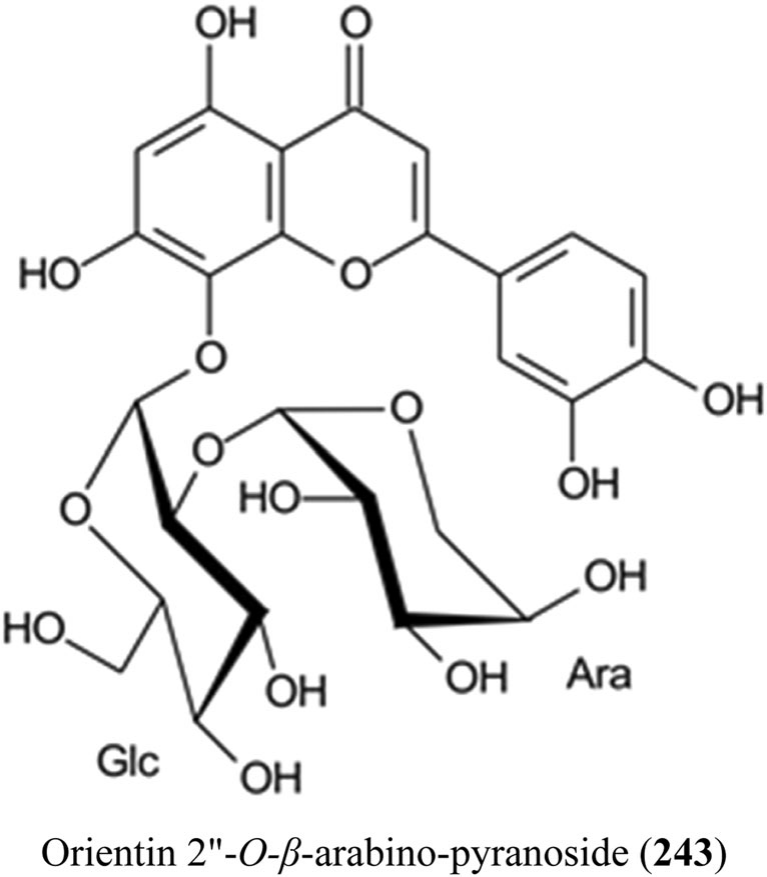



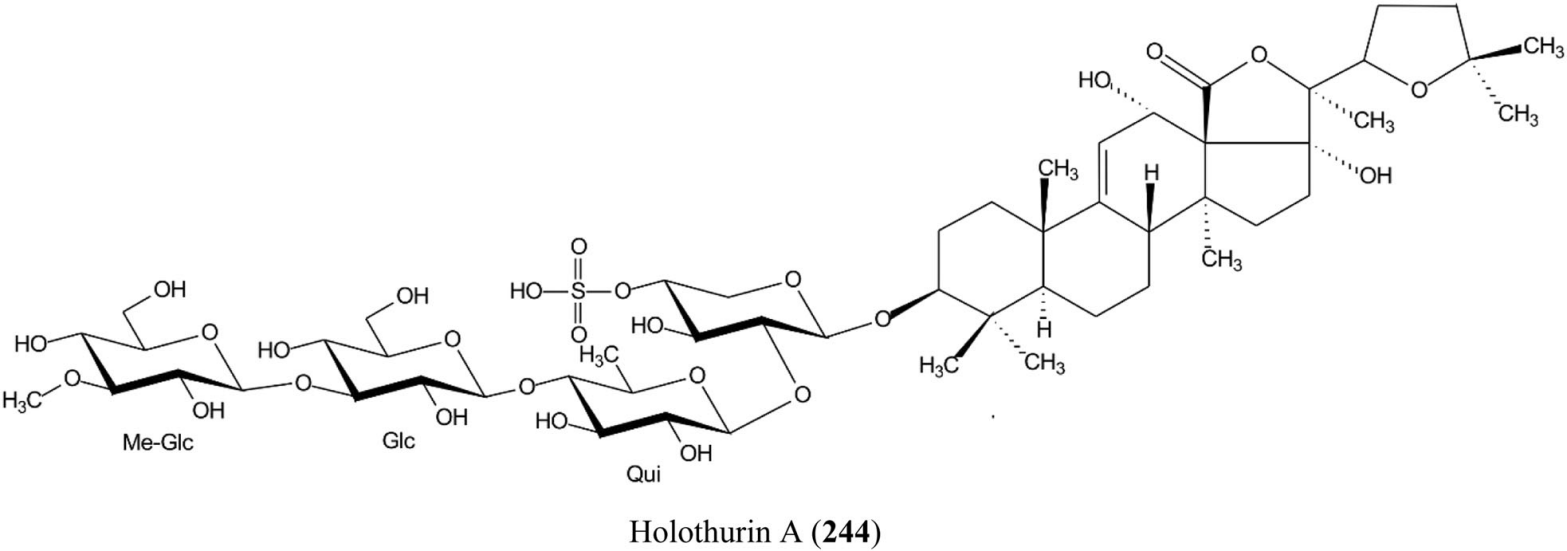



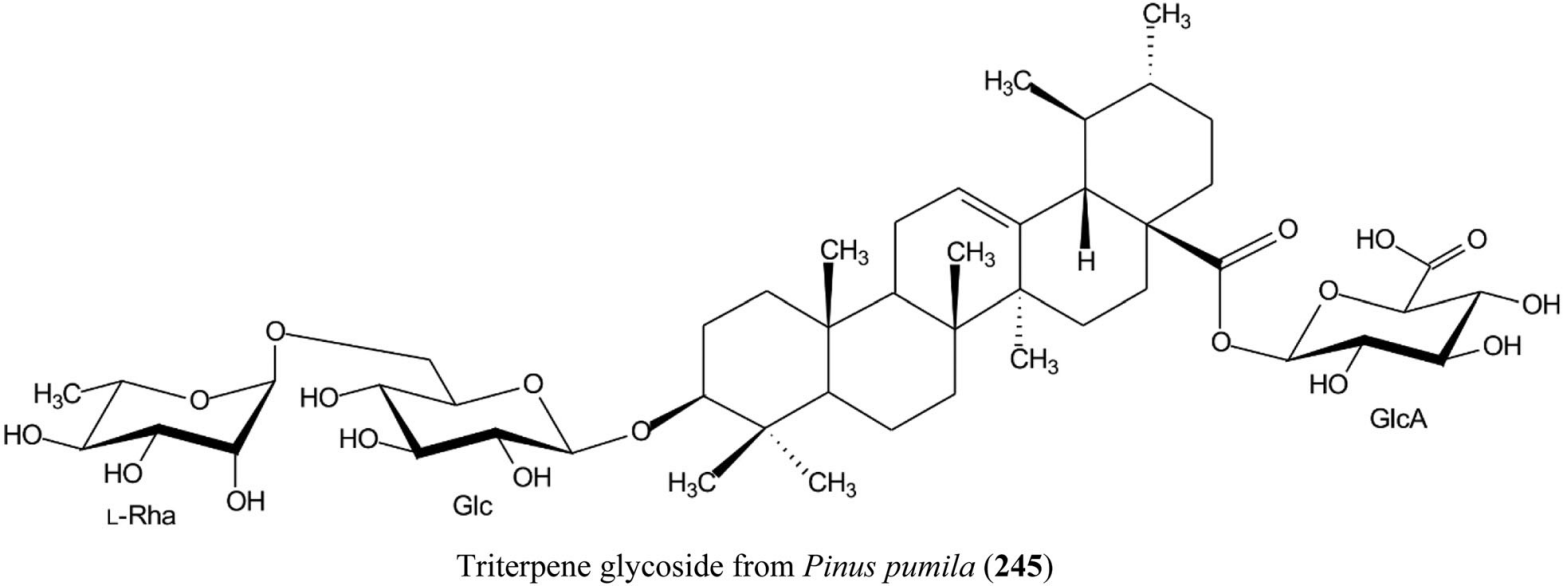



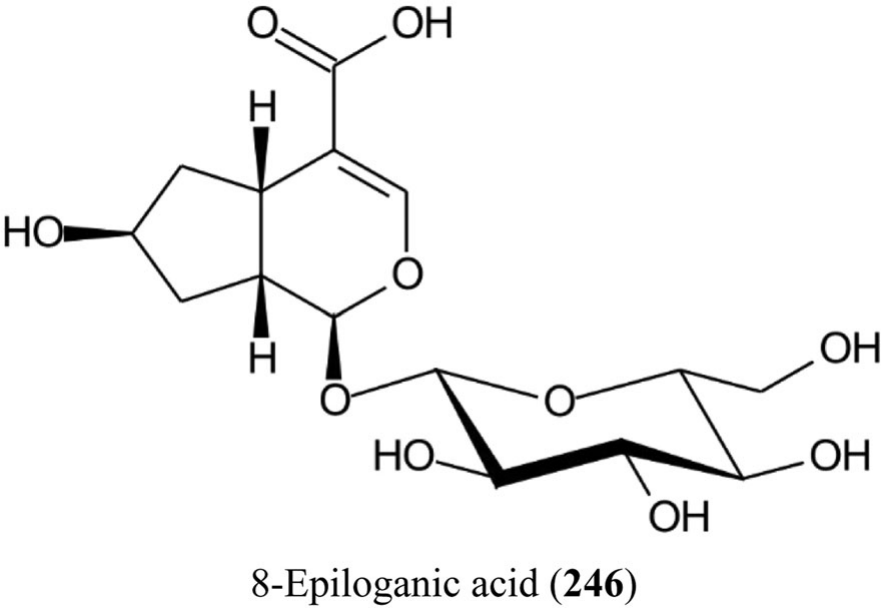



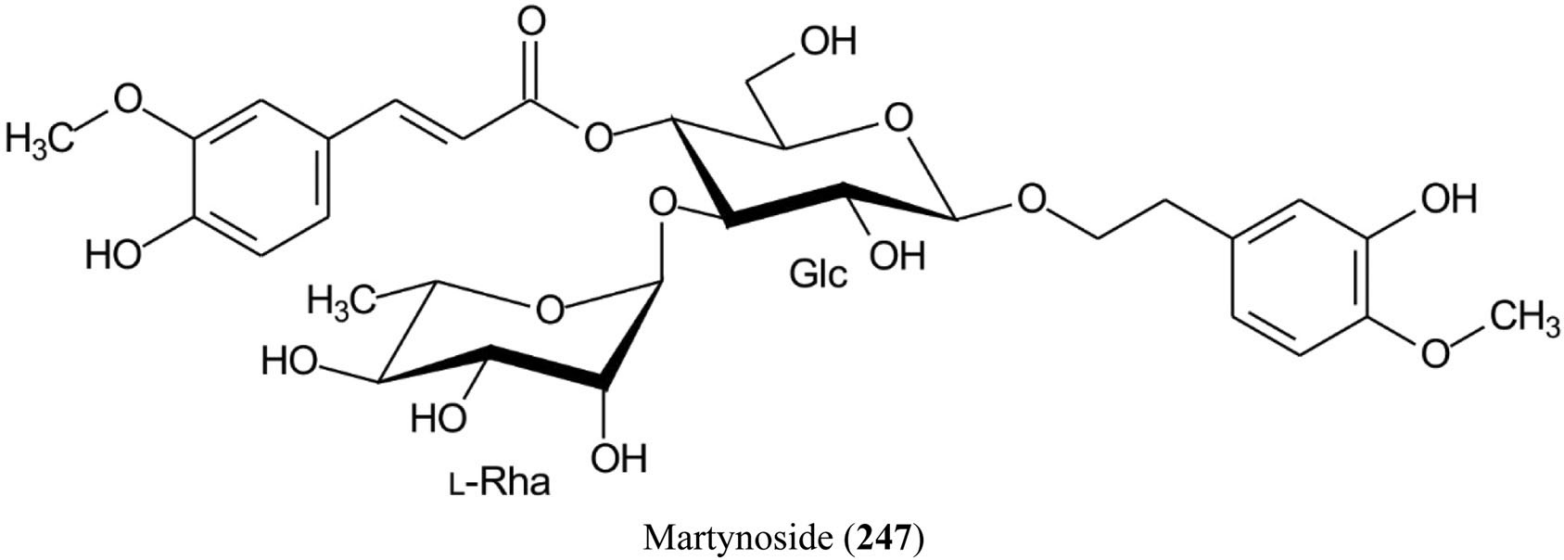



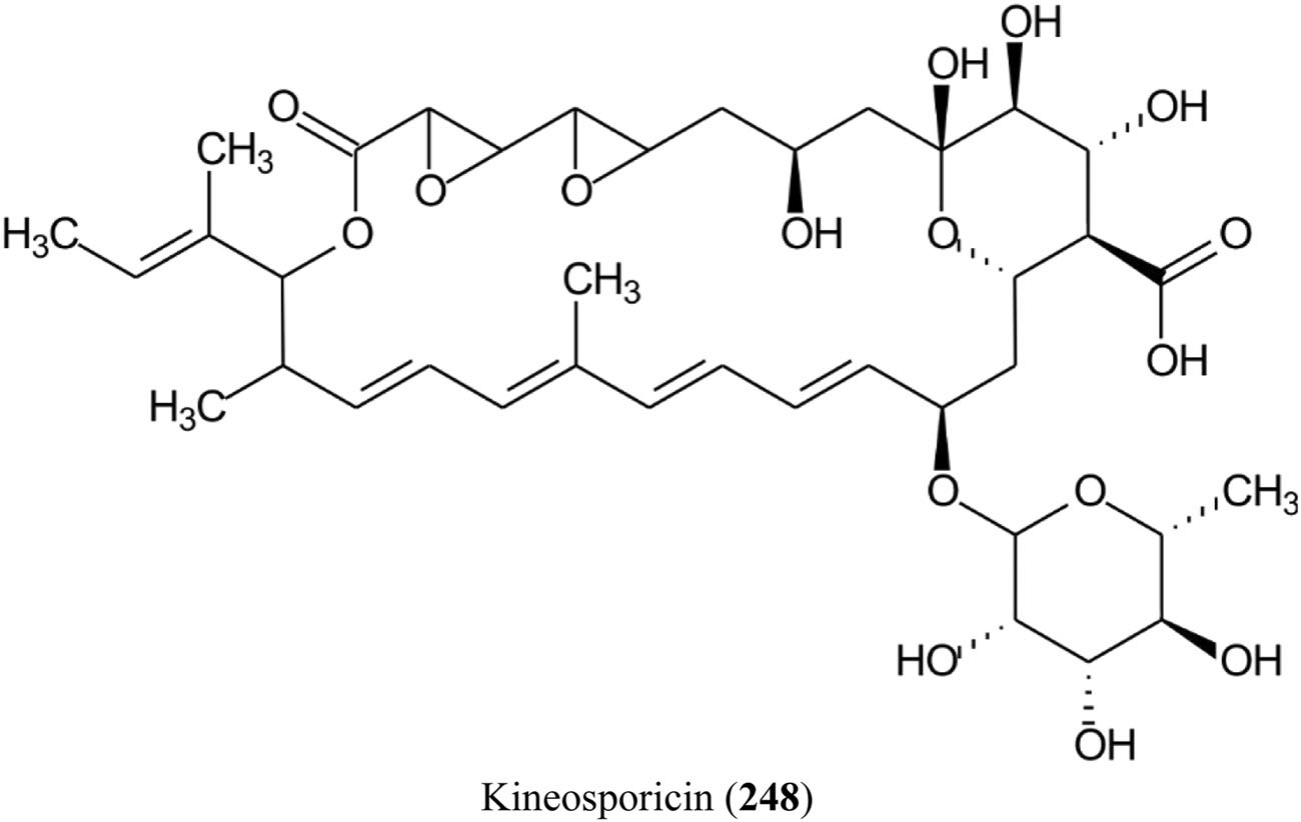



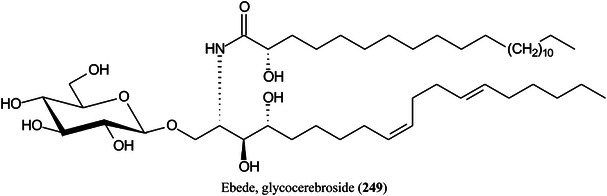



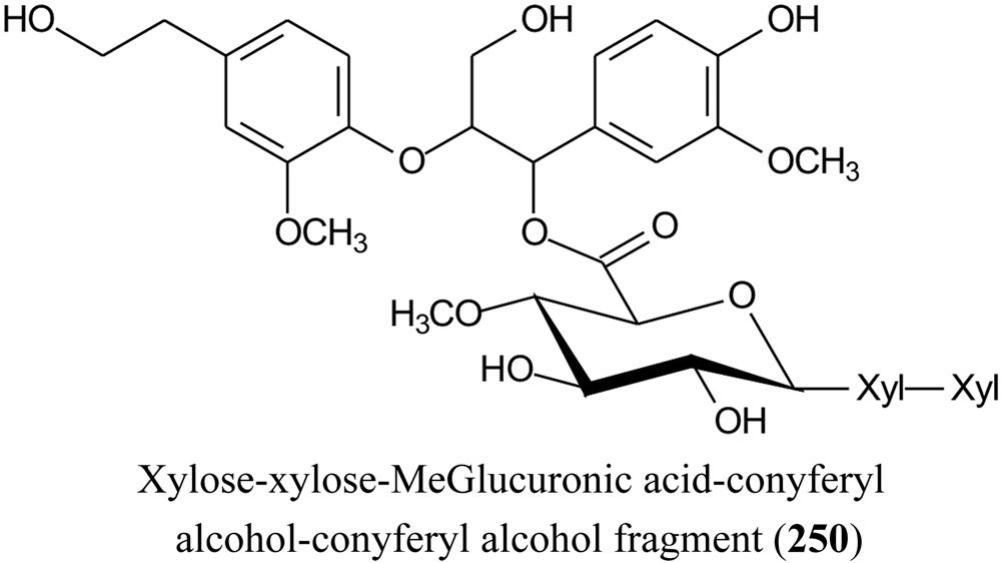


## 14 USE OF MALDI MASS SPECTROMETRY IN OTHER FIELDS

### 14.1 Enzymes

Another area where MALDI finds use is in monitoring the products of enzymatic digestions. One group of enzymes that has received considerable attention (see Table 34) are lytic polysaccharide monooxygenases (LPMOs) and a review on tools for assessing their activity on cellulose (includes HPLC, MALDI‐TOF, LC‐MS, NMR) has been published by Calderaro et al. (2021).

**Table 34:** Use of matrix‐assisted laser desorption/ionization to study the products of enzymes' action on carbohydrates.

Enzyme	Source	Methods^a^	Notes	References
**Glycosyltransferases**				
Acholetin phosphorylase	*Acholeplasma laidlawii*	MALDI, glycans	Shown to synthesise poly‐β‐1,3‐GlcNAc, completing the suite of β‑linked GlcNAc polysaccharides	Macdonald et al. (2022)
Archaeal β‐1,4‐*N*‐acetylglucosaminyl‐transferase (Agl24),	*Sulfolobus acidocaldarius*	TOF (**DHB**/TFA), glycolipids	Investigation of enzyme responsible for synthesis of *N*‐glycan chitobiose core in archaea	Meyer, Adam, et al. (2022)
Cycloisomalto‐oligosaccharide glucanotransferase	*Thermo‐anaerobacter thermocopriae*	TOF/TOF (**DHB**), glycans	Carbohydrate‐binding module of the enzyme shown to improves its cyclodextran production	Hong et al. (2022)
Enzyme from *blr2358* gene	*Bradyrhizobium diazoefficiens* USDA110	TOF/TOF (**2,3‐DHB**), glycans	Identification of gene involved in exopolysaccharide biosynthesis	Xu, Ruan, et al. (2021)
Fucosyl transferase FUT8	Recombinant in HEK293F cells	PNGase F, TOF/TOF (**DHB**), *N*‐glycans	Investigation as to which modifications affect enzyme's ability to fucosylated core of *N*‐glycans	Zhang, Yang, et al. (2021)
Fucosyl transferase FUT8	Human FUT8	Glycans from egg yolk, TOF/TOF (**DHB**)	Investigation of factors influencing core fucosylation	García‐García et al. (2021)
Galacturonosyl‐transferases	*Arabidopsis thaliana*	TOF, glycans	Multiple *Arabidopsis* Gal‐transferases shown to synthesize polymeric homogalacturonan by oligosaccharide acceptor‐dependent or *de novo* synthesis	Engle et al. (2022)
α‐1,3‐Glucosyl‐transferase	*Pneumococcus* serotype 8C	TOF, glycans	Biochemical characterization and synthetic application	Wang, Sun, et al. (2021)
Glycosyl‐transferases, SdgB and SdgA,	Recombinant in *E. coli*	TOF/TOF (**DHB**) glycopeptides	SdgB but not SdgA transferred GlcNAc to staphylococcal adhesive proteins	Kim, Baek, et al. (2021)
Glycosyltransferase GT43 family	*Phyllostachys edulis* (bamboo)	TOF, glycans	Identification and expression analysis of enzymes reveal their potential function in xylan biosynthesis during rapid growth	Li, Wang, Yang, et al. (2021)
Glycosyltransferase family 61	Conifers	*R*‐TOF/TOF, glycans	Study of xylan substitutions in conifers	Zhong, Phillips, et al. (2022)
Human exostosin‐like 3	Human	TOF (**DHB**), glycans	Investigation into its mechanism of action for heparin sulfate synthesis	Wilson, Dendooven, et al. (2022)
Inverting S/O‐HexNAc‐transferase	*Streptomyces venezuelae* ATCC 15439	TOF, glycopeptides	First experimental evidence of *S*‐linked glycosylation in actinobacteria	Sharma, Ahlawat, et al. (2022)
IRX10 Xylan synthases	Rice (*Oryza sativa*)	TOF/TOF (**DHB**), glycans (procainamide derivatives)	Identification of a xylan‐rich nanodomain at pit borders of xylem vessels	Wang, Yang, Wen, et al. (2022)
β‑KDO transferase KpsS	Recombinant	MALDI	Characterization of the initiating enzyme in the biosynthesis of the lipid acceptor for *E. coli* polysialic acid	Lanz et al. (2021)
Mannan synthase	*Arabidopsis thaliana*	*R*‐TOF (**DHB/DMA**)	The *TaCslA12* gene expressed in the wheat grain endosperm shown to synthesize wheat‐like mannan when expressed in yeast and *Arabidopsis*	Verhertbruggen et al. (2021)
Protein S‐glycosyl‐transferases (thuS)	*Bacillus thuringiensis* serovar andalousiensis BGSC 4AW1	TOF, glycoprotein, glycopeptides	Structural and mechanistic investigations of protein *S*‐glycosyltransferases	Fujinami et al. (2021)
RG‐1 Rhamnosyl‐transferases	*Arabidopsis thaliana*	TOF (**DHB**), glycans (2‐AB)	Investigation of the role of the enzyme in the biosynthesis of rhamnogalacturonan I in plants	Amos et al, (2022)
Trans‐sialidase	*Trypanosoma congolense*	*R*‐TOF/TOF (**CHCA**), glycopeptides	*N*‐Glycosylation shown to modulate enzymatic activity	Rosenau et al. (2022)
UTP‐glucose‐1‐phosphate uridylyltransferase YngB	*Bacillus subtili*	*R*‐TOF (**DHB/s‐DHB**), R‐TOF/TOF‐MS/MS, glycolipids	Enzyme shown to contribute to wall teichoic acid glucosylation and glycolipid formation during anaerobic growth	Wu, Rismondo, et al. (2022)
Xylan arabinosyl‐transferases (OsXATs)	Various grasses	*R*‐TOF (**DHB**/**HIQ**), glycans	Results show that that multiple OsXATs are involved in 3‐*O*‐arabinosylation of xylan	Zhong, Cui, et al. (2021)
Xylan arabinosyl‐transferases	Grasses recombinant in *Arabidopsis thaliana*	R‐TOF/TOF, glycans	Identification of enzymes catalyzing addition of 2‐*O*‐xylosyl residue onto arabinosyl side chains of xylan in grass species	Zhong, Lee, et al. (2022)
Xyloglucan xylosyltransferase	*Arabidopsis thaliana* and *Oryza sativa* (rice) in HEK293 cells	TOF (**DHB**), glycans	A single xyloglucan xylosyltransferase shown to be sufficient for generation of the XXXG xylosylation pattern of xyloglucan	Zhong, Phillips, et al. (2021)
Xyloglucan xylosyltransferase 1	*Arabidopsis thaliana*.	TOF (**DHB**), glycans	Enzyme shown to display promiscuity toward donor substrates during *in vitro* reactions	Ehrlich et al. (2021)
**Glycosidases**				
Chitosanase	*Bacillus toyonensis* CCT 7899	TOF/TOF (**DHB**), glycans	Purification and functional oligosaccharide production	Dantas et al. (2022)
β‐*N*‐Acetylhexosaminidase	*Cateni‐bacterium mitsuokai*	TOF/TOF, glycan (LNT2 glycan, **251**)	Biochemical characterization of a form suitable for the synthesis of lacto‐*N*‐triose II	Liu, Ma, Shi, et al. (2021)
α‐Agarases	*Colwellia echini* A3^T^	TOF (**DHB**), glycans	A novel auxiliary agarolytic pathway shown to expand the metabolic versatility in the agar‐degrading marine bacterium *C echini* A3T	Pathiraja et al. (2021)
β‐Agarase	*Cellulophga* sp. J9‐3	TOF/TOF, glycans	Purification and biochemical characterization of β‐agarase produced by marine microorganism (In Korean)	Kim, Kim, et al. (2021)
Alginate lyases (AlgLs)	*Pseudomonas aeruginosa* (Pae‐AlgL) and *Azotobacter vinelandii* (Avi‐AlgL)	TOF/TOF, glycans	Investigation of mannuronate preference	Zeng, Li, et al. (2021)
*α*‐Amylase (Amyrel)	*Diptera muscomorpha)* (Drosophila)	*R*‐TOF/TOF, glycans	A novel glucose‐forming *α*‐amylase with 4‐*α*‐glucanotransferase activity	Feller et al. (2021)
α‐l‐Arabino‐furanosidase, r*Cs*Abf62A	*Cellulomonas* sp. B6	TOF (**DHB**), glycans	Investigation of the xylan degradation system	Garrido et al. (2022)
Chitinase	*Enterobacter cloacae* subsp*. cloacae* (EcChi2)	TOF/TOF (**DHB**), glycans	Catalytic efficiency of multi‐domain transglycosylating chitinase is influenced by polycystic kidney disease domains	Mallakuntla and Podile (2021)
Chitinase	*Streptococcus macrosporeus* VTCC 940003	TOF/TOF (**DHB**), glycans	Chito‐oligosaccharide production by the enzyme and their inhibition activities on *Botrytis cinerea*	Anh et al. (2021)
Chitosanase	*Bacillus amyloliquefaciens*	TOF, glycans and protein	Identification of a new class of chitosanase from *B. amyloliquefaciens* for the generation of chitooligosaccharides	Bhuvanachandra et al. (2021)
Chitosanase	*Streptomyces niveus*	*R*‐TOF/TOF (**DHB**, +ve. –ve), glycans	Expression and biochemical characterization of enzyme suitable for preparation of chitobiose	Chen, Cheng, et al. (2021)
Difructose dianhydride I synthase/hydrolase	*Bifidobacterium dentium*	TOF (**5‐Me‐DHB**), glycans (free and OAc)	Identification of a novel glycoside hydrolase family	Kashima et al. (2021)
Endo‐chitosanase, AqCoA	*Aquabacterium* sp. A7‐Y	TOF/TOF (**DHB**), glycans	Use for synthesis of active chitooligosaccharides and their application in fungal disease protection	Wang, Li, Liu, et al. (2021)
Endoglucanase *Rf*GH5_4	Recombinant from *Ruminococcus flavefaciens* FD‐1 v3	*R*‐TOF (**DHB**), glycans	Use for recycling lignocellulosic plant biomasses	Gavande et al. (2022)
Exo‐β‐1,3‐glucanase	Moose rumen	TOF/TOF, glycans	Enzyme shows a structural framework similar to yeast exo‐β‐1,3‐glucanases	Kalyani et al. (2021)
α‐l‐Fucosidases	Human, *Lactobacillus casei* and *Bacteroides fragilis*.	*R*‐TOF/TOF (**DHB/DMA**), *N*‐glycans	Comparative studies on the substrate specificity and defucosylation activity of three α‐l‐fucosidases using synthetic fucosylated glycopeptides and glycoproteins as substrates	Prabhu et al. (2021)
β‐1,3‐Galactosidase (WceF)	*Pantoea stewartii*	MALDI, glycans	WceF shown to be a glycan biofilm‐modifying enzyme with a bacteriophage tailspike‐like fold	Irmscher et al. (2021)
β‐Galactosidase	*Arion lusitanicus* and *Arion vulgaris (A0A0B7AQJ9*), from Sf9 cells	TOF/TOF (**CHCA**) glycans	Identification, characterization, and expression	Thoma et al. (2022)
1,3‐β‐Glucanases	*Penicillium sumatraense*	*R*‐TOF/TOF (**DHB**), glycans	Investigation into why the enzyme digests food but not endogenous glycans	Scafati et al. (2022)
β‐1,3‐Glucanase Gns6	*Oryza sativa* (rice)	TOF/TOF (**DHB**), glycans	Enzyme shown to possess antifungal activity against *Magnaporthe oryzae*	Wang, Liu, Wang, et al. (2021)
GH13 α ‐glucosidase	*Weissella cibaria*	TOF/TOF (**DHB**), glycans	Enzyme shown to uncommonly act on short‐chain maltooligosaccharides	Wangpaiboon, Laohawuttichai, et al. (2021)
*Endo*‐glucanase 16 (EG16)	Various	TOF (**DHB**), glycans	Demonstrates conservation of enzyme activity across highly divergent plant lineages	Behar et al. (2021)
β‑1,3‐Glucanase (MoGluB)	*Magnaporthe oryzae*	TOF/TOF (**DHB**), glycans	Functional characterization and biocontrol of *M. oryzae*	Wang, Zhao, Wang, et al. (2021)
GH10 endo‐xylanase r*Cs*Xyn10A	*Cellulomonas* sp. B6	TOF (**DHB**), glycans	Investigation of the xylan degradation system	Garrido et al. (2022)
GH18 chitinase	Recombinant (*E. coli* BL21(DE3)star cells	*R*‐TOF (**DHB**)	Auxiliary active site mutations shown to enhance the glycosynthase activity for polymerization of chitooligosaccharides	Alsina et al. (2021)
β‐1,3‐Glucanase (thermostable)	*Trichoderma harzianum* in *Pichia pastoris*	*R*‐TOF/TOF, glycans	Expression and use in oligoglucoside hydrolysis	Gao, Yan, et al. (2021)
Family 55 β‑1,3‑glucanase, AcGluA	*Archangium* sp strain AC19	TOF, glycans	Heterologous expression and characterization	Wang, Li, Dong, et al. (2021)
Glucomannanase	*Paenibacillus polymyxa*	TOF/TOF, glycans (konjac glucomannan)	Identification and characterization	Li, Jiang, et al. (2021)
Glycoside hydrolyses (family 39)	*Bifidobacterium longum* subsp. *longum*	TOF (**DHB**), glycans	Mechanism of cooperative degradation of gum arabic arabinogalactan protein by *B. longum* surface enzymes	Sasaki et al. (2022)
Glycoside hydrolyses	*Ustilago maydis*	TOF (**DHB**/**DMA**), glycans	Identification of glycoside hydrolases and carbohydrate oxidases directed toward components of the fungal cell wall	Reyre et al. (2022)
β‐Hexosaminidases	*Nicotiana benthamiana*	*R*‐TOF/TOF (‐ve), glycans	β‐Hexosaminidases along the secretory pathway of *N. benthamiana* shown to have distinct specificities toward engineered helminth *N*‐glycans on recombinant glycoproteins	Alvisi et al. (2021)
Laccase	*Madurella mycetomatis*	Endo H, TOF, glycoprotein	Enzyme immobilized in silica‐coated ZIF‐8 nanocomposites for environmentally friendly cotton bleaching process	Tülek et al. (2021)
β‐Mannanase *Tr*Man5A variants	*Trichoderma reesei*	*R*‐TOF/TOF (**DHB**)	Transglycosylation activity and enzyme synergy for synthesis of allyl glycosides from galactomannan	Butler, Birgersson, et al. (2022)
Pectate lyase AnPL9	*Aspergillus nidulans* in *Pichia pastoris*	TOF/TOF, glycans	First report of a fungal pectate lyase belonging to the PL9 family.	Suzuki, Morishima, et al. (2022)
Pectinase	*Streptomyces hydrogenans* YAM1	TOF (**DHB**), glycans	Investigation of antioxidant and anticancer activities of unsaturated oligo‐galacturonic acids	Abari et al. (2021)
PL17 Oligoalginate lyase	*Zobellia galactanivorans* Dsij^T^	TOF, glycan	Structure–function analysis	Jouanneau et al. (2021)
α‐Rhamnosidases	*Lactobacillus plantarum* WCFS1	TOF/TOF (**DHB**), glycosides	Production and role in deglycosylation of dietary flavonoids naringin and rutin	Ferreira‐Lazarte, et al. (2021)
Xyloglucanase MtXgh74	Recombinant strain *Pichia pastoris* GS115	TOF, glycans	Strategic aromatic residues in the catalytic cleft shown to modify thermostability, mode of enzyme action, and viscosity reduction ability	Berezina et al. (2021)
Xyloglucanase	*Rhizomucor miehei* CAU432 in *Pichia pastoris*	TOF/TOF (**DHB**), glycans	High level expression for production of xyloglucan oligosaccharides and its application in yoghurt	Wang, Li, Miao, et al. (2021)
GH74 xyloglucanase	*Paenibacillus* sp.	TOF (**DHB**/DMA), glycans	Mode of action on tamarind seed xyloglucan	Chen, Ropartz, et al. (2022)
Xyloglucanase B	*Rhizomucor miehei* exptessed in *Pichia pastoris*.	TOF/TOF, glycans	High‐level expression and its application in the preparation of partially hydrolysed apple pomace xyloglucan	Wang, Li, et al. (2022)
Xyloglucanase GH74	*Thielavia terrestris*	TOF/TOF (**DHB**), glycans	Comparison of the roles of GH74 xyloglucanase and its CBM‐deleted variant in the degradation of xyloglucan‐rich biomass	Wang, Chen, Zhang, et al. (2022)
β‐Xylosidases GH8, GH39, and GH52	*Bacillus halodurans* C‐125	TOF/TOF, glycans	Substrate specificities toward substituted xylooligosaccharides	Teramoto et al. (2021)
α‑Xylosidase	*Aspergillus oryzae*	TOF/TOF (**DHB**), glycans	Characterization of an extracellular α‑xylosidase involved in xyloglucan degradation	Matsuzawa et al. (2022)
α‐Xylosidase 1	*Arabidopsis thaliana*	TOF (**s‐DHB**), glycans	Cell wall modifications by the enzyme shown to be required for control of seed and fruit size	Di Marzo et al. (2022)
Yeast GH30 xylanase	*Sugiyamaella lignohabitans*	TOF/TOF (**DHB**), glycans	Enzyme shown to be a glucuronoxylanase with auxiliary xylobiohydrolase activity	Šuchová et al. (2022)
**Other enzymes acting on sugars**				
Acetyl xylan esterase GELP7	*Arabidopsis thaliana*	TOF, glycans	Overexpression shown to improve saccharification efficiency	Rastogi et al. (2022)
Bifunctional feruloyl and acetyl xylan esterase	Metagenomes from beaver droppings and moose rumen	*R*‐TOF (**DHB**), glycans	Biochemical characterization and crystal structure	Hameleers et al. (2021)
Carbohydrate esterase family 16	*Arabidopsis thaliana*	TOF/TOF (**DHB**), glycans	Enzyme shown to contain fungal hemicellulose acetyl esterases with varying specificity	Venegas et al. (2022)
Chondroitin sulfate/dermatan sulfate 4‐*O*‐endosulfatase	Commercial from *squid cartilage*	*R*‐TOF (**HABA**/**TMG**_2_, (**252**), ‐ve), glycans	Investigation of mode of action	Wang, Przybylski, et al. (2021)
Galactan precursor transporter	*Mycobacterium smegmatis*	*R*‐TOF/TOF (**DHB**), glycans	An ATP‐binding cassette transporter Wzm–Wzt shown to catalyze translocation of lipid‐linked galactan across the plasma membrane in mycobacteria	Savková et al. (2021)
Heterologous invertase	Expressed in *Yarrowia lipolytica*	PNGase F, Endo H, TOF/TOF (**DHB**), HPLC, glycans, exoglycosidases	Study of the influence of *Y. lipolytica* glycosylation on the biochemical properties and oligomerization of heterologous invertase	Szymański et al. (2022)
Human Gb3/CD77 synthase	Human	TOF/TOF (**norharmane**), GSLs	One (Asn121) of the two *N*‐glycans on human Gb3/CD77 synthase shown to be expendable	Mikolajczyk et al. (2021)
Lytic polysaccharide monooxygenase	*Eupenicillium parvum* 4‐14	TOF/TOF (**DHB**), glycans	Identification of a highly xyloglucan active enzyme that shows boosting effect on hydrolysis of complex lignocellulosic substrates	Shi, Chen, et al. (2021)
Lytic polysaccharide monooxygenases	*Coptotermes gestroi* (termite)	TOF/TOF (**DHB**), glycans	Shown not to be involved in lignocellulose digestion but might play a role in termite development	Cairo et al. (2021)
Lytic polysaccharide monooxygenases	*Pleurotus ostreatus*	TOF/TOF **(DHB**), glycans	Enhanced konjac glucomannan hydrolysis and generation of prebiotic oligosaccharides	Li, Sun, et al. (2021)
Lytic polysaccharide monooxygenases (cellulose‑active)	*Cellulomonas* species	TOF/TOF (**DHB**), glycans	Identification and characterization of four lytic polysaccharide monooxygenases	Li, Solhi, et al. (2021)
Lytic polysaccharide monooxygenase *Tg*AA11	*Trichoderma guizhouense* NJAU 4742	TOF/TOF (**DHB**/TFA), glycans	Functional characterization of a novel copper‐dependent enzyme in the oxidative degradation of chitin	Ma, Liu, et al. (2021)
Lytic polysaccharide monooxygenase	*Podosphaera xanthii*	TOF/TOF glycans	The enzyme from the cucurbit powdery mildew pathogen *P., xanthii* contributes to the suppression of chitin‐triggered immunity	Polonio et al. (2021)
Lytic polysaccharide monooxygenases	*Aspergillus oryzae*	TOF, glycans	Comparison of C4‐oxidizing and C1/C4‐oxidizing AA9 LPMOs in substrate adsorption, H_2_O_2_‐driven activity and synergy with cellulase on celluloses of different crystallinity	Chen, Zhang, Long, et al. (2021)
Lytic polysaccharide monooxygenases	*Aphanomyces astaci* (fungus)	TOF/TOF, glycans	Enzyme identified as a chitin‐specific virulence factors in “crayfish plague”	Sabbadin et al. (2021)
Lytic polysaccharide monooxygenase	*Aspergillus fumigatus* (AfAA11B)	TOF, glycans, HPAEC‐PAD	Enzyme shown to have a preference for soluble substrates and absence of monooxygenase activity	Rieder et al. (2021)
Lytic polysaccharide monooxygenase	*Cellvibrio japonicus*	TOF/TOF (**DHB**)	C‐type cytochrome shown to initiate reduction of bacterial LPMOs	Branch et al. (2021)
Lytic polysaccharide monooxygenases	*Ceriporiopsis subvermispora*	TOF/TOF (**DHB**), glycans	Identification of two C1‐oxidizing monooxygenases and demonstration of enhancement of the saccharification of wheat straw	Long et al. (2021)
Lytic polysaccharide monooxygenases	*Sordaria brevicollis*	TOF/TOF (**DHB**), glycans	Two C1‑oxidizing AA9 lytic polysaccharide monooxygenases differ in thermostability, activity, and synergy with cellulase	Zhang, Chen, Long, et al. (2021)
Lytic polysaccharide monooxygenase	*Aspergillus fumigatus*	TOF/TOF (**DHB**), glycans	Characterization of enzyme which shows functional variation among family AA11 fungal LPMOs	Støpamo et al. (2021)
Lytic polysaccharide monooxygenase	*Irpex lacteus* 254	TOF/TOF (**DHB**), glycans	Investigation of lignin degradation *via* Fenton reaction	Li, Zhao, et al. (2021)
Lytic polysaccharide monooxygenase	*Ceriporiopsis subvermispora*	TOF/TOF (**DHB**), glycans	Functional and structural characterizations	Nguyen et al. (2022)
Lytic polysaccharide monooxygenase	*Thermoascus aurantiacus*	TLC. TOF/TOF (**CMBT/DHB**)	Purification, structural characterization and identification of its C1‐ and C4‐oxidized reaction products	Yu et al. (2022)
Lytic polysaccharide monooxygenases	*Thielavia terrestris,* TtAA9F and TtAA9G, in *Trichoderma reesei*	TOF/TOF (**CMBT**, **DHB**), glycans	For development of a high‑throughput gluco‑oligosaccharide oxidase‑based HRP colorimetric method for assaying LPMO activity	Wu, Tian, et al. (2022)
Lytic polysaccharide monooxygenases	*Thermothielavioides terrestris*	*R*‐TOF/TOF (**DHB**), glycans	Comparison of six LPMOs	Tõlgo et al. (2022)
Lytic polysaccharide monooxygenase	*Natrialbaceae archaeon*	TOF/TOF (**DHB**), glycans	Characterization and application for chitin biodegradation	Li, Liu, et al. (2022)
Lytic polysaccharide monooxygenase cMPO2	Compost	TOF/TOF (**DHB**), glycans	Structural and functional study for the oxidative degradation of cellulose	Ma, Li, et al. (2022)
Lytic polysaccharide monooxygenase, *Ssc*LPMO10B	*Streptomyces scabies*	TOF/TOF (**DHB**), glycans from cellulose	Apparent monooxygenase activity observed in reactions without exogenously added H_2_O_2_ reflects a peroxygenase reaction	Stepnov et al. (2022)
Lytic polysaccharide monooxygenase PpAA10	*Pseudomonas putida* W619 recombinant in *E. coli*	TOF/TOF (**DHB**), glycans	Activity and substrate specificity: An ATR FTIR‐based sensitive assay using attenuated total reflection‐FT‐ICR	Serra et al. (2022)
Lytic polysaccharide monooxygenase AA15	*Tribolium castaneum* and *Locusta migratoria*	TOF/TOF, glycans	Enzyme shown to be required for efficient chitinous cuticle turnover during insect molting	Qu et al. (2022)
Lytic polysaccharide monooxygenase AA9	*Aspergillus nidulans*	TOF/TOF, glycans	Deletion of AA9 LPMO shown to impact secretome and growth on lignocellulose	Terrasan et al. (2022)
Lytic polysaccharide monooxygenase	*Cellulomonas flavigena*	TOF/TOF (**DHB**), glycans	Chitin‐active LPMOs shown to be rare in *Cellulomonas* species	Li, Goddard‐Borger, et al. (2022)
Lytic polysaccharide monooxygenase	*Chaetomium thermophilum*	TOF, glycans	Oxidation properties and synergism (In Chinese)	Xia, Liu, et al. (2022)
Lytic polysaccharide monooxygenase	*Aspergillus fumigatus*	TOF, glycans	Light shown to boost the activity of enzyme	Velasco et al. (2022)
Sucrose‐6‐phosphate hydrolase	*Lactobacillus gasseri*	TOF (**DHB**), glycans	Crystal structure and potential applications in fructan production and the food industry	de Lima et al. (2021)
Xylan *O*‐acetyltransferase 1 (XOAT1)	Recombinant and engineered	TOF (**DHB**), glycans (2‐AB)	Redesign for controlled functionalization of acetylated xylan for cell‐free polymer biosynthesis	Wang, Bharadwaj, et al. (2021)

a Format (not all items present): MALDI method (**matrix**), compounds studied (derivative) other methods.
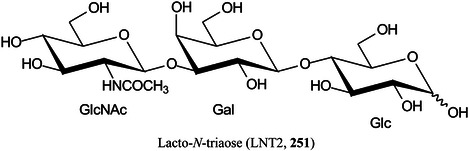
 
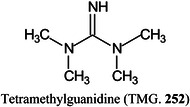

A multiplexed nanostructure‑initiator mass spectrometry (NIMS) assay has been described for simultaneously detecting glycosyl hydrolase and lignin modifying enzyme activities (Ing, et al., 2021). [*U*]‐^13^C glucose and [*U*]‐^13^C cellobiose were used as internal standards with detection by MALDI‐TOF MS.

Other work on enzymes is summarized in Table 34.

### 14.2 Medical applications

MALDI has been used extensively in medical research and is involved in the identification of biomarkers, tracking changes in glycosylation in various disease states, particularly cancer, identification of congenital disorders of glycosylation CDG), and patient monitoring. Reviews on these and other topics are listed in Table 35.

**Table 35:** Reviews and general articles on the application of matrix‐assisted laser desorption/ionization to disease.

Subject	Comments	Citations	References
**General**			
Glycan imaging mass spectrometry: Progress in developing clinical diagnostic assays for tissues, biofluids, and cells	General review: Covers instrumentation, FFPE samples, sample preparation	120	Blaschke, McDowell, et al. (2021)
Advances in understanding *N*‐glycosylation structure, function, and regulation in health and disease	Short review. Structure and function of *N*‐glycans. *N*‐Glycosylation in disease states	104	Esmail and Manolson (2021)
Clinical applications of mass spectrometry	Covers clinical applications published between 2015 and 2020 and includes glycoproteome and glycome profiling, potential biomarker and drug target discovery and characterization of therapeutic glycoproteins	268	Fang and Lu (2022)
Mass spectrometry imaging spatial tissue analysis toward personalized medicine	Several references to the use of MALDI for glycan analysis	105	Gonçalves et al. (2022)
Immunoglobulin A glycosylation and its role in disease	Mainly biology, very little on MALDI	249	Hansen et al. (2021)
Enhancing precision medicine through clinical mass spectrometry platform	General review. Emphasis on MALDI. imaging	100	Hristova and Svinarov (2022)
Mass spectrometric biosensing: A powerful approach for multiplexed analysis of clinical biomolecules	Use of compound‐specific mass tags. Much use of MALDI analysis	140	Hu, Liu, et al. (2021)
Importance of evaluating protein glycosylation in pluripotent stem cell‑derived cardiomyocytes for research and clinical applications	Contains table listing published methods where MALDI used for glycan detection	125	Kelly et al. (2021)
Progress of proteomics‐driven precision medicine: From a glycosylation view	Glycoproteomics of cancer	78	Liang, Fu, et al. (2022)
Lipids and glycolipids as biomarkers of mycobacterial infections	Emphasised advantages of MALDI in providing rapid diagnosis	124	Liu and Larrouy‐Maumus (2022)
Quantitative clinical glycomics strategies: A guide for selecting the best analysis approach	Compares performance of different methods (e.g. MALDI, LC‐MS) of released glycans and glycopeptides	111	Patabandige et al. (2022)
MALDI Mass spectrometry imaging in the clinical landscape	Use of MALDI for disease detection, disease subtyping, disease outcome prediction	188	Schwamborn (2022)
Proteomic and glyco(proteo)mic tools in the profiling of cardiac progenitors and pluripotent stem cell derived cardiomyocytes: Accelerating translation into therapy	Short section on glycomic and proteomic analysis and table listing methods for analysis of cardiac progenitor cells	170	Sebastião et al. (2021)
Glycomic technology and its application in disease marker mining	Concentrates on methods based on MALDI (In Chinese)		Shifang and Jianxin (2022)
MALDI‐TOF Mass spectrometry technology as a tool for the rapid diagnosis of antimicrobial resistance in bacteria	Identification of bacteria with small section on MS of lipid A	80	Yoon and Jeong (2021)
**Cancer**			
Lipid (and glycolipid) biomarkers for breast cancer diagnostics	Mainly lipids. A few glycolipids included	89	Bibi et al. (2022)
Glycosylation changes in prostate cancer progression	General review with references to MALDI analysis	110	Butler and Huang (2021)
Importance of glycosphingolipids on cellular and molecular mechanisms associated with epithelial‐to‐mesenchymal transition in cancer	Discusses individual compound types with section on analytical methods	139	Cumin et al. (2021)
Glycoproteogenomics: Setting the course for next‐generation cancer neoantigen discovery for cancer vaccines	Discusses cancer vaccines, glycosylation in cancer and glycomics and glycoproteomic methods	206	Ferreira et al. (2021)
Mass spectrometry‐based glycoproteomics and prostate cancer	Few direct references to MALDI	76	Gabriele et al. (2021)
Cancer glycomics offers potential biomarkers and therapeutic targets in the framework of predictive, preventive and personalized (3P) medicine	*N*‐ and *O*‐glycans. Different analytical methods – mass spectrometry, lectins, immunological, fluorescence imaging	170	Guo, Jia, et al. (2022)
Glycosylation in cancer: Its application as a biomarker and recent advances of analytical techniques	Brief review, biochemistry, analysis and use for biomarker discovery	131	Haga and Ueda (2022)
Urinary glycan biomarkers in prostate cancer	Detection of biomarkers and MALDI methods for *N*‐glycan analysis	118	Hatakeyama et al. (2021)
The repertoire of glycan alterations and glycoproteins in human cancers	Tables listing use of MALDI in clinical studies	277	Kori et al. (2021)
Molecular tissue profiling by MALDI imaging: Recent progress and applications in cancer research	Methods (instrumentation, matrices, matrix deposition, quantification), applications (identification of disease, biomarkers, drug distribution)	142	Lee, Yeoh, et al. (2021)
Glycosphingolipids in human embryonic stem cells and breast cancer stem cells, and potential cancer therapy strategies based on their structures and functions	MALDI and other analytical methods. GSLs as potential biomarkers for breast cancer	125	Liang (2022)
Blood‐based protein biomarkers in bladder urothelial tumors	Protein and glycoprotein biomarkers	246	López‐Cortés et al. (2021)
Mass spectrometry in the lipid study of cancer	Mainly concentrates on changes in lipid metabolism	165	Nabi et al. (2021)
Glycosylation alterations in cancer cells, prognostic value of glycan biomarkers and their potential as novel therapeutic targets in breast cancer	Glycans as biomarkers and use for development of targeted therapies	118	Peric et al. (2022)
Glycosylation and its research progress in endometrial cancer	Role of glycosylation, characterization of cancer biomarkers, chemotherapy	173	Pu et al. (2022)
Mass spectrometry: A powerful method for monitoring various types of leukemia, especially MALDI‐TOF in leukemia's proteomics studies	Mainly concentrates on proteins but a few references to glycoproteins and *N*‐glycans	130	Ramandi et al. (2022)
Mass spectrometry imaging in gynecological cancers: The best is yet to come	Different types of cancer, problems with imaging, types of imaging.	98	Pietkiewicz et al. (2022)
Separation based characterization methods for the *N*‐glycosylation analysis of prostate‐specific antigen	Covers MS techniques, including MALDI, LC/MS and CE/MS,	90	Reider et al. (2021)
Recent advances in mass spectrometry‐based glycomic and glycoproteomic studies of pancreatic diseases	Subjects include diabetes and cancer biomarkers	157	Tabang et al. (2021)
Causal link between immunoglobulin G glycosylation and cancer: A potential glycobiomarker for early tumor detection	Reports promising novel biomarkers for noninvasive‐cancer diagnosis, Only few MALDI references	102	Wang, Huang, et al. (2021)
MS imaging of multicellular tumor spheroids and organoids as an emerging tool for personalized medicine and drug discovery	Ionization methods, sample preparation, data analysis, quantification.	116	Wang and Hummon (2021)
Glycomic‐based biomarkers for ovarian cancer: Advances and challenges	Concentrates on *N*‐glycans. Short section on instrumentation	103	Wanyama and Blanchard (2021)
MALDI‐TOF/MS Analysis of noninvasive human urine and saliva samples for the identification of new cancer biomarkers	Mainly concentrates on proteins but some references to glycoproteins and glycopeptides	89	Zambonin and Aresta (2022)
**Aging**			
Glycosylation and aging	Discusses various changes in the aging process; little on MALDI	267	Cindrić et al. (2021)
Immunoglobulin G glycans – Biomarkers and molecular effectors of aging	Contains table listing methods (many MALDI) used for examination of changes to IgG glycosylation.	173	Krištić et al. (2022)
Glycosylation biomarkers associated with age‐related diseases and current methods for glycan analysis	Discusses various disease types with tables of application. Also analytical methods	223	Paton et al. (2021)
**Neurological disease**			
What can *N*‐glycomics and *N*‐glycoproteomics of cerebrospinal fluid tell us about Alzheimer's disease?	Discusses methods used for analysis of *N*‐glycans	112	Gaunitz et al. (2021)
Glycomic and glycoproteomic techniques in neurodegenerative disorders and neurotrauma: Towards personalized markers	Methods (enrichment, ionization, chromatography‐MS, software), applications to different diseases	377	Kobeissy et al. (2022)
MS‐based glycomics: An analytical tool to assess nervous system diseases	First section deals with techniques – MALDI, LC‐MS (permethylation), CE‐MS, ion mobility, fragmentation. Second section on applications (biomarkers) – Alzheimer's disease, Parkinson's disease, CNS disease, traumatic brain injury	311	Peng et al. (2022)
Role and therapeutic implications of protein glycosylation in neuroinflammation	Types of glycosylation, results of injury to CNS, neuroinflammation, glycodysregulations	127	Rebelo et al. (2022)
Gangliosides as biomarkers of human brain diseases: Trends in discovery and characterization by high‐performance mass spectrometry	Discusses the use of MS in different diseases.	214	Sarbu et al. (2022)
MALDI imaging mass spectrometry: An emerging tool in neurology	Discusses applications to different diseases and summarises in a table	85	Schnackenberg et al. (2022)
**Other diseases**			
Congenital disorders of glycosylation	Contains table of different diseases but with no associated references. Also incorrect *N*‐glycan structures.	24	Mendes et al. (2022)
Mass spectrometry‐based *N*‐glycosylation analysis in kidney disease	Sample preparation, general workflows, *N*‐glycosylation in specific diseases, table of applications with many MALDI references.	122	Ren, Bian, and Cai (2022)
Diagnostics of lysosomal storage diseases by mass spectrometry	Concentrates on the identification of biomarkers for various diseases	59	Pančík, Pakanová, Květoň, et al. (2022)
Recent advances and potential future applications of MALDI‐TOF mass spectrometry for identification of helminths	Causative agents of major neglected tropical diseases. Several references to the use of MALDI for glycan and glycoprotein identification.	52	Sy et al. (2022)
Structural and functional diversity of neutrophil glycosylation in innate immunity and related disorders	Comprehensive review covering various glycoproteins with notes on analytical methods	379	Ugonotti et al. (2021)
Glycosylation in autoimmune diseases	Discusses various diseases such as multiple sclerosis and rheumatoid arthritis. Little on MALDI	86	Ząbczyńska et al. (2021)

Since 2018 (MALDI‐TOF‐MS), termed MASS‐FIX, has replaced serum immunofixation for the detection and isotyping of serum monoclonal protein at Mayo Clinic Rochester campus. It offers the advantages of rapid throughput, high sensitivity and specificity for the detection of monoclonal protein, and ability to differentiate therapeutic monoclonal antibodies. It can easily identify light chain *N*‐glycosylation which has diagnostic implications, as it is more common in some disorders than others (Mellors, Dasari, et al., 2021; Kohlhagen et al., 2021). It has been used to detect the novel antibody drug conjugate Belantamab mafodotin (Mellors, Kohlhagen, et al., 2021). Since its introduction, MASS‐FIX has been used extensively. Google Scholar lists over 100 papers although few mention glycans.

#### 14.2.1 Cancer

The application of MALDI with imaging MS in cancer research, with particular emphasis on the sample preparation step, has been discussed by Buszewska‐Forajta et al. (2022). Several protocols based on cryosections and FFPE tissue were compared, taking into account the measured metabolites of potential diagnostic importance for a given type of cancer. The importance of the sample collection and storage, pretreatment protocols were emphasised and it was noted that proper preservation of tissue material should start during collection. Use of an appropriate quenching method will stop the reactive enzymatic autolysis and tissue degradation. The choice of the MALDI matrix and its method of application is also critical.

Much work has been aimed at the discovery of biomarkers. Many examples are listed in Table 36. *N*‐Glycosylation often appears significantly different in cancer patients as exemplified by a study of stage II and III colon cancer in serum and tissues. *N*‐glycosylation was generally decreased in serum whereas high‐mannose, hypogalactosylated, and tetra‐antennary glycans were overexpressed in tumor tissues (Coura et al., 2021). The quantities of multiantennary glycans were also elevated in some reports (Takei et al., 2022) as were α2→3‐linked sialic acids (Boyaval et al., 2022). Fucosylation levels have also been reported to vary. With respect to the methods that are generally used for MALDI analysis; permethylation is popular as is the preparation of linkage‐specific derivatives for sialic acids.

**Table 36:** Use of matrix‐assisted laser desorption/ionization‐mass spectrometry for examination of carbohydrate‐containing compounds in clinical studies.^a^

Disease	Medium	Methods^b^	Notes	References
**Cancer**				
Bladder cancer	Cancer cell line (150 μg)	PNGase F, TOF/TOF (**3,4‐Di‐NH**_**2**_ **‐benzophenone**) *N*‐, *O*‐glycans (per‐Me)	Identification of major differences in glycosylation and fucosyltransferase expression in low‐grade versus high‐grade bladder cancer cell lines	Ezeabikwa et al. (2021)
Bladder cancer	Cell culture	TOF/TOF (**DHB**), Free *O*‐glycans (per‐Me)	Characterization of the CD44 splicing code associated with bladder cancer invasion	Gaiteiro et al. (2022)
Bladder cancer	Urine (4 h collection)	PNGase F, TOF, *N*‐glycans	Use of lamprey immunity protein for early detection and recurrence monitoring by recognizing Neu5Gc‐modified uromodulin glycoprotein in urine	Teng et al. (2022)
Bladder cancer	FFPE tissue	PNGase F, TOF/TOF, *N*‐glycans (per‐Me)	High‐mannose H_6‐9_N_2_ and complex H_6_N_5_F_1_ increased in cancer. H_5_N_3_ (hybrid) and H_4_N_3_, H_4_N_4_ and H_6_N_5_F_1_S_2_ (complex) decreased (In Chinese)	Cheng, Sun, et al. (2022)
Brain tumors (secondary)	Metastatic tissue (4.31 mg of lipids)	TOF (**DHB**, ‐ve), GSLs	Preliminary analysis of the glycolipid profiles	Serb et al. (2022)
Breast cancer	Serum (6 μL)	PNGase F, FT‐ICR (**sDHB**), *N*‐glycans (Et ester)	Serum *N*‐glycan profiles shown to differ for various breast cancer subtypes	Vreeker et al. (2021)
Breast cancer	MCF7breast cancer cells	PNGase F, *R*‐TOF/TOF (**DHB**), *N*‐glycans	Characterization of paclitaxel resistance in breast cancer cells	Cao, Zhou, et al. (2021)
Breast cancer cells	Cancer cell line	β‐Elimination, *R*‐TOF/TOF (**DHB**), *O*‐glycans (reduced, per‐Me)	*O*‐Linked mucin‐type glycosylation shown to regulate the transcriptional programme downstream of the epidermal growth factor receptor (EGFR)	Tajadura‐Ortega et al. (2021)
Breast cancer cells	Breast cancer‐derived MCF‐7 cells (1 mg cell powder)	PNGase F, TOF/TOF (**DHB**), *N*‐ and *O*‐glycans (per‐Me)	Evaluation of the anticancer effect of violacein, phycocyanin and phycocyanobilin on apoptotic genes expression and glycan profiles	Hussein et al. (2021)
Breast cancer	Saliva (1 mL)	TOF/TOF, *N*‐glycans	Alternations of *N*‐glycans recognized by *Phaseolus vulgaris* leucoagglutinin in the saliva of patients with breast cancer	Yang, Ma, et al. (2021)
Breast cancer	Tissue (5 μM thick)	PNGase F, FT‐ICR (**CHCA**), imaging, *N*‐glycans	Clinical importance of high‐mannose, fucosylated, and complex *N*‐glycans in breast cancer metastasis	Ščupáková et al. (2021)
Breast cancer	Serum (1 μL) glycoproteins	PNGase F, (TM sprayer), (**CHCA**), *N*‐glycans	Differentiation between benign lesions or breast cancer in mammograms	Blaschke, Hill, et al. (2022)
Colon cancer	Serum (40 μL) and tissue (1 x 1 cm)	PNGase F, TOF/TOF (**DHB**), *N*‐glycans (per‐Me), LC‐MS/MS	Identification of differential *N*‐glycan compositions in the serum and tissue of colon cancer patients	Coura et al. (2021)
Colon carcinoma cell line (murine)	Murine cell line	PNGase F, *L*‐TOF/TOF (**DHB**), *N*‐glycans (2‐AB)	The solute carrier MFSD1 shown to decrease the activation status of β1 integrin and thus tumor metastasis	Roblek et al. (2022)
Colorectal cancer	Serum (10 μL) IgG	Trypsin, TOF/TOF (**DHB**), *N*‐glycopeptides	Revealing the changes of IgG subclass‐specific *N*‐glycosylation in colorectal cancer progression	Liu, Yu, et al. (2021)
Colorectal cancer	Serum (5 μL)	PNGase F, QIT‐TOF (**s‐DHB**) *N*‐glycans (reduced, Et ester)	Screening and diagnosis of colorectal cancer and advanced adenoma by Bionic Glycome method and machine learning	Pan, Zhang, Zhang, et al. (2021)
Colorectal cancer	Cell line	β‐Elimination, FT‐ICR (**s‐DHB**), *O*‐glycans (per‐Me)	As reference for development of automated method for *O*‐glycan profiling	Kotsias et al. (2021)
Colorectal cancer	FFPE tissue	Imaging, PNGase F, *R*‐TOF/TOF (**CHCA**, spray), (DMA derivatives)	Identification of high‐mannose *N*‐glycans as malignant progression markers in early‐stage colorectal cancer	Boyaval et al. (2022)
Colorectal cancer	Serum (10 μL)	PNGase F, TOF/TOF, *N*‐glycans (Me ester, Bz‐oxime)	Biomarker identification. (ratio of A1 to A2F biantennary glycans shown to be significant in detecting advanced cancer)	Takei et al. (2022)
Colorectal cancer (stage II)	FFPE tissue (6 μm sections)	PNGase F, TOF/TOF (**CHCA**, sprayer), *N*‐glycans (amide derivatization)	Cancer cells found to have higher levels of sialylation and high‐mannose glycans, less fucosylation and branching	Boyaval et al. (2021)
Endometrial cancer	FFPE tissue blocks	PNGase F (spray), *R*‐TOF/TOF (**CHCA**), *N*‐glycans	Detection of altered *N*‐linked glycosylation in endometrial cancer	Mittal et al. (2021)
Esophageal squamous cell carcinoma	Salivary (approx. 1 mL) glycoproteins	PNGase F, TOF/TOF (**DHB**), *N*‐glycans	Altered profiles in cancer patients. More complex, less fucosylation and high mannose	Shu et al. (2021)
Gastric cancer	Urinary (100 mL) exosomes	PNGase F, TOF, *N*‐glycans	Use of magnetic porous carbon‐dependent platform for the determination of *N*‐glycans from urine exosomes	Wu, Zhang, et al. (2021)
Glioblastoma	Tissue sections (5 or 10 μm sections)	Imaging, (**9‐AA**, TM sprayer), FT‐ICR, (‐ve ion), glycolipids	Discrimination between glioblastoma tumor cell subpopulations and different microvascular formations based on their lipid profiles	O'Neill, Liapis, et al. (2022)
Hepatocellular carcinoma	Tissue (imaging)	PNGase F, FT‐ICR (**CHCA**, TM sprayer), *N*‐glycans	*N*‐Glycosylation patterns shown to correlate with hepatocellular carcinoma genetic subtypes	DelaCourt et al. (2021)
Intrahepatic cholangio‐carcinoma	Human and rat tissue (approx. 25 mg)	TOF/TOF (**DHB**), GSLs (per‐Me)	Globo H shown to be a promising theranostic marker	Hung et al. (2022)
Invasive ductal carcinoma	FFPE tissue (10 μm sections)	PNGase F, TOF (**s‐DHB**), *N*‐glycans (Et ester, 2‐AA), LC‐MS/MS	Five *N*‐glycans (H_5_N_2_, H_3_N_3_F_1_, H_6_N_2_, H_7_N_2_, and H_5_N_5_F_1_) found to be significantly associated with invasive ductal carcinoma	Yaman, Kayili, et al. (2021)
Liver cancer	Hepatocellular carcinoma cells	TOF/TOF (**DHB**), glycosphingolipid	Ganglioside synthesis was increased in liver cancer	Su, Qin, et al. (2021)
Lung cancer	Serum haptoglobin	TOF, *N*‐glycans	Investigation of variation of fucose on biomarkers	Boonyapranai et al. (2021)
Lung cancer (A549)	Multicellular spheroids (14 μm sections)	*R*‐TOF (**DHB**, **CHCA**, **SA**, **THAP**, **CA** (**255**), **DMCA** (**256**), **AQ**, **HCQ** (**258**)), lipids and Glc‐Cer	Alterations of lipid metabolites in multicellular tumor spheroids in response to hydroxychloroquine revealed by imaging	Chen, Wang, et al. (2021)
Lung cancer	Cell culture	PNGase F, *R*‐TOF (**DHB**), *N*‐glycans (2‐AP)	Identification of distinct *N*‐glycosylation patterns on extracellular vesicles from small‐cell and non–small‐cell lung cancer cells	Kondo, Harada, et al. (2022)
Lung cancer	Tissue	MALDI imaging, FFPE sections	Use of high‐dimensionality reduction and clustering analysis and imaging to study metabolic heterogeneity	Conroy et al. (2022)
Melanoma	Cell line	TLC‐*R*‐TOF (**9‐AA**, ‐ve) Gangliosides and other lipids	Identification potential biomarkers in exosomes from melanoma cells with different metastatic potential	Lobasso et al. (2021)
Mucoepidermoid carcinoma	Salivary gland tissue	β ‐Elimination, TOF/TOF, QIT‐TOF (**DHB**), *O*‐glycans (per‐Me)	Characterization of tumor‐associated MUC1	Isaka et al. (2021)
Neuroblastoma	Serum (5 μL) glycoproteins	PNGase F, QIT‐TOF (**s‐DHB**), *N*‐glycans (Et esters)	Identification of possible biomarkers for neuroblastoma	Qin et al. (2021)
Oral cancer	Tissue (100 μg protein)	PNGase F, TOF/TOF (**DHB**), (Fmoc, Me‐amide)	Minor differences found in the relative abundances of eight glycans in cancer patients	Wu, Liu, et al. (2022)
Ovarian cancer	Epithelial tissue, serum (5 μL)	PNGase F, TOF/TOF (**DHB**), *N*‐glycans (per‐Me)	*N*‐Glycome changes reflecting resistance to platinum‐based chemotherapy	Zahradnikova et al. (2021)
Ovarian cancer (epithelial)	Epithelial tissue (5 μm sections)	PNGase F, imaging, TOF (**CHCA**), (DMA, spray), (sialic acid derivatization), *N*‐glycans	Identification of biomarkers	Grzeski et al. (2022)
Pancreatic cancer	Tumor lysates (approx. 1 mg protein)	Hydrazine (gas‐phase), *L*‐TOF (**DHB**), *N*‐, *O*‐glycans (**2‐AP**)	Quantitative structural analysis of glycans expressed within tumors derived from pancreatic cancer patient‐derived xenograft mouse models	Hasehira et al. (2021)
Pancreatic cancer	Tumor tissue	PNGase F or endo F3, FT‐ICR, Q‐TOF (**CHCA**, TM sprayer), amidation of sialic acids, *N*‐glycans	Imaging of *N*‐glycans, high‐mannose, bi‐, tri‐, tetra‐antennary complex. Increased sialylation in cancer tissue	McDowell et al. (2021)
Pancreatic cancer	Cell line	β‐Elimination, FT‐ICR (**s‐DHB**), *O*‐glycans (per‐Me)	As reference for development of automated method for *O*‐glycan profiling	Kotsias et al. (2021)
Pancreatic cancer	Serum (25 μL)	Orbitrap (**9‐AA**)	Lipidomic profiling of human serum enables detection of pancreatic cancer	Wolrab et al. (2022)
Pancreatic cancer	Serum (6 μL)	PNGase F, FT‐ICR (**s‐DHB**), *N*‐glycans (Et ester)	Longitudinal changes of serum protein *N*‐glycan levels may support earlier detection of pancreatic cancer in high‐risk individuals	Levink et al. (2022)
Pancreatic ductal adenocarcinoma	FFPE samples (3 μm sections)	FT‐ICR (**9‐AA**, spray), free glycans	Native glycan fragments shown to be independent prognostic factors of cancer	Sun, Trajkovic‐Arsic, et al. (2021)
Papillary thyroid cancer	Plasma	PNGase F TOF/TOF (**DHB**), *N*‐glycans, (Et ester)	To distinguish benign and malignant thyroid nodules and to identify lymph node metastasis	Zhang, Reiding, et al. (2021)
Papillary thyroid cancer	Serum (5 μL)	PNGase F, TOF (**s‐DHB**), *N*‐glycans (Et ester)	Serum linkage‐specific sialylation changes shown to be potential biomarkers for monitoring and predicting the recurrence of papillary thyroid cancer following thyroidectomy	Cao, Zhang, et al. (2022)
Papillary thyroid microcarcinoma	Serum (10 μL)	PNGase F, TOF/TOF (**DHB**), (Et‐ester/lactone), *N*‐glycans	Use of nomograms for diagnosis of papillary thyroid microcarcinoma and prediction of lymph node metastasis	Zhang, Cao, et al. (2022)
Prostate cancer	Tissue (4 μm sections)	PNGase F (imaging, TM sprayer) (**CHCA**), *N*‐glycans	Investigation of *N*‐glycans as potential biomarkers of prostate cancer. (Higher high‐mannose, tri‐ and tetra‐antennary complex)	Conroy et al. (2021)
Pseudomyxoma peritoneil (mucinous adenocarcinoma)	FFPE tissue sections	PNGase F, *R*‐TOF/TOF, *N*‐glycans (Et esters)	Detection of altered linkage pattern of *N*‐glycan sialic acids	Nummela et al. (2021)
Renal cell carcinoma	Plasma (25 μL), urine (2 mL) and tissue (25 mg)	Orbitrap (**9‐AA**, ‐ve), sulfatides	Identification of altered profiles of sulfatides and sphingomyelins in patients with renal cell carcinoma	Jirásko et al. (2022)
Thyroid cancer	Plasma (70 μL blood) IgG	PNGase F, TOF (**s‐DHB**), *N*‐glycans	Diagnostic potential of plasma IgG *N*‐glycans in discriminating thyroid cancer from benign thyroid nodules	Zhang, Wu, et al. (2021)
Various (15 types)	Tissues	PNGase F, TOF/TOF (**CHCA**, TM sprayer), high‐mannose *N*‐glycans	Re‐evaluation of previous data and re‐examination of tissues to evaluate contribution of high‐mannose *N*‐glycans to cancer	Chatterjee et al. (2021)
Various (4 types in mice)	Cancer cells (2–3 x 10^6^ cells)	PNGase F, *R*‐TOF/TOF (**DHB**), (Me esters, aoWR derivatives, “Glycoblotting” method)	Investigation of the role of the glycocalyx of tumor cell‐derived exosomes in organotropic cancer metastasis	Koide et al. (2022)
**Congenital disorders of glycosylation (CDG)**				
ALG2‐CDG	Serum (10 μL) glycopeptides	PNGase F, *R*‐TOF/TOF (**CMBT**), *N*‐glycans (per‐Me)	Characterization of ALG2‐CDG in Argentinean patients with a new genetic variant in homozygosis	Papazoglu et al. (2021)
ALG12‐CDG	Serum (10 μL) transferrin	Trypsin, MALDI	Absence of *N*‐glycans on Asn611	Hiraide et al. (2021)
ALG12‐CDG	Serum (10 μL) glycoproteins	PNGase F, *R*‐TOF/TOF (**DHB**), *N*‐glycans	A novel homozygous mutation in the human ALG12 gene found to produce an aberrant profile of high‐mannose *N*‐glycans in patient's serum (Man_5‐7_GlcNAc_2_ up, Man_8‐9_GlcNAc_2_ down)	Ziburová et al. (2021)
CAMLG‐CDG	Serum (125‐150 μL) transferrin	*R*‐TOF/TOF (**DHB**)	Identification of a novel CDG linked to defective membrane trafficking	Wilson, Durin, et al. (2022)
CDG with Golgi homeostasis disruption	Serum (5 μL) apoC‐III	PNGase F, TOF/TOF (**DHB**), *N*‐glycans (per‐Me)	ApoC‐III glycosylation used to diagnose disease when transferrin glycosylation appeared normal	Raynor, Vincent‐Delorme, et al. (2021)
COG6‐CD	Serum (10 μL)	TOF, *N*‐, *O*‐glycans (per‐Me)	Case study. badly disrupted glycosylation, under processed glycans	Cirnigliaro et al. (2022)
*DPM2* deficient CDG	Serum transferrin	TOF, *N*‐glycans	Expanding the clinical and metabolic phenotype	Radenkovic et al. (2021)
GM2 Gangliosidoses	Glycoproteins	*R*‐TOF/TOF (**DHB**), *N*‐glycans	Increased phosphorylation of HexM shown to improve lysosomal uptake and potential for managing GM2 gangliosidoses	Benzie et al. (2022)
*HNF1a* Variant and liver adenomatosis	Serum (10 μL) and serum glycoproteins (100 μL)	PNGase F, TOF/TOF (**CMBT**), *N*‐glycans (per‐Me)	Detection of highly sialylated complex glycans (two Neu5Ac per antenna)	Sturiale et al. (2021)
Leukocyte adhesion deficiency II	HEK293T and HepG2 cells	TOF, (**DHB**), *N*‐ (2‐AB) and *O*‐glycans (per‐Me), desialylated	Identification and investigation of salvage pathway. Cαaused by mutations in the SLC35C1 gene encoding Golgi GDP‐fucose transporter	Skurska et al. (2022)
MAN1B1‐CDG	Serum (5 μL)	PNGase F, Endo H, *R*‐TOF/TOF (**DHB**) *N*‐glycans (per‐Me)	Identification of disease in three individuals	Sakhi et al. (2021)
MAN1B1‐CDG	Serum transferrin	Trypsin, TOF (**DHB**), *N*‐glycopeptides	No change in glycosylation observed after disulfiram treatment	Kemme et al. (2021)
MAN1B1‐CDG	Serum transferrin	TOF (**DHB**), *N*‐glycans, HPLC	Siblings with MAN1B1‐CDG showing novel biochemical profiles	Okamoto et al. (2021)
MOGS‐CDG	IgG	MALDI, *N*‐glycans	Epilepsy and movement disorders in the oldest‐known MOGS‐CDG patient	Lo Barco et al. (2021)
MOGS‐CDG	Urine	*R*‐TOF/TOF (**DHB**), (per‐Me), HPLC (2‐AB), oligosaccharides	Clinical, biochemical and genetic characteristics of the disease	Shimada et al. (2022)
MPI‐CDG	Serum (20 μL) and serum transferrin	PNGase F, TOF/TOF (**DHB**), *N*‐glycans (per‐Me)	Variation of the serum *N*‐glycosylation during the pregnancy of a MPI‐CDG patient. Glycosylation improved	Lebredonchel et al. (2021)
PMM2‐CDG (Zebrafish embryo model)	Tissue imaging	PNGase F, Q‐TOF, *N*‐glycans	Protease‐dependent defects in N‐cadherin processing shown to drive PMM2‐CDG pathogenesis	Klaver et al. (2021)
*Slc35a1* Deficiency	Platelets	PNGase F, β‐elimination, TOF, *N*‐, *O*‐glycans (per‐Me)	*Slc35a1* Deficiency shown to cause thrombocytopenia due to impaired megakaryocytopoiesis and excessive platelet clearance in the liver	Ma, Li, Kondo, et al. (2021)
SLC35A2‐CDG	Serum transferrin	TOF	Identification of novel variant	Quelhas et al. (2021)
SLC37A4‐CDG	Serum glycoproteins	PNGase F, TOF, *N*‐glycans (per‐Me)	Mutation in SLC37A4 shown to cause a dominantly inherited CDG characterized by liver dysfunction	Ng et al. (2021)
SLC37A4‐CDG	Serum glycoproteins	Endo‐H, PNGase F, TOF/TOF (**DHB**), *N*‐glycans (per‐Me)	High‐mannose and hybrid glycans. Abnormally high Man_5_GlcNAc	Raynor, Haouari, et al. (2021)
Mild variant of leukocyte adhesion deficiency type II (SLC35C1‐CDG)	Serum *N*‐glycoproteins	PNGase F, TOF/TOF (**DHB**), *N*‐glycans (per‐Me)	Study of the response of two children to oral fucose therapy	Tahata et al. (2022)
Various	Serum transferrin and apoCIII	TOF (**DHB**), glycoproteins	Profiling of apoCIII to monitor changes in *O*‐glycosylation	Wada and Okamoto (2021)
SLC37A4‐CDG	Serum transferrin	PNGase F, TOF (**THAP**, ‐ve), *N*‐glycans	Second patient with novel variant	Wilson et al. (2021)
SLC39A8‑CDG	Serum (10 μL) transferrin	PNGase F, R‐TOF/TOF, *N*‐glycans (per‐Me)	Glycan profile showed small decrease in galactosylation	Bonaventura et al. (2021)
**Other**				
Aging heart	Tissue (20 μg protein)	PNGase F, TOF/TOF, *N*‐glycans (per‐Me)	Proposal that changes in the heart glycoproteome likely contribute to the age‐related functional decline of the cardiovascular system.	Franzka et al. (2021)
Alzheimer's disease	Plasma (3 μL) and CSF (100 μL)	*L*‐TOF/TOF, glycoproteins	Distinct patterns of apolipoprotein C‐I, C‐II, and C‐III isoforms shown to be associated with markers of Alzheimer's disease	Hu, Meuret, et al. (2021)
Alzheimer's disease	Mouse brain slices	*R*‐TOF/TOF (**NEDC**, ‐ve), sulfatide	Adult‐onset CNS myelin sulfatide deficiency shown to cause Alzheimer's disease‐like neuroinflammation and cognitive impairment	Qiu et al. (2021)
Alzheimer's disease	Serum (5 μL) and brain (20‐100 mg)	TOF/TOF, *N*‐glycans (per‐Me)	*N*‐Glycome profiling in Alzheimer's disease and Alzheimer's disease‐related dementias	Yu, Huo, et al. (2021)
Anti‐neutrophil cytoplasmic antibody‐associated vasculitis	Serum (50 μL)	R‐TOF/TOF (**9‐AA**), sulfatide	Serum sulfatide levels identified as a biomarker	Harada et al. (2022)
Anti‐PLA2R1–associated membranous nephropathy	Serum IgG4	PNGase F, TOF *N*‐glycans (sialic acid derivatization)	Altered glycosylation of IgG4 shown to promote lectin complement pathway activation in anti‐PLA2R1–associated membranous nephropathy	Haddad et al. (2021)
Behcet's disease	Serum (50 μL) glycoproteins	R‐TOF/TOF (**DHB**), *N*‐glycans	Isomer‐specific monitoring (PGC chromatography) of sialylated *N*‐glycans reveals association of α2,3‐linked sialic acid with Behcet's disease	Seo et al. (2021)
Carotid atherosclerosis in patients with rheumatoid arthritis	Serum sulfatides	TOF/TOF (‐ve)	Serum sulfatide level proposed as a predictor (biomarker) for the progression of accelerated atherosclerosis in rheumatoid arthritis cases.	Li, Yin, et al. (2022)
Chronic obstructive pulmonary disease (COPD) lung transplant patients	Plasma IgG and IgG1‐3	PNGase F, FT‐ICR (**CHCA**), *N*‐glycans	Pro‐inflammatory IgG1 *N*‐glycan signature shown to correlate with primary graft dysfunction onset in COPD patients	McQuiston et al. (2022)
Congenital aortic valve stenosis	Aortic valve tissue	PNGase F, FT‐ICR (**CHCA**, TM sprayer), *N*‐glycans	Spatial *N*‐glycomics of the human aortic valve in development and pediatric endstage congenital aortic valve stenosis	Angel, Drake, et al. (2021)
Covid‐19	Plasma (20 μL) IgG_1_, total IgG_2_, and anti‐spike IgG,	Trypsin, *R*‐TOF/TOF (**CHCA**, ‐ve), glycopeptides	Differences in glycosylation (fucosylation and galactosylation), particularly in the anti‐spike IgG	Schwedler et al. (2022)
Covid‐19	Glycated HSA hyperglycosylated IgG3 in serum	TOF (**SA**), glycated HSA	Patients recovering from Covid‐19 found to have increased levels of glycated HSA and IgG3	Iles et al. (2022)
Crohn's disease	Serum	PNGase F, TOF (“**carbon**”), *N*‐glycans	Investigation of serum *N*‐glycan patterns for rapid and precise detection of Crohn's disease	Wu, Chen, et al. (2021)
Cystic fibrosis	Broncho‐alveolar lavage fluid	β‐Elimination, *R*‐TOF, (per‐Me)	Evidence of early increased sialylation of airway mucins and defective mucociliary clearance in CFTR‐deficient piglets	Caballero et al. (2021)
Diabetic nephropathy	Rat kidney	Orbitrap, TOF/TOF (**DAN**, TM sprayer)	Identification of tissue‐specific metabolic reprogramming	Wang, Fu, et al. (2021)
Endoplasmic reticulum (ER) stress (several disease states)	HeLa cells	PNGase F, Glycoblotting, *R*‐TOF/TOF (**DHB**), *N*‐glycans (Me ester, aoWR)	For quantitative evaluation of ER stress. Increased high‐mannose and ratio of sialylated and non‐sialylated glycans	Fujitani et al. (2021)
Fatty liver disease	Mouse plasma	PNGase F, TOF (**DHB**, **THAP**), *N*‐glycans (per‐Me)	Defective lipid droplet–lysosome interaction shown to cause fatty liver disease as evidenced by human mutations in TMEM199 and CCDC115	Larsen, van den Boogert, et al. (2022)
Hereditary angioedema	Plasma (5 μL)	PNGase F, TOF/TOF (**s‐DHB**), *N*‐glycans (Et ester)	Validation of diagnostic and predictive *N*‐glycan biomarkers	Zhang, Wang, et al. (2021)
HIV	Plasma	TIMS‐TOF (**norharmane**, ‐ve), Lipid A	Variation in blood microbial LPS shown to contribute to immune reconstitution in response to suppressive antiretroviral therapy	Luo, Health, et al. (2022)
Invasive candidiasis	Serum	TOF/TOF, LTQ‐Orbitrap (**DHB**/pyridine)	Identification of fungal trehalose for the diagnosis of invasive candidiasis by mass spectrometry	Mery et al. (2022)
Isolated hyper‐prolactinaemia	Serum IgG (35 μg)	Trypsin, *R*‐TOF (**Cl‐CHCA**) glycopeptides	Altered immunoglobulin demonstrated in patients	Hirschberg et al. (2021)
Liver disease	Blood	PNGase F, FT‐ICR (**CHCA**), *N*‐glycans	For development of a comprehensive biomarker data model	Lyman et al. (2022)
Liver fibrosis	IgG from serum	PNGase F, FT/ICR (**CHCA**), *N*‐glycans	Development of biomarker	Scott et al. (2022)
Lupus nephritis	IgG (7 μg) from urine	PNGase F, *R*‐TOF/TOF (**DHB**), *N*‐glycans (per‐Me)	Presence of lupus nephritis indicated by aberrantly glycosylated IgG which elicits pathogenic signalling in podocytes	Bhargava et al. (2021)
Lyme disease	Serum (1 μL) IgG	Sialidase A, PNGase F, FT‐ICR, *N*‐glycans	Results show that during the acute phase of infection, IgG shifts its glycosylation profile to include structures that are not associated with the classic pro‐inflammatory IgG *N*‐glycan signature.	Haslund‐Gourley et al. (2022)
Meat allergy	Glycolipids and glycoproteins from rabbit erythrocytes	TOF/TOF	α‐Gal residues present on both glycolipids and glycoproteins contribute to immune response in meat‐allergic patients	Chakrapani, Fischer, et al. (2022)
Migraine	Serum IgG (10 μg)	Trypsin, *L*‐TOF (**DHB**), *N*‐glycopeptides	Bisected glycans increases but no change in fucosylation and sialylation	Xu, Wang, et al. (2022)
Nucleus pulposus (intervertebral discs)	Cell membranes	TOF/TOF (**DHB**). Identification by GlycoWorkBench	Enhancement of nucleus pulposus repair by glycoengineered (addition of unnatural sialic acids) adipose‐derived mesenchymal cells	Ying et al. (2022)
Parkinson's disease	Serum (5 μL) glycoproteins	PNGase F, TOF/TOF (**DHB**/DMA), *N*‐glycans (Et ester)	Increased abundance of glycans containing core fucose, sialic acid, and bisecting GlcNAc detected	Xu, Jin, et al. (2022)
Pediatric ulcerative colitis	Colonic aspirate (100 μg protein)	PNGase F, TOF/TOF (**DHB**), *N*‐glycans (Me‐amide derivs.)	Elevated colonic microbiota‐associated paucimannosidic and truncated *N*‐glycans	Li, Zhang, et al. (2021)
Pemphigus vulgaris (autoimmune disease after corticosteroid treatmennt)	Serum IgG (0.5 mg)	PNGase F, TOF/TOF (**DHB**), *N*‐glycans (per‐Me)	No evidence found for a correlation between the IgG *N*‐glycans profile in the active phase and in the remission phase of pemphigus	Petit et al. (2021)
Pemphigus	IgG	PNGase F, TOF/TOF (**DHB**), *N*‐glycans (per‐Me)	Changes in *N*‐glycan profile from IgG in patients treated with Rituximab (less galactosylation)	Font et al. (2022)
Plasma cell disorders	Serum	TOF, *N*‐glycans	MASS‐FIX (MALDI method) for the detection of monoclonal proteins and light chain *N*‐glycosylation in routine clinical practice: a cross‐sectional study of 6315 patients	Mellors et al. (2021)
Plasma cell disorders	Serum	PNGase F, TOF (**DHB**), *N*‐glycans (Girard's T)	Characterizing M‐protein light chain glycosylation	Miller et al. (2022)
Schizophrenia	Mouse brain regions (1 mg brain protein)	PNGase F, β‐elimination, TOF (**DHB**), *N*‐ and *O‐*glycans (per‐Me)	The schizophrenia‐associated variant in *SLC39A8* found to alter protein glycosylation in mouse brain	Mealer et al. (2022)
Seasonal allergic rhinitis	Serum (50 μL)	PNGase F, *R*‐TOF/TOF‐MS/MS (s‐**DHB**) (Et ester)	Structural changes. Several triantennary glycans decreased, tetra‐antennary increased	Yaman, Avci, et al. (2021)
Sickle cell anaemia and malaria	Red blood cell ghosts	TOF/TOF, *N*‐glycans (per‐Me)	Patches of high mannose *N*‐glycans), expressed on diseased or oxidized RBC surfaces, shown to bind the mannose receptor (CD206) on phagocytes to mediate clearance.	Cao, Antonopoulos, et al. (2021)
Splenic function	Erythrocytes	PNGase F, TOF/TOF (**DHB**), *N*‐glycans (per‐Me)	Measurement of erythrocyte membrane high‐mannose glycans to assess splenic function	Cao, Mathur, et al. (2022)
Status epilepticus	Serum (10 μL) glycoproteins	PNGase F, *R*‐TOF/TOF (**DHB**), *N*‐glycans	*N*‐glycan profiling in pilocarpine induced status epilepticus in immature rats	Kapoor et al. (2022)
Systemic lupus erythematosus	Kidney FFPE tissue,	PNGase F *R*‐TOF/TOF, *N*‐glycans (DMA/NH_2_ amidation)	Increased production of high mannose glycans as a diagnostic and prognostic biomarker	Alves et al. (2021)
Vernal and atopic kerato‐conjunctivitis	Tears (8 μL)	PNGase F, TOF/TOF (**CHCA**), *N*‐glycans (per‐Me)	Over 150 high‐mannose, bi‐, tri‐ and tetra‐antennary complex glycans. Variations in bisected and fucosylated glycans detected.	Messina, Palmigiano, Tosto, et al. (2021)

^a^ Human unless otherwise stated

^b^ Format (not all items present): Glycan release method and/or protease, MALDI method (**matrix**), compounds run (derivative), other methods.
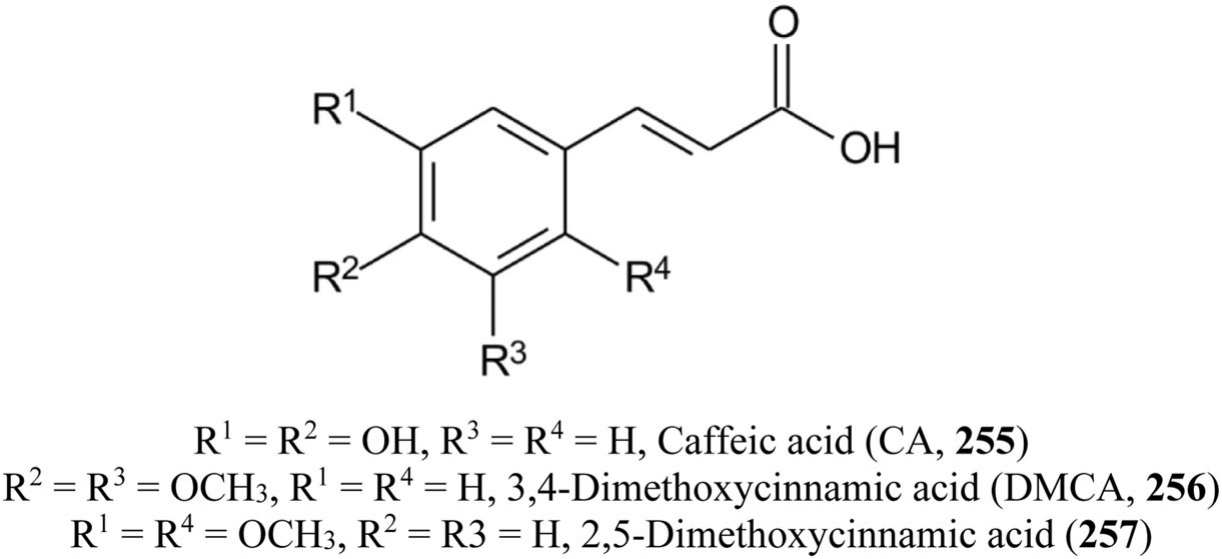
 
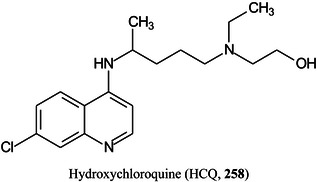

#### 14.2.2 Congenital disorders of glycosylation (CDG)

These disorders are comparatively rare but have attracted much use of MALDI mass spectrometry for their diagnosis. The diseases affect various aspects of the glycosylation of proteins and lipids and are usually diagnosed by observations of the glycosylation of serum transferrin, a glycoprotein with two *N*‐linked glycosylation sites that are normally occupied by sialylated biantennary glycans. However, recently three cases have been detected where serum transferrin glycosylation appeared normal. Diagnosis was achieved by observation of abnormally glycosylated apolipoprotein C‐III (Raynor, Vincent‐Delorme, et al., 2021).

#### 14.2.3 Biomarkers for other diseases

Some unusually large glycolipids (e.g., **253** and **254**) have been detected by negative ion MALDI‐TOF in human breath of tuberculosis patients and have been proposed as biomarkers for the disease (Mosquera‐Restrepo et al., 2022).


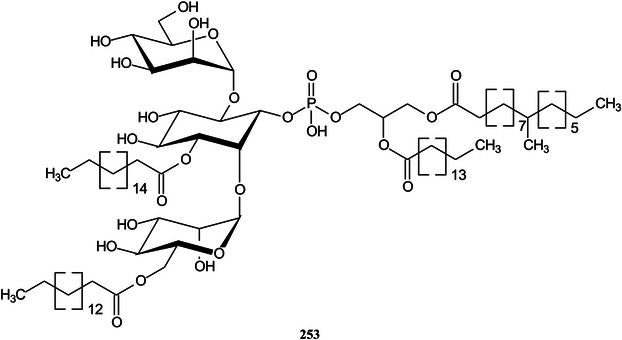



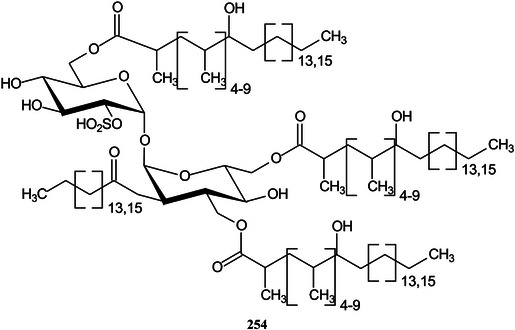


Using a solid‐phase glycoblotting technique and MALDI‐TOFMS‐based quantitative glycomics, Otaki et al. (2022) have mapped *N*‐glycosylation patterns of 16 mouse organs/tissues, serum, and serum‐derived exosomes from Slc:ddY mice. Data are presented mainly as heat maps in the main paper but full quantitative data are presented in the Supplementary information. A preliminary examination showed that machine learning analysis of the mouse lung *N*‐glycome data set enables differentiation of lungs from different mouse strains such as the outbred mouse Slc:ddY, inbred mouse DBA/2Crslc, and systemic lupus erythematosus model mouse MRL‐*lpr*/*lpr* emphasising the usefulness of a similar human organ/tissue glycome database for understanding the importance of the *N*‐glycome‐for identification of disease‐specific biomarkers.

Other applications are listed in Table 36.

### 14.3 Biopharmaceuticals

Relevant general reviews are listed in Table 37.

**Table 37:** Reviews and general articles on the analysis of biopharmaceuticals.

Subject	Comments	Citations	References
Glycoproteomics technologies in glycobiotechnology	Short sections on different techniques such as MALDI and ion mobility	92	Alagesan et al. (2021)
Characterization of glycosylation in monoclonal antibodies and its importance in therapeutic antibody development	Summarizes analytical techniques for monitoring glycosylation and effects of glycosylation on function	93	Kaur (2021)
Biopharmaceutical quality control with mass spectrometry	Recent advances, including post‐translation modifications and structural characterization	87	Liu and Schulz (2021)
State‐of‐the‐art glycomics technologies in glycobiotechnology	General review of different methods: CE, MALDI, LC‐MS, ion mobility	217	Pralow et al. (2021)
*N*‐Glycosylation of monoclonal antibody therapeutics: A comprehensive review on significance and characterization	Structure, function and analysis	180	Shrivastava et al. (2022)

#### 14.3.1 Monoclonal antibodies

Much of the work being undertaken with biopharmaceuticals is concerned with the production of monoclonal antibodies in species other than human and where glycosylation is invariably different. In some cases, such as when terminal α‐galactose residues or glycolylneuraminic acids are added, these glycans can be antigenic prompting the development of methods for their detection and great scope for genetic engineering to “humanize” the glycosylation in such nonhuman species.

Martín et al. (2021) have investigated the effect of storage on IgG Fc *N*‐glycosylation in the commonly analyzed biofluids, serum and plasma. Stability was tested by incubating samples from three healthy donors for up to 2 weeks at 50°C compared with storage at −80°C for 2 weeks. All tested IgG glycosylation features, namely sialylation, galactosylation, bisection, and fucosylation remained unchanged up to room temperature as well as during multiple freeze−thaw cycles and exposure to light. Only when subjected to 37°C or 50°C for 2 weeks, did galactosylation and sialylation subtly change.

#### 14.3.2 Vaccines

Effective vaccines against pathological bacteria can be prepared by linking expressed O‐antigen polysaccharides with specific carrier proteins. These polysaccharides are typically polydisperse, and the carrier proteins can have multiple glycosylation sites. Consequently, the resultant recombinant glycoconjugate vaccines frequently have a high structural heterogeneity, making their characterization difficult. Nicolardi et al. (2022) have addressed this problem using three glycoconjugate vaccine candidates, obtained from the bioconjugation of the O‐antigen polysaccharides from *E. coli* serotypes O2, O6A, and O25B with the genetically detoxified exotoxin A from *Pseudomonas aeruginosa*. They used MALDI‐ISD FT‐ICR MS to analyse protein and glycan ISD fragment ions which were selectively detected using DAN and s‐DHB respectively. MS/MS analysis of O‐antigen ISD fragments enabled detection of specific O‐repeats and fragments from the ends of the protein chain provided identification. The rapid method required only minute sample amounts and avoided the use of chemical derivatization.

Vaccine development involving conjugation of glycans to proteins such as BSA is another area where MALDI has played a major part in monitoring products and estimating the number of sugars that are attached to the protein. Relevant work is listed in Table 38. A protocol: “Oligosaccharide antigen conjugation to carrier proteins to formulate glycoconjugate vaccines employing dicarboxylic acid linkers” has been published (Smith & Guo, 2021) and the review with 199 references: “Cross‐reacting material (CRM_197_) as a carrier protein for carbohydrate conjugate vaccines targeted at bacterial and fungal pathogens” (Khatuntseva & Nifantiev, 2022) is also relevant.

**Table 38:** Use of matrix‐assisted laser desorption/ionization‐mass spectrometry to study carbohydrate‐protein conjugates.

Sugar	Protein	Methods^a^	Notes	References
Azido‐gluconolactone (**259**)	Gly‐His‐Tagged proteins	TOF	Synthesis of *N*‐terminally modified proteins	Brune et al. (2021)
Methylated rhamnan oligosaccharide	HSA	TOF	For development of vaccine against *Pseudomonas aeruginosa*	Cairns et al. (2022)
Oligomannose, from hepta‐ and nona‐high‐mannose glycans	BSA	TOF/TOF (**DHA**)	As specific HIV‐1‐neutralizing antibodies	Cattin et al. (2022)
Globo H analogs	CRM_197_	TOF (**SA**)	Chemoenzymatic synthesis for immunogenicity evaluation	Chen, Lin, et al. (2022)
Lipoteichoic acid and PS‐II polysaccharide from *Clostridium difficile*	HSA	TOF (**SA**)	To develop a vaccine against the dental pathogen *Streptococcus mutans*	Cox et al. (2021)
Glucosinolates	BSA	TOF	Use of the myrosinase‐glucosinolate system to generate neoglycoproteins	Cutolo et al. (2022)
Biantennary and high‐mannose *N*‐glycans	Bacteriophage Q*β* nanoparticles and BSA	TOF/TOF (**SA**)	Synthesis and immunological study reveals dominant antibody responses to the conserved chitobiose core	Donahue et al. (2022)
α‐d‐Rha4NFo (**260**)‐containing oligosaccharides	BSA	TOF	As potential vaccine against *Brucellosis* infection	Duncombe et al. (2022)
Saponin adjuvants and the Tn antigen plus linker (**261**)	BSA	TOF/TOF (**SA**)	Design, synthesis, and initial immunological evaluation as self‐adjuvanting glycoconjugate cancer vaccine	Fuentes, Aguinagalde, et al. (2021)
Pyruvylated‐human‐type complex *N*‐glycans	HiLyte Fluor 750‐conjugated HSA	TOF	*In vivo* imaging of fluorescent albumin modified with pyruvylated‐human‐type complex oligosaccharide reveals sialylation‐like biodistribution and kinetics	Fukuhara et al. (2022)
Tetravalent glycodendrons (αGal, βGal and/or αFuc)	BSA	TOF/TOF	Prepared by click chemistry, as ligands for bacterial lectins	Goyard et al. (2022)
Rha4NFo(1 → 2)Rha4NFo from *Brucella* sp.	BSA, CRM_197_	TOF/TOF (**SA**)	Synthesis and immunogenicity	Hao et al. (2021)
C‐3‐Substituted *N*,*N*’‐diacetyl‐lactosamine glycomimetics (**262**)	HSA	TOF	Chemoenzymatic synthesis for inhibition of cancer‐related galectin‐3	Heine et al. (2021)
3‐*O*‐Methyl‐d‐rhamnose oligosaccharide (**263**)	HSA	TOF	Synthesis and immunogenicity of a methyl rhamnan pentasaccharide conjugate from *Pseudomonas aeruginosa* A‑band polysaccharide	Jamshidi et al. (2022)
Several Lewis^a^ six‐aminohexyl glycoside (e.g., **264**)	BSA	TOF (**SA**)	Anti‐Le^a^ monoclonal antibody SPM 522 recognizes an extended Le^a^ epitope	Jegatheeswaran et al. (2022)
β‐1,2‐Mannans	HSA	TOF	As potential antifungal vaccines	Liao, Pan, et al. (2022)
Trisaccharides (**265**) related to *Bacillus anthracis*	KLH, BSA	TOF	Potential vaccine development. Induced immune response in mice	Liao, Zhuo, et al. (2022)
Man_5_GlcNAc_2_	BSA, CRM_197_	TOF/TOF (**DHB**)	Synthesis and immunological evaluation as HIV‑1 vaccine candidates	Liu, Huo, et al. (2022)
α‐Gal‐containing oligosaccharides derived from *Leishmania major* (**266**)	BSA	TOF (**SA**)	Reversed immunoglycomics used to identify α‐galactosyl‐bearing glycotopes specific for *L. major* infection	Montoya et al. (2021)
Synthetic β ‐Gal*f* ‐containing glycans	BSA	TOF (**SA**)	For specific recognition of β ‐galactofuranose‐containing glycans of synthetic neoglycoproteins by sera of chronic Chagas disease patients	Montoya et al. (2022)
O‐Antigen from *E. coli* O25B (**267**)	CRM_197_	TOF/TOF (**SA**)	For vaccine against *E. coli* O25B	Naini et al. (2022)
Pentasaccharide repeating unit of LPS derived from virulent *E. coli* O1	BSA	TOF	Synthesis of pentasaccharide and identification of a glycotope candidate of avian pathogenic *E. coli* O1	Nishi et al. (2021)
Amarogentin (glycoside) (**268**)	BSA, HSA	TOF	For development of competitive immune‐chromatographic assay	Nuntawong et al. (2021)
Structurally rigid TnThr mimic (Gal analogue)	BSA	TOF/TOF	As template for molecularly imprinted polymers. A promising tool for cancer diagnostics	Palladino et al. (2022)
Lactose and 3‐ and 2′ ‐fucosyl lactose	*Erythrina cristagalli* lectin, *Aleuria aurantia* lectin, and *Ulex europaeus* agglutinin‐I	TOF/TOF (**CHCA**)	Synthesis of photoactivable oligosaccharide derivatives from 1,2‐cyclic carbamate building blocks and study of their interaction with carbohydrate‐binding proteins	Podvalnyy et al. (2021)
*Streptococcus pneumoniae* serotype 14 capsular polysaccharide (**269**)	Adenoviral type 3 dodecahedron	Q‐TOF (**SA**)	Investigation of the use of adenovirus dodecahedron as a carrier for glycoconjugate vaccines	Prasanna et al. (2021)
Type K9 capsular polysaccharide of *Acinetobacter baumannii*	BSA, chicken ovalbumin and snail hemocyanin [KLH])	*L*‐TOF/TOF (**DHB**)	Determination of immune response	Rudenko et al. (2022)
High‐mannose and complex *N*‐glycans	CRM_197_	TOF (**SA**)	Immunogenicity evaluation of *N*‑glycans recognized by HIV broadly neutralizing antibodies	Shivatare et al. (2021)
Various	Phage pVIII protein	TOF (**SA**)	For construction of multivalent liquid glycan array	Sojitra et al. (2021)
Lipid A analog CRX‐527	Synthetic peptides	TOF, FT‐ICR (**DHB**)	Shown to enhance vaccination efficacy and tumor control	Tondini et al. (2022)
Mannose dendrimers	CRM_197_	TOF	For development of site‐specific multifunctionalization of CRM_197_ by disulfide rebridging for conjugate vaccine development	Trattnig et al. (2022)
Phenolic glycolipids *from Mycobacterium leprae*,	BSA	TOF/TOF	For development of diagnostic tests for leprosy	van Dijk et al. (2021)
Gal*f*‐β1→3‐Man‐α‐, Gal‐α1→3‐Gal*f*‐β1→3‐Man‐α‐ and Gal‐α1→6‐Gal‐α1→3‐Gal*f*‐β1→3‐Man‐α‐	BSA	TOF	For monitoring of New‐World tegumentary leishmaniasis using synthetic type‐2 glycoinositolphospholipid‐based neoglycoproteins	Viana et al. (2022)
Group A streptococcal trisaccharide	Fn, Fn2, rsScpA193 or CRM_197_	TOF (**SA**)	Investigation of best potential carrier protein for glycoconjugate vaccine development	Wang, Zhao, Zhao, et al. (2021)
GalCer	Receptor‐binding domain (RBD) of SARS‐Cov‐2	TOF/TOF (**SA**)	Shown to induce potent immunity against SARS‐CoV‑2 and its variants of concern	Wang, Wen, et al. (2022)
d‐Glycero‐β‑d‐mannoheptose phosphate	HSA	*L*‐TOF (**SA**)	For production of molecular probes	Williams et al. (2021)
9NHAc‐GD2	BSA	TOF (**SA**)	To overcome the hydrolytic instability of *O*‐acetylated‐GD2 for anticancer conjugate vaccine development	Wu, Ye, et al. (2021)
GalNAc	CRM_197_	*L*‐TOF (**SA**)	Development of a GalNAc‐tyrosine‐specific monoclonal antibody and detection of tyrosine *O* **‑**GalNAcylation	Xia, Bellomo, et al. (2022)
GM3 Glycan	BSA	TOF	Synthesis and evaluation of liposomal anti‐GM3 cancer vaccine candidates	Yin, Lu, et al. (2021)
Tn antigen	HSA, CRM_197_	TOF/TOF (**DHB**)	As part of Tn‐based three‐component cancer vaccine	Yang, Luo, et al. (2022)
Tetrasaccharide haptens from *Vibrio vulnificus* MO6‐24 and BO62316 (**270**)	CRM_197_ or BSA	IT‐TOF (**SA**)	Total synthesis of glycans and immunological evaluation of their protein conjugates	Zhang, Wang, Meng, et al. (2022)

a Format: MALDI method (**matrix**).


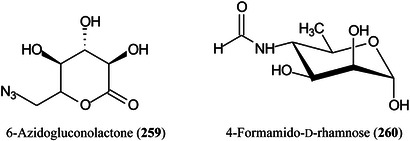



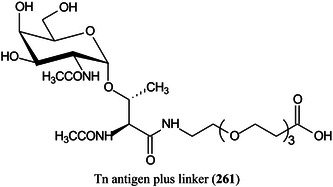



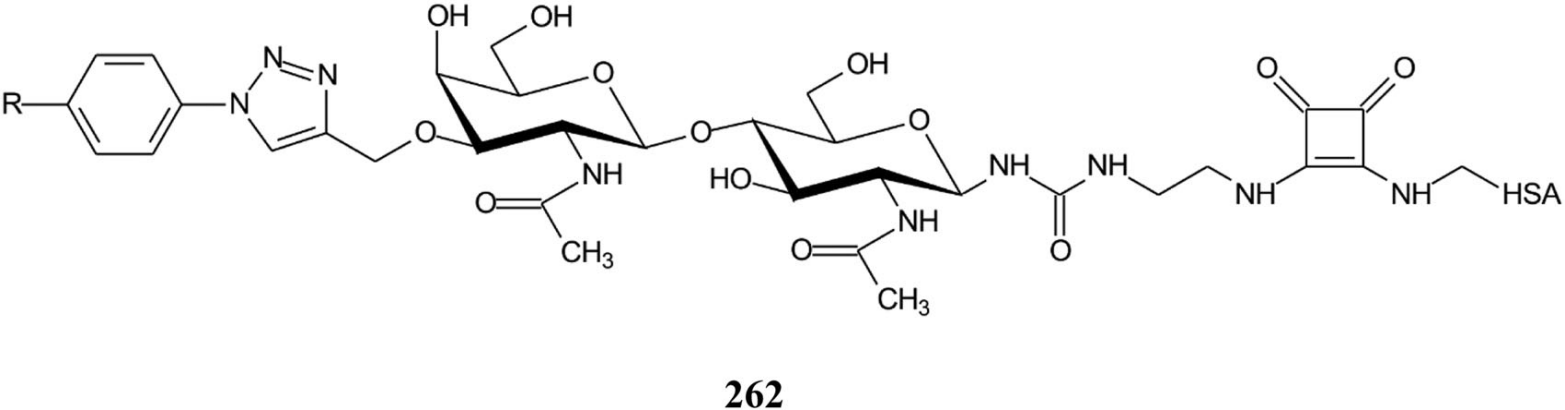



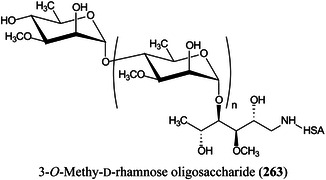



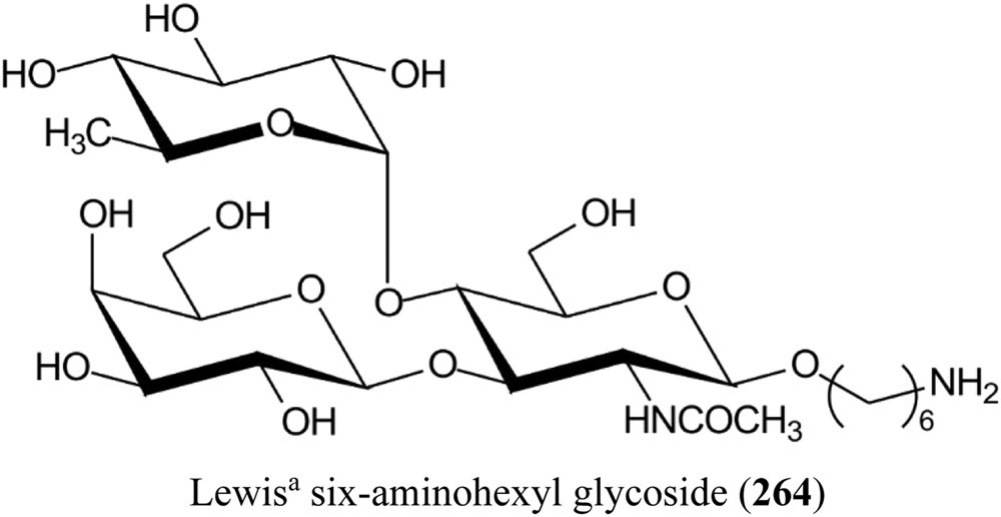



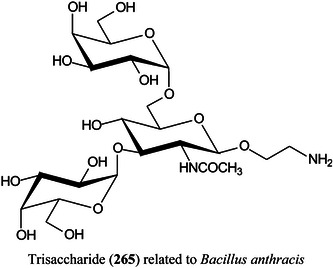



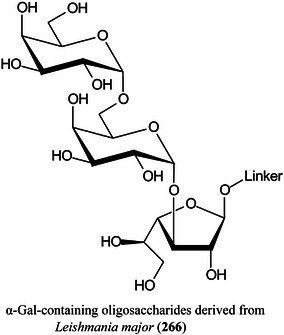



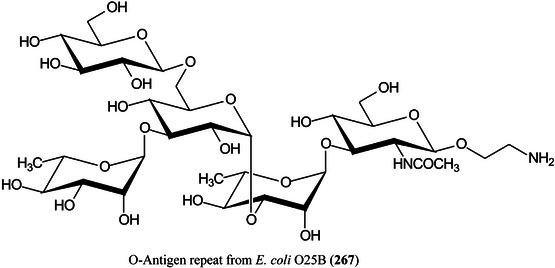



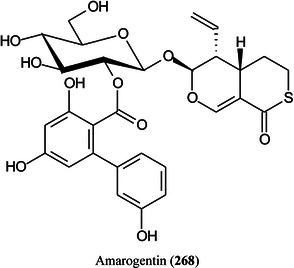



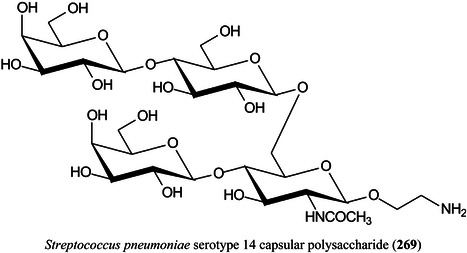



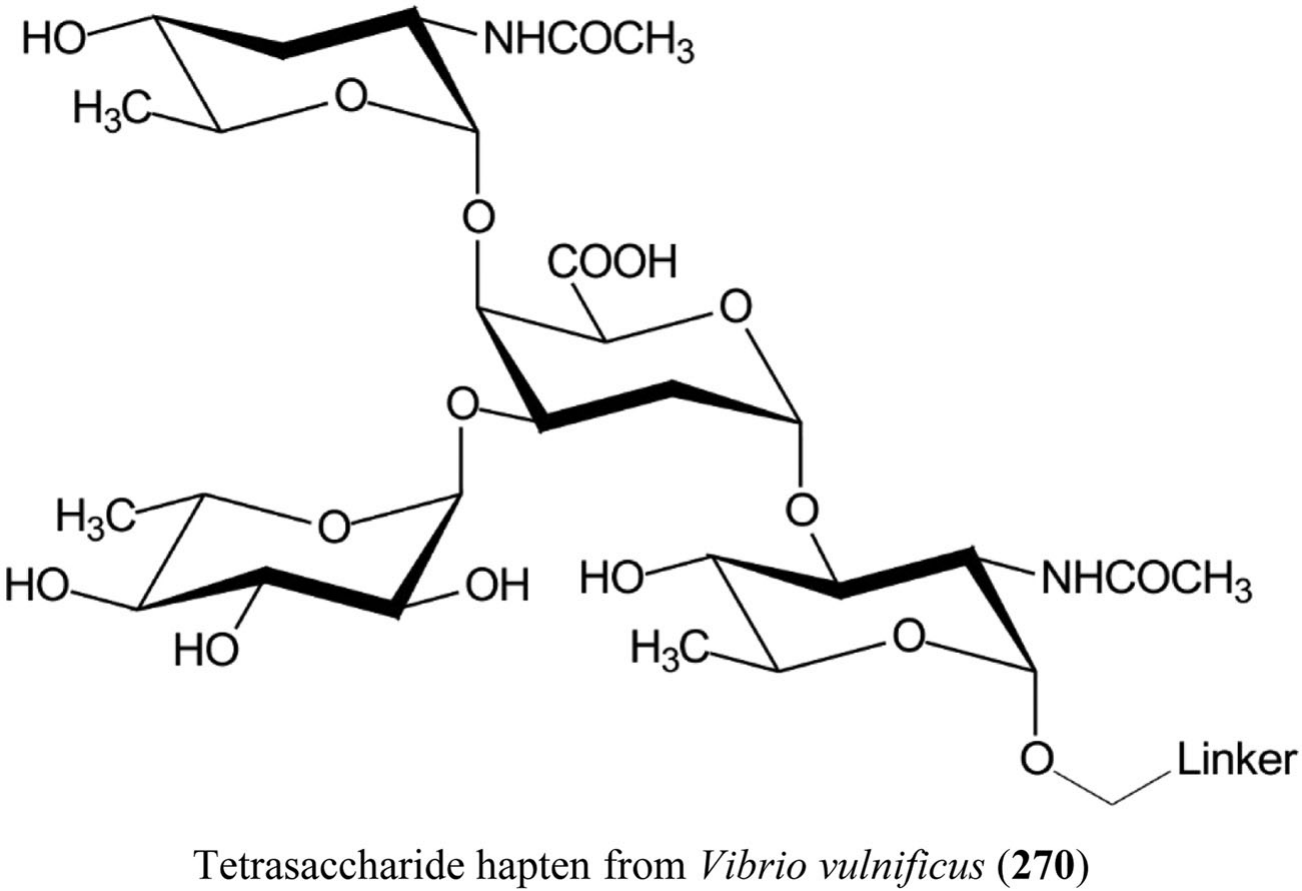


Other work in the area of biopharmaceuticals is summarized in Table 39.

**Table 39:** Use of matrix‐assisted laser desorption/ionization analysis to monitor *N*‐ and *O*‐glycosylation in biopharmaceuticals and related materials.

Biopharmaceutical and Expression System	Methods^a^	Notes	References
Arabidopsis *alg3*	TOF (2‐AP)	For production of *N*‐glycans lacking 3‐fucose and xylose substituents	Sariyatun et al. (2021)
Cell walls from *Saccharomyces cerevisiae* Δ*alg3* Δ*alg11*	PNGase F, TOF/TOF (**s‐DHB**, **THAP**), MS/MS, *N*‐glycans (per‐Me)	Production of galactosylated complex‑type *N*‑glycans in glycoengineered *S. cerevisiae*	Piirainen et al. (2022)
Colorectal cancer antigen produced in tomato fruits	Trypsin, PNGase A, TOF (**DHB**), *N*‐glycans	Immunotherapeutic effects	Park et al. (2022)
Erythropoietin (EPO)	PNGase F, *R*‐TOF/TOF (**DHB**), *N*‐glycans, (per‐Me)	Evaluation of erythropoietin biosimilars Epotin^TM^, Hemax® and Jimaixin^TM^, Comparison with original alfa drug Eprex®	Capdevilleet al. (2021)
Erythropoietin (EPO) in *Spodoptera frugiperda* cells with multiple *Mgat1* deletions	PNGase F, *R*‐TOF/TOF, *N*‐glycans	Production of a new insect cell line engineered to produce recombinant glycoproteins with cleavable *N*‐glycans	Mabashi‐Asazuma and Jarvis (2021)
Etanercept from CHO cells	PNGase F, TOF/TOF (**DHB**), *N*‐glycans (reduced) and *O*‐glycopeptides (per‐Me)	Production of an *O*‐glycovariant with enhanced potency	Biel et al. (2022)
Ectonucleotide pyrophosphatase phospodiesterase‐1	PNGase F, TOF, *N*‐glycans (per‐Me)	Improvements to the pharmacodynamics and *in vivo* activity through protein and glycosylation engineering	Stabach et al. (2021)
Human tissue plasminogen activator, Reteplase fused to IgG Fc in *Nicotiana benthamiana*	PNGase F, MALDI	Reteplase Fc‐fusions produced in *N*. *benthamiana* shown to be able to dissolve blood clots *ex vivo*	Izadi et al. (2021)
Human acid α‑glucosidase in rice cells	PNGase A, *R*‐TOF/TOF (**DHB**), (2‐AP)	Production of recombinant human acid α‑glucosidase with mannosidic *N*‑glycans	Jung (2022)
IgG (anti‐CD20 antibody, with sequence similar to Rituximab) various cell lines	PNGase F, *R*‐TOF (Et ester, *p*‐toluidine amidation)	Study of the interplay of protein engineering and glycoengineering to fine‐tune antibody glycosylation and its impact on effector functions	Wang, Wang, Zhang, et al. (2022)
IgG From CHO cells	PNGase F, *R*‐TOF/TOF (**DHB**), per‐Me	Modulation of *N*‐glycan galactosylation and fucosylation in CHO cells by feeding with galactose and fucose	Prabhu et al. (2022)
*Oryza sativa* (rice)	TOF/TOF (**DHB**), *N*‐glycans (per‐Me)	Inactivation of the β (1, 2)‑xylosyltransferase and the α (1,3)‑fucosyltransferase gene by multiplex CRISPR/Cas9 strategy	Jung, Shin, et al. (2021)
Rituximab	PNGase F, TOF/TOF (**SA**), *N*‐glycans (2‐AB)	Comparison of glycoprofiles of Rituximab versions licensed for sale in India	Kaur, Shukla, et al. (2021)
Tobacco BY‐2 cells	PNGase F, FT‐ICR (**DHB**), *N*‐glycans (2‐AB)	Inactivation of *N*‐acetylglucosaminyl‐transferase I and α1,3‐fucosyltransferase genes in *N. tabacum* BY‐2 cells shown to give glycoproteins with highly homogeneous, high‐mannose *N*‐glycans	Herman et al. (2021)
Trastuzumab and cetuximab	PNGase F, TOF/TOF (**DHB**)	Remodelling by glycan cleavage with Endo S and attaching mannose‐6‐phosphate glycan ligands for targeted protein degradation	Zhang, Liu, et al. (2022)

^a^ Format (not all items present): Glycan release method and/or protease, MALDI method (**matrix**), compounds run (derivative), other methods.

### 14.4 General Biochemistry

Applications to general biochemistry are listed in Table 40.

**Table 40:** Use of matrix‐assisted laser desorption/ionization‐mass spectrometry to study general biochemistry.

Study	Methods^a^	References
Functional glycomics and anxiety‐related behaviors in single versus group‐housed C57BL/6 and DBA/2 male mice. Shows increase in sialylated *N*‐glycans	PNGase F, TOF/TOF (**CHCA**/Di‐Et‐ammonium salt), *N*‐glycans (Me ester, BOA derivs.)	Abou‐Elnaga et al. (2021)
Fungi hijack a ubiquitous plant apoplastic endoglucanase to release a ROS scavenging β‐glucan decasaccharide to subvert immune responses	*R*‐TOF (**DHB**)	Chandrasekar et al. (2022)
Analysis of the proteome and PTMomes of C2C12 myoblasts reveals that sialylation plays a role in the differentiation of skeletal muscle cells	PNGase F, TOF/TOF (**DHB**), *N*‐glycans (per‐Me)	Chen, Sun, et al. (2021)
Characterization of the noncovalent interactions between lysozyme and panaxadiol glycosides by intensity‐fading – matrix‐assisted laser desorption ionization – mass spectrometry (IFMALDI‐MS)	*R*‐TOF (**DHB, SA**)	Du, Du, et al. (2021)
*In vitro* fermentation of chitooligosaccharides and their effects on human fecal microbial community structure and metabolites	TOF	Ji, Chang, et al. (2021)
HOIL‐1 ubiquitin ligase activity shown to target unbranched glucosaccharides and is required to prevent polyglucosan accumulation	TOF (**DHA**, NH_4_Cit) of ubiquitin‐Glc_7_	Kelsall et al. (2022)
Site‐selective chemoenzymatic modification on the core fucose of an antibody enhances its Fcγ receptor affinity and ADCC activity	TOF/TOF (**DHB/DMA**)	Li, Chong, et al. (2021)
Human gut *Faecalibacterium prausnitzii* shown to deploy a highly efficient conserved system to cross‐feed on β‐mannan‐derived oligosaccharides	TOF/TOF (**DHB**)	Lindstad et al. (2021)
Investigation of *in vitro* histone H3 glycosylation using H3 tail peptides. GlcNAcylation of histone H3 tail peptide in the presence of *O*‐GlcNAc transferase shown not to occur *in vitro*	*R*‐TOF/TOF (**CHCA**)	Merx et al. (2022)
Mechanistic studies and *in vivo* efficacy of an oxadiazole‐containing antibiotic. MALDI of Glc_2_‐diacyl glycerol (reduction of lipoteichoic acid synthesis)	TOF	Naclerio et al. (2022)
New insights into the molecular mechanism behind mannitol and erythritol fructosylation by β‑fructofuranosidase from *Schwanniomyces occidentalis*	*R*‐TOF/TOF (**DHB**)	Rodrigo‑Frutos et al. (2021)
Study on the origin of life; Investigation of the effect of proton irradiation on *N*‐glycosidic bond formation	Q‐Exactive	Saladino et al. (2021)
Effect of inhibitory mycobacterial cell wall lipids on survival of mycobacteria and their effect on the promotion of disease.	*R*‐TOF/TOF (**DHB**)	Weng et al. (2022)
Activation of regulatory T cells triggers specific changes in glycosylation associated with Siglec‐1‐dependent inflammatory responses	PNGase F, TOF (**DHB**), *N*‐glycans (per‐Me)	Wu, Murugesan, et al. (2021)

^a^ Format (not all items present): Glycan release method and/or protease, MALDI method (**matrix**), compounds run (derivative).

### 14.5 Industrial applications

Two reviews are of interest: “Algal glycobiotechnology: Omics approaches for strain improvement” (Sirohi et al., 2021), (68 references). Contains a table of metabolic studies listing analysis of *N*‐ and *O*‐glycans by MALDI; and “Comprehensive approach of methods for microstructural analysis and analytical tools in lignocellulosic biomass assessment” (Rodrigues et al., 2022), 118 references.

Several applications can be found listed in Table 12 (Polysaccharides). Others are in Table 41.

**Table 41:** Industrial and other applications.

Method/notes	Methods^a^	References
Bioactive compounds (mainly glycosides) in waste by‐products from olive oil production: Applications and structural characterization by mass spectrometry techniques	Many methods (review)	Abbattista et al. (2021)
Saccharification of cellulose‐containing raw materials using *Aspergillus niger*	TOF	Budenkova et al. (2021)
A simple procedure to obtain a medium‐size oligogalacturonic acids fraction from orange peel and apple pomace wastes	Q‐TOF (**DCTB**)	Cano et al. (2021)
Manufacturing of hemicellulosic oligosaccharides from fast‐growing Paulownia wood *via* autohydrolysis: Microwave versus conventional heating	TOF/TOF (**DHB**/TFA)	del Río et al. (2022)
Fast saccharide mapping method for quality consistency evaluation of commercial xylooligosaccharides collected in China	TOF/TOF (**DHB**)	Deng, Chen, et al. (2021)
Strategy for recycling miscellaneous waste carbohydrates from high‐fructose syrup production by *Pichia pastoris* fermentation	TOF/TOF	Gao, Duan, et al. (2021)
Fiber‐degrading enzymes released oligosaccharides in the upper gastrointestinal tract in wheat‐fed broilers to increase animal growth	TOF/TOF (**DHB**)	Kouzounis et al. (2021)
Compositional analysis of commercial galactooligosaccharide product NeoGOS‐P70	TOF/TOF (**DHB**)	Park, Eom, et al. (2021)
*Cyttaria hariotii* E. Fisch. as a promising source of pullulan and Mn(II)‐pullulan complexes for Mn‐deficiency remediation in winter cereals	TOF/TOF	Ramos‐Sánchez et al. (2021)
Antioxidant neoagarooligosaccharides (NAOs) and dietary fiber production from red algae *Gracilariopsis lemaneiformis* using an enzyme assisted one‐step process	TOF/TOF (**DHB**)	Song, Liu, et al. (2022)
Chitosan grafting *via* one‐enzyme double catalysis: An effective approach for improving performance of wool	TOF/TOF (**DHB**)	Wang, Zhang, et al. (2021)
Novel two‐step process in cellulose depolymerization: Hematite‐mediated photocatalysis by lytic polysaccharide monooxygenase and Fenton reaction	TOF (**DHB**)	Wang, Kao, et al. (2022)
Environmentally friendly chitosan adhesives for plywood bonding	*L*‐TOF (**DHB**)	Xi et al. (2022)
Efficient and green production of manno‐oligosaccharides from *Gleditsia microphylla* galactomannans using CO_2_ and solid acid in subcritical water	TOF (**DHB**)	Xu, Han, et al. (2022)
Novel immunological and mass spectrometry methods for comprehensive analysis of recalcitrant oligosaccharides in ammonia fiber expansion pretreated corn stover. Presence of methylated uronic acids	TOF, GC/MS	Xue et al. (2022)
Efficient removal of bacterial endotoxin and related risks in tailwater by dielectric barrier discharge plasma	TOF	Zhang, Wang, Zhou, et al. (2022)

^a^ Format (not all items present): MALDI method (**matrix**).

### 14.6 Food and Drink

Table 42 lists some reviews and general articles and applications are listed in Table 43.

**Table 42:** Reviews and general articles on the analysis of food and drink.

Subject	Comments	Citations	References
Recent advances in the knowledge of wine oligosaccharides	Summary of work published in the last 10 years. Origins of oligosaccharides, isolation, structure determination and dependence on grape origin	120	Apolinar‐Valiente et al. (2021)
Progress in the pretreatment and analysis of carbohydrates in food: An update since 2013	Sample preparation, analytical methods (LC, LC‐MS, MALDI, SEC, HPAEC, GC, CE, SFC)	112	Jie et al. (2021)
Biomolecular profiling by MALDI‐TOF mass spectrometry in food and beverage analyses	Analysis categorized by food type (milk and milk products, edible oils and fats, wine, beer, other foods).	104	Šebela (2022)
Recent trends in the analysis of honey constituents	Discusses various compound types (phenols, carbohydrates, amino acids and proteins, vitamins, lipids, minerals and organic acids)	120	Valverde et al. (2022)
The practice of application and features of the control of oligosaccharides in the production of specialized food products	Contains references to analysis of milk oligosaccharides by MALDI (In Russian)	59	Yurova and Ananyeva (2022)

**Table 43:** Use of matrix‐assisted laser desorption/ionization‐mass spectrometry for the characterization of carbohydrates from foods and drink.

Compound	Methods^1^	Notes	References
Noncovalent and covalent complexes between proteins and mono‐ or di‐glucoside anthocyanins	TOF/TOF (**SA**)	Effect of complexes on β‐lactoglobulin‐digestibility	Khalifa et al. (2022)
Glc_2‐13_ from *Schisandra chinensis* syrup	*R*‐TOF/TOF (**DHB**)	Synthesis and biological characterization	Kwak et al. (2022)
Pectin oligosaccharide	TOF (**graphene oxide**)	Effect of pectin oligosaccharide on quality control of quick‐frozen pumpkin puree	Li, Wang, et al. (2022)
Polysaccharides from *Glycine max* (soybean)	TOF (**DHB**)	Chemical composition and sugar spectroscopy of polysaccharides obtained by microwave‐assisted salt extraction	Li, Zhang, Chen, et al. (2022)
Maltooligosaccharides from beer	TOF (**DHB** and **PAPAN**)	Use of reactive matrix to form PAPAN derivatives (see text)	Ling, Jiang, et al. (2021)
β‐Mannans	TOF/TOF (**DHB**)	Human gut *Faecalibacterium prausnitzii* deploys a highly efficient conserved system to cross‐feed on β‐mannan‐derived oligosaccharides	Lindstad et al. (2021)
*N*‐Glycans	EndoBI‐1, rapifleX™ MALDI Tissuetyper™ (**DHB**)	Use of deglycosylated whey and chickpea protein matrices for enrichment by black mulberry polyphenols	Ozleyen et al. (2022)
Shiitake mushrooms	Q‐TOF (**DHB**)	Changes in the morphometric, textural, and aromatic characteristics of shiitake mushrooms during combined humid‐convective drying. Yield of polysaccharides, predominantly β‐glucans higher than with hot air	Subramaniama et al. (2021)
Metabolites and thymocytes from mice	PNGase F, *R*‐TOF/TOF (per‐Me)	Dietary glucosamine shown to overcome the defects in αβ‐T cell ontogeny caused by the loss of *de novo* hexosamine biosynthesis	Werlen et al. (2022)
Oligogalacturonide	TOF/TOF (**2,5‐Di‐OH‐cinnamic acid** (**257**) above)	Fungal polygalacturonase‐generated oligogalacturonide shown to restrain softening in ripening tomatoes	Yang, Lu, et al. (2022)
Shiitake mushrooms (*Lentinula eddoes*)	*R*‐TOF (**DHB**)	Analysis of glucan from chitin nanofibers prepared from Shiitake stipes	Zhang, Zhao, et al. (2022)
Pinot noir wines	TOF/TOF (**DHB**, ‐ve, +ve)	Isolation, characterization, and compositional analysis of polysaccharides	Zhu, Alcazar‐Magana, et al. (2022)

^1^ Format (not all items present): Glycan release method and/or protease, MALDI method (**matrix**), (derivative).

### 14.7 Carbohydrate synthesis

Relevant reviews are listed in Table 44.

**Table 44:** Reviews and general articles containing applications of matrix‐assisted laser desorption/ionization to carbohydrate synthesis.

Subject	Citations	References
Synthesis of cello‐oligosaccharides by depolymerization of cellulose	97	Chen, Shrotri, et al. (2021)
Chemical synthesis of cell wall constituents of *Mycobacterium tuberculosis*	347	Holzheimer et al. (2021)
Glucan phosphorylase‐catalyzed enzymatic synthesis of unnatural oligosaccharides and polysaccharides using nonnative substrates	77	Kadokawa (2022)
Ring‐opening of cyclodextrins: An efficient route to pure maltohexa‐, hepta‐, and octa‐oses	59	Pélingre et al. (2021)
Discovery of semi‐ and fully‐synthetic carbohydrate vaccines against bacterial infections using a medicinal chemistry approach	208	Seeberger (2021)
Carbohydrate‐based macromolecular biomaterials	667	Su, Feng, et al. (2021)
Chemical synthesis of polysaccharides	53	Wang, Yang, Zhu, et al. (2022)

As reported in the previous review, a large number of papers were found with the experimental details for MALDI measurements detailed in the Methods section of the paper but with no subsequent indication of its use; for example, all individual products were examined by ESI‐MS or Atmospheric pressure chemical ionization (APCI)‐MS (sometimes both) but with no details of these techniques in the “Methods” section. In several other cases, spectra were clearly acquired by ESI with the unfortunately named “MALDI‐Synapt” instrument. Many false positives were produced by computer searches for MALDI and carbohydrate names when this instrument was employed. In one publication, spectra were labelled as MALDI‐TOF spectra when, clearly, they had been obtained by ESI with this instrument. In another case, ESI spectra were said to be acquired with a MALDI‐TOF/TOF Ultraflex instrument, attributed to the wrong manufacturer and referred to as MALDI‐TOF spectra in the text. In yet another publication, samples were injected directly into a MALDI‐TOF mass spectrometer. Many papers omit to report the matrix even though the type of compound used is important to enable the analyte to “fly.” One paper reported the matrix as MeCN, H_2_O, TFA (50/49.5/0.5, v/v/v). Clearly, greater care needs to be taken with the description of methods and better reviewing is required. Needless to say, most of these papers are not cited in this review.

Synthesis of *N*‐glycans is hampered by the limited availability of functional glycoenzymes, many of which are membrane proteins that fail to express in heterologous hosts. Jaroentomeechai et al. (2022) have devised a method converting membrane‐bound glycosyltransferases into water soluble enzymes, which are expressed at high levels in the cytoplasm of living cells with retention of biological activity. Ninety eight difficult‐to‐express enzymes, predominantly of human origin, were produced and used to remodel both free and protein‐linked glycans including those found on the monoclonal antibody therapeutic trastuzumab.

#### 14.7.1 Synthesis of multivalent carbohydrates, dendrimers and glycoclusters

Two reviews are relevant: “Cu(I)‐catalyzed click chemistry in glycoscience and their diverse applications” (1331 references) (Agrahari, Bose, et al., 2021) which mainly discusses synthesis of glycodendrimers, and “Review of photoresponsive and glycoside dendrimers in biomaterials and sensors applications” (87 references) (Rajasekar et al., 2022).

Applications are listed in Table 45 with the largest compounds analysed being those shown in **271** and **280**. Compound **271** had 36 acetylated galactose residues and a molecular formula of C_768_H_876_Cl_32_N_148_O_372_, giving a calculated molecular mass of 19,240.4212. The mass found by MALDI‐TOF was 19,300 approx. (Agrahari, Jaiswal, et al., 2021). Compound **280** gave a mass of 98,900 with DHB as the matrix.

**Table 45:** Use of matrix‐assisted laser desorption/ionization‐mass spectrometry for studies on glycodendrimers and glycoclusters.

Scaffold	Sugar^a^	Methods^b^	Notes	References
*Cyclen* (1,4,7,10‐tetra‐azacyclododecane) (**271**)	Galactose OAc (12, 36)	TOF (**DHB**, **CHCA**)	CuAAC mediated synthesis	Agrahari, Jaiswal, et al. (2021)
Porphyrin (**272**)	Mannose (4)	TOF	As fluorescent sensors for Cu(II) ions. Synthesis by click chemistry	Agrahari et al. (2022)
Tris, tetrakis and hexakis‐(4‐(sulfanylmethyl) phenylacetic acid) benzene (**273**)	Fucose (3, 4, 6)	TOF/TOF (**DHB**, **CHCA**)	Used for targeting ***β*** **‑**propeller lectins from lung pathogens. Show promising anti‐adhesive properties	Duca et al. (2022)
PAMAM (**274**)	β‐Cyclodextrin (2, 4)	TOF (**DHB**)	Synthesis by copper(I)‐catalyzed alkyne–azide cycloaddition click chemistry under microwave irradiation with yields up to 99%. As potential drug carriers	González‐Méndez et al. (2022)
Tetravalent benzene (**275**)	1→2‐Di‐pseudo‐mannose (12)	TOF (**DHB**)	For DC‐SIGN targeting	Goti et al. (2022)
Linear (**276**)	Mannose (32)	TOF	Synthetic glycomacromolecules with defined valency	Hartweg et al. (2021)
Di‐COOH benzene plus two solanesol groups (**277**)	Malto‐oligosaccharides (2)	*R*‐TOF/TOF (**DHB**)	For construction of star‐shaped molecules	Isono et al. (2022)
Poly‐(propyleneimine)	Maltose (43), mannose (6)	*R*‐TOF/TOF (**DHB**)	Synthesis of nanoparticles for directed immunomodulation	Jatczak‐Pawlik et al. (2021)
PAMAM	Mannose (~110)	TOF	For study of the adherence of *Escherichia coli* 83972 on α‐biphenyl mannoside‐presenting polydimethylsiloxane surfaces	Liu, Liang, et al. (2021)
Di‐amide, tetra‐alcohol	Mannose (6, 12)	MALDI	Synthesis by photoinitiated thiol‐ene click protocol for efficient inhibition of gram‐negative bacteria	Mahadevegowda et al. (2021)
Peptide (**278**)	Galactose (4, 8)	TOF	Synthesis and evaluation as inhibitors of the adhesion of *Candida albicans*	Martin, Masterson, et al. (2021)
Cyclic decapeptide	Galactose (12, 32)	TOF	As inhibitors of the adhesion of fungal pathogen *Candida albicans* to human buccal epithelial cells	Martin, Goyard, et al. (2021)
Carbosilane (**279**)	GlcNAc (6)	TOF/TOF (**DHB, CHCA**)	Synthesis of dendritic maleimide‐thiol adducts carrying pendant glycosides as high‐affinity ligands for wheat germ agglutinin	Matsushita, Toda, et al. (2022)
PAMAM	GlcNAc and large glycans on peptides (generation 6, 231 glycopeptides)	TOF	MALDI‐TOF to study mass of released glycopeptides with *S. aureus* V8 protease	Matsushita, Hinou, et al. (2022)
Carbosilane	Galactose, glucose (4‐32)	*R*‐TOF/TOF **(DHB**)	For anticancer drug delivery: Synthetic route, characterization, and biological effect of glycodendrimer‐doxorubicin complexes	Müllerová et al. (2022)
Pentaerythritol‐peptides	Fucose (9)	TOF **(DCTB**)	Fucodendropeptides shown to induce changes in cells of the immune system in food allergic patients *via* DC‐SIGN receptor	Palomares et al. (2022)
Fullerene	Mannose (10)	TOF (**DCTB**), ESI	As EBOLA virus inhibitors	Ramos‐Soriano et al. (2022)
Dipentaerythritol	Galactose (24)	*L*‐TOF	For hepatocyte‐specific targeting and intracellular drug delivery for the treatment of liver disorders	Sharma, Porterfield et al. (2021)
PAMAM **(280**)	Galactose β1‑4 fucose (10, 24, 48, 117)	TOF (**IAA**, **DHB**)	Preparation of nanoparticles to study lectins in *Caenorhabditis elegans*	VanKoten et al. (2021)
Tris(2‐aminoethyl)amine	Sulfated mono‐, di‐ and tri‐saccharides (6)	MALDI	Synthesis of sulfoglycodendrimer therapeutics for HIV‐1 and SARS‐CoV‐2	Wells et al. (2021)
Perylene bisimide	Iminosugars (6)	MALDI	As multivalent glucosidase inhibitors	Yang, Li, et al. (2022)

^a^ Number of sugar residues in parentheses

^b^ Format (not all items present): MALDI method (**matrix**). “MALDI” used when instrument not specified.


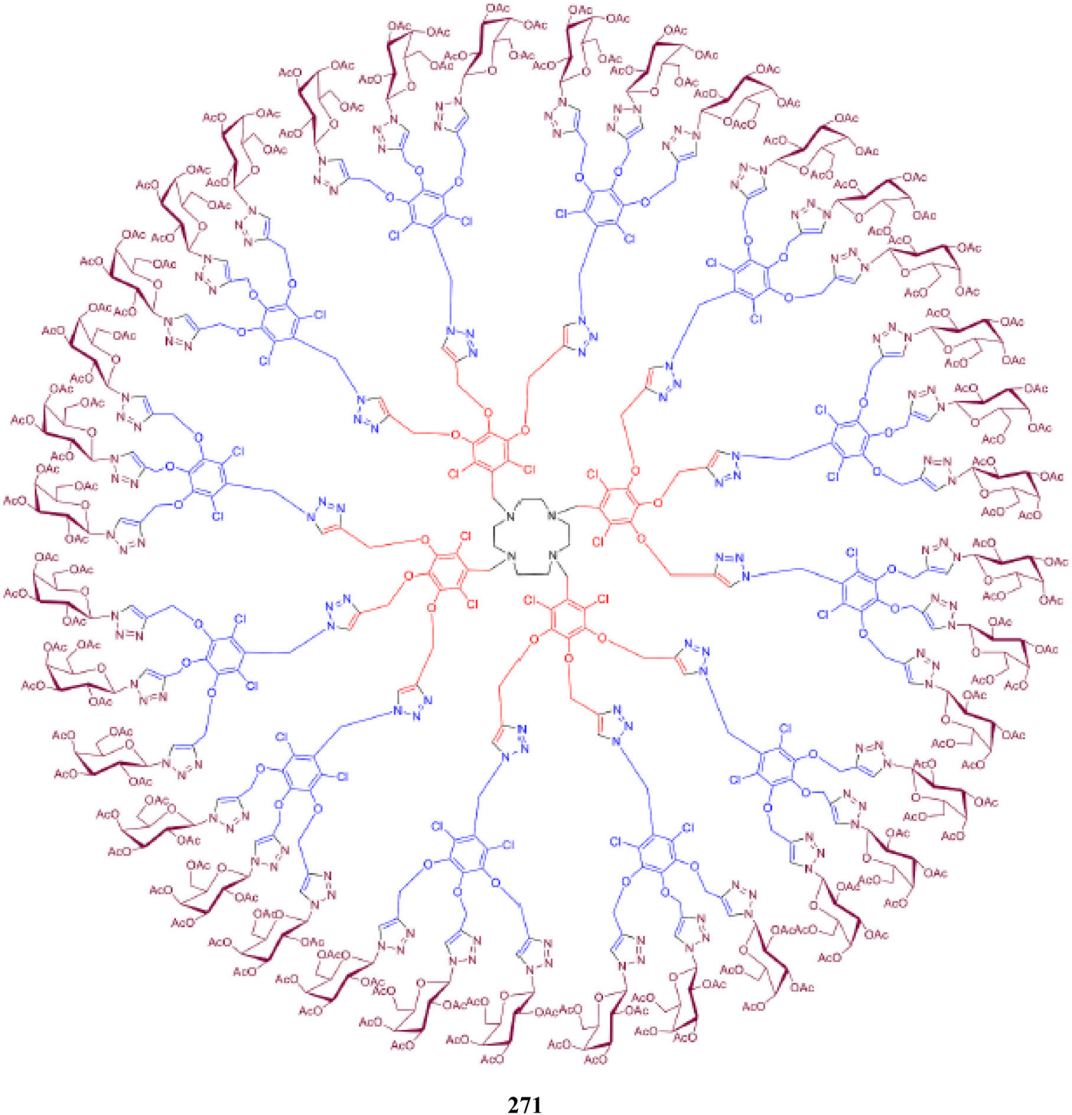



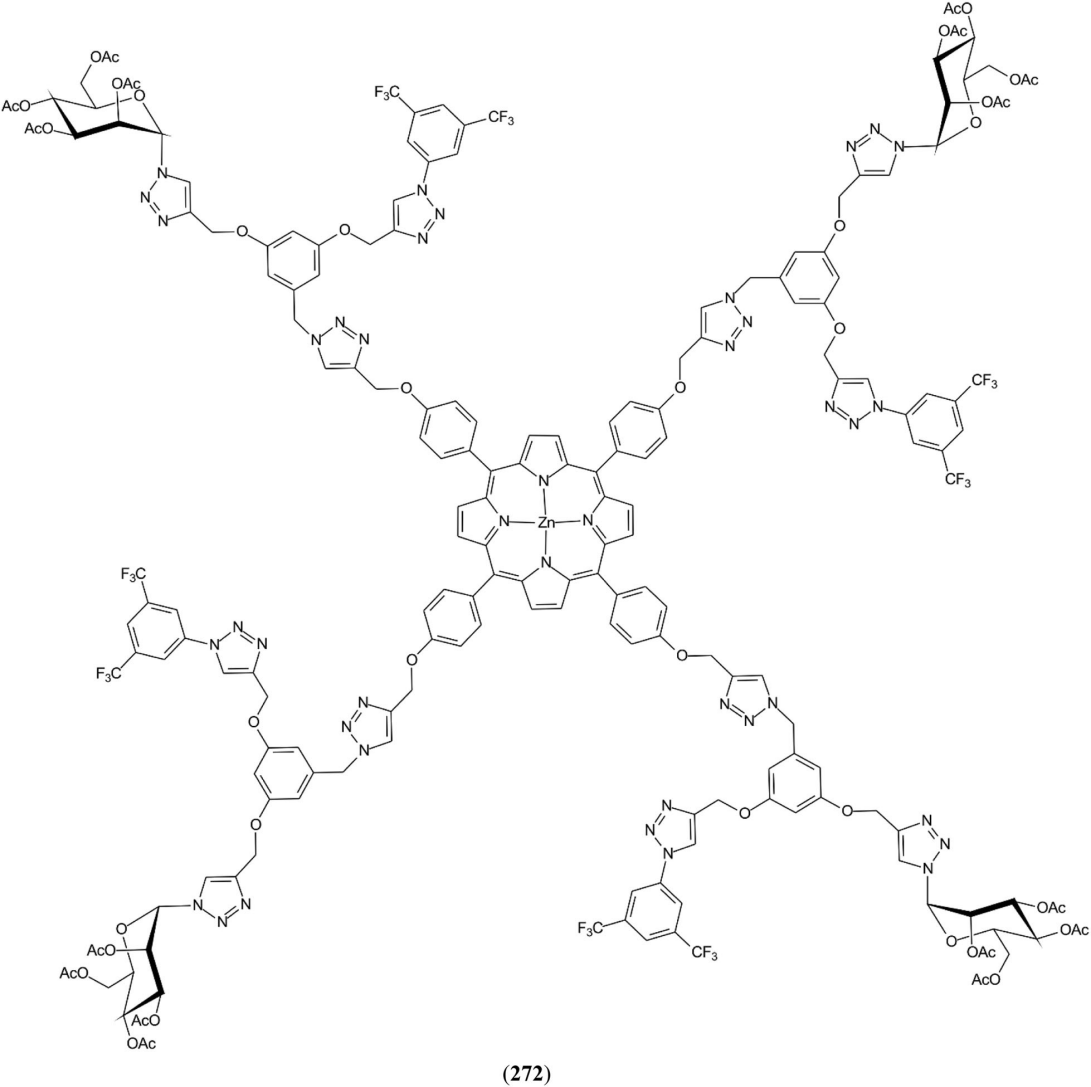



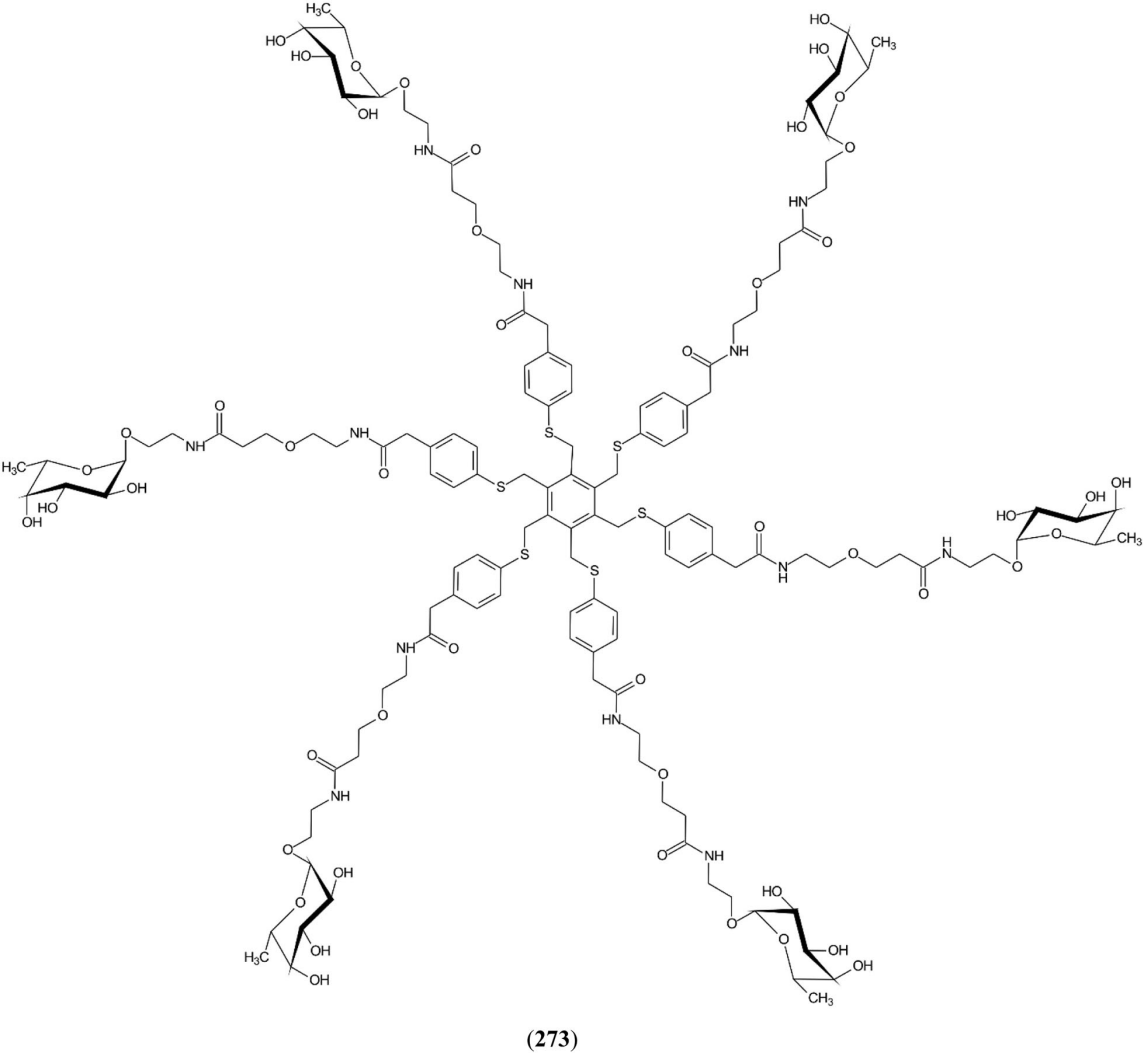



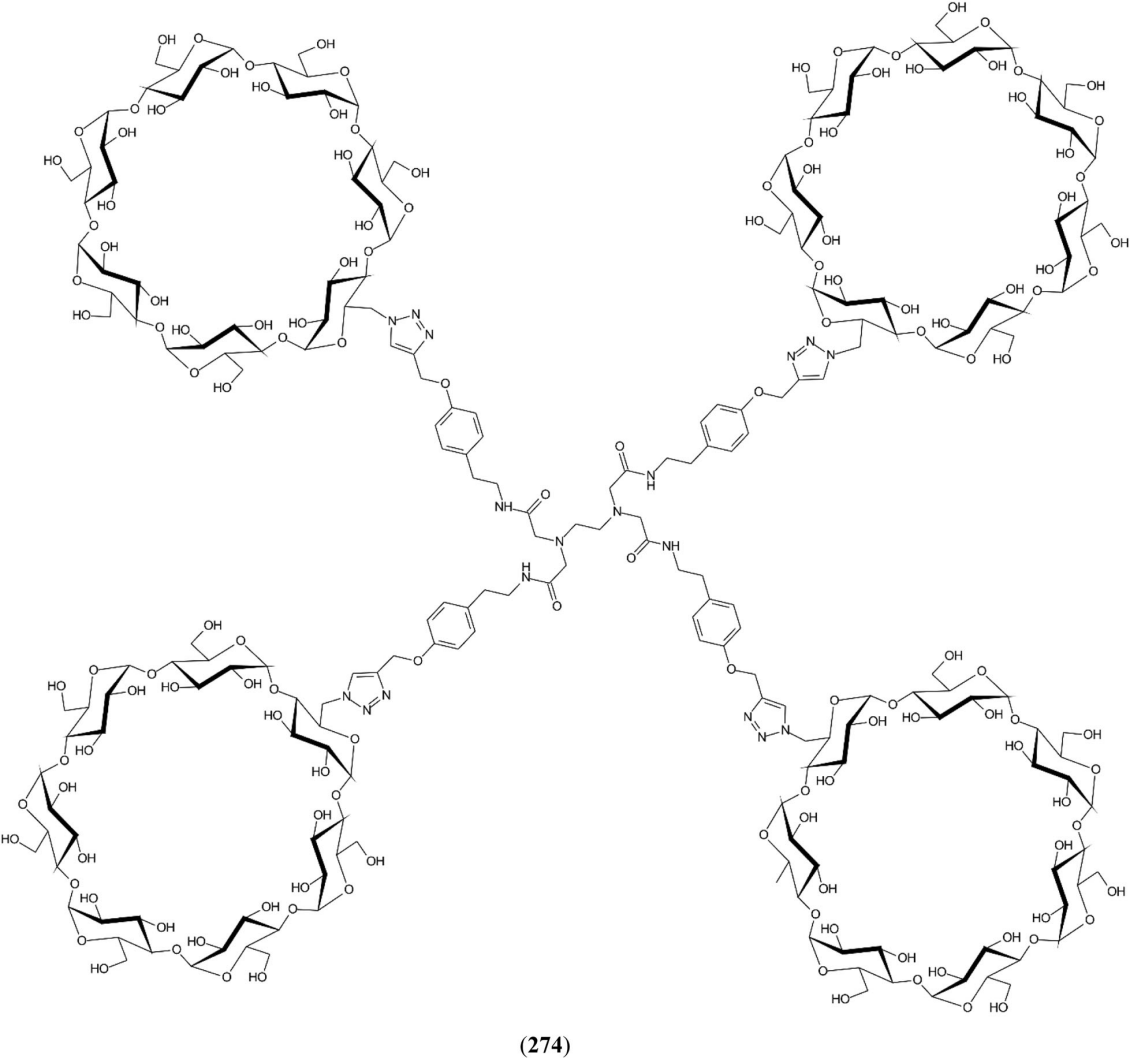



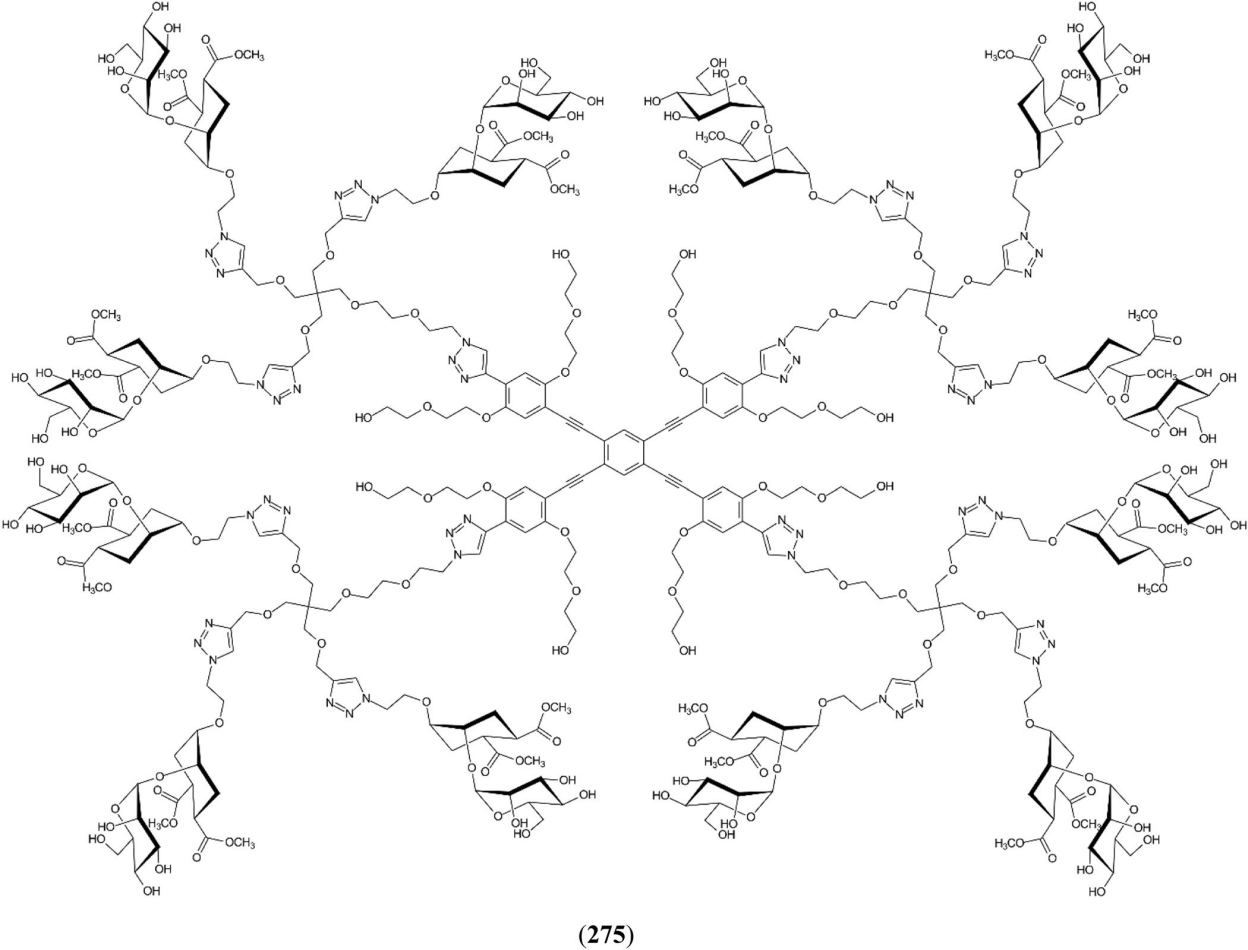



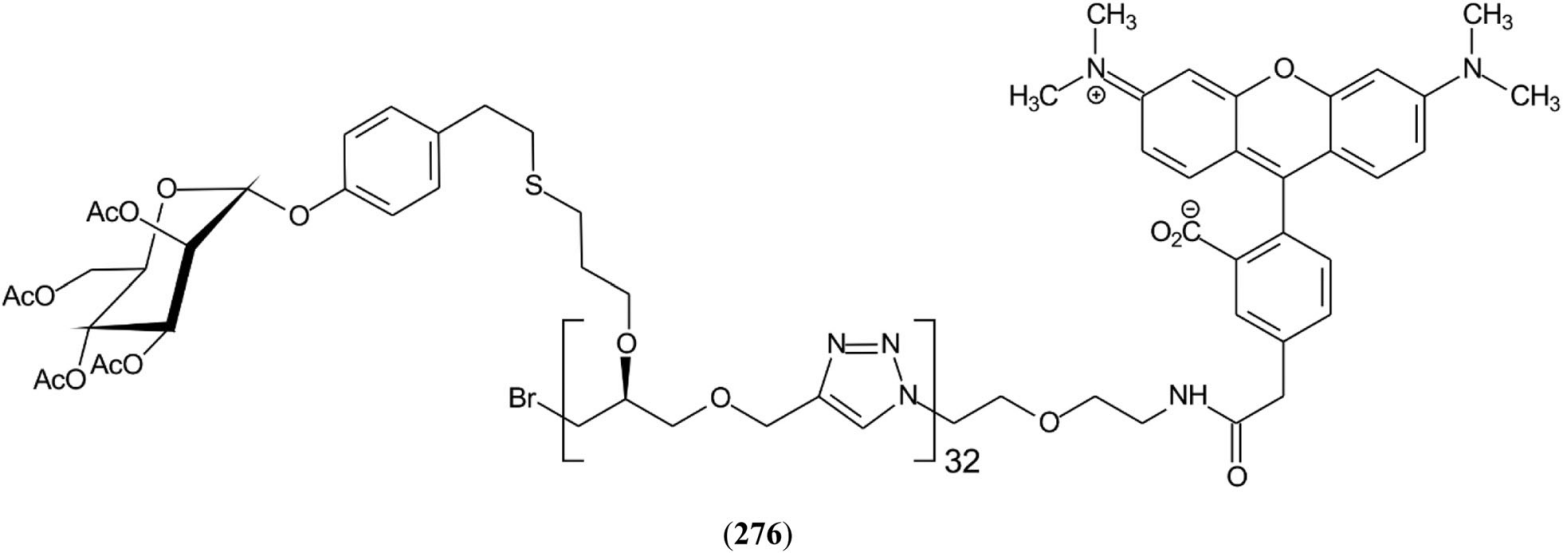



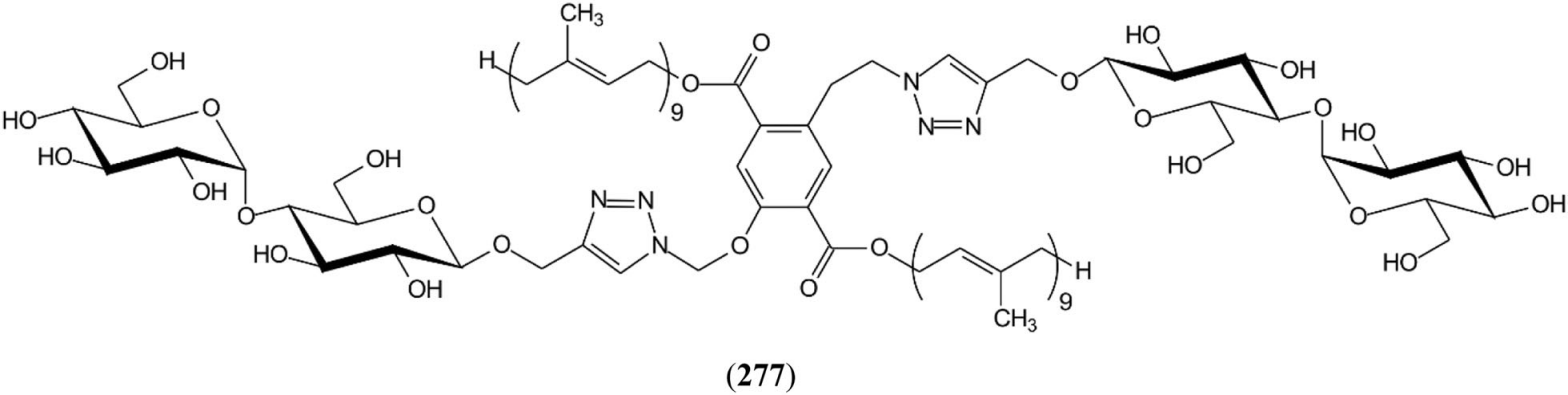



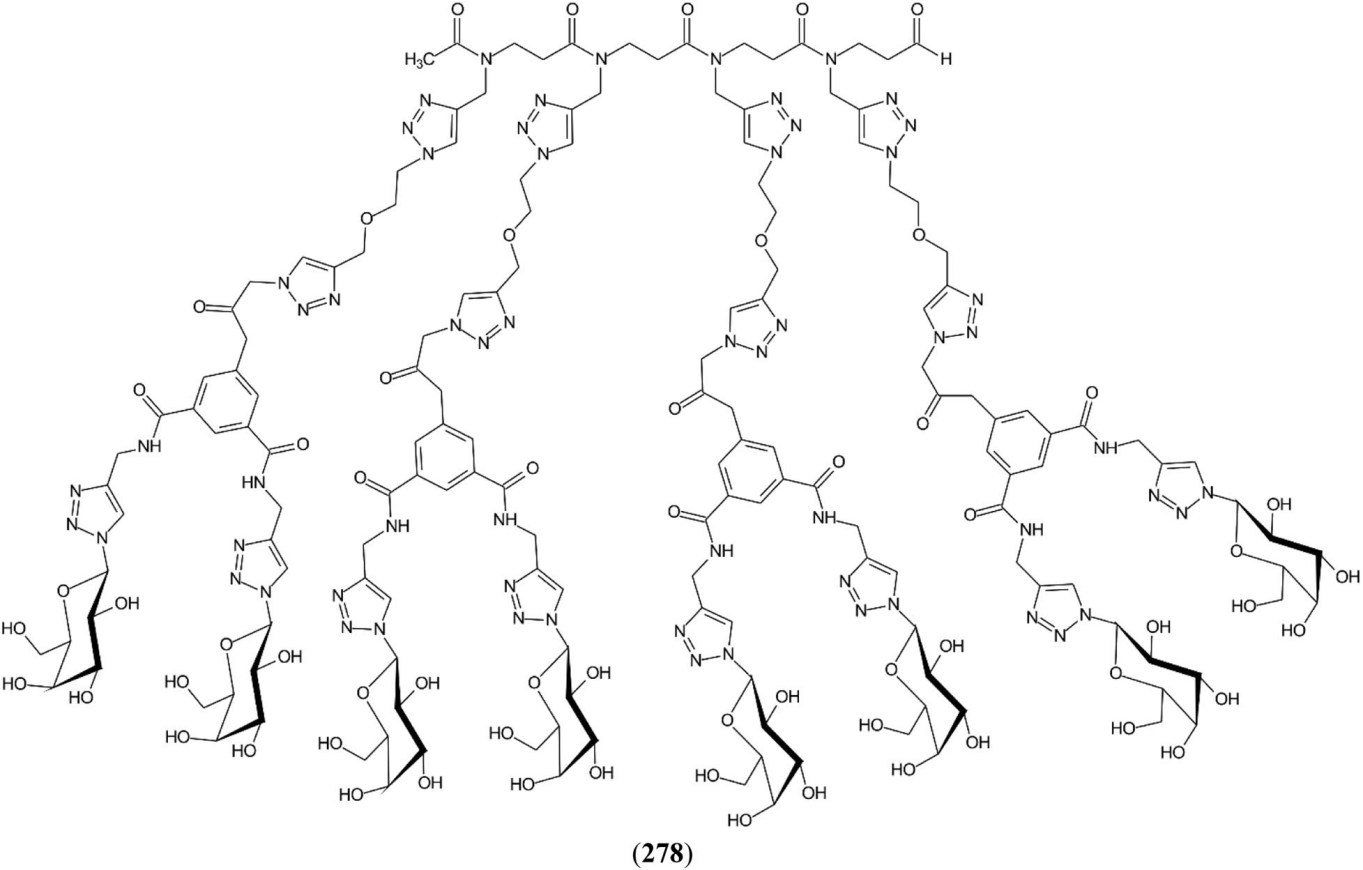



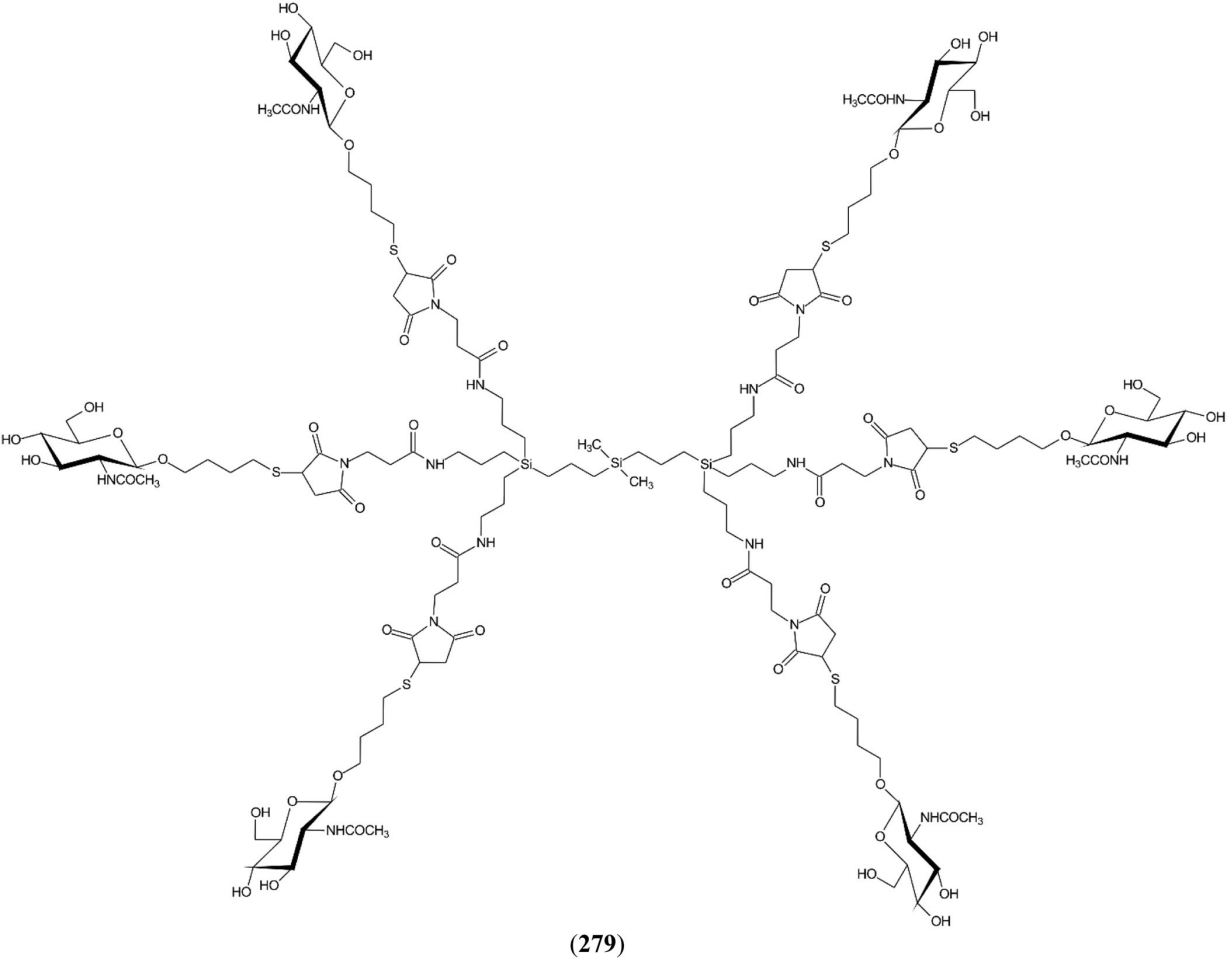



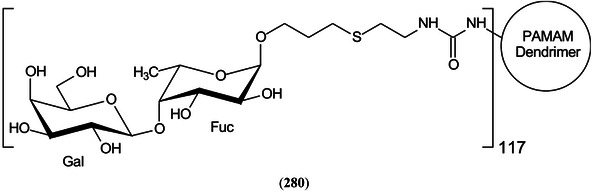


#### 14.7.2 Other synthesised compounds

Many other types of glycan or glyca,‐containing compounds have been synthesised. These are listed in Table 46.

**Table 46:** Use of matrix‐assisted laser desorption/ionization‐mass spectrometry for monitoring products of synthetic reactions.

Carbohydrate	Methods^a^	Synthetic methods and/or comments	References
**Monosaccharides**			
Many derivatized and amino acid conjugates	TOF	Synthesis of chiral acidic amino acids as tethers for intramolecular glycosylation	Fukushima et al. (2021)
Acetylated of d‐glucosamine 3‐*O*‐sulfate	TOF	For studies on lysosomal degradation of 3‐*O*‐sulfate containing heparan sulfate by arylsulfatase G	Kowalewski et al. (2021)
Oxidized trehalose	TOF	Synthesis and application as a hydrophilic anti‐crease finishing reagent for cotton fabric	Lou, Yuan, et al. (2021)
α‐d‐Ribofuranose derivatives	TOF	Synthesis, *in vivo* and *in silico* analgesic and anti‐inflammatory properties	Spriha et al. (2021)
Functionalized 4‐acetylthio‐butyl glucopyranosides	*R*‐TOF (**Dithranol**)	For studies of carbohydrate‐carbohydrate‐interactions by atomic force microscopy	Thimm et al. (2022)
**Oligosaccharides**			
(GlcNAc)_5_	TOF	For studies on the impact of HILIC amino‑based column equilibration conditions on the analysis of chitooligosaccharides	Abla et al. (2022)
*N*,*N*‐Diacetyllactosamine	TOF (**6‐ATT**)	Enzymatic synthesis. Mouse β1α4‐GalT1 wild‐type and mutant Y286L found to perform best for transferring β1→4‐Gal and β1→4‐GalNAc residues	Cao, Li, et al. (2022)
Maltose phosphate	QIT‐TOF (**DHB**)	To study polysaccharide storage in *Chlamydiae*	Colpaert et al. (2021)
Agaro‐oligosaccharides	*L*‐TOF (**DHB**)	Synthesis by microwave assisted hydrothermal hydrolysis	Dan et al. (2022)
Branched oligoglucosides	TOF	One‐step production using mutant *endo*‐β−1→3‐glucanase	Gao, Xu, et al. (2021)
Gellan oligosaccharides	TOF	Synthesis by irradiation treatment and acid hydrolysis of gellan gum	Gao, Li, et al. (2022)
Lewis b hexasaccharide thioglycoside donor	TOF	Synthesis and use towards an extended mucin core Tn heptasaccharide structure and a photoreactive biotinylated serine linked hexasaccharide	Hollinger et al. (2022)
Fe(III)‐Rhamnoxylan	*R*‐TOF (**DHB**)	Synthesis of a novel high spin Fe(iii) octahedral complex	Hayat et al. (2022)
Galβ1–3GlcNAc	TOF	In study identifying *N*‐glycans with one reducing‐terminal GlcNAc at the reducing terminus	Huang, Seino, et al. (2022)
Fluorogenic biantennary dextrins	*R*‐TOF/TOF (**DHB**)	For study of the mechanism of action of glycogen debranching enzyme	Ikeda et al. (2022)
β‐l‐Arabinofuranosyl‐l‐arabinofuranosides	TOF	Towards the substrate specificity evaluation of β‐l‐arabinofuranosidase	Ishiwata et al. (2022)
Mixed linkage trisaccharide derivatives	*R*‐TOF/TOF (**ATT**)	As endo‐β‐glucanase inhibitors	Jain et al. (2021)
Chitosan	TOF (**DHB**, **CHCA**)	By hydrolysis of high molecular weight chitosan. Antibacterial activity	Lee, Park, et al. (2022)
Short‐chain glucan oligomers	TOF/TOF	Production and separation from corn stover in an unacidified LiBr molten salt hydrate *via* pre‐extraction of hemicellulose	Liu, Zhou, et al. (2022)
6, 6’‐Carboxy trehalose and ring opened	*R*‐TOF/TOF (**DHB**)	For improving the antiwrinkle and hydrophilicity performance of cotton fabric *via* crosslinking cellulose	Lou, Wang, et al. (2021)
Cyclic α‐nigerosyl‐(1→6)‐nigerose (Ac and Me derivatives)	TOF	Derivatives to achieve complete protection	Matsuoka et al. (2022)
(LacNAc)_3_ plus ^13^C‐labels	TOF/TOF (**DHB**)	To study protein binding epitopes by NMR	Moure et al. (2021)
Keratan sulfate oligosaccharides	TOF/TOF	Use of blockwise synthesis	Ozaki et al. (2022)
Hexasaccharide from pneumococcal serotype 3 capsular polysaccharide	TOF	For studies of ligand binding of pneumococcal serotype 3 capsular polysaccharide‐specific protective antibodies	Ozdilek et al. (2021)
Methacryl‐10,2,20,3,30,4,6,60‐hepta‐*O*‐acetyl‐d‐maltose	TOF	For polymer synthesis	Palodkar et al. (2022)
Alginate oligosaccharides	TOF	Alginate oligosaccharides shown to maintain activities of lysosomes under low pH condition	Park, Nguyen, et al. (2021)
Long‐chain isomaltooligosaccharide	TOF/TOF (**DHB**)	Synthesis from maltodextrin with a novel glucosyltransferase derived from *Thermoanaerobacter thermocopriae*	Park, Park, et al. (2021)
β(1→4)‐GlcNAc oligosaccharides	TOF/TOF (**DHB**)	Size‐controlled synthesis using an endo‐glycosynthase	Rousseau et al. (2021)
Modified artichoke pectin and pectic ligosaccharides.	TOF	Optimisation of an enzymatic method using artificial neural network tools	Sabater, Blanco‐Doval, Montilla, et al. (2021)
Oligogalactofuranosides	TOF/TOF	Automated glycan assembly reveals the influence of protecting groups on oligosaccharide stability	Sabbavarapu and Seeberger (2021)
Alkyl β‐celluloside	TOF/TOF (**DHB**)	Investigation of parallel versus antiparallel molecular arrangements in crystalline assemblies	Serizawa et al. (2021)
α‐Glucosidase inhibitory oligosaccharides	TOF/TOF (**DHB**)	Preparation, structure and α‐glucosidase inhibitory by enzymatic hydrolysis from *Annona squamosa* polysaccharide	Sun, Sun, et al. (2022)
α(1→6)‐d‐Mannans and α(1→5)‐d‐arabinans	TOF	Synthesis and nitric oxide‐inducing activities	Suwanwong et al. (2022)
Xylβ1→2Manβ	TOF (**DHB**)	As the core fragment of plant *N*‐glycans	Tsygankova et al. (2022)
Chitooligosaccharides	TOF (**DHB**)	Use of plant chitinase mutants as the catalysts with sugar oxazoline derivatives	Umemoto et al. (2022)
Branched β−1→3‐glucan oligosaccharide	TOF/TOF	Synthesis by fermentation of β−1,3‐glucan producing fungi and *Trichoderma harzianum* capable of secreting *endo*‐β−1,3‐glucanase with *Sclerotium rolfsii* and *Schizophyllum commune*	Wu, Yang, et al. (2021)
Mannooligosaccharide	TOF (**DHB**)	Production from *Gleditsia microphylla* galactomannan using acetic acid and FeCl_2_	Xu, Han, et al. (2021)
Curdlan oligosaccharides	TOF/TOF (**DHB**)	Synthesis from curdlan by hydrolysis with HCl	Xu, Wang, et al. (2021)
Gellan gum oligosaccharides	TOF/TOF (**DHB**)	Synthesis from gellan by hydrolysis with HCl	Xu, Wang, et al. (2021)
Xanthan gum oligosaccharides	TOF/TOF (**DHB**)	Synthesis by treatment of xanthan gum with H_2_O_2_ for 5 days	Xu, Wang, et al. (2021)
Pullulan oligosaccharides	TOF/TOF (**DHB**)	Synthesis by hydrolysis of pullulan with pullulanase	Xu, Wang, et al. (2021)
Xylooligosaccharides	TOF/TOF (**DHB**)	Production from *Camellia oleifera* Abel fruit shell using a shell‐based solid acid catalyst	Xu, Zhang, et al. (2022)
Polysialic acid	TOF/TOF (**ATT)**	For development of photothermal therapy of neuroblastoma	Xu, Zhao, et al. (2022)
Homogalacturonan	TOF/TOF (**DHB**)	For study of binding togalectin‐3	Zheng et al. (2021)
Azide‐modified disaccharide oxazolines	TOF/TOF (**DHB**)	As enzyme substrates for single‐step Fc glycan‐mediated antibody‐drug conjugation	Zhang, Ou, et al. (2022)
**Polysaccharides**			
Bacterial cellulose nanocrystals	TOF/TOF (**DHB**)	Use of lytic polysaccharide monooxygenases and cellulases	Buruaga‐Ramiro et al. (2022)
Chitosan (from crab shells).	*L*‐TOF/TOF (**Dithranol**)	For preparation of pH‐sensitive nanoparticles loaded with dolutegravir as milk and food admixture for paediatric anti‐HIV therapy	Dharshini et al. (2021)
Fluorinated cellodextrins	TOF	Chemoenzymatic synthesis and identification of a new allomorph for cellulose‐like materials	de Andrade et al. (2021)
Quaternized and sulfated xylan‐derivatives	TOF/TOF (**s‐DHB**)	With enhanced microbiological and antioxidant properties over natural xylans	Fröhlich et al. (2022)
Oligocellulose	TOF/TOF (**DHB**)	Oligocellulose production from acid hydrolysis: A revisit	Jiang et al. (2021)
Oxidized inulin	*L*‐TOF (**CHCA**/TFA)	Use for synthesis of oxidized inulin cross‐linked collagen‐ZrO_2_ hybrid scaffolds for tissue engineering applications	Kalirajan et al. (2022)
Terminally carboxylated cellulose oligomers	TOF	For synthesis of organic−inorganic hybrid hydrogels	Sugiura et al. (2022)
Cellulose	TOF/TOF (**DHB**)	Solvent‐assisted fractionation of oligomeric cellulose and reversible transformation of cellulose II and IV	Zhang, Jiang, et al. (2021)
Reducing end thiol‐modified nanocellulose	TOF/TOF (**DHB**)	13% In two steps. For binding studies	Zhong, Zajki‐Zechmeister, et al. (2021)
Reducing‐end thiol‐modified cellulose	*R*‐TOF/TOF (**DHB**)	Use of cellodextrin phosphorylase from *Clostridium stercorarium*	Zhong, Nidetzky, (2022)
**Chitooligosaccharides**			
Sulfated chitosans	TOF/TOF (**CHCA**)	Extracted from marine waste. For evaluation of antibacterial, teratogenicity and antibiofilm effect against microorganisms	Gomathy et al. (2021)
Chitooligosaccharides	TOF/TOF (**DHB**)	Synthesis and anti‐inflammatory activity on VitD3‐induced human THP‐1 monocytes	Jitprasertwong et al. (2021)
Chitooligosaccharides	*R*‐TOF/TOF (**DHB**)	Production and characterization by the fungal chitinase Chit42 immobilized on magnetic nanoparticles and chitosan beads	Kidibule et al. (2021)
Chitooligosaccharides	TOF/TOF (**DHB**)	Production of structurally defined chito‐oligosaccharides with a single *N*‑acetylation at their reducing end using a new chitinase from *Paenibacillus pabuli*	Li, Wang, Chang, et al. (2021)
Chitooligosaccharides	TOF/TOF	Synthesis by hydrolysis of chitosan (to model chitooligosaccharides found in seawater) Action in diabetic mice.	You et al. (2022)
Chitosan (commercial)	*L*‐TOF/TOF (**DHB, CHCA, SA**)	For determination of chitosan characteristics in electrolyte solutions	Lupa et al. (2022)
Chitooligosaccharides	TOF	For preparation of chitooligosaccharide ‐polyphenol conjugates	Mittal et al. (2022)
Protected precursors of chitin oligosaccharides	TOF	Synthesis by electrochemical polyglycosylation of thioglycosides	Rahman et al. (2022)
Oligomeric chitin	TOF (**DHB**)	Efficient production of oligomeric chitin with narrow distributions of degree of polymerization using sonication‐assisted phosphoric acid hydrolysis	Zhang, Mao, et al. (2022)
**Glycosaminoglycans and related compounds**			
Non‐glycosaminoglycan‐type heparin‐analogue trisaccharides	*R*‐TOF (**DHB**)	Synthesis and cell growth inhibitory activity	Lisztes et al. (2021)
***N*** **‐linked glycans**			
BODIPY‐labelled Neu5Ac‐CMP	TOF/TOF (**9‐AA**)	Development of BODIPY labelled sialic acids as sialyltransferase substrates for direct detection of terminal galactose on *N*‐ and *O*‐linked glycans	Abukar et al. (2021)
High‐mannose‐Asn‐Fmoc	TOF/TOF (**DHB**)	For development of array for recognition of oligomannose isomers by glycan‐binding proteins involved in innate and adaptive immunity	Gao Stavenhagen et al. (2021)
Sulfated *N*‐glycans	TOF	Site‐selective sulfation of *N*‐glycans by human GlcNAc‐6‐*O*‐sulfotransferase 1 and chemoenzymatic synthesis of sulfated antibody glycoforms	Huang, Li, et al. (2022)
Decamannoside	MALDI	Binding evaluation of pradimicins for oligomannose motifs from fungal mannans	Nakagawa et al. (2021)
High‐mannose glycans	TOF	One‐pot glycosylation strategy assisted by ion mobility−mass spectrometry analysis	Ponnapalli et al. (2022)
Paucimannosidic glycans	TOF	To determine the minimal glycan recognition epitope for Mannitou IgM	Robakiewicz et al. (2021)
*N*‐Glycans from the parasite *Schistosoma mansoni*	TOF	Chemoenzymatic synthesis and examination of importance of epitope presentation on DC‐SIGN recognition	Srivastava et al. (2021)
***O*** **‐linked glycans**			
Sulfated and nonsulfated core 2 *O*‑GalNAc glycans	TOF (**DHB**)	Chemoenzymatic synthesis	Xu, Deng, Zhang, et al. (2021)
**Glycopeptides/glycoproteins**			
GM1 Glycolipid plus dodecapeptide	TOF	Ceramide structure shown to dictate glycosphingolipid nanodomain assembly and function	Arumugam et al. (2021)
MUC1 Glycopeptides	TOF (**CHCA**)	For calorimetric analysis of the interplay between synthetic Tn antigen‐presenting MUC1 glycopeptides and human macrophage galactose‐type lectin	Beckwith et al. (2021)
Glycocins	TOF/TOF (**CHCA**)	Development of SELECT‐GLYCOCIN: A recombinant microbial system for expression and high‐throughput screening of glycocins	Choudhary and Rao (2021)
Fluorine‐substituted MUC1 glycopeptide	*R*‐TOF/TOF (**DHB, SA**)	As a self‐adjuvanting antitumor nanoliposomal vaccine	Dong, Cheng, et al. (2022)
Fluorescently labelled glycopeptide (biantennary glycan)	TOF	*In vivo* imaging reveals sialylation‐like biodistribution and kinetics	Fukuhara et al. (2022)
IgG ((Fucα1, 6)GlcNAc‐rituximab or GlcNAc‐rituximab) by transglycosylation with endo‐S2	PNGase F, TOF (**2‐AA**)	For development of synthetic nanobodies as tools to distinguish IgG Fc glycoforms	Kao et al. (2022)
*N*‐Glycoproteins	TOF (**SA**)	Synthesis using a combination of genetic code expansion and chemoselective ligation techniques (click chemistry)	Hyun et al. (2021)
Evasin‐3	TOF	Use of 2,20‐dipyridyl disulfide‐mediated thiazolidine ring‐opening reaction	Katayama and Nagata (2021)
Insulin‐like androgenic gland factor from crayfish	TOF	Chemical synthesis and functional evaluation	Katayama et al. (2022)
D‐α‐Gal*p*‐l‐Ser/l‐Thr‐l‐Ala‐l‐Ala	TOF (**DHB**), FAB	As precursors of new glycopeptide antibiotics	Khodair et al. (2022)
Glycopeptide with biantennary glycan from trasnsferrin	*R*‐TOF/TOF (**CHCA**)	Screening for glycan‐specific aptamers using the glycosylated peptide as a scaffold	Li, Ma, et al. (2021)
*N*‐Glycoproteins	PNGase F, TOF, glycans (per‐Me)	Design of a new bacmid for customized protein glycosylation pathway engineering in the baculovirus‐insect cell system	Maghodia et al. (2021)
*C*‑Mannosyl tryptophan	MALDI	For quantification of serum *C*‑mannosyl tryptophan by novel assay to evaluate renal function and vascular complications in patients with type 2 diabetes	Morita et al. (2021)
*O*‐Glycopeptides (mucins)	TOF	Use of gene engineered cells	Nason et al. (2021)
*O*‐Glycopeptide (Mg7a). Major component of the venom of the ant *Myrmecia gulosa*	*L*‐TOF/TOF (**CHCA**)	Synthesis by solid‐phase peptide synthesis, combined with diselenide–selenoester ligation‐deselenization chemistry	Robinson et al. (2021)
SARS‐CoV**‑**2 homogeneous *O* **‑**linked glycopeptides	TOF	Chemoenzymatic synthesis. For exploring their inhibition functions	Rong et al. (2022)
D‑Fructose‑derived Heyns peptides	*R*‐TOF/TOF (**CHCA**)	Synthesis utilizing N^α^‑Fmoc‑Lysin‐[N^ε^‑(2‑deoxy‑D‑glucos‑2‑yl),N^ε^‑Boc]‑OH as building block	Schmutzler et al. (2021)
Clusterin glycopeptides	TOF	For development of a selective reaction monitoring approach using structure‐defined synthetic glycopeptides for validating glycopeptide biomarkers	Shiratori et al. (2022)
Amyloid‐β precursor protein with GalNAc at Tyr681	TOF (**CHCA**)	Tyrosine *O*‑GalNAc shown to alter the conformation and proteolytic susceptibility of APP model glycopeptides	Singh et al. (2021)
GalAc plus nonapeptide	TOF	As an antibiotic nano‐adjuvant to inhibit *Pseudomonas aeruginosa* biofilm and enhance antibacterial activity	Song, Zhang, et al. (2022)
[^2^H_3_]‐Methylamide labelled glycopeptides	*R*‐TOF/TOF	For quantitative method for measuring *N*‐glycopeptides	Sun, Ji et al. (2021)
*N*‐Glycopeptides with ^13^C‐fucose	*R*‐TOF/TOF (**DHB**)	For LC/MS quantitation of serum IgG glycopeptides	Wang, Liu, Qu, et al. (2021)
**Carbohydrates from bacteria**			
Lactic acid bacteria exopolysaccharides	TOF	Identification of binding sites for oligosaccharide repeats from lactic acid bacteria exopolysaccharides on bovine β‑lactoglobulin identified by NMR	Birch et al. (2021)
Lipid A mimetics	TOF (**6‐ATT**, ‐ve)	Synthesis based on an unnatural disaccharide scaffold as potent TLR4 agonists for prospective immunotherapeutics and adjuvants	Strobl et al. (2022)
Oligosaccharides derived from the capsular polysaccharide of *Streptococcus pneumoniae* serotypes 6A and 6B	TOF	Synthesis and immunological studies	Mettu et al. (2022)
**Carbohydrates from fungi**			
*Cordyceps militaris* glycans	TOF/TOF	Total synthesis *via* stereoselective orthogonal one‐pot glycosylation and *α* **‑**glycosylation strategies	Ma, Jiang, et al. (2022)
**Glycosphingolipids and related glycans**			
Deuterium‐labelled acyl‐globotriaosylceramide	TOF (**CHCA**), FAB	Synthesis by transesterification of N_3_‐lyso GM3 with ^2^H_35_−18:0 acid *p*‐nitrophenyl ester for potential imaging of subcellular localization of GB3 using nanoSIMS	Aly and El Azab (2021)
6‐NH_2_‐α‐GalCer.	FT‐ICR (**DHB**, **CHCA**)	For construction of an antitumor vaccine of MUC1 glycopeptide and α‐GalCer *via* a gold‐nanoparticle delivery system.	Liu, Wang, Yu, et al. (2021)
Fluorescently labelled lacto‐series ganglioside	TOF (**CHCA**)	For single molecule imaging	Takahashi et al. (2022)
**Other glycolipids**			
2‐*O‐*Ac‐3,4,6‐tri‐*O*‐Ac‐α‐d‐Glc*p*‐(1→6)−1‐*O*‐(2‐oleoyl‐1‐stearoyl‐*sn*‐glycero‐3‐phosphonate)−2,3,4,5‐tetra‐*O*‐Ac‐d‐*myo*‐inositol	MALDI	Glycosylphosphatidylinositol oligosaccharide intermediate	Guerrero et al. (2021)
Poly‐amido‐saccharides containing myristoyl, palmitoyl, or stearoyl terminal chains	TOF	As water‐soluble biosurfactants	Sockett et al. (2022)
**Glycosides and related compounds**			
Propargyl‐(oligo)‐mannopyranoside	TOF	Intermediate in the synthesis of mannose‐based surfactants	Argudo, Spitzer, Ibarboure, et al. (2022)
6’’‐*O*‐Lauroyl‐1‐kestose and 6’’’‐*O*‐lauroylnystose	TOF (**DHB**)	Regioselective synthesis by sequential enzymatic reactions of transfructosylation and acylation	Campos‐Valdez et al. (2022)
Pixatimod (PG545), a sulfated oligosaccharide‐steroid conjugate	Spiral‐TOF (**DHB**, ‐ve)	Development of improved synthetic routes	Chhabra et al. (2021)
2 and 6‑*S*‑Hexyl‑β‑d‐glucopyranose S‑linked maleimide	TOF (**DHB**)	For study of a UDP‑glucose, cereblon‑dependent proinsulin degrader	Cho, Miyagawa et al. (2022)
Modified QS‐21 glycoside	MALDI	Replacing the rhamnose‐xylose moiety with simpler terminal disaccharide units attenuates adjuvant activity in truncated saponin variants	Fuentes, Ruiz‐de‐Angulo, et al. (2021)
Rutin polyglucoside	TOF (**DHB**)	Addition of (Glc)_n_ catalyzed by a cyclodextrin glucanotransferase to increase solubility	González‐Alfonso et al. (2021)
Glycosylated polyene macrolides labelled with 3,6‐di‐2‐pyridyl‐1,2,4,5‐tetrazine	TOF	To investigate antifungal action by sterol sponge mechanism	Guo, Zhang, et al. (2021)
β‐*C*‐glycoside‐2‐aminoundecanes	*R*‐TOF (**DHB**)	From glucose, lactose, and maltose. For use in personal care and cleaning products	Jackson et al. (2021)
Glucosyl‐α‐(1→6)‐mangiferin	TOF	For enhancement of water solubility and antioxidant capacities of mangiferin	Lee, Kim, Moon, et al. (2022)
Flavonoid glycosides, oroxins C and D from the seeds of *Oroxylum indium*	MALDI	Concise synthesis and antidiabetic activity	Li, Wang, Tong, et al. (2021)
2‐Deoxyiminosugar *C*‐glycosides	TOF/TOF (**DHB**, **DCTB**)	Stereocontrolled synthesis and evaluation as glycosidase inhibitors	Lumbroso et al. (2021)
Fisetin‐ 4’‐*O*‐α‐d‐glucopyranoside	TOF (**DHB**)	Synthesised with dextransucrase from *Leuconostoc mesenteroides*	Moon et al. (2022)
Transglycosylated mogrosides (steroid glycosides) from *Siraitia grosvenorii*, (Luo Han Guo fruit)	TOF **(DHB**), LC/MS	High‐yield synthesis improves the flavor profile of monk fruit extract sweeteners	Muñoz‐Labrador et al. (2021)
Galactooligosaccharide‐ modified mogrosides	TOF (**DHB**)	As new sweeteners	Muñoz‐Labrador et al. (2022)
Maltoheptaose‐palmitate ester	TOF/TOF (**DHB**)	Synthesis of a sugar ester having excellent emulsifying properties	Nguyen et al. (2021)
Acetoglucose‐substituted methacrylate	TOF	Intermediate in the synthesis of carbohydrate‐based block copolymer micelles for photodynamic therapy	Park, Jung, et al. (2021)
*Bis*‐glucosides	TOF/TOF (**DCTP**)	Synthesis and characterization of a small library of *bis*‐glucosides	Patry et al. (2021)
Thio‐ether functionalized glycolipids	TOF/TOF (**dithranol**, **CHCA**)	Synthesis and use to reveal a potent activator of SK3 channel with vasorelaxation effect	Sevrain et al. (2021)
Schaftoside	TOF	Total chemical synthesis in 11 steps (8.83% yield)	Shang et al. (2021)
Triterpene glycoside from *Eupentacta fraudatrix*	TOF	In study of structure‐activity relationships of holothuroid's triterpene glycosides	Zelepuga et al. (2021)
**Cyclodextrins (CDs)**			
6^I^–*O*‐Allyl‐γ‐CD	TOF	For synthesis of γ‐CD poly (glycidyl‐co‐ethylene dimethacrylate) for host‐guest interactions for extracting antibiotics	Belenguer‐Sapiña et al. (2021)
β‐CD + (PhCHO)_7_	TOF/TOF	For preparation of glycopeptide dendrimers:	Bi et al. (2022)
Betulinic acid‐CD conjugates	TOF/TOF	For inhibition of influenza infection	Chen, Wang, Ma, et al. (2022)
Heptakis‐6‐octanethio‐β‐CD (CD‐C8) and CD‐C12	TOF (**DCTB**)	Amphiphilic CD‐based nanoparticulate vaccines shown to trigger T‐cell immune responses	Geisshüsler et al. (2022)
Heptavalent glycyrrhetinic acid β‐CD conjugates	TOF/TOF (**CHCA**)	Synthesis, characterization, and anti‐influenza activity	Liang, Ma, et al. (2022)
BODIPY‐modified β‐CD	TOF	For use in fluorescence sensing of glutathione thiyl radical	Liu, Dai, et al. (2022)
Oligopeptide‐decorated amphiphilic CD nanomagnet intermediates	TOF/TOF	For selective amyloid beta recognition and fishing	Mazzaglia et al. (2022)
γ‐CD–Fullerene	TOF	Synthesis of amphiphilic γ‐CD–fullerene complexes with photodynamic activity	Miki et al. (2022)
Peptide/BODIPY‐modified per‐*O*‐methyl‐β‐CDs	TOF	For FRET‐based in‐cell detection of highly selective supramolecular complexes of *meso*‐tetraarylporphyrin	Nakagami et al. (2021)
β‐CD‐Fluvastatin conjugates	TOF/TOF (**DHB**)	Synthesis and biological evaluation as potential therapeutics for neuronal disorders such as Alzheimer's and Niemann Pick type C disease	Nicolosi et al. (2021)
Lactose‐appended hydroxypropyl‐β‐CD	TOF	Shown to lower cholesterol accumulation and alleviate motor dysfunction in Niemann−Pick type C disease model mice	Nishida et al. (2022)
CD‐oligocaprolactone derivatives	TOF/TOF (**DHB**, **CHCA**)	Synthesis and structural characterization by MALDI‐MS/MS	Peptu et al. (2022)
Sulfur‐bridged β‐CD dimers	TOF	For enantiodifferentiating photocyclodimerization of 2‐anthracenecarboxylate. Protocol	Wei et al. (2022)
α‐, β‐, and γ‐Ureido‐CDs	TOF	Synthesis of upper critical solution temperature‐type responsive cyclodextrins	Zhu, Liu, et al. (2021)
**Rotaxanes**			
Pillar[5]arene‐based polycationic glyco[2]rotaxanes	*R*‐TOF/TOF (**DCTB**)	As *Pseudomonas aeruginosa* antibiofilm agents	El Dine et al. (2021)
Polyfluorene/permodified CD polyrotaxanes	TOF/TOF (**DCTB**)	Synthesis, photophysics and production of Langmuir films	El Haitami et al. (2021)
**Polymers**			
Branched glycopolymer–pyropheophorbide‐a conjugate	IT‐TOF (**DHB**, **CHCA**)	For cancer chemotherapy	Duan et al. (2021)
Sulfur‐linked sugar polymers	*R*‐TOF/TOF	Chemoenzymatic synthesis. As heparanase inhibitors	He, Zhang, et al. (2022)
Sucrose‐1,6‐hexamethylene diisocyanate polymer	TOF/TOF (**DHB**)	For synthesis of novel polyurethane networks	Lakatos et al. (2022)
Glycosylated poly(ethylene oxide)–poly(propylene oxide)	TOF	For preparation of glucosylated polymeric micelles to actively target breast cancer	Lecot et al. (2021)
Polymers from d‐ and l‐xylose	TOF	Synthesis with control of crystallinity and stereocomplexation	McGuire, Bowles, et al. (2021)
Maltotriose‐*b*‐poly(*N*‐*n*‐propylglycine	TOF (**CHCA**)	For construction of permeable polymer vesicles	Okuno et al. (2021)
Fructo‐oligosaccharides/inulin polymers	TOF (**DHB**)	For preparation of nanodisks	Ravula and Ramamoorthy (2021)
**Antibiotics and other drugs**			
Teicoplanin derivative	*R*‐TOF (**DHB**)	Synthesis of the first dimeric derivatives	Bereczki et al. (2022)
Vancomycin derivative	TOF	Synthesis of novel semisynthetic antibiotics active against *Staphylococcus aureus* biofilms and cells in late stationary growth phase	Vimberg et al. (2021)
**Microarrays, Nanoparticles**			
Trehalose‐based nanoparticles	*L*‐TOF/TOF (**DHB**, **DHAP**)	For organ‐selective gene delivery	Carbajo‐Gordillo et al. (2021)
β‐CD‐poly (β‐amino ester) nanoparticles	TOF/TOF (**DHB**)	Nanoparticles for high loading and sustained release of histone deacetylase inhibitors	Chaudhuri et al. (2021)
Controlled density glycodendron microarrays	TOF	For studying carbohydrate–lectin interactions	Di Maio et al. (2021)
Doublecortin like kinase 1 (DCLK1) antibody functionalized folic acid conjugated hesperetin encapsulated chitosan nanoparticles	TOF/TOF	For targeting colon cancer stem cells	Lazer et al. (2022)
*N*‐Acetylgalactosamine‐decorated nanoliposomes	TOF/TOF	For targeted delivery of paclitaxel to hepatocellular carcinoma	Li, Yu, et al. (2021)
Glycans linked to glycerophosphate	QIT‐TOF (**THAP**)	Synthesis of noncovalent microarrays from synthetic amino‐terminating glycans	Li, Palma, et al. (2021)
Sialylated solid lipid microparticles	TOF	As inhibitors of influenza A virus infection	Richard et al. (2022)
TLR7 Ligands on gold nanoparticles	TOF/TOF	The resulting glyco‐nanoadjuvants shown to affect their immunostimulatory activities	Shinchi et al. (2021)
Manα1→6Glc‐nanoparticles	TOF/TOF	For study of the effect of linker length for conjugating a synthetic small‐molecule TLR7 ligand to glyco‐nanoparticles on immunostimulatory effects	Shinchi et al. (2022)
Xylan microparticles	TOF (**DHB**)	Enzymatic synthesis of xylan microparticles with tunable morphologies	Smith, Curry, et al. (2022)
Gold nanoparticles with lactose	TOF/TOF (**DHB**)	For investigations of affinity labelling for target protein analysis	Suto et al. (2021)
Sugar‐coated hierarchical platinum nanostructures	TOF/TOF (**DHB**)	Method to support and heterogenize nanoparticles	Woitassek et al. (2022)
Peptide‐CD nanoparticles	TOF/TOF	Effect and mechanism of action on hepatoma of nanoparticles loaded with tyroserleutide	Wu, Hua, et al. (2021)
**Miscellaneous**			
Mono‐, di‐, and trivalent α‐d‐mannopyranosyl conjugates	MALDI (**CHCA**)	On an aromatic scaffold. Synthesis and hemagglutination inhibitory properties	Al‐Mughaid and Khazaaleh (2021)
Almost‐linear mannose polysaccharides linked to an oleic or ricinoleic acid	TOF/TOF (**DHB**)	Design and self‐assembly of sugar‐based amphiphiles: Spherical to cylindrical micelles. Use of click chemistry	Argudo, Spitzer, Jerome, et al. (2022)
Liposomes displaying glycan ligands	TOF	Increasing phagocytosis of micoglia by targeting CD33 with product	Bhattacherjee et al. (2021)
Triazolylisatins glycoconjugates	TOF/TOF	Use of click reaction of propargylisatins with some azido‐sugars	Bogdanov et al. (2021)
Glucosylated 5‐hydroxymethyl‐pyrimidines	TOF	As epigenetic DNA bases regulating transcription and restriction cleavage	Chakrapani, Ruiz‐Larrabeiti, et al. (2022)
Galactose‐modified multifunctional nanoprobe	TOF	For cancer therapy based on nitric oxide prodrug delivery and release	Dang et al. (2021)
*S*‐Linked sugar‐nucleotide analogues	*R*‐TOF (**DHB**)	Synthesis of potential glycosyl transferase inhibitors by thio‐click reactions	Debreczeni et al. (2021)
Porphyrin glycoconjugates	TOF (**CHCA**)	Synthesis and evaluation of porphyrin glycoconjugates varying in linker length and preliminary effects on the photodynamic inactivation of *Mycobacterium smegmatis*	Dixon et al. (2021)
DSPE‐PEG (2000)‐GalNAc	*R*‐TOF/TOF	For construction of liposome‐GalNAc nanoparticles for hepatocellular carcinoma chemotherapy	Farinha et al. (2021)
Glycan‐oligonucleotide conjugates.	TOF (**3‐HPA**)	For preparation of glycan chips for on‐chip biosynthesis of cancer‐associated complex glycans	Heo et al. (2021)
Glycocholic acid‐chitosan oligosaccharide conjugate	TOF	For oral administration of chemotherapeutic drugs	Liu, Han, et al. (2022)
Lactose‐functionalized dimeric camptothecin	TOF	For targeted and fluorescence imaging‐guided chemo‐photodynamic therapy	Ma, Shi, et al. (2022)
Guanosine diphosphate l‐fucose	TOF/TOF (**9‐AA**)	*In vitro* synthesis using multi‐enzyme cascades	Mahour et al. (2021)
*N*,*N*‐*Bis*(hexyl α‐d‐acetylmannosyl) acrylamide	TOF (**DHB**)	Synthesised as a by‐product of the monomer	Miyagawa et al. (2021)
Sugar‐polyolefin conjugates	MALDI	For synthesis of stable thermotropic 3D and 2D double gyroid nanostructures with sub‐2‐nm feature size	Nowak et al. (2021)
Oxazolidine boronate sugar complexes	TOF	In construction of fluorescent sensor array for quantitative determination of saccharides	Pushina et al. (2021)
Carbohydrate‐attached fullerene derivative	TOF	For selective localization in ordered carbohydrate‐block‐poly(3‐hexylthiophene) nanodomains	Sakai‐Otsuka et al. (2021)
Mannosylated‐calix[4]arene	TOF	Dynamic self‐assembly into micelles for the delivery of hydrophobic drugs	Sreedevi et al. (2021)
Cholesterol‑undecanoate‐glucose conjugate	TOF	For the treatment of cerebral malaria	Tian, Zheng, et al. (2022)
α‑Dystroglycan mucin type core m1 (glyco)peptide library	TOF/TOF (**DHB**)	Exploring the *in situ* pairing of human galectins toward synthetic *O*‑mannosylated core M1 glycopeptides of α‑dystroglycan	Villones et al. (2022)
^64^Cu‐Containing carbohydrate fluorescence biomarker	TOF/TOF	Fluorescence marker for improved surgical precision	Wang, Hansen, et al. (2021)
Cyanidin‐3‐*O*‐glucoside and β‐lactoglobulin conjugate	TOF	Effect of ultrasound on binding interaction and functional properties	Wang, Wang, Luo, et al. (2022)
Glucose−lipopeptide conjugates	TOF	Conjugates reveal the role of glucose modification position in complexation and the potential of malignant melanoma therapy	Zhao, Zhang, Li, et al. (2021)
Disaccharide oxazolines carrying four or six azide tags	*R*‐TOF (**DHB**)	Chemoenzymatic method for glycan‐mediated site‐specific labeling and conjugation of antibodies	Zhang, Ou, et al. (2021)

^a^ Format (not all items present): MALDI method (**matrix**), “MALDI” is used when the instrument is not specified.

A number of other papers, listed in Table 47, report more general methods and a few report work on synthetic mechanisms (Table 48).

**Table 47:** Use of matrix‐assisted laser desorption/ionization‐mass spectrometry to study methods for general synthesis.

Method/notes	Methods^a^	References
Bifidobacterial β‐galactosidase‐mediated production of galacto‐oligosaccharides	TOF (**DHB**)	Ambrogi et al. (2021)
Facile synthesis of per(6‐*O*‐tertbutyldimethylsilyl)‐α‐, β‐, and γ‐cyclodextrin as protected intermediates for the functionalization of the secondary face of the macrocycles	TOF	Benkovics et al. (2021)
Site‐selective attachment of polymer chains to glycoproteins by sodium periodate oxidation of glycans (shown for HRP)	TOF/TOF	Bi, Xiong, et al. (2021)
MALDI mass spectrometry monitoring of cyclodextrin‐oligolactide derivatives synthesis	TOF, TOF/TOF (**CHCA**, **DHB**)	Blaj et al. (2021)
Diisobutyl aluminum hydride promoted debenzylation of **α**‐cyclodextrin	MALDI	Bols and Friis (2022)
Synthesis of poly‐β‐1,4‐glucan derivatives by use of promiscuous glycosyltransferase	TOF	Bulmer et al. (2021)
Conformation‐controlled hydrogen‐bond‐mediated aglycone delivery method for α‑xylosylation	TOF/TOF	Cai, Bian, et al. (2021)
A mild glycosylation protocol with glycosyl 1‐methylimidazole‐2‐carboxylates as donors	MALDI	Chen, Tang, et al. (2021)
High‐quality palladium on carbon catalysts for hydrogenolysis—use with serotype A decasaccharide	TOF	Crawford et al. (2021)
Endo‐M mediated chemoenzymatic approach enables reversible glycopeptide labeling for *O*‐GlcNAcylation analysis	TOF	Chen, Tang, et al. (2022)
Enhanced binding and reduced immunogenicity of glycoconjugates prepared *via* solid‐state photoactivation of aliphatic diazirine carbohydrates	TOF	Congdon and Gildersleeve (2021)
Solid‐phase synthesis of polysaccharides from unprotected glucose catalyzed by H*β* zeolites	TOF/TOF (**DHB**)	Dong, Xiao, et al. (2022)
Controlled depolymerization of cellulose by photoelectrochemical bioreactor using a lytic polysaccharide monooxygenase	TOF	Gao, Zhang, et al. (2022)
Phosphine‐mediated three‐component bioconjugation of amino‐ and azidosaccharides in ionic liquids	MALDI	Hall et al. (2022)
Synthesis of unnatural cyclodextrins with cyclodextrin glucanotransferase	MALDI	Larsen, Ferreira et al. (2022)
Insights into the synergistic effect of catalyst acidity and solvent basicity for effective production of pentose from glucose	TOF	Li, Lin, et al. (2022)
Iterative synthesis of 2‐deoxyoligosaccharides enabled by stereoselective visible‐light‐promoted glycosylation	TOF/TOF	Liu, Wang, Guo, et al. (2022)
*In vitro* glycosylation of membrane proteins using *N*‑glycosyltransferase	TOF (**CHCA**)	Liyanage, Harris, et al. (2021)
Sustainable polyesters *via* direct functionalization of lignocellulosic sugars	TOF	Manker et al. (2022)
Ring‐opening copolymerization of a d‑xylose anhydrosugar oxetane to produce polymers from sugars and cyclic anhydrides	TOF	McGuire, Clark, et al. (2021)
Precursors of iminosugars with 7‐membered rings	Q‐TOF/TOF	Osuch‐Kwiatkowska and Jarosz (2022)
β‐1,3‐Glucan synthesis, novel supramolecular self‐assembly, characterization and application	TOF	Pylkkänen et al. (2022)
Design, synthesis, and characterization of stapled oligosaccharides	TOF	Ricardo et al. (2022)
Solid‐phase synthesis of glucosyl glycopeptides from synthesised amino acid derivatives: Optimization of the synthetic route	TOF	Rodríguez et al. (2021)
Phosphorylase‐catalyzed synthesis and self‐assembled structures of cellulose oligomers in the presence of protein denaturants	TOF (**DHB**)	Sakurai et al. (2022)
Enzyme‐catalyzed propagation of cello‐oligosaccharide chains from bifunctional oligomeric primers for the preparation of block co‐oligomers and their crystalline assemblies	*L*‐TOF (**DHB**)	Sugiura et al. (2021)
Catalytic, regioselective sulfonylation of carbohydrates with dibutyltin oxide under solvent‐free conditions	MALDI	Traboni et al. (2021)
Introducing hyaluronic acid into supramolecular polymers and hydrogels	TOF (**CHCA**, **DCTB**)	Varela‐Aramburu et al. (2021)
Synthesis and characterization of regioselectively functionalized mono‐sulfated and ‐phosphorylated anionic poly‐amido‐saccharides	*R*‐TOF (**aminoacridine**)	Varghese et al. (2022)
Per‐glycosylation of the surface‐accessible lysines: One‐pot aqueous route to stabilized proteins with native activity	TOF (**SA**)	Walther et al. (2021)
Chemoenzymatic modular assembly of *O*‐GalNAc glycans for functional glycomics	*R*‐TOF/TOF	Wang, Chen, et al. (2021)
Synergistic enzyme cocktail between levansucrase and inulosucrase for levan‐type fructooligosaccharide synthesis	TOF	Wangpaiboon, Klaewkla, et al. (2021)
Facile preparation of polysaccharide−polypeptide conjugates *via* a biphasic solution ring‐opening polymerization	TOF	Yang, Liu, et al. (2022)
Merging reagent modulation and remote anchimeric assistance for glycosylation: Highly stereoselective synthesis of α‐glycans up to a 30‐mer	TOF/TOF	Zhang, He, et al. (2021)
Production of water‐soluble sugar from cellulose and corn stover *via* molten salt hydrate impregnation and separation	TOF	Zhou, Liu, et al. (2022)
Automated assembly of starch and glycogen polysaccharides	TOF	Zhu, Delbiabco, et al. (2021)

^a^ Format (not all items present): MALDI method (**matrix**). “MALDI” used when instrument not specified.

**Table 48:** Use of matrix‐assisted laser desorption/ionization‐ mass spectrometry to study carbohydrate reactions.

Reaction	Methods	References
Regioselective reductive ring‐opening reactions of 4,6‑*O*‑halobenzylidene acetals of glucopyranosides	*R*‐TOF/TOF (**DHB**)	Mezö et al. (2021)
VaporSPOT: Parallel synthesis of oligosaccharides on membranes	TOF/TOF	Tsouka et al. (2022)
A study of the diisobutylaluminum hydride‐promoted selective debenzylation of α‐CD protected with two different benzyl groups	TOF	Yousefi et al. (2022)

## 15 MISCELLANEOUS STUDIES

A method for detection of ricin B by MALDI using lactosylated Fe_3_O_4_ magnetic nanoparticles has been developed and used to detect ricin B spiked into corn starch (Kandasamy et al., 2021). The nanoparticles were prepared by attaching lactose to the surface of aminated nanoparticles through the Maillard reaction and enrichment of ricin B took about 1 h by incubating the nanoparticles with samples under shaking at room temperature, followed by magnetic isolation. The limit of detection toward ricin B was about 3 nM.

The use of fluorescently labelled glycans has been advocated as a convenient method for the study of microbial degradation of glycans such as those pertaining to the gut microbiome. Acetylated galactoglucomannan from Norwegian spruce wood and acetylated (arabino) glucuronoxylan from Norwegian birch wood were used in the study with 2‐AB as the fluorescent label, chosen for its similar size to the monosaccharide constituents of the target sugars (Leivers et al., 2022). Monitoring of the labelling reaction was performed by MALDI and HPLC.

## 16 OTHER METHODS FOR GLYCAN AND GLYCOCONJUGATE ANALYSIS

As mentioned in the Introduction, this review also includes methods for the analysis of carbohydrates other than those based on MALDI analysis. Most of these methods could, however, be adapted for MALDI analysis. Several relevant reviews have been published as listed in Table 49.

**Table 49:** Reviews and general articles relating to methods other than matrix‐assisted laser desorption/ionization.

Subject	Comments	Citations	References
Capillary electrophoresis‐mass spectrometry of carbohydrates and glycoconjugates	MS interfacing, oligosaccharides and glycoconjugates	101	do Lago et al. (2021)
Reversed‐phase and hydrophobic interaction chromatography of carbohydrates and glycoconjugates	Comprehensive review. Some references to MALDI	333	El Rassi (2021b)
Carbohydrate analysis by modern liquid phase separation techniques, second edition	Book	‐	El Rassi (2021a)
Capillary electrophoresis and links with MALDI: Advances in *N*‐glycomics and glycoproteomics	Coupling of CE with MS and applications to glycan analysis	66	Makrydaki et al. (2021)
Advances in ion chromatography‐mass spectrometry (IC‐MS) for improved separation and analysis of carbohydrates	Mainly *O*‐glycans	43	Rumachik et al. (2021)

Addition of a dopant to the gas flow after separation in an LC/MS system has been shown to improve the signal strength in negative ion mode (Madunić et al., 2021). Isopropanol‐enriched gas was shown to greatly improve the detection of both *N*‐and *O*‐glycans and their MS/MS mass spectra, particularly for the early eluting species.

Monoclonal antibodies radiolabelled with positron emitting radionuclides incorporated by use of bifunctional chelators, are widely used in nuclear imaging. Three methods for assessment of the average functionalisation and heterogeneity of the conjugated mAbs, radiometric and photometric titrations, MALDI‐TOF‐MS and UPLC/ESI‐TOF MS, have shown that all three gave comparable results. MALDI/TOF MS provided equivalent results to those obtained by the radio‐ and photo‐metric titrations although investigation of the heterogeneity of the conjugates was challenging and inaccurate, whereas UPLC/ESI‐TOF gave good peak resolution but was unable to discriminate between different smaller conjugates (Feiner et al., 2021).

Meyer, Montero, et al. (2022) have compared four common chromatographic methods (SFC, HILIC, RP‐LC, and GC) with detection by triple quadrupole mass spectrometer for analysis of monosaccharides. They showed that GC and RP‐LC, with suitable derivatization, are superior to the other two methods in terms of separation performance. Overall, RP‐LC–MS in MRM mode after derivatization with PMP gave the best separation, sensitivity and repeatability.

Wang, Wang, Wu, et al. (2022) have investigated several hydroxylamine reagents for analysis of monosaccharides and found that *O*‐(4‐methoxybenzyl)‐hydroxylamine hydrochloride gave the best results. Twelve monosaccharides were readily detected although not all were fully separated by HPLC. The d_3_ analogue of the derivatization reagent was also synthesised and used for quantitative studies.

Other methods are listed in Table 50.

**Table 50:** Other methods for glycan and glycoconjugate analysis.

Method	Compound type	Main technique	References
Capillary (gel) electrophoresis‐based methods for immunoglobulin glycosylation analysis	IgG Glycosylation	CE and CE‐MS	Cajic et al. (2021)
Exploiting *pglB* oligosaccharyltransferase‐positive and –negative *Campylobacter jejuni* and a multiprotease digestion strategy to identify novel sites modified by *N*‑linked protein glycosylation	Glycoproteins	LC‐FAIMS‐MS/MS	Cain et al. (2021)
A multidimensional mass spectrometry‐based workflow for *de novo* structural elucidation of oligosaccharides from polysaccharides	Polysaccharides	LC‐MS/MS, UHPLC‐QqQ‐TOF	Castillo et al. (2021)
Methods to improve quantitative glycoprotein coverage from bottom‐up LC‐MS data	Glycoproteins	LC‐MS	Chang and Zaia (2022)
Characterization of galacto‐oligosaccharides using high‐performance anion exchange chromatography‐tandem mass spectrometry	Galacto‐oligosaccharides	HPAEC‐MS/MS	Chen and Liu (2021)
High‐throughput analyses of glycans, glycosites, and intact glycopeptides using C4‐and C18/MAX‐tips and liquid handling system	Glycans, glycosites, and intact glycopeptides	Protocol, unspecified mass spectrometry	Chen, Clark, et al. (2021)
Resolving structural detail and occupancy of glycans on intact glycoproteins	*N*‐ and *O*‐glycans on glycoproteins	HPLC, LC‐MS, exoglycosidase	Chen, Wu, et al. (2021)
Mirror‐cutting‐based digestion strategy enables the in‐depth and accuracy characterization of *N*‑linked protein glycosylation	*N*‐Glycopeptides	LC‐MS/MS	Chen, Fang, et al. (2021)
Targeted *N* **‑**glycan analysis with parallel reaction monitoring using a quadrupole‐Orbitrap hybrid mass spectrometer	*N*‐Glycans (per‐Me)	LC/MS/MS	Cho, Reyes, et al. (2022)
Desalting paper spay mass spectrometry for rapid detection of glycans and glycoconjugates	Glycans and glycoconjugates	Paper spray	Chiu et al. (2021)
In‐depth profiling of *O*‑glycan isomers in human cells using C18 nanoliquid chromatography‐mass spectrometry and glycogenomics	*O*‑Glycan isomers	LC/MS (2‐AB derivatives)	de Haan, Narimatsu, et al. (2022)
Data‐independent acquisition‐based mass spectrometry for quantitative analysis of intact *N*‐linked glycopeptides	*N*‐Glycopeptides	LC‐MS/MS	Dong et al. (2021)
Immobilized exoglycosidase matrix mediated solid phase glycan sequencing	*N*‐Glycans	CE, exoglycosidase digestion	Farsang et al. (2022)
Mesoporous graphitized carbon column for efficient isomeric separation of permethylated glycans	*N*‐Glycans	LC‐MS (LTQ Orbitrap)	Gautam et al. (2021)
Glycine additive shown to enhance sensitivity for *N*‐ and *O*‐glycan analysis with HILIC‐ESI‐MS	*N*‐ and *O*‐glycans	HILIC‐ESI‐MS	Guo, Nayak, et al. (2021)
Fast and ultrasensitive glycoform analysis by supercritical fluid chromatography−tandem mass spectrometry	*N*‐Glycans (per‐Ac)	SFC‐MS (main) also HPLC, MALDI‐TOF/TOF	Haga, Yamada, et al. (2022)
In‐source microdroplet derivatization using coaxial contained‐electrospray mass spectrometry for enhanced sensitivity in saccharide analysis	Oligosaccharides	ESI	Heiss and Badu‐Tawiah (2021)
Liquid chromatography−tandem mass spectrometry with online, in‐source droplet‐based phenylboronic acid derivatization for sensitive analysis of saccharides	Oligosaccharides	LC/MS	Heiss and Badu‐Tawiah (2022)
Fc glycosylation characterization of human immunoglobulins G using immunocapture and LC‐MS	*N*‐glycans from serum or plasma IgG, Fc	LC‐MS, protocol	Helali et al. (2021)
*N*‐glycan profiling of glycoproteins by hydrophilic interaction liquid chromatography with fluorescence and mass spectrometric detection	*N*‐Glycans	HILIC‐HPLC, LC‐MS/MS, video of protocol	Kayili and Salih (2021)
High‐throughput *N*‐glycan screening method for therapeutic antibodies using a microchip‐based DNA analyzer	*N*‐Glycans from monoclonal antibodies	CE‐MS of ANTS‐labelled *N*‐glycans	Kinoshita, Nakajima, et al. (2021)
Development of a novel, label‐free *N*‐glycan method using charged aerosol detection	*N‐*Glycans	HPLC without fluorescent derivatization	Knihtila et al. (2022)
Separation of glycoproteins using novel stationary phases modified with poly(ethylene glycol)‐conjugated boronic‐acid derivatives	Glycoproteins	HPLC	Kobayashi et al. (2022)
Characterization of protein glycoforms at intact level by Orbitrap mass spectrometry	Glycoproteins	LC‐MS (Orbitrap), protocol	Kristensen et al. (2021)
Capillary electrophoresis‐based *N*‐glycosylation analysis in the biomedical and biopharmaceutical fields	*N*‐Glycans	CE	Kun et al. (2021)
Separation and identification of permethylated glycan isomers by reversed phase nano‐LC‐NSI‐MS^n^	Oligosaccharides, *N*‐glycans	LC‐ESI‐MS^n^	Kurz et al. (2021)
Cross‐identification of *N*‐glycans by CE‐LIF using two capillary coatings and three labeling dyes	*N*‐glycans	CE‐LIF	Li, Wang, Guo, et al. (2022)
High sensitivity capillary electrophoresis with fluorescent detection for glycan mapping	Glucose oligomers, *N*‐glycans	CE with fluorescence	Liénard–Mayor et al. (2021)
Lab‐in‐droplet: From glycan sample treatment toward diagnostic screening of congenital disorders of glycosylation	*N*‐Glycans	CE with fluorescence	Liénard–Mayor et al. (2022)
High sensitivity acidic *N*‐glycan profiling with MS‐enhancing derivatization and mixed mode chromatography	*N*‐Glycans	LC/MS, charged derivatives (RapiFlour)	Liu, Wang, Lauber, et al. (2022)
Distinguishing carbohydrate isomers with rapid hydrogen/deuterium exchange‐mass spectrometry	Trisaccharides	ESI‐Orbitrap	Liyanage, Quintero, et al. (2021)
High‐sensitivity glycan profiling of blood‐derived IgG, plasma, and extracellular vesicle isolates with CZE‐MS	*N*‐Glycans	CZE‐ESI‐MS	Marie et al. (2021)
HILIC‐UPLC‐MS for high throughput and isomeric *N*‐glycan separation and characterization in CDGs and human diseases	*N*‐Glycans	HILIC‐UPLC‐ESI‐MS	Messina, Palmigiano, Esposito, et al. (2021)
Liquid chromatography and capillary electrophoresis in glycomic and glycoproteomic analysis	*N*‐Glycans	LC and CE	Molnarova et al. (2022)
Polysaccharide identification through oligosaccharide fingerprinting	Polysaccharides	HPLC‐QTOF	Nandita et al. (2021)
An improved method for galactosyl oligosaccharide characterization	Galactosyl oligosaccharides	HPAEC‐ESI‐MS	Patil and Rohrer (2021)
Lectin and liquid chromatography‐based methods for immunoglobulin glycosylation analysis	*N*‐ and *O*‐glycans	LC‐MS, Lectin chromatography, lectin microarrays	Petrović and Trbojević‐Akmačić (2021)
Nanoflow LC−MS method allowing in‐depth characterization of natural heterogeneity of complex bacterial lipopolysaccharides	Complex bacterial lipo‐polysaccharides	Nano‐LC‐MS	Pupo et al. (2021)
2‐Dimensional ultrahigh performance liquid chromatography and methyl ester formation paired with tandem mass spectrometry for comprehensive serum *N*‐glycome characterization	*N*‐Glycans	Weak anion exchange (WAX) and HILIC chromatography‐MS/MS	Smith, Millán‐Martín, et al. (2021)
*N*‑glycomics of various tissue samples that may contain glycans with unknown or unexpected structures	*N*‐Glycans (2‐AP derivatives)	LC‐MS, exoglycosidase digestions.	Suzuki et al. (2021)
*N* **‑**Glycan isomer differentiation by zero flow capillary electrophoresis coupled to mass spectrometry	*N*‐Glycans	Zero‐flow CE	Wagt et al. (2022)
Streamlined subclass‐specific absolute quantification of serum IgG glycopeptides using synthetic isotope‐labeled standards	Glycopeptides	Nano‐LC‐MS	Wang, Liu, Qu, et al. (2021)
Improving the sensitivity for quantifying heparan sulfate from biological samples	Heparan sulfate	LC‐MS/MS (AMAC label)	Wang, Dhurandhare, et al. (2021)
A LC‐MS/MS method to simultaneously profile 14 free monosaccharides in biofluids	Monosaccharides	LC‐MS	Wang, Zhang, Peng, et al. (2022)
Paired derivatization approach with H/D‐labeled hydroxylamine reagents for sensitive and accurate analysis of monosaccharides by liquid chromatography tandem mass spectrometry	Monosaccharides	LC‐MS/MS	Wang, Wang, Wu, Cai, et al. (2022)
High‐sensitivity glycoproteomic analysis of biological samples by CZE‐ESI‐MS ‐ Protocol	*N*‐Glycans	CE‐ESI‐MS	Wang and Lageveen‐Kammeijer (2022)
Carbon fiber paper spray ionization mass spectrometry. Said to give stronger spectra than MALDI for glycans	Glycans	Paper spray	Wang, Bai, et al. (2022)
A versatile strategy for high‐resolution separation of reducing glycan mixtures as hydrazones by two‐dimensional high‐performance liquid chromatography	Glycans (*N*‐glycans) (per‐Me)	HPLC, ESI‐MS/MS, MALDI	Wang, Gao, et al. (2022)
High‐throughput and high‐sensitivity *N*‐glycan profiling: A platform for biopharmaceutical development and disease biomarker discovery	*N*‐Glycans	HPLC	Xie, Mota, et al. (2021)
HPLC separation and preparative conditions for 8‐aminopyrene‐1,3,6‐trisulfonic acid‐labeled *N*‐glycans using a hydrophilic interaction column	*N*‐Glycans	HPLC	Yamamoto et al. (2022)
Glycan mapping of low‐molecular‐weight heparin using mass spectral correction based on chromatography fitting with “Glycomapping” software	Low‐molecular‐weight heparin	LC‐Q‐TOF	Yan et al. (2022)
Improved online LC‐MS/MS identification of *O*‐glycosites by EThcD fragmentation, chemoenzymatic reaction, and SPE enrichment	*O*‐Glycosites	EThcD Fragmentation, chemoenzymatic reaction, and SPE enrichment	Yang, Wang, et al. (2021)
In capillary labeling with APTS and online electrophoretic separation of *N*‐glycans from glycoproteins	*N‐*Glycans	CE	Yang, Mai, et al. (2022)
Capillary zone electrophoresis‐electrospray ionization tandem mass spectrometry for total analysis of chondroitin/dermatan sulfate oligosaccharides. ‐ Protocol	Chondroitin/dermatan sulfate oligosaccharides	CE‐ESI‐MS	Zamfir (2022)
Routine analysis of *N*‐glycans using liquid chromatography coupled to routine mass detection	*N*‐Glycans	LC‐MS, protocol	Zhang, Vimalraj, et al. (2021)
GlycoHybridSeq: Automated identification of *N*‑linked glycopeptides using electron transfer/high‐energy collision dissociation (EThcD)	*N*‐Glycans	Using electron transfer/high‐energy collision dissociation	Zhang, Zhu, et al. (2021)
Fractionation and characterization of sialyl linkage isomers of serum *N*‐glycans by CE‐MS	*N*‐Glycans	CE‐MS	Zhou, Song, et al. (2022)

## 17 REPORT OF RETRACTIONS

The paper by Zhao et al. (2014), reported in the review (Harvey, 2018); has been retracted. The paper “Discrimination of urinary exosomes from microvesicles by lipidomics using thin layer liquid chromatography (TLC) coupled with MALDI‐TOF mass spectrometry” by Singhto et al. (2022) has also been retracted because the mass tolerance was too large and the ion mode was at times inappropriate for the lipid species analysed.

## 18 CONCLUSIONS

MALDI continues to be a major technique for the analysis of carbohydrates and glycoconjugates with its advantage of speed and production of mainly singly charged ions. Thus, unlike ESI, the technique allows profiles of mixtures to be reproduced accurately. Although the rapid year‐by‐year increase in the number of publications reported in earlier reviews appears to have slowed somewhat, largely as the result of more analyses being conducted by LC‐MS, new methods and applications continue to appear. Growth areas are in applications to clinical practice for biomarker discovery, and particularly MALDI imaging with many new methods and matrices being reported. The incorporation of ion mobility into glycan assays, particularly for isomer separation is another growing area, and the use of linkage‐specific sialic acid derivatization, now appears in a large number of publications. The next review in this series (period from 2023 to 2024) will probably see a continuation of the trends reported above, particularly in the areas of MALDI imaging and incorporation of ion mobility into glycan analysis.

## AUTHOR CONTRIBUTIONS

**David J Harvey**: Conceptualization; Project administration; Writing—original draft; Writing—review & editing.

## CONFLICT OF INTEREST STATEMENT

The author declares no conflict of interest.
